# Large-scale mass spectrometry-based analysis of *Euplotes octocarinatus* supports the high frequency of +1 programmed ribosomal frameshift

**DOI:** 10.1038/srep33020

**Published:** 2016-09-06

**Authors:** Ruanlin Wang, Zhiyun Zhang, Jun Du, Yuejun Fu, Aihua Liang

**Affiliations:** 1 Key Laboratory of Chemical Biology and Molecular Engineering of Ministry of Education, Institute of Biotechnology, Shanxi University, Taiyuan 030006, China

## Abstract

Programmed ribosomal frameshifting (PRF) is commonly used to express many viral and some cellular genes. We conducted a genome-wide investigation of +1 PRF in ciliate *Euplotes octocarinatus* through genome and transcriptome sequencing and our results demonstrated that approximately 11.4% of genes require +1 PRF to produce complete gene products. While nucleic acid-based evidence for candidate genes with +1 PRF is strong, only very limited information is available at protein levels to date. In this study, *E. octocarinatus* was subjected to large-scale mass spectrometry-based analysis to verify the high frequency of +1 PRF and 226 +1 PRF gene products were identified. Based on the amino acid sequences of the peptides spanning the frameshift sites, typical frameshift motif AAA-UAR for +1 PRF in *Euplotes* was identified. Our data in this study provide very useful insight into the understanding of the molecular mechanism of +1 PRF.

Protein translation is an essential life process, in which both efficiency and fidelity are important. A ribosome translates the triplet genetic codes on an mRNA into amino acids of a protein in high fidelity^1^ which requires the strict maintenance of in-frame reading of the genetic codes of the mRNA. As mRNA shifts, a truncated and nonsense protein will be produced, resulting in an increase in the energetic cost of translation, and additional loads for cellular cleanup and quality control machineries^2^. However, in special cases, the translating ribosome can switch from the initial (0) reading frame to a −1 or +1 reading frame at a specific position, and then continues its translation^3^. This process is called programmed ribosomal frameshifting (PRF). PRF commonly occurs in all life forms, including bacteria and eukaryotes^4^,^5^,^6^.

Researchers have found two main PRFs (−1 and +1) in viruses and other cellular organisms. The −1 PRF occurs frequently in viruses and other cellular organisms with well documented roles. In viruses and bacteria, for example, such frameshifting is used to expand the information content of their genomes^7^,^8^. Recent results have implicated the roles of −1 PRF in quality control of mRNA and DNA stability in eukaryotes^9^,^10^. Unlike −1 PRF, +1 PRF are less frequently found in bacteria, fungi, mammals and ciliated protozoa of *Euplotes*. In bacteria, +1 PRF is used as a sensor and effector of an autoregulatory circuit to express release factor 2^11^,^12^. In mammals and fungi, such frameshifting is widely employed to regulate the expression of ornithine decarboxylase antizyme, a negative regulator of cellular polyamine levels^13^,^14^. Protein sequencing has also revealed that +1 PRF frameshifting is used to express the *Tsh* gene of *Listeria monocytogenes* phage PSA, the *PA-X* gene of influenza A virus and the *pol* gene of the *Saccharomyces cerevisiae* retrotransposons Ty1 and Ty3^15^,^16^,^17^,^18^.

The +1 PRF is more frequently used for *Euplotes* genes than in genes of other known species^19^,^20^,^21^. Our previous survey indicated that approximately 11% of the genes in *Euplotes octocarinatus* require one or more +1 PRF to produce their protein products^19^. In addition, +1 PRF genes have been identified in other *Euplotes* species (Table 1). In all *Euplotes* cases, DNA sequencing has revealed two separate open reading frames (ORFs) that, if joined by translational frameshifting, would produce a single protein similar to homologous ones in other species. Although nucleic acid-based evidence for frameshifting is strong, information at protein levels is limited (Table 1). For instance, Western blot analysis on the expression of *MAPK1* genes in *E. raikovi*^22^ and *E. nobilii*^23^ revealed a mass close band of MAPK1 using different antibodies, confirming the presence of +1 PRF. Mass spectrometry (MS) has also been conducted to analyze the La-motif protein associated with telomerase in *E. aediculatus*^24^. Several peptides derived from the purified La-motif protein were sequenced. One peptide was found to be encoded within the 0 frame ORF, while the remaining peptides are encoded by the +1 frame ORF. These data indicate that the protein is produced by frameshifting. However, the precise site of the frameshift in *Euplotes* remains unclear because of the lack of peptides that actually spanning the frameshift site.

In our current study, total proteins of *E. octocarinatus* were subjected to large-scale MS-based analysis through shotgun liquid chromatography tandem MS (LC-MS/MS). A total of 2,842 proteins were detected, among which 226 were translated via +1 PRF. Furthermore, seven frameshift sites in six proteins were covered by one or two unique peptides. The amino acid sequences of these peptides indicated that the frameshift occurred at “U” of the slippery sequence “AAA-UAR” in *Euplotes*. Moreover, 14 +1 PRF proteins with putative novel slippery sequences were detected. These results provided evidence for the reality of these novel slippery sequences in *Euplotes*. Our data shed light onto the molecular mechanism of +1 PRF in *E. octocarinatus*.

## Results

### Large-scale MS-based analysis of *E. octocarinatus* supports the high frequency of +1 PRF

Previous work suggested that a high frequency of +1 PRF existed in *E. octocarinatus*^19^. A shotgun analysis was applied to investigate the proteome of *E. octocarinatus* to experimentally verify such high frequency +1 PRF *in vivo*. The *Euplotes* protein sample was initially digested with trypsin, which cleaved peptides at the C-terminal side of a lysine (K) and an arginine (R) residues. However, the AAA codon, coding for a lysine, immediately preceded the stop codon of the 0 frame ORF in the majority of +1 PRF genes (approximately 94.2%) of *E. octocarinatus*^19^. Therefore, sample digestion with trypsin likely affects the recovery of the slippery sites. Two additional protein samples were digested with GluC and chymotrypsin to obtain peptide spanning the shift site. A total of 2,842 proteins were obtained from the analyses of the three samples, including 226 +1 PRF proteins, based on the criteria reported in the literature^25^. The previously reported +1 PRF gene in *E. octocarinatus*, namely, cAMP-dependent protein kinase^26^ (CUFF.28794.1), was also identified in this study. Two separate peptides of cAMP-dependent protein kinase encoded by the +1 frame ORF were obtained (see supporting information). The peptide sequences of 226 +1 PRF proteins identified by LC-MS/MS were presented in the supporting information.

The MS data provided direct or indirect protein evidence for the translation of these +1 PRF candidate genes requiring frameshifts *in vivo*. The observed high frequency of approximately 8% (226 of 2,842 proteins) of frameshifting also supported the notion that euplotids possess an extremely high number of +1 PRF genes.

### PRF of *E. octocarinatus* occurs within the motif “AAA-UAR” in the +1 direction

**Figure 1:**
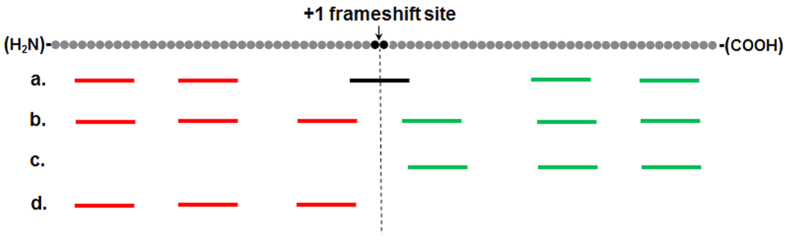
Overview of the LC-MS/MS result of +1 PRF proteins. The dots on top of the figure indicate the amino acids of the detected +1 PRF proteins. Dark black dots correspond to the putative frameshift site. The lines refer to the peptides identified by MS. Black line shows the peptide that spans the frameshift site. Red lines and green lines indicate peptides located in the upstream and downstream of the frameshift site, respectively. The 226 detected +1 PRF proteins are divided into four classes (a, b, c and d).

Based on their positions of detected peptides relative to the potential frameshift site, 226 +1 PRF proteins were divided into four classes (Fig. 1): (a) the frameshift site was covered by one or two peptides (6 proteins); (b) both the upstream (0 frame ORF) and the downstream (+1 frame ORF) of the frameshift site was covered by peptides (81 proteins); (c) only the downstream (74 proteins) or (d) the upstream (65 proteins) of the frameshift site was covered by peptides (full output of the MS/MS analysis is presented in Table S1 and Table S2.)

**Figure 2:**
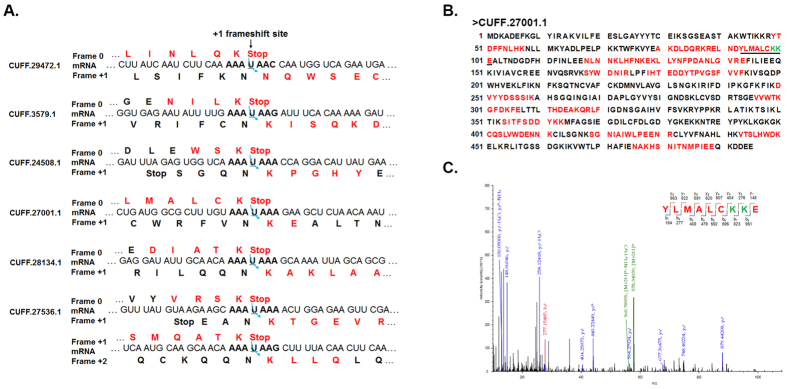
MS analysis of the six frameshift proteins. (**A**) Close-up of the frameshift region is shown. The AAA-UAA motif is shown in bold. The +1 frameshift events are illustrated by curved arrows at the “skipped” nucleotides, which are underlined. The conceptual translations in the 0 reading frame and +1 reading frame are aligned above and below the mRNA sequence, respectively. The amino acids of the peptides identified through mass spectrometry are indicated in red. (**B**) Complete amino acid sequence of the CUFF.27001.1 protein. The peptides identified by MS are indicated in red. The peptide spanning the frameshift site is underlined. The putative frameshift site is highlighted in green. (**C**) LC-MS/MS fragmentation spectrum of the shift site peptide YLMALCKKE from CUFF.27001.1. The insert shows the peptide sequence with ‘b−’ and ‘y−’ type fragment ions that strongly support the shift site peptide identified in the LC-MS/MS analysis. The protein was alkylated with iodoacetamide to protect Cys residues.

**Figure 3:**
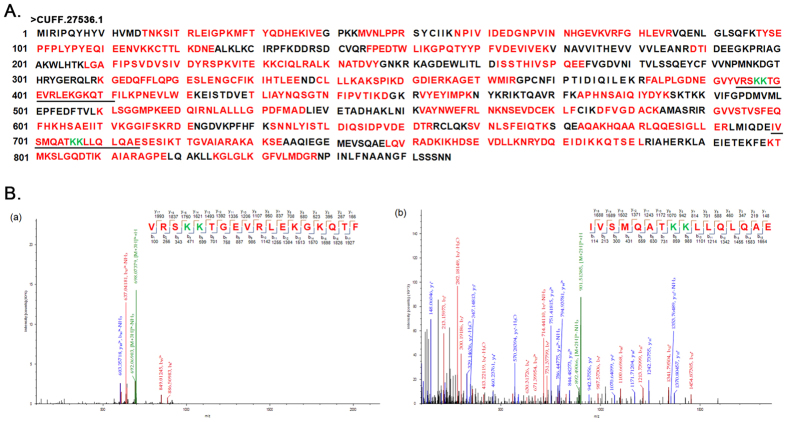
MS analysis of the CUFF.27536.1 protein. (**A**) Complete amino acid sequence of the CUFF.27536.1 protein. The peptides identified by MS are indicated in red. The peptide spanning the frameshift site is underlined. The two frameshift sites are highlighted in green. (**B**) LC-MS/MS fragmentation spectrum of the two shift site peptides “VRSKKTGEVRLEKGKQTF” and “IVSMQATKKLLQLQAE” from CUFF.27536.1. The insert shows the peptide sequence with “b−” and “y−” type fragment ions that strongly support the shift site peptides identified in the LC-MS/MS analysis.

We focused on the six proteins whose frameshift sites were covered. Except for CUFF.29472.1 whose frameshift site was covered by two peptides (Fig. S2), the frameshift sites of the other five proteins were covered by a unique peptide (Figs 2B and 3A and Figs S3–S5). The amino acid sequence of these peptides allowed the determination of the location and direction of the shift. Figure 2A shows the nucleotide sequences in the vicinity of the seven frameshift sites (CUFF.27536.1 has two frameshift sites) along with the predicted translation products from the 0 and +1 reading frames. All the seven sequences indicated that a frameshift apparently occurred at the “U” of the “AAA-UAA” when the translating ribosome was transferred from the AAA lysine codon to the next codon (AAA, AAG or AAC). The +1 shifts were also proved by the identification of the specific peptide fragments. Although the possible products of −2 shifts would feature the same C-terminal sequences, the fragments overlapping the slippery sequence would contain an additional amino acid (the first residue was introduced into the chain after shifting back), and would therefore have a mass more than 100 Da difference. However, the determined masses of the individual polypeptide fragments were very close to the calculated masses for a +1 shift. Therefore, +1 shift rather than −2 shifts is used to produce the complete protein products.

### Evidence for the presence of two +1 PRFs in one single gene of *E. octocarinatus*

Nucleic acid-based analysis suggested that several genes of *Euplotes* may use two^27^ or three^20^ +1 PRFs to produce their protein products. The CUFF.27536.1 protein was a major vault protein that may require two +1 frameshifts for expression (Fig. 3A). The shotgun LC-MS/MS analysis yielded a total fragment covering 64% of this protein. Two peptides spanning the two putative frameshift sites were identified (Fig. 3B). The peptide VRSKKTGEVRLEKGKQTF defined the frameshift site (AAA-UAA A) and direction (+1) of the first frameshifting, the peptide IVSMQATKKLLQLQAE clearly defined the frameshift site (AAA-UAAG) and the direction (+1) of the second frameshifting (Fig. 2A).

The other three proteins (CUFF.16975.1, CUFF.4515.1 and CUFF.7325.1) were also identified to harbor two frameshift sites (see supporting information). In all three cases, the peptides that were encoded by all three reading frames were detected, thereby providing a solid evidence confirming that a single protein is produced by two +1 frameshifting processes.

### Possible novel slippery sequences for +1 PRF

In addition to the 212 +1 PRF proteins with the classical “*Euplotes* frameshift motif” (5′-AAA-UAR-3′), our data also suggested the presence of novel +1 PRF slippery sequences in other 14 proteins (Table 2). Eight of these proteins showed peptides covering both upstream and downstream of the frameshift site, thereby providing a clear indication that a complete protein was produced via frameshifting.

**Figure 4:**
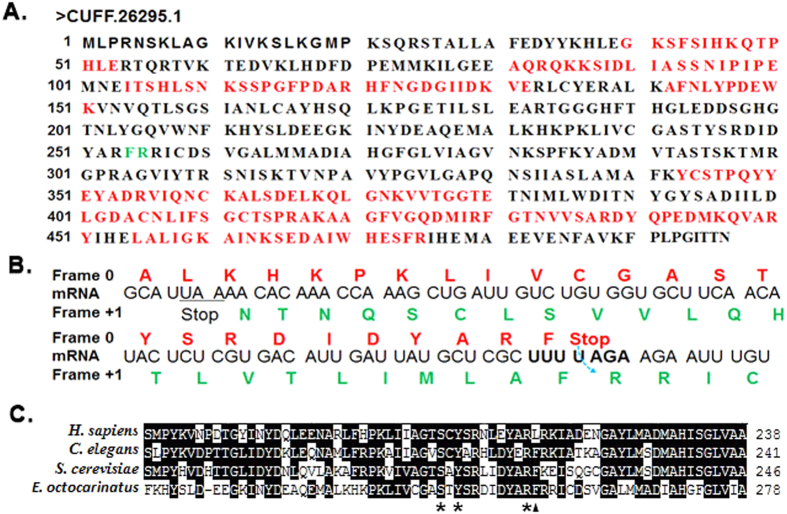
Sequence analysis of the CUFF.26295.1 protein. (**A**) Complete amino acid sequence of the CUFF.26295.1 protein. The peptides identified by MS are indicated in red. The putative frameshift site is highlighted in green. (**B**) Close-up of the putative frameshift region is shown. The UUU-UAGA motif is shown in bold. The putative +1 frameshift events are illustrated by curved dashed arrow at the “skipped” nucleotide. Conceptual translations in the 0 reading frame (red) and +1 reading frame (green) are aligned above and below the mRNA sequence, respectively. (**C**) The parts of the predicted protein sequence of the CUFF.26295.1 protein (*E. octocarinatus*) are aligned with the respective regions of SHMT proteins from humans (*H. sapiens*), *Caenorhabditis elegans* (*C. elegans*), and *Saccharomyces cerevisiae* (*S. cerevisiae*). The amino acids in black shading are identical in all four proteins. The numbers to the right of each of the sequence refer to their end amino acid positions. The sequence shown in this figure assumes the frameshift event at “U” of the “UAGA”. The conserved residues is marked by an asterisk. The putative location of the frameshift is marked by a black triangle.

Due to the inability to obtain peptides spanning the frameshift site, we could not determine their precise frameshift sites. However, the frameshift site could be deduced from further sequence analysis. The CUFF.26295.1 protein is a serine hydroxymethyltransferase (SHMT) with a putative slippery sequence “UUU-UAGA” (Fig. 4). In this protein, an overlap occurred between the two ORFs because the +1 frame ORF has a termination codon located at the 71 bases upstream of the termination codon of the 0 frame ORF (Fig. 4B). This region represented part of the conserved SHMT domain. The alignment of the CUFF.26295.1 protein with SHMT proteins from a set of evolutionarily diverse organisms revealed several highly conserved residues (Fig. 4C), among which, Ser_243_ and Tyr_245_ could participate in catalysis or stabilizing the structure^28^. Furthermore, Arg_253_ was also highly conserved. It was followed by a less conserved phenylalanine or leucine. These data supported the frameshift event that may occur at “UUU-UAGA” motif.

## Discussion

In this article, we performed a large-scale MS-based analysis of *E. octocarinatus-*derived protein samples and observed extremely high frequency of +1 PRF. In total, 226 +1 PRF proteins were detected, of which we obtained seven peptides spanning the frameshift sites. The amino acid sequences of these peptides suggested that the frameshift occurred at the slippery motif “AAA-UAR” in *Euplotes*. One of the six proteins, CUFF.27536.1, provided solid evidence indicating that a single protein was produced by two +1 frameshifting. Furthermore, putative novel slippery motifs were detected in 14 +1 PRF proteins, suggesting the presence of novel +1 PRF proteins in *Euplotes*.

In our previous study, a genome-wide investigation of *E. octocarinatus* based on its genome and transcriptome sequencing indicated that approximately 11% genes required +1 PRF to produce the corresponding protein products^19^. The observed 8% (226 of 2,842 proteins) frequency of frameshifting in this study was somewhat lower than the 11% from the previous survey. This inconsistency may be due to the fact that the majority of +1 PRF genes in *Euplotes* express low-abundant proteins^19^,^20^ that might not be detected in an MS analysis. Nevertheless, our results still supported the notion that euplotids possessed an extremely high number of genes requiring a +1 frameshift for expression. The MS data also showed the presence of two +1 PRFs in one single gene in *E. octocarinatus,* indicating that two frameshifts are required to produce a complete protein. Failure at any frameshift site because of low efficiency will end up with a truncated and/or deleterious protein. The observed high frameshift frequency suggested that euplotids may possess a unique mechanism to process frameshifts efficiently.

In our previous study, we identified 211 novel +1 PRF genes with different types of slippery sequences^19^. In the present study, the MS data provided protein evidence for eight types of novel slippery sequences (Table 2). However, we were unable to obtain peptides that actually span the frameshift site. Nevertheless, the alignment of our putative proteins sequence with homologous counterparts of a set of evolutionarily diverse organisms showed the conservation of functionally important amino acid residues. This provided an indirect evidence for the existence of these novel slippery sequences. The diverse slippery sequences complicate our understanding on the mechanism behind +1 frameshift in *Euplotes*, which warrants future study.

Stimulatory elements, such as upstream Shine-Dalgarno-like sequences or downstream pseudoknot structures, have been shown to promote efficient frameshifts in other organisms^1^,^3^. However, none of these sequences were seen to associate with the frameshift in *Euplotes*. The only common feature was the “shifty stop” (UAA or UAG) in the slippery site. In *Euplotes*, the codon UGA was reassigned as cysteine^29^ or selenocysteine^30^. Previous study has shown that the reassignment of UGA to Cys in *E. octocarinatus* results in an increased +1 PRF at both UAA and UAG codons^31^. However, the poor recognition of the terminators was necessary but not sufficient to evolve efficient frameshifting^31^. A possible factor may be the unusual tRNAs in *E. octocarinatus*. Expanded or modification-deficient anticodon stem loops could promote +1 translational frameshifting^32^,^33^,^34^. In our previous study, we reported a suppressor tRNA of UAA with an apparently nine-base anticodon loop^19^. Subsequently, we identified two genes for the AAA decoding tRNA^Lys^ with nine-base anticodon loops from the *E. octocarinatus* genome (data not shown). The structure study of the 70S ribosome bound to frameshift suppressor tRNA^SufA6^ and N1-methylguanosine at position 37 modification-deficient anticodon stem loop revealed that the disruption of the conserved U32-A38 base pair promotes +1 decoding^34^. The expansion of anticodon loops to nine-base in these unusual tRNAs might also disrupt the interaction of the 32–38 pair, thereby causing the +1 frameshifting in *E. octocarinatus*. Further experimental verification is needed to clarify whether and how these unusual tRNAs regulate +1 PRF in *E. octocarinatus.* A survey of these unusual tRNAs in other species of the genus *Euplotes* is also needed.

An additional issue that need to be addressed is the function of +1 PRF in euplotids. PRF plays a role in regulating gene expression of some other organisms^1^,^3^. However, there is no experimental evidence showing that +1 PRF has such a role in *Euplotes*. Since *E. octocarinatus* does appear to use frameshift frequently, it would be of significance to further investigate the role of this PRFs. In euplotids, frameshifting invariably results in a substantial extension of translation and accordingly euplotids may have evolved an efficient frameshifting system to process the unusually high numbers of PRFs; as such, the decrease in protein expression can be negligible. In this case, +1 PRF unlikely palys a regulatory role in these organisms. Further studies on the expression of individual genes under different conditions should be performed to clarify this issue.

## Methods

### Sample Preparation

Cells of line 69 of *E. octocarinatus* were cultured and harvested as described previously^19^. The harvested cells were snap-frozen in liquid nitrogen and then homogenized in SDT buffer (4% SDS, 1 mM DTT, 150 mM Tris-HCl, pH 8). After 15 min incubation in boiling water, the homogenate was subjected to continuous sonication treatment on ice. This crude extract was then clarified via centrifugation at 12,000 g and 4 °C for 15 min. Proteins were precipitated by adding 1/5th volume of 100% (w/v) trichloroacetic acid (TCA) and the protein pellet was collected via centrifugation at 12,000 g and 4 °C for 30 min and incubated overnight in 1 mL cold (−20 °C) acetone. The protein pellet was recollected by centrifugation followed by two additional washing steps with 1 ml of acetone. Finally, the protein pellet was resuspended in 200 μL SDT buffer and subjected to a continued sonication treatment. The sample was centrifuged at 12,000 g for 15 min. The suspension was stored at –20 °C until use. Protein concentration was measured with BCA protein assay reagent. Subsequently, SDS-PAGE electrophoresis was performed to confirm the presence of protein bands (Fig. S1).

### In-solution digestion

Protein digestion was performed according to the filter-aided sample preparation (FASP) procedure described by Wiśniewski *et al.*^35^. A protein sample (approximately 30 μg) was briefly solubilized in 30 μL of cell lysis buffer (4% SDS, 100 mM DTT, 150 mM Tris-HCl, pH 8.0) at 90 °C for 10 min. The detergent, DTT and other low-molecular-weight components were removed with 200 μL UA buffer (8 M Urea, 150 mM Tris-HCl, pH 8.0) via repeated ultrafiltration (Microcon units, 10 kD). Then 100 μl 50 mM iodoacetamide in UA buffer was added to block reduced cysteine residues. After 30 min incubation in darkness, the sample was collected by centrifugation at 14,000 g and room temperature for 20 min. The filter was washed with 100 μL UA buffer for three times and then with 100 μL 25 mM NH_4_HCO_3_ for three times. Finally, the protein suspensions were digested with three enzymes (trypsin, GluC and chymotrypsin) in 40 μL 25 mM NH_4_HCO_3_ at 37 °C overnight. The resulting peptides were collected as a filtrate.

### Shotgun LC-MS/MS analysis

The shotgun LC-MS/MS were performed on a Q Exactive mass spectrometer coupled to Easy nLC (Proxeon Biosystems, now Thermo Fisher Scientific). Six μL of each fraction was injected for nanoLC-MS/MS analysis. The peptide mixture (5 μg) was loaded onto a C18-reversed phase column (Thermo Scientific Easy Column, 10 cm long, 75 μm inner diameter, 3 μm resin) in buffer A (0.1% formic acid) and separated with a linear gradient of buffer B (80% acetonitrile and 0.1% formic acid) at a flow rate of 250 nL/min controlled by IntelliFlow technology over 140 min. The MS data were acquired with a data-dependent top 10 method by dynamically choosing the most abundant precursor ions from the survey scan (300–1800 m/z) for HCD fragmentation. The determination of the target value was based on predictive automatic gain control. The dynamic exclusion duration was 60 s. The survey scans were acquired at a resolution of 70,000 at m/z 200, and the resolution for HCD spectra was set at 17,500 at m/z 200. The normalized collision energy was 29 eV and the underfill ratio, which specified the minimum percentage of the target value likely to be reached at maximum fill time, was defined as 0.1%. The instrument was run by enabling the peptide recognition mode.

### Sequence Database Searching and Data Analysis

MS/MS spectra were searched with MASCOT^36^ engine (Matrix Science, London, UK, version 2.2) against the *E. octocarinatus* protein database including 32,353 protein entries. The following options were used to identify protein: peptide mass tolerance, 20 ppm; MS/MS tolerance, 0.1 Da; enzyme = trypsin, chymotrypsin or GluC; missed cleavage, 2; fixed modification, carbamidomethyl (C); and variable modification, Oxidation (M). The filter parameters were protein FDR ≤ 0.01 and peptide FDR ≤ 0.01. Multiple peptide identifications were generally returned by SEQUEST for each MS/MS spectrum and for each parent-ion change state.

### Multiple sequence alignment

DNAStar was used to perform multiple sequence alignment. Sequences with the following accession number were used to generate the alignment: NP_004160.3 (*H. sapiens*), CCD63201.1 (*C. elegans*), and AAA21024.1 (*S. cerevisiae*).

## Additional Information

**How to cite this article**: Wang, R. *et al.* Large-scale mass spectrometry-based analysis of *Euplotes octocarinatus* supports the high frequency of +1 programmed ribosomal frameshift. *Sci. Rep.* **6**, 33020; doi: 10.1038/srep33020 (2016).

## Supplementary Material

Supplementary Information

Supplementary Table S1

Supplementary Table S2

## Figures and Tables

**Table 1:** Summary of +1 PRF genes in ciliated protozoa of the genus *Euplotes*.

Species	Genes	Putative slippery sequences	Methods & References
*Euplotes raikovi*	*Mitogen-activated protein kinase*	AAA UAA A	Western blot^22^
*Euplotes nobilii*	*Mitogen-activated protein kinase*	AAA UAA A	Western blot^23^
*Euplotes aediculatus*	*La motif protein*	AAA UAA A	Mass spectrometric analysis^24^
*Euplotes octocarinatus*	*Nuclear protein kinase*	AAA UAA A	Nucleic-acid-based analysis^37^
	*cAMP-dependent protein kinase*	AAA UAA A	Nucleic-acid-based analysis^26^
*Euplotes minuta*	*Reverse transcriptase subunits of telomerase*	AAA UAA A	Nucleic-acid-based analysis^38^
*Euplotes vannus*	*Reverse transcriptase subunits of telomerase*	AAA UAA G	Nucleic-acid-based analysis^38^
*Euplotes crassus*	*Reverse transcriptase subunits of telomerase-1*	AAA UAA C & AAA UAA G	Nucleic-acid-based analysis^39^
	*Reverse transcriptase subunits of telomerase-2*	AAA UAA C	Nucleic-acid-based analysis^27^
	*Reverse transcriptase subunits of telomerase-3*	AAA UAA C & AAA UAA G	Nucleic-acid-based analysis^27^
	*C*_*2*_*H*_*2*_*-type zinc finger protein*	AAA UAA U	Nucleic-acid-based analysis^20^
	*MORN repeat protein*	AAA UAA G	Nucleic-acid-based analysis^20^
	*Ser/Thr protein kinase*	AAA UAA C & AAA UAA G & AAA UAA A	Nucleic-acid-based analysis^20^
	*Tec2 transposon ORF2*	AAA UAG U	Nucleic-acid-based analysis^40^

**Table 2:** +1 PRF proteins with novel slippery sequence.

Sequence ID	Putative slippery sequence	Unique Peptides
CUFF.26295.1	UUU UAG	16
CUFF.26296.1	UUU UAG	15
CUFF.8736.1	UUU UAA	14
CUFF.25615.1	UUU UAA	3
CUFF.1984.1	UUU UAA	1
CUFF.21695.1	AUU UAA	4
CUFF.2970.1	AUU UAA	1
CUFF.28108.1	AUU UAG	1
CUFF.4268.1	AAG UAA	1
CUFF.166.1	ACC UAA	11
CUFF.13288.1	AGA UAG	2
CUFF.16329.1	UAU UAG	3
CUFF.9615.1	CUU UAA	1
CUFF.2860.1	AAU UAA	1
