# Supplementary material for: Large-scale mass spectrometry-based analysis of Euplotes octocarinatus supports the high frequency of +1 programmed ribosomal frameshift
Source: Sci Rep. 2016 Sep 6;6:33020. doi: 10.1038/srep33020 (PMC5011710; doi:10.1038/srep33020)
Supplement: Supplementary Information [file srep33020-s1.pdf]

Large-scale mass spectrometry-based analysis of *Euplotes octocarinatus* supports the high frequency of +1 programmed ribosomal frameshift

Ruanlin Wang, Zhiyun Zhang, Jun Du, Yuejun Fu, Aihua Liang*

Key Laboratory of Chemical Biology and Molecular Engineering of Ministry of Education, Institute of Biotechnology, Shanxi University, Taiyuan 030006, China.

*Corresponding author: Aihua Liang

E-mail: aliang@sxu.edu.cn.

Tel.: +86-351-7018731

Fax: +86-351-7011499

Running title: Large-scale mass spectrometry analysis of *Euplotes octocarinatus*

Supporting informations

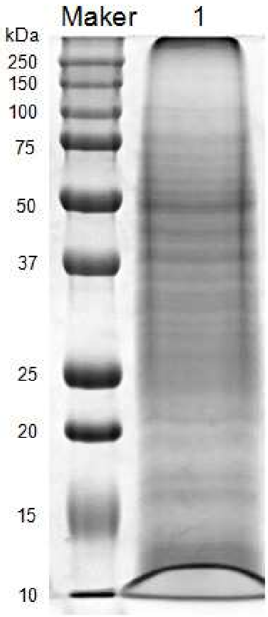

Figure S1. SDS-PAGE image of the total proteins of *E. octocarinatus*. Maker: Protein marker with low molecular weights; Lane 1: total proteins of *E. octocarinatus*.

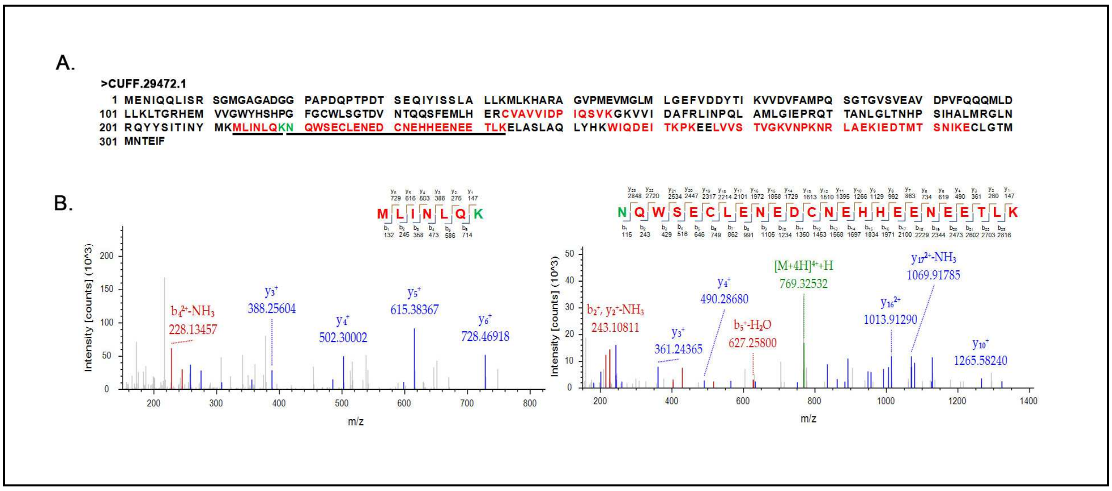

Figure S2. MS analysis of the CUFF.29472.1 protein. (A) Complete amino acid sequence of the CUFF.29472.1 protein. The peptides identified by MS are indicated in red. The two peptides spanning the frameshift site are underlined. The putative frameshift site is highlighted in green. (C) LC-MS/MS fragmentation spectrum of the shift site peptides "MLINLQK" and "NQWSECLENEDCNEHHEENEETLK" from CUFF.29472.1. The insert shows the peptide sequence with "b-" and "y-" type fragment ions that strongly support the shift site peptides identified in the LC-MS/MS analysis.

A.

>CUFF.3579.1

1 MDKEIKKRKE ELENKVMQSM NHFNTVINII SKCITQVFDQ AYEIVNVFNK TEKHYVSGKV TDEKIINKNT EIKHKCAEFY DQLHNIIYEN NRFLDIIHIY
101 TACVVSNKEH SINEYTLNLC FKVLTVLKSL TILADCKSPI VDISDCESPQ LDDSIKELSS FLTWSLSKIA HDYILCTTNY QKNSLHKIMM SKLMSGGIER
201 RYETLFSENS QKEMTLINRY LDDIAPSISE HNENDKTLES IMQPGNNNHI DTLLNLLNWN IQKKYPEEVP KTEDEIGVVR IVFTCLIKMN DLCSDFETLS
301 EQVDLHTKYN QSTSENEKFE KLNAELDHSI LDCLLRCWKF SSKILECYRS QIDSKRKLEE TKQTQNIEET KQTLEKLEDT KEENIINSGE NNDENNNKTL
401 LRANTSEINN TKERILKVFN EKANILLKMN VPNCWNLQQL DLKICRELSN RSNNGAKSRD KIDESQHLIL SFFKSDFTSK DVLMRIEEVQ IRSLRRIIGL
501 MIIGGVINTK MSQTAVVSCL NWLCSALRKG ANFIVHYTED LDGCSRYIKE KLQNEFFCII RGIIYQMKSC NEVEHARFLL NCLRWKYTSS EHSQLCNSGI
601 IEVLTHGAEI DKQHKNMFKF SWGHKIIYRN YLSNNELSHA VIETFECLYT NVLSRITSES HVKFEEKELE RIERMKRVHS SVGDESEALM SKLCNAIICE
701 LNRYSKLVKS FDPIDWKAFV KHRNKMRSNG QIINEEDPIE LDDEKTLEEL KEEQEKQEFE RLRNCENREE QSISGSQPRN RIDEILEDRA DIECRKIRKL
801 YDDKIIMKLL RMFELFTSIS LVNSKVREYA NRIISSENIA LLLEMLLSCQ FKHNLIICRI LHNLSMLEKG PETLDNACIN LCNSQSKELF DLNPVSQFKE
901 SPFLKFAFNF LLLIRSSQWR SQEFTNSGEY EVSCCLVRFF NSIMKDKHSH KWRDITYSVL DDFLSDTDQQ SYSIEEFDVM ISLIEGGEFL GLNVGSCGIT
1001 SDNRMFTIIG FCKEWFDLKP LNQAGYNGNF NIHKICYEYK NKDDSILALS YDKQYPEKRS LFLVKPDDVQ LISDFDDKTS TYLLNQTRLQ NFISKMRINE
1101 LPDKTDSTSL TKRCVGMKIL VNLINKNGDE INSLIEPQFK NNLISMMLRE CSTPEEGAES FRCEWYDQKI YLIKKLATER QLSLRKEVYK SITFKGNMMS
1201 ISKPLEISTK ELYCQVFYLT SAMNYGRIEQ GIKYQVLNQE HFKSISCRFD SVVILDSSQC DSIDKLEKIF KNVDTLITSD IDLNQIHDQL KTTNKNIAFF
1301 KSIVLVDRKD ICSLIELLEN DPKVKVASTD EMNLNETFIS EITVFGGFNE KEVKKVLRGS ESTNLSQKVA LITKWMKDSK RKEENKEEMK EEDQKQTEDT
1401 KKIDSDSEED DPEENDPLTI YGLESHEKLE SKVNELAAFD NNNFNMAVDV QHASKDDLYT KVYSIKINDI HLEYTKTLAT YYKQLCRSTI STFFNELAIE
1501 DILDIICIEK EVTKKFINYI LMKGNEAAII KIKSKDSKPI DDFMIMFNKL ITVCSHKSKF INIIDIIYSK AIIKNTKNLL IDSIKAGKDN IEGIYSNEIV
1601 SAKTMNVLLI PQTLSQILKI APDIISTNVK NFPNLISILL LIAICFRKEF ELQRSVYMLI YKILVPIAKS PKSYNPQLIE GLLRLKIFKK MIEKCNFDVT
1701 VLSGNKLTPE QKLLFEINLL IHKIDSNMVD KWSLYTYTDI LLELGKCKEV LKEGKEFDMI SYLIYFNNEL SGPQQHTKVY ADTYYNMFET KISAKVHFDG
1801 FKKMTLRVDE TSKIRDDTFI GISSDPDGEN ILKKISQKDI EKESTRIDIY FNQCFIHYPY NPPRILGFGE GYDYKLGNSN TAITSEPTEF SNFRKSIKSI
1901 KTRRNFTVAL DENGQLYYSG YKTSLSNTNQ TMTKYHKKLP SDKIIRIEVG SYNIIALTDS GKFYCEGSDY LYQFDSYSTE KYDFWYKQRP DEEKELVVDF
2001 DAGNYYHLYV TDNGKLYGAG NTFLSGLNLN SDSKDYKLIP LPENVKPIRV RCSESSETPK SALIFVEVDG KRQIWSGGNS STGMLGQGEE ITESKTFKVL
2101 NYDTDNIEFT EVKLGTNHAL GITSNAELYG WGSNSSYQCG LPEAKNYFIP TEIPFFKDYF THLIALGEDF SIVKVSPRTD RENTMLFSIG NVHGINAEGK
2201 TAEGLLHLSQ FDNQNIKIIE AGEKQVVLVY DGELKASDNV GIHHEYKCEV TGQNPIIGTL HFYKDNSGDW HFLSREGYNQ NKHILPPICF VTKYYIENIQ
2301 GKEWPEVNVN EILENSHDEF APSYLSNVSV KGELIRPMPE TDEIGIFEKE TNDMIPLIFY RITRPIQDCK ALPTIELEKF HKHCESYRIR LEADPDYSFL
2401 KNDGIIGIEY DRYMSIKNRI QNFDEKYDKE ILRVIQNYLK VNSQDFIECP WVFLMAAELE FNEPGIYSFP TDDIQLRIDS LVQFNQYFLK SIPYVLLDDE
2501 LTHEETIGET ILQSESLSAS FILGK

B.

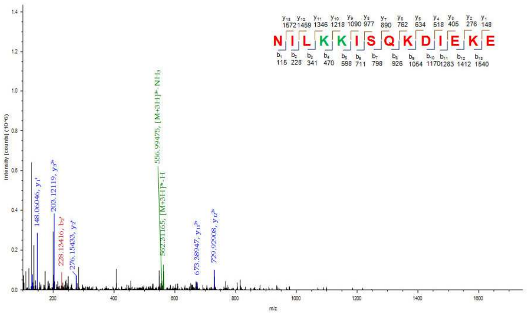

Figure S3. MS analysis of the CUFF.3579.1 protein. (A) Complete amino acid sequence of the CUFF.3579.1 protein. The peptides identified by MS are indicated in red. The peptide spanning the frameshift site is underlined. The putative frameshift site is highlighted in green. (C) LC-MS/MS fragmentation spectrum of the shift site peptide "NIL<u>KK</u>ISQKDIEKE" from CUFF.3579.1. The insert shows the peptide sequence with "b-" and "y-" type fragment ions that strongly support the shift site peptides identified in the LC-MS/MS analysis.

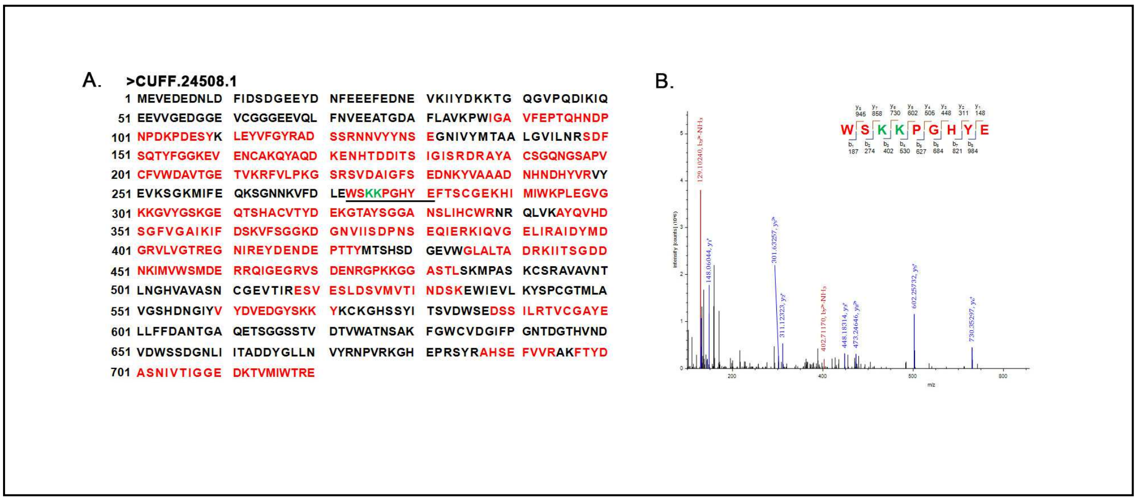

Figure S4. MS analysis of the CUFF.24508.1 protein. (A) Complete amino acid sequence of the CUFF.24508.1 protein. The peptides identified by MS are indicated in red. The peptide spanning the frameshift site is underlined. The putative frameshift site is highlighted in green. (C) LC-MS/MS fragmentation spectrum of the shift site peptide "WSKKPGHYE" from CUFF.24508.1. The insert shows the peptide sequence with "b-" and "y-" type fragment ions that strongly support the shift site peptides identified in the LC-MS/MS analysis.

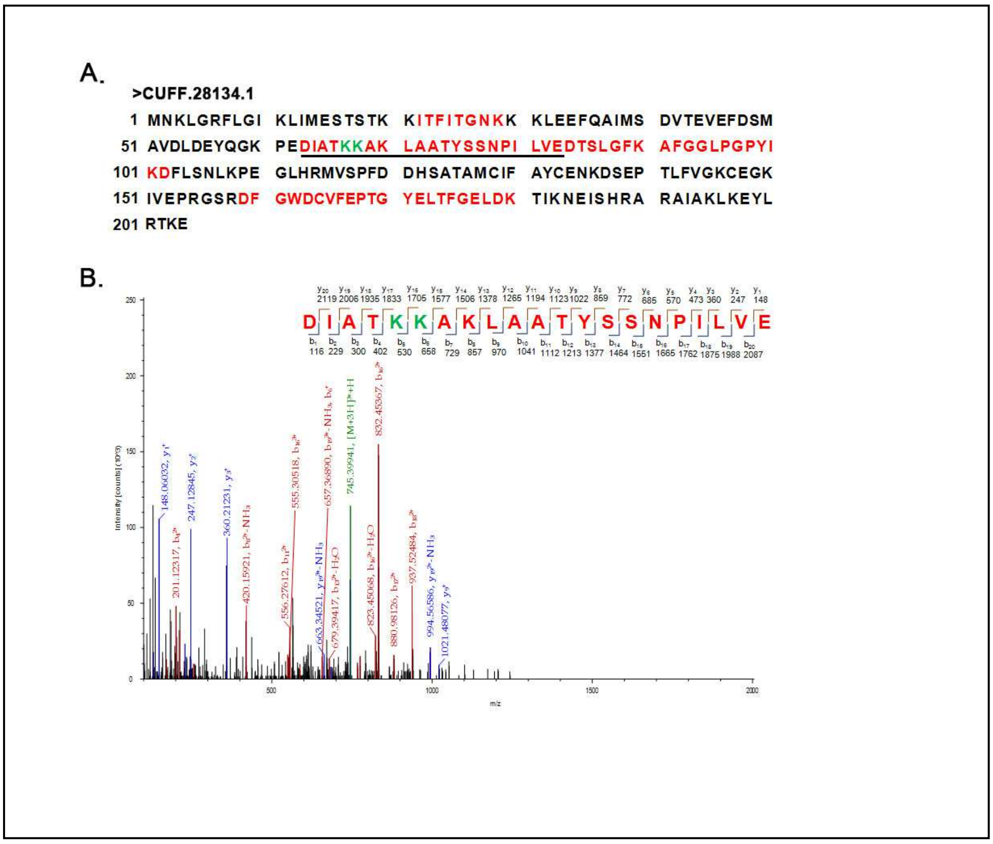

Figure S5. MS analysis of the CUFF.28134.1 protein. (A) Complete amino acid sequence of the CUFF.28134.1 protein. The peptides identified by MS are indicated in red. The peptide spanning the frameshift site is underlined. The putative frameshift site is highlighted in green. (C) LC-MS/MS fragmentation spectrum of the shift site peptide "DIATKKAKLAATYSSNPILVE" from CUFF.28134.1. The insert shows the peptide sequence with "b-" and "y-" type fragment ions that strongly support the shift site peptides identified in the LC-MS/MS analysis.

The peptide sequences of the 226 +1 PRF proteins identified by LC-MS/MS. In the protein sequences, the putative frameshift sites were highlighted in green. The peptides detected by LC-MS/MS were in red. The peptides spanning the frameshift sites were underlined.

>CUFF.18690.1 HECT E3 ubiquitin ligase, putative
MGNKSITERIESASKVNLVSKFNIEDLIDNTLLDSIYDEFKNQSALSFEIDEAYKADFTEFNEQKNEFLSDLI
KDRVIKEINEAITTGFSTPDFFIEELKEPRFEKYFQVKEKGDAGEYFKKQYKKFFILYLYYKIKTQDLEVTK
KDFYESLDSYFGKTSTSEKFFEFETGLRMTFSIMKDLGKTNPKLLYTSLKSLYQSFVTTECENRRKVDVFD
YYAEEELFNEIRTYLIGLLTDRNSTDSIQEICIKLILLIGNIRGSGEDFLIAFNLINKHGYEFNIDAELSQCKFID
SSSHDLKEGEADDRLKVSYEGNKSAHILKGGDIDFDFNVEINMTFDSQFIYAYQHGKGLLKLGASDSAAT
KLGRLISKNSSFSDDHRSFMYLNGKLYCRFKNSDGKPFTLINTETLEEITNNEEFNKKIEALKEKGPEEEKK
EAEPEEDEKTEEDKKPEETPTLGWTKEDSEEELKGGRYLCASPLFTDGVDIYVISTIREVKIIRKEDENLDE
DRKELAITKWNLEVYDGSTWKFKKSVEIQLDIKHRGIDEHMTEAEEKEIDAIKQLRESFNANHLAYCSFA
TNGTKLLVGENNRWHSYDINKLKWNFNLELTEGNWAYNYMANTFWQITSSPEVKSFKIPAFKRLETGIS
HSKGIIEYINNKVKDIQKLQTVADKLTKRTPKNLFKNLMKKADEQTKNPIFETKTKADYSQFLILNILKEG
SKELITIAENINPNGPVDNKKLELFRGSFSTAVTGTFFSELLNAVEGSVGLLSKKQKKSSNILEQYNFYMILI
LLRTSMMCLSKLNIPISTVLYERNDFDRLVKIVEQNISVMVSKEAIDYTIEDGNAHEELSCIWSSIESECRSI
MSYCLSLVYESLEDLMRKVTSLLERMDIGQSDNSLGIYINYLTLPETMNKALKSNEIKDLILVVYEKLCML
KTKKLISAIDSVKYETHQLHDVEYSSLEISNEGFIYAVTQNIINLYFEAYRDVEKQKEQSKSDKDKLEMLE
RKLERTEFDLIKVLERISESTIQILDKCSSSTQYYLDEALKQQKEDDKIEDQEKKPKVTGKMKYVEFHRYL
YKIAHLNNNFPNLMHVYVAAFASLKDSTNVKTLSLLSIKILKVIENFSKFKEIVSEDLPLSESEDLLRNNLD
KDIIKFFVWTISKLSYSFINVKKVAELSEEEKMNKKILDSKLLSGGIENRFLPILSKDTVSSLRDLCEITADRT
IKEYLVNDPKELDEDQVFMAIIHQGKDKNIDRVIDLLQFNMERKHPWAKSAKEEGMRLSRAAFACMIKIG
ELYTDFIMMVDETEMNADSVDADDEATAKKQLAQELSKIDSYETIFKHWESSAKMRSWYQEKKKSWAN
KIKKEEEEKKKKDEADSKTTEENKDKENKDKDKENKEKDKVKPKDEKEDKKDDGEEVIDTTTKPTDDN
QPKKKTKDQIELAKIIEKVVEKAELLIKLNMPTSWGSKEQTKFNMIETLAEEEEKKIHSSEVKEEIPDWKTR
LDKWREIQESAGSIRNFEEQKAAIFSSSTTSILACLQTPISAQRIKKQIETIYLNALKRICGFRLIARLSYMKH
STEIRTEYLNWFCSSLRHNTNILSHYSNDVTGCGEHLRNELRKGFFLVFNGIVKQIGECTDESEIKFLLNCC
KWQFSASDHDDLIKSGIFEILSQGNGKKEKDKNPIKYCWGHNFKYDSKEDYPLCQDVLDLFEQVLMACF
ARIIKKDGGEEQKLKTSGTSIPGMEKAHSLVNTTTTEGLIAKGFKHVFDQLERYIKLTKKFEGIDWEFYVS
KRNNQRKKGDKIEEIETNELEDENEPVPEETKEPEATESVADSESVVEEAKKEETEVKPAEVDNEETKDSA
IPDDKVEETKEIKPEEDTKENDDPNEDAEDAQDDNEGGNEEEVKDKVREKVEEKENKEIEKLKKLYEEK
YITRFLKLIEIFTSIAADQKGVLGMVLKIASPQELTILVDLLVYAAPRHGFTILKIFDNLFRIKVPHELFDESII
RLSKIDGSLHQKIMNINLIKIDDTKQQNKFENTPFLRFLFNYIVSIRAAMWNDSHFEGSGSFILSSMMLGILR
TILNNDIYPKYQKEIINAIDHLIDNIDKYNLEEFEPLISVLNGAEYLGLGLGTNGVSSDGSMFTVLGFVQKW
YGLKTQDDPNNNQNDDEFVKHRIQSHLEDKNDHILALYYDPKHPERTDIFTAIPEEVRLIPRLEKETNKILL
DKTRINKFLRALKLDEKLDKGDYIQSTKRSIGLKILEQYLVDHGEELSKLIDTEFYNSFLKLLYSEACSPSS
GKSTMQNDWIEQKIHALQEYAAENNSSLRTSSDTKARFADKKMVLSKVLKSAGNQRYSKCIDLVSAMN
YGRVIQAENYRIAESVKLPEKDEDLSKIKEKDLRLIDPSEISTVDLMCKHLKTAKILLSCDVDIESMHKGLV
EKDSEIAYFKSIVIVSKKDFMEIKQFLAEDPKIKVVNTEGLSSYEAFIKEMVEYGGFERKTLEEILKGNEEA
PLSRKSNLVSDWCKQQEEKKDKEKKDKAEESKNSKEEVKEEKKEEKKEKDAKEMKDKKEEIKEEIIDTK
EVKVKEGEKHDKDKKEESKEDKEAFDEETIFNLEKNDKFTKIFETVSKYGSTNYDDSANKPKKQRGTLFN

NVYNVNSDNVEGEYLNCIGTYFVSMCRKTILSFFKISQVDNLLKLLLSSKHDTENFIKFVKIIGNELVLTKL
NMIDDKPYEDLKGILGNIIKSCISNKEFTPFLHQLFKDVIVNGSIKGLKTALEAGAKDVKSYFNNETVAIET
MNFYILVDLVNLYIKECPDLIFENHDLFKKFMTMLFLIPCVFRNDGKLQKNVYLSILKIVVLVDQSIGKKNI
FNNLFIEKGTYSKKIREHLLSLKIFKKLIECINFEKDSANSYNFKLTDEHKLLFEIFLKFSSIHKASAKDGIEPI
YTYTESLLQMERCKIILKERKQFDMISYLMYLNELDDASKQSTILVDSQHPHFVPKVTACLKNEGFKKMT
LKVQSGSELEKYSSVAITSDRKGENILKNIYQKDITDNKTEVTIFSDACYIHYPFRQPKIIAFGDKDQNKLG
TDASSDPTKEPEVVPDLFEDLKCLANHQSFTAALDSKGVLYEAGFKTTFSQTYTKFTKHVVNGFKENEDK
LVKMVAGFENIILLTEKGKIFIEGSNEQYQIDDSGDKYQFTEKKIPNDDDPVIDVSAGRCHHLIVTKSGKLY
GAGNYFLKDIKIECGKKYAKIDLPNNAKALRVFCTNVEKPCVAFVLVEVKKGKTELWSAGESSKGLLGQ
GENTKKSGTFARLDYEHEKLNFVEVFAKYDHAMALTDDGQLYGWGCNIQHRLGLKKEGDKFKPTHIAF
FKDYIVENVSVGMSHSLVIASPKTDKSKKMVFSIGKEEGVFSHYGITEEDSKNMEEIITHLKSFDHLEPYLI
EAGNKTSFVAFRGDKLPSSGVSVHQEAKCEITGESPIQGIMYFYKDADNKMHCFSEKGYAKAKDTLPGIM
FATKYPLKNLKCKFPKLKEGDLLISEDSDNDAKYPLYITNLKLGDKPLTKVELTETEFHNSNVNDIDPLVY
YRISRPLAEGKELPVMNLSDYFDQTKQKGLSIELSPDYSYLKNDKIIDKAKETYKEIYDQVVKFPKQCDRE
LLECLEKHIKDNDLNFDETNSSKIEISPSSLTFKNKKLKGLPDKTKQQRINALLMYNQYFLKTIPYVLLDED
TLSQMATSRDAMSDKLNELFIVGKALAFSSIKNKYIKNIVNNLPCNWDQPECKIKRRKAQRFQDSGKVDH
KGEYTIFGQIWSTLKKTNYNSLKKNDANTKAWSASLVGEGSIDGGGPYRETITNLCGELMHNVLPLLIPTP
NNKNDHGLNRDCWTINPSAKSPTHLEMYEFLGALMGMAFRSGQILDLKLTSFFYKSLAGEPLTIEDLKAI
DLYAVQAIKEIENTKKNVTEEMFDSYGAQTMTTRLSNQEEVDLIPKGKEITLKYKDIDEFVRLTLDARFKE
AEKQMQAIRKGFEIVFPTTVMGILTWREVEYRIIGPTEIDMEELKKITNYSCCSETDEYVQRFWRAFEGFT
QEERSMYLKFVWGRSRLPPPDSSGIQRHVIYLMYAGQYADHNQVLPQAHTCFFQLDLPRYTTDAACRDK
VLYAIETCGEIDTDGYGRYADEDE

>CUFF.21110.1 putative MORN repeat protein

MGICSSCCANEENKKEINMVIDTGKAKDGTYLNKVVMIQAIMRTYLDMKKVKQLKESKKVQGNIAKFEP
EGRPLREEGQSKQESKPTFSDDIYKNPVVEEISKNLGEFEYGAVANDGIKREKRPAVYFENGSKYTGEWN
TTNNQRDGKGVQFWADGSKYEGFWKNDKANGRGRLIHSDGDVYEGEWKDDKSHGYGVYLHSDGAQY
QGDWVEDKQEGFGVEEWPDKARYQGTYKDGMKEGKGVFYWSDGSKYTGEFHNNNIHGKGVYEWVD
GRKFDGDWENNKMHGEGIFEWSDGRRYQGHYVNDKKEGYGVFTWPDGRKYKGNWANGKQHGEGTFI
NDKGVEQTGIWENGKKKEWTRAK

>CUFF.25804.1 Putative MORN repeat protein

MQASGSLPVNQGADLQNKAAQEINPEINEELYYNPTVEEIANHLGDFEYGPPLNDGVRREKRPYVYFENG
SRYLGEWNIESNKRDGRGIQYWADGSKYEGYWKTDKANGRGRLIHSDGDVYEGEWKDDKSHGYGVYL
HSDGAQYQGEWIDDKQEGYGVEEWPDGARYEGTYKDGMKEGKGVFHWSDGSKYTGDFHNNNIQGKG
VYEWSDGRKYDGEWENNKMHGEGRFEWNDGRSYEGHYINDKKEGYGVFTWPDGRQYRGFWLNGKQ
HGKGVFVNDKGIEQEGMWENGKKKEWIKGNKNK

>CUFF.26319.1 Putative MORN repeat protein

MLSSKSKVIVDNAKRSNNFHLILRVQTIMKSYLASKRAAKIKAERGMVGSGAPRVDNNYQNEVVEQIFSQ
LGPYDYAMAKNVVYDENIIMKGWRTLENGAKYNGEWDANRGVKHGKGTQIWPDGSRYDGYWRNDR
ANGPGRLIHADGDVYQGDWVDDKANGWGQYMHLDGSTYEGYWQNDKQHGHGKEKWPDKAEYEGE
YEGGKKHGHGRFFWADGSVYEGQFFDNNIQGEGIYQWADGRRFQGSWINNKMHGLGSFEWADGRRYE

GEYIDDKKEGTGIFFWPDGRVYEGAWKNGKQHGYGRYCNQSGEEKFGFWRNGVRERWLTPDEFNQAR
NQGHFDFLNHQ

>CUFF.29096.1 MORN repeat-containing protein 1
MKTSSKKDNTSNIQDNGERGKGRFQYPNTFFNYEGQWENGMMQGYGVLKFADGGYFEGEFKDNQIIGY
GTKIWSDGSEYKGYWNMGEKDGEGTLKKPNGEIYTGDWSMNVRHGNGKWTKPNKEVIEGEFINNQPQ
GYCSYRLSNGDLYQGNLDKGIINGKGMYKSLTKKMCYEGDFINGRREGTGKYYAISGAYYYEGEFKDD
APTIMPNQALFFKEQKEEEPVDPKQKKDPKKGAEEEDENPNKLIFEIGKSEPLKFEIRAVYQGPPYQDDTP
LDEEELKKQAAQKKGKNADEPEIRMITPPPVIITQESGRVFEFVLGRMEVPPKEGDEASEAPPSQVPASQA
KTEPEPEKIFVNYPFDQASGQAASRYVTNEGVVLIEGLQYAINEKFTAGKYDIFVRDVTQSIDTILPEVKIQ
LEIINPEEPQIPVKGKKK

>CUFF.24508.1 Ciliary WD repeat-containing protein
MEVEDEDNLDFIDSDGEEYDNFEEEFEDNEVKIIYDKKTGQGVPQDIKIQEEVVGEDGGEVCGGGEEVQL
FNVEEATGDAFLAVKPWIGAVFEPTQHNDPNPDKPDESYKLEYVFGYRADSSRNNVYYNSEGNIVYMTA
ALGVILNRSDFSQTYFGGKEVENCAKQYAQDKENHTDDITSIGISRDRAYACSGQNGSAPVCFVWDAVT
GETVKRFVLPKGSRSVDAIGFSEDNKYVAAADNHNDHYVRVYEVKSGKMIFEQKSGNNKVFDLEWSKK
PGHYEFTSCGEKHIMIWKPLEGVGKKGVYGSKGEQTSHACVTYDEKGTAYSGGANSLIHCWRNRQLVK
AYQVHDSGFVGAIKIFDSKVFSGGKDGNVIISDPNSEQIERKIQVGELIRAIDYMDGRVLVGTREGNIREYD
ENDEPTTYMTSHSDGEVWGLALTADRKIITSGDDNKIMVWSMDERRQIGEGRVSDENRGPKKGGASTLS
KMPASKCSRAVAVNTLNGHVAVASNCGEVTIRESVESLDSVMVTINDSKEWIEVLKYSPCGTMLAVGSH
DNGIYVYDVEDGYSKKYKCKGHSSYITSVDWSEDSSILRTVCGAYELLFFDANTGAQETSGGSSTVDTV
WATNSAKFGWCVDGIFPGNTDGTHVNDVDWSSDGNLIITADDYGLLNVYRNPVRKGHEPRSYRAHSEF
VVRAKFTYDASNIVTIGGEDKTVMIWTRE

>CUFF.846.1 UNKNOWN
MSDFSENVRKAYEQXLLGNKQEAFKLLIPGTKHHYXLSIMDALKRXRHAISADTLXMNKQFRNAFYDED
STRTKIQELFLRFDGAKTDEERDQIIKELDTSFIYGFYNHTKPADIKSKKTVKDKSADKETHLFDQNKYFN
EEKFMKKLYSQNGLIYSLHRTLYEKIDFRKISENEFLGFCNSSECFASMTNETFWEKLITIFDQKYKTNKHF
APDQYLYDKFTLEQMEMLGEKIPNIKSDIHYIGKMFEKKFHFELDDENKDTFTLEQRREQLITIYEASTDK
PQSFKSALLLEILENGVKMDIYDKNYFIEYLKNPLKNWHMNKETQKKKEIHDHVWNQYMQSLNNRSGG
RMDSGLDQKLYKKYLEQFYIAAGDLEAFKDYFDQDFLSNLYEEFEFMTGKEIKKEKIDAKKFENLSNLVL
INLLECNKSVFKKEDRVKLVVEIKNVSTLHVKIFEFNSENYYRKNLAPFRTDVNLDGLVTSHEDTFEFKEIP
QKKFRHVFEFPQLDHIVGLFVIEFISNGYSSRAVIKKGSLSLIYKSTVAGQVAYILDENKEICKSEKTGMWY
KNQYFKADPEKGGRIVIPYEKNSSNDKTILINDGFAQLTEFRRMSESYSFDVAYIVQPESLLMGKEAEILLK
PVVKVNDRRCNLSILKNTKITLTTTSFIDNLPVTKTFENLELDNKHEIPIRFQVPPNLQTVSIFIEAEVKNISK
GTNDKLSHSHIISMDTKCNSLCFYESYLRKLKNDYYYYIFGKNGEPLDDVTVNFTLTHKFFSNQPENVSLT
TDEEGKINLGSLEDISYLNTNFNGPNGACTSSHNISNIKERVSMPDSLNILEDEEIQLPYLSNTPFSQSSVWL
VSFSSSYKVIKNCFDKISFRHAKGYEYGRITISCLERGSYNLYLNATGESIPIKVHKGVYWESDSFILKNHSL
VEKREKTNIIRIKDILLEEEKDQGHTLSFNIEDYGKNARAHVFAHTFLQEDMNTLFTQVENVGRDWASLDI
FPFAKWENIYLSNRKLGDEFRYVFDRKFVKRFMGNTLDRPQLLLNRYKLRETQFDQEVVGAGNQYEEVK
ESAVNYDMRMQSLAGPSTRLARNMAPMMEQQRFMPQSVMMQNIAPMAYSNSNINANYSNSYGGNYGC
PTGSFKSFQNFLNNSSCVISNLTPDEKGHITCSFDASKYSTVLILVIDENTVSQLYVDVHTSLEMLEKRDLS
LSNPLNPTKYYNEMRNSEILRKGDDYGIEDITSTNYIMIDSIERVNQVQKEIRKTIGAYATEDLSFLIKWNT

FTHDEKNKKYSQYVCHEVNLFLFFKDPEYFKTVVRPFISNKMEKSFIDLWLVGDHDAVVKYKNIELLDRL
NTLEKCLLISEVVKDSKEEAKAIADRIKLLSDQKELKIDVVNRIIDSVINLNMTQNQPTLELLRDEMKLEEK
CSKEYICLDEARSSNRLMGYAPRGMAMTDKVQLRSAGKKREKEVFKESYNDEDDISFDDYEDLRKSTRT
QFQEAEAATEYCETHYYKNPNVFNPNTVQENPFWVDFANHIIETGSLNNFVSSNFIFATNGDTQAWSVLG
LMNLPYESPRQIMKSLGGKGIKITATDNLVLY**KK**EIREAEAELDTNLLVIHRFFEANNRNSEKKIKEFLVN
QVYGCETIITNVSTKSQDFQILWQIPEGSLPLQNTNYQKSENKKLGSYSTTTFQYYFYFPHPGNFIQFPSNIT
IGEKVVAVANECKFEVVKERQEISNETFRDILLSGNNKAIIEFLATVNLYKGDKGFNFSDILWMLKDKEFFI
EVTDTLRNRRIYDDLVWKYGFYHRDPNTIQESIMRNNAILTQAGVFFESSLIRCSPETSNFRHLDYFPMINA
RAHKLGGDANPGILNRQFRETYNRFLKVMIEKIKLDENDHLNFTYYLLLQDRINEAIDVFEKINPKHFDEK
EALRMQYDYMRAYLDFFTGSETGFKVAKSISKKYADYPILAWKVLFTEIFDQLEEFEEGVDYDEDINQED
ETKRKANLKKSINLEPTLHCELDGKNIKVEYNNIPKVVIKYYVIDPEVMFSRAPFLNQNTEDFAYVKPMEI
QEVELDKKLKSESFTIIEKLQKNNLVIEISGEGKQSFLTYFSTSLKIVINENFGELKATDQNDKPLARVYVK
AFSKETNGTIKFFKDGYTDIRGKFEYAQINSKKLNSVEKFAILVMSDEHGSITREAKPPTNISADLGEEVAF
LQPSHANKWAHQQKRSMNKKLK

>CUFF.27536.1 major vault protein
MIRIPQYHYVHVMDTNKSITRLEIGPKMFTYQDHEKIVEGPKKMVNLPPRSYCIIKNPIVIDEDGNPVINNH
GEVKVRFGHLEVRVQENLGLSQFKTYSEPFPLYPYEQIEENVKKCTTLKDNEALKLKCIRPFKDDRSDCV
QRFPEDTWLIKGPQTYYPFVDEVIVEKVNAVVITHEVVVVLEANRDTIDEEGKPRIAGAKWLHTKLGAFIP
SVDVSIVDYRSPKVITEKKCIQLRALKNATDVYGNKRKAGDEWLITLDISSTHIVSPQEEFVGDVNITVLSS
QEYCFVVNPMNKDGTHRYGERQLRKGEDQFFLQPGESLENGCFIKIHTLEENDCLLLKAKSPIKDGDIERK
AGETWMIRGPCNFIPTIDIQILEKRFALPLGDNEGVYVRS**KK**TGEVRLEKGKQTFILKPNEVLWEKEISTDV
ETLIAYNQSGTNFIPVTIKDGKRVYEYIMPKNYKRIKTQAVRFKAPHNSAIQIYDYKSKTKKVIFGPDMVM
LEPFEDFTVLKLSGGMPKEEDQIRNLALLLGPDFMADLIEVETADHAKLNIKVAYNWEFRLNKNSEVDCE
KLFCIKDFVGDACKAMASRIRGVVSTVSFEQFHKHSAEIITVKGGIFSKRDENGDVKPFHFKSNNLYISTLD
IQSIDPVDEDTRRCLQKSVNLSFEIQTKSQEAQAKHQAARLQQESIGLLERLMIQDEIVSMQAT**KK**LLQLQ
AESESIKTTGVAIARAKAKSEAAQIEGEMEVSQAELQVRADKIKHDSEVDLLKNRYDQEIDIKKQTSELRI
AHERKLAEIETEKFEKTMKSLGQDTIKAIARAGPELQAKLLKGLGLKGFVLMDGRNPINLFNAANGFLSSS
NN

>CUFF.3579.1 hect e3 ubiquitin
MDKEIKKRKEELENKVMQSMNHFNTVINIISKCITQVFDQAYEIVNVFNKTEKHYVSGKVTDEKIINKNTE
IKHKCAEFYDQLHNIIYENNRFLDIIHIYTACVVSNKEHSINEYTLNLCFKVLTVLKSLTILADCKSPIVDISD
CESPQLDDSIKELSSFLTWSLSKIAHDYILCTTNYQKNSLHKIMMSKLMSGGIERRYETLFSENSQKEMTLI
NRYLDDIAPSISEHNENDKTLESIMQPGNNNHIDTLLNLLNWNIQKKYPEEVPKTEDEIGVVRIVFTCLIKM
NDLCSDFETLSEQVDLHTKYNQSTSENEKFEKLNAELDHSILDCLLRCWKFSSKILECYRSQIDSKRKLEET
KQTQNIEETKQTLEKLEDTKEENIINSGENNDENNNKTLLRANTSEINNTKERILKVFNEKANILLKMNVP
NCWNLQQLDLKICRELSNRSNNGAKSRDKIDESQHLILSFFKSDFTSKDVLMRIEEVQIRSLRRIIGLMIIGG
VINTKMSQTAVVSCLNWLCSALRKGANFIVHYTEDLDGCSRYIKEKLQNEFFCIIRGIIYQMKSCNEVEHA
RFLLNCLRWKYTSSEHSQLCNSGIIEVLTHGAEIDKQHKNMFKFSWGHKIIYRNYLSNNELSHAVIETFEC
LYTNVLSRITSESHVKFEEKELERIERMKRVHSSVGDESEALMSKLCNAIICELNRYSKLVKSFDPIDWKAF
VKHRNKMRSNGQIINEEDPIELDDEKTLEELKEEQEKQEFERLRNCENREEQSISGSQPRNRIDEILEDRADI
ECRKIRKLYDDKIIMKLLRMFELFTSISLVNSKVREYANRIISSENIALLLEMLLSCQFKHNLIICRILHNLSM
LEKGPETLDNACINLCNSQSKELFDLNPVSQFKESPFLKFAFNFLLLIRSSQWRSQEFTNSGEYEVSCCLVR

FFNSIMKDKHSHKWRDITYSVLDDFLSDTDQQSYSIEEFDVMISLIEGGEFLGLNVGSCGITSDNRMFTIIGF
CKEWFDLKPLNQAGYNGNFNIHKICYEYKNKDDSILALSYDKQYPEKRSLFLVKPDDVQLISDFDDKTST
YLLNQTRLQNFISKMRINELPDKTDSTSLTKRCVGMKILVNLINKNGDEINSLIEPQFKNNLISMMLRECST
PEEGAESFRCEWYDQKIYLIKKLATERQLSLRKEVYKSITFKGNMMSISKPLEISTKELYCQVFYLTSAMN
YGRIEQGIKYQVLNQEHFKSISCRFDSVVILDSSQCDSIDKLEKIFKNVDTLITSDIDLNQIHDQLKTTNKNI
AFFKSIVLVDRKDICSLIELLENDPKVKVASTDEMNLNETFISEITVFGGFNEKEVKKVLRGSESTNLSQKV
ALITKWMKDSKRKEENKEEMKEEDQKQTEDTKKIDSDSEEDDPEENDPLTIYGLESHEKLESKVNELAAF
DNNNFNMAVDVQHASKDDLYTKVYSIKINDIHLEYTKTLATYYKQLCRSTISTFFNELAIEDILDIICIEKE
VTKKFINYILMKGNEAAIIKIKSKDSKPIDDFMIMFNKLITVCSHKSKFINIIDIIYSKAIIKNTKNLLIDSIKAG
KDNIEGIYSNEIVSAKTMNVLLIPQTLSQILKIAPDIISTNVKNFPNLISILLLIAICFRKEFELQRSVYMLIYKI
LVPIAKSPKSYNPQLIEGLLRLKIFKKMIEKCNFDVTVLSGNKLTPEQKLLFEINLLIHKIDSNMVDKWSLY
TYTDILLELGKCKEVLKEGKEFDMISYLIYFNNELSGPQQHTKVYADTYYNMFETKISAKVHFDGFKKMT
LRVDETSKIRDDTFIGISSDPDGENIL**KK**ISQKDIEKESTRIDIYFNQCFIHYPYNPPRILGFGEGYDYKLGNS
NTAITSEPTEFSNFRKSIKSIKTRRNFTVALDENGQLYYSGYKTSLSNTNQTMTKYHKKLPSDKIIRIEVGSY
NIIALTDSGKFYCEGSDYLYQFDSYSTEKYDFWYKQRPDEEKELVVDFDAGNYYHLYVTDNGKLYGAG
NTFLSGLNLNSDSKDYKLIPLPENVKPIRVRCSESSETPKSALIFVEVDGKRQIWSGGNSSTGMLGQGEEIT
ESKTFKVLNYDTDNIEFTEVKLGTNHALGITSNAELYGWGSNSSYQCGLPEAKNYFIPTEIPFFKDYFTHLI
ALGEDFSIVKVSPRTDRENTMLFSIGNVHGINAEGKTAEGLLHLSQFDNQNIKIIEAGEKQVVLVYDGELK
ASDNVGIHHEYKCEVTGQNPIIGTLHFYKDNSGDWHFLSREGYNQNKHILPPICFVTKYYIENIQGKEWPE
VNVNEILENSHDEFAPSYLSNVSVKGELIRPMPETDEIGIFEKETNDMIPLIFYRITRPIQDCKALPTIELEKF
HKHCESYRIRLEADPDYSFLKNDGIIGIEYDRYMSIKNRIQNFDEKYDKEILRVIQNYLKVNSQDFIECPWV
FLMAAELEFNEPGIYSFPTDDIQLRIDSLVQFNQYFLKSIPYVLLDDELTHEETIGETILQSESLSASFILGK

>CUFF.24543.1 Membrane alanyl aminopeptidase
MEDFIKKNKTAIMLSAGAILALGAFGFFWRRRKHAAKIDAHGYNLDEKFNDNVCLYWDEAKKRKQIVS
NVSYKLLLALQKGDTFEGHVLVSFDVAEGDFTENDLFLDYHGLGIKDLQINGTNTDAHKAFDGQRIHVA
KELLKLGGRNTVSVTFKSKYRNDGTGVHHFTDPEDNNKYIYTQFEAFNCHRAFPCFDQPDLRAVLSLKTL
APKDWIVLSNGYEKDIVANDSKDFDSHNIDLNSEFLDRYAEGFNLQTYTDTPSIAPYLYAFIAGPYGFLET
NEKIPGRTEPLRMRLYFRKTLKADAERVQQYMLEPAVLGIHWYSEFFGYNYPYEKYDQIFCPEFKFGAME
NVGCVTFTERLLFRGKDITERDYTSLINVVLHELCHHWFGDLVTMTWWNDLWLNESFATYVSYLCMSK
VKSLFEKCPNLWVNVNGY**KN**WGYAEDDLATGHPICKQAPHTDSADDMINGITYGKGCAFLKQLYHLIG
YDKFSLATQLYFERFQWKNTVLKDFLSCLDEADDRIGKKTLGLVISKWADTFLNTRGANVLEAKISGDSL
VITQTVPEFSDGLRQQKIDVLLFDEDFNETVVTVMSSEEDPEIKVKLPDKKKQYVVLNHGDHAYAKIVLD
ENTTNFLSSKLNCIKDSLTRSLVWKAIQAMVKTCRVKSTLYFTYIKQNMPTEDSPYLIETVLTTAAGVMG
SYVPDECYDDIASEIFDVFYKLLPTKPAYKDSLFNFLRSQAHVEQSVAWLEAGSVLYEGSPIKGCELSKNN
KHTILKAVFSKPGFSREEKDRLLKLVVGDDKSDISKNLALSCEALIPDAKSKEKIWKQISDMDSTLSSYER
GALMGYFFNRDSAEILKPYYDKYLEAVRAFANCGNRDLMQDFVGHVCPNFSITDSFIESLKKIEAEFKSD
DKYDSFCRSVGEAAGVLEINQKIKKFAQS

>CUFF.27098.1 HELP domain containing protein
MGTAITKEKTEMPEDLKNLSNFSPGFISKLKDNLESSTYQFSLDKEGLKKLFKCSDKEVQTVFDYFDINGD
GKIDSYEFLCALTMLSHSTLDEKAEILFSFYDFDRSKYITRDELVILITNALTSLNAMAKKDPPKIKEIEKET
DDFFSQADLNSDNKITLKEFKRYLKKNPVILNILMNFNIAKMEDLGTDFGGGDVPECDSDLENEINPPELN
RKGKITQAKQGVEFAVAESEGGLFSIDEVGGGDEFMAVKPWKGVIDHSVPSNYKPSKSDGAEPDANLKL

EYVYGYRCHDVRNNLRYTNEDHFIYHTAALGICMNPFKNTQKFHFGHKDDIMSFALHPNGKIVATGEIGP
KPLISIWSIENMEMITSFNAPLTKGVSQLAFSPSGKYLAGGAMDDDHCIAIWEWENNEKALKSKGKVNSLI
ASGKGSRAAYHAFVFSPSENELVTTGVKEVRFFTYDKGVLKSKTGTGWGKVNQQSVLCGCYVGDTLVT
GLYSGALLLWKGLSIKKNIEAHKSACNAVYSRTAEKGFITGGNDGMVIIWSESFEQLKSLDIKDPSINSYIP
KVRSICENAAGTKILVGTRGGEIIEFTGKKSVMHLKSHCEDELWGLAVHPSEDQFYTVGQDCMLAIWDIK
TRRQLKFARLDCAANVIEFSPNSKFISIGYTNGQVTVLDSKSFAIKSVRRDRKKEISEIKFDPQTKIMAVGA
HDSMIYLYQVEKFKPLRKLKGHHSAILHIDFTEDGSALKSVCTSYEILFFDTTTGKQITSGASDYKDEVWD
SYTARLGWHVQGIWPPCADGSDINSVDRSPDGTVCATADDFGFVKLFRFPCPVEKASFKQFIGHSSHVTR
VRFHKKINYLISTGGNDKSVFQWKYEIDKEGEQEVENEVDLGELGDEFVTNKYEGDEKLPPAVKEEDED
GLFGLEDVGGGDERMAVLPFKGEVDNSWPSDFSKNKNDSDIPEGNLKIDYVHGYRSFDMRDNLKYADD
PNEIIYTTAALGVVLKKKENEQRWFNQHRDDVVSMDIHPERNIVATGQMAEKGRAKMIDCYVWDSDDC
SIIQKLTGFHLRAINVIKFSPSGQYLVSVGEDDFHSCAVYEWKTGRLVGTSKVGGDKMLTAAWKNDKEF
MVAGVKEIKFFTLNGSKLESDKGLFGKTGNIPICSSTFTFKDQLATGTSKGKLLIWTGRKVSKAVQLSKDS
GQMWAICSTKSNLIVGDSTGTVYFLDSKFEQKRKIVVNTQFNPQIRAIDYLEDSDMLLVGTRGAEIYEYV
KSDKGKCLMRGHFDGEVWGAATHPSETLFVTCGGDKTIRVWDTRKMIAASEPFESDIKSCDWSSNGKWI
CVGGANGKAFNVDAKTLAVLGEVTSILAKKSDHCWIEDIKFAPDNTQFVFGTHGGLSKIEFVKVEDNGKI
SKGKVVDVGMSSALTHLDWSTDSEYVVVNSQGYELFWINTSSYERVTASSAKDIDWYTWTCVLGFPVIG
IWPGVDMTDVNTCCRSFNRRILATGEDSSKVKLFKYPCYVEKAKFKEYYGHSSHLTEVKFTVGDNYLVT
VGGNDKTVIVWSTDFGKDTGEDDEEDKDAEIYQDDDGEIDIAKDEFDDSENDIGAVKQKKFEQKQTKRE
KVAKPVDEMGLFEEEDAGGGDEFMAVKPWMGQMREPSDFRKAPKNQEQPPTIKFDLEWVHGYRARDS
KNNLSILKDGCIAYHAAAVGIIYDSDDHSQKHFNKHIDDITSIAFSSDERTIATGEIGPKPSIYVWDGCTMQ
ELVQIKGKLKKGIQALSFSPSGNYLAGCAIDTDHHVAVFNPKTGDCLAMDKGDGNMIVDICMKNDNEFV
TVGVRHFKEWSLNNGVLKSKRGKFGKGQYSDMIVSCKFYKNNCITGTLKGEVLIWNGTTISKGIKGKHD
GPVDAINVWNDYIFTGGRQGNIVVLDSKFNVHNTWDISKFDSICPGINAFAYDGRRLIVGTRGSEVYEINF
NLDSSDIKVKNVVTAGHYSPCRKDNNEVWGLAVFPNKDLYVSVSDDATLRVWSTSERKMVKLVKLNY
DSTGKEIELDPTTKEFSNSVKARSVDVSPNGKMAAVGFREGTFRIYLTKDWKLATEKKDRKEWIQDIKFS
PNGEYLAVGSHDNFIDVYNIEQSFKKFTFKGHSSFITHIDWSESGEALHSTCGAYELLYWDMGSKKQDTS
GASNFRDEKWYTWTTILGWPVQGIWEKGMDGSDINAVDRSPKPHPDGYQLLAAGDDKGKVRVFRYPSI
VENSLSVAGNGHSSHVTMVKFSMDGERLYSAGGNDTCIFQWKVTI

>CUFF.12124.1 WD-repeat protein, putative
METELDLAAVIGFQGKVVDGLILHPDNEHLLFPLGTTIVVRHIISRTQQFLRGHENKISVIVVSSSGKYLAS
GQYTHMGFLADIIIWDFEKMELMHRLKLHKVQIQTLSFSFNELYLASCGGQDDNNLVIWEVETGNALCG
NPTGTNAVKQIKFYNNSDDKIVSIHDYQVLIWTADFLKKKITTQSVNLGQIKRKFSCIVIDHSDSFAYLGT
KTGDIFEISLEMALFKRIGPVKRLFSLGINCITQLPNGDVMIGAGDGTIAKIGYKDMKLKAEAQVLGAVTSI
SLTADGTHFFAGTSKSTIYWSDADTVNPELRNTCHYERINDLAFPHEYSSVFATCSMNDIRVWNSSTRQEL
LRIEVPGLECFTVAFMNDGKSVISGWNDGKIRAFLPQSGKLFYVINDAHNHGVSAITATNDCKRIISGGDQ
GEVRVWKIGSQTQTMEASMKEHRARVSDIKVNHKDDQAVSASHDGSCIVWDLENFTRIICLFESTMFKQ
TVFHPDESQLLTAGSDRKVTYWDCYDGQAIRMLDGSDSGEINALDITREGEHFISGGDDKLVKIWDYDEG
ICYYHGTGHSGAITKIKVSPDQSFVVSVGSEGAIFIWSTPEGILNAKKDKDMPTGPLDE

>CUFF.25561.1 dynein heavy chain family protein
MKGSPYAKFMLDDILDWEKKLLRNQDNLEVWLKVQAVWLYLAPVFSSEDIMKQMPVEGRNFREVDRA
WKNLMMRVNEKPAALVVMEIEELGEILNGANAKLEQVQKGLNDYLESKRKLFPRFFFLSNDELLEILSET

KEPLKVQPHLKKCFEGINSLEFDED**KK**IHGMYSSEKEHVPFKNIIDPIAARGCVEQWLSEVEETMIKSVKE
VCESAIQDYTKRPREKWILMWQGQAVLSGSMIYWTEESEAAMNKSGVQGLIQYFDILTTQLNETVGVVR
TEIDKLQRCTLEALIVLDVHNKDVIKQDLIQHNINDPNEFAWLAQLRYYWMDNDIWIKITNAVLEYNYEY
LGNSARLVITPLTDRCYRTLCGAIHLNYGGAPEGPAGTGKTETVKDLSKALARQCVVFNCSDGLDYLQM
GKFFKGLASCGAWSCFDEFNRIDLEVLSVIAQQLQTIQKAIELRKEKFDFEGSIIPLKFTCNCFITMNPGYA
GRSDLPDNLKALFRTVAMMVPDYAMIAEIVLYSFGFTEAKPLSGKIVTTYKLCSEQLSSQKHYDYGMRA
VKSVLTAAGQLKRKYMNQNESILLLRAINDVNLAKFLVYDVPLFKGITSDLFPGVILPTPDYGDLMSSIRN
QCVALNLQDVEYFMIKIIQLYETICVRHGLMIVGRPFSGKTSSIKVLANALSELAEKGLMGEMKTQCSTLN
PKSINMGQLYGNFDEISHDWSDGVLAVLYRLFANSQNHEIRKWLIFDGPVDAIWIENMNTVLDDNKKLC
LMSGEIIQMSPNMNLIFEPMDLDVASPATVSRCGMIFLEPELLGWAPLYKSWLKTLPEGFDAEIETIIDDLV
NWLIPPMLDFVRTNAQETTPTQNQNLVRSCLRTFRVLLKVFEEDKTKLELPKKDIATIIDGAFLFSLIWSIC
VSVNTEYRKPCNDCFKKLLQGDLDAGTKPLKKVIFPDRGTIYDHFFDPAKNKWQHWNELIDKDEKIPSN
MLPQDVIVTTSDKVKYSYLLELFINNDIPVLYVGPTGTGKSIYIKNVLQISLPKDKFTTIEVGFSAQTSAYQ
VQEIIDGKLDKRKKDHFGPRFGMKCVIHVDDLNMPKKEYYGAQPPVELLRQFLDQGGWFDLKDNKHPF
RNIIDTMLICSMGPPGGGRTFITPRMQRHFNVVAFTDCDEGTLTHIFKTILNWHYSTNGFNTDVAKMEDKI
VAGTMKIYNNIQLDLKPTPAKSHYTFNLRDFSKVIMGICMCDKNKCTTVDTTIRLWAHETMRVFGDRLIN
NEDRMWMLEAVKDTVRAPFSASFDTTFRHLDIDGDGKIDKLDEIRGLLFGDLLIPYGMTERPYEEILDIEK
CIESCEDALSQYNIMTDKPMELVLFRFAVEHLIRISRILKQSGSHALLIGVGGSGRQSLTRLASKISDFNVSQ
IEIKKTYGMNEWRENIKEILRQCGGKGEITTFLFTDTQMKEEGFLEDINNILNTGEVPNLFPPDEKSDVCEL
VRPVAKAENKCPDGTPAQLFSFFVEKCKKNLHIVLCFSPIGEGLRNRIRNFPSLVNCTTIDWFSEWPPDAL
DSVAKRFLSTVEMESKVRDSCAHMVRYFHEATVSASIKFLNNLKRHYYVTPTSYLELISTFKKLLKDKRD
EILGLKDRYENGYECLITTEGQVSKMQSELEELKPQLIETSKLTDEKMVKVAGEKAEADKIAAKVSVEEA
EAQKIADQVSAIKEDCENELNKALPILKESEDALNCITAQDISYIKKLGQPPDDVRMVLEAVCVLQGRKPK
RSLDPNTQKVTYDYWTVSKQMMNEGNFLKSLLEYDKEQIEQATIDQLKKYIDNPKFTKEALKSVSEIAAN
LACWVIAMNKFYHVNLIVKPKQIALEKATKEKNQVMAELAIKQRELKEVMDKVQLLEDDLNITKQRKD
ELEAQVEDCTQKLDRAQKLIGGLGGEKKRWSETAAHLKVVYTNLTGDVLISSGMIAYLGAFTSAYRSEL
TSDWVKNCLEREIPSSGKFNINSVLGDPIAVRNWTICGLPSDQFSIENGIITSKARRWPLYIDPQGQANKWI
RNMEKDRKIDIIKFSDSTYMRTLENSIQFGKPVLLENVGEDLDPSIEPLLQKQIFKKGNSFNIRFGDTTIEYS
SDFNFYITTKLRNPHYMPETSTKVTLLNFMITYEGLTDQLLGILVAKEKPDLEMEKEKLVINGAKNKTKLE
EIEDQILKTLQNSSNILADSKGVKILSDAKILSDKITKEQEEAEITEKSIDEARLEYKPVALKTSGLFFCISDL
ANIDPMYQYSLTFFIFLFTTAIQNSPMSEVLEERLNSLNSEFLYSLYRNICRSLFEKDKLIFSFLLNIKLMELA
CELDFDEWRFLLTGGISLGETLPPCPAKWLEEKSWGELCRCSKLPAFKGLLEAFGKHVDEYEKLYESQNP
QDLELPGGWSEKLNNFQKMIVIRCIRPDKIIPSVYNFVKQNLGEKFIDPPPFDLASIYQDSTATTPLIFVLSPG
SDPLQSLQKFAESKKKQLTPVSLGQGQGPVAQRHIKEGVVNGNWVVLQNCHLAVSWMGTLEKICEELSP
DPKKTNREFRLWLTSYPSKNFPVAVLQNGIKMTNEPPKGLRSNLLGSYNVHPISAPEFFDGCKKPREWRK
LLFGLCFFHAVIQERRKFGPLGWNIPYEFNESDLRISVRQLQMFLNEYPDKIPFDALKYLTGECNYGGRVT
DDKDRRLILCILNDYFTDKAFDDNYKFSPSGLFYAPKTGSYDSYIEYIKNLPLAPLPEVFGLHENADIAKDR
NETAEMFESILSTQTNEGGGESASVEDTVIEVATKILEGFPEPFNIKDAEHRYPVSYEQSMNTVLTQELQRF
NGLISIIRSSLKDLKRAIAGEILLSSELETAMNSLFDGKVPELWMKHSFPSLKPLGGYVLDTRQRLTYFQK
WLNEGIPRLFHISKFFFTQGFLTGAMQNYARKTKIPIDELEFDFEVIQEEDPVPPVDGINIVGLFLEGCRWD
EETMLLGESKPKILYDKCPILWLNPGKITDMKQFPHYNCPVYKTTARRGVLSTTGHSTNFVMLIKLPSDKP
QSHWIKRGVALLTGLND

>CUFF.5262.1 hypothetical protein RFI_33639, partial

MSIGSVSISVENLTQFPLNEKHDQYFTLFDYLEDDLFDGTIGVDDSELPMIQIRYHATDSPLSRATYESRKE
ETVVTKTKTVIKEKTTTSSIAPVVRDANLESSDAYSVKTLTTDLRSGLNQLVDELKAEQSDIFNYEDARAD
TLAHLEKVHKELEQEHINDQVSGAELKRLEKEIISEIGIAKSGFEDDKQSLGRSIAHTDEQLKEKDPQLKAK
QDTNVELNKTLSQDEKSVYHTPQAAKDLRKSNDDVRKKMEEAASNVRKEREDKAKVFNQHADLVNQY
NDLIARYEKLLLDAETARRDAERDANATTGEISVESAEGTNLEHYLEATNKSSHYHQNQVDALKADFNS
FTKHYGNFVSQLNKFVQAQNDEAARLKKNFDDQNKSISQLQGQLSESAKKIVDLHAVVDKNNAANLNA
KLSTLIGTLIDVEKTRRTSQNDLENSQENWSYKVRTFLDEAARASRENANVKRAKEVENLLNKLDRLNRE
RNEIAKKRDEMEAKVVTDRNRDAVNENFDNELDGLNSKLRWANDEIVRTHNDLQDLLKLLATKRGFIT
DQEEQIRTLKEELTTIIRERIGGGTIDDVIKSLDDEIARLRAEIEDLDRRIAELQESIALKDAEIDELNRLLQER
LRRIKELEALLKGKSYVAVKGDLVDEMLAQYIQNCPVPVKRLGGGFYLFGLKKIFAKILNGRLVIRVGGG
YMVIEKFIETYADQELTKLRKVAEREGVADFMQLDLEMIALGPKVPLGSSPTSKSPTGKSPRTTFNSSMNS
SINGTNRKTFTGSAKAIVRSTGTTETTVVTTEKIKKQ

>CUFF.5111.1 kinesin motor domain protein
MADSSVKVAVRVRPFNAREKGLNAVCCIKMNGKKTTIVNEDGGERDFTFDHSFWSHDGYIEEDDGYLR
RDPSHNGTKYDDQELVYNKLGLEVLENAWNGYHCCLFAYGQTGAGKSYSMIGYGQNKGIVPQATQEIF
RRIEANDDENKSYEVTAQMVEIYNEKVQDLLIDPSKRPGNGLKIRENKVSGVYVEGVMKKPVSSYDQIE
AIMELGNKHRSIGSHAMNATSSRAHTIIAIEFKQITQSAKIKTEKFSVINLVDLAGSEKAEQTGATGDRLKE
GCAINKSLTTLGQVIKVLADKSSGKGTKEVVPYRNSALTRMLQNALGGNSKTLMICALSPASSNYEETLG
TLRYADRAKSIKNKAIVNESETEKLIRELKEENDRLKKMLESGGYSPGSGGEDEEDFKKMIEENQKEMEE
REKSWAQKLKEAEEKAKSEEKKVNLNVPNIANLSEDPQLNKIAVYDVATDGKVYVGRKNGDPTPKIILG
GSGIQKNHAYFENKGEVISLIPNSKEANGQIKVNGKDIGKGLKLKNNDRIVFGASSVFLFRMPGELEDSTID
YEMALDEVNAELKAAQEAKMEEQKKEDEEKIKEIERKYEEEKKAEEAKKLQELQDYEAKIKELESKINK
ETEEDEVEKNEKLKRKIEDDMKQKELEREMAEKKLLEEKEEQVKLLERKKKEHQRLDEVLNTLLPLVKE
GNISAEELKKKYNFEPTIVQEIDDKPGMSPLEELKNSKSSVKVKVTNKEDGYHYFWDPEKFTNRLYMIRDI
MDKYFESGQKPKLEREEDPFWDPQEAVLIGRSYLYLKSLGYMLDNESSCKIMNTNIGGDLGKLVCNIKPT
DETGEAEEAPEELFVDNPSELLGKRIDFNIIISQANDLPEMLCKDTYVKYSWYLDNAEFRTETYEGIDRNP
KFKYKHHMTVDCVTEDLLKYFENDALSFKIYGTPTQEKFRKQITTKEEKAKADVKNKPKPEIGASKATKI
TTENKTVKVETIKMKTPDGKEVEVVKKGGGCCSIF

>CUFF.1049.1 cytoplasmic dynein 1 heavy chain 1
MSPYVKNFEEEISKWDEKLQEMRIIFDIWIDVQRRWVYLEGIFFGSADIKTQLPQEFSRFKTIDADFIQLMK
VVSVKPLTQEVYGIPNLQKTLQRLSDMLNKIQKALGDYLETQRSNFARFYFVGDEDLLEIIGNAKDITNIQ
RHFTKMFAGISTLKSPDGNVLEGMFSREGEYVNFVSKLAMSDDATIYQRLTKIEGMMQNSLANELQKAV
EALEIIDDFSQESDPYAKTSSTGQMTFLGWIESFPAQIVLTAMQISWAQRVEDSIKKSKREMTTTEELIGKY
LVLLAERVLTDLPKDVRQKYEQLITDLVHQRDTTRHLIDKKVQSIEDFSWLYHMRYYWNEKEKEAVKK
VRILMANAFFYYGFEYLGVGEKLVQTPLTDRCYLTLTQALHFRLGGNPFGPAGTGKTESVKALGCQLGR
FVLVFNCDETFDGNAMARIFVGLCQVGAWGCFDEFNRLEERMLSAVSQQILTIQTGLQEQTAKIELHGSQ
IKLNPAMGIFVTMNPGYAGRSNLPDNLKQLFRQVAMIKPNREMIARVMLYSQGFKIAEKLSGKIVSLFEL
CGDQLSNQPHYDFGLRALKSVLVSAGNLKRAERDKQNDGIEGSEVEKNILIRSVCNTVVPKLIAEDIPLLS
KLLSGVFPGSDIMKIEEEELIDCIKELLNTRYNLMYDERFISKVLQLMLIQTLHHGVMMVGPTGSGKTSAY
KLLQDCIQKVRKVKIDAYVIEPKSICKDDLYGKLDPTTGEWTDGVFTGILRRILDNVRGEATRTHWIIFDG
DVDPEWAENLNSVLDDNKLLTLPSGERLSIPPNVRIMFEVESLKYATLATVSRCGMVWFSQEVVCLDMM
YFHYLERLKQVNYDSLIREDAVESTDEDASKKAQPAFEVDNSAQSAKKAREVCVEKMKRLFMKDGFITQ

CLEYAARQNHIMKFTEIRVIEAMFALIRKGVNLIVQYNEERSEFPLSESQIEAFMTKWVISSAIWGIGGSLN
LAGRIDFCLKIHELTDVELPDPSGPPLIDYEVRIDDQCWHLWKERVPRIDVEIEKVRTADTVITTVDTIRHQ
EMLCSWLSQRVPFILCGPPGSGKTMTLISTLNALTDCIMVFVNFSSTTSPELILRSFDQHCEYKKTTKNEVV
LLRPKLPNKWLIVFCDEINLPETDKYGTQKVISFLRQLTEQNGFFRPSDKSWVTLERIQFVGACNPPTDQG
RHPMTDRFMRHYPLVFVDFPGEESLKQIYSTFNRSMLKQIPVLSGFAEPLTEAMVEFYSESQRHYTADQQ
PHYVYSPRELTRWKLAIYEALPGLDCLEDLVRLYVHEGLRLFEDRLVTQDEKDWCNETINMVASKWFPS
SDLDKALVRPVLFSEYLDRVYKSVDQDKLRNFIQAKLRTFNEEELDVPLVVFDSVVDHVLRIDRVLKQPL
GHLLLVGASGVGKTTLARFVAWINNQTVFQIKAG**KN**YSLDNFDEDIRSVMRRSGTKGERICFIFDESNVL
SSAFLERMNALLASGEVPGLFEGDDYTHLINQCRETAQKMIDSEDELYHNFVKEVQKNLHVVFTMNPSN
PDFSNRTASSPALFNRCVIDWFGEWDEQSLWQVAKEFTKDIELTDESFDNYNPQDETGNEEINPKHASLIS
SIVNIHSSVKQANTRLARNAKKYNFITPRDYLDFIRHFISVLSTKKESLQEQELHLNTGLEKLKETEAEVIN
LQQNVLVKIQAELEIKNTEANKKLTLMLEEQNIAEKSREASIKTNEEVKKMQIEIAKRTEEVNNDLGKAEP
ALRDAQESVNSIKSAHLNEMKAMLKPPDKVRFAVEAVCTLIFGLTSKPDWKECRSYLGRTDFINTVLNFD
KDSVTSKTKKLVLSQYIETDIWDIESINTASKAAGPLAKWVQSLIEYADIFLKIEPLRNEVAELEKKENELF
TKSKELTDKISELEHNIEQYKKDYAILIAEVERIKSEMDKVTIKVERSKQLIKNLSSERVRWDFSSQDIKNQ
NATLIGDCLLSGAFLTYIGFFDHSYRKQLNQEFKIILGNVGIKYKDDISYLEFLSKASERLEWQGEGLPNDD
ICFENAIIFKYYNRYPLIIDPSEQAYEFILKHYEKSKIQKTSFVDDAFMKQLESSIRFGYPLLVQDVEKYDPIL
NSVLNKEIHNTNGRVLIRVGDQDIDFSPSFKMYMITRDSQAQFTPDLCSRVTFCNFTVTPSSLQNQCLNIYL
KNERPEIESKRQDLLKLQGECKVLLRELEDKLLNTLSTFEGSILENDQLISTLETIKKESIEIQNKVDATDETI
HEIELVSDIYLPLSIVTSRTYFTLENMPSIHFLYQFSLEHYMDTVFELIRDNKELNAVPKSDPDTRLKMIFDL
IFIVVYKAFSPSLLYQDKILFALRLAQIKMEGRGANEFDILMKTPTVLDITISSSLVDGRLSEHQLMYLQYL
STQPQFTYLLDHMETNEARWVAFITDPEAENSVPELPGDDELKQHDKAVLDLKHLILIKILRPDRFSFAVK
SFIQQILACKHLDEEELNFSTSVEEKSQAKSPLLLISAPGYDASTKVEELAKILNKKLFSIAIGSAECYDIAEK
NIVSASKIGGWVLLKNVHLAPGWLVDIEKKLHRLSHHKAFRIFMTMEFNPKIPTTLLRMSMKYVFEPPSGI
KASLVRTYQGNVNPQRSEKAPKERCRMHFLLSWFHAVIQERLRYTPIGWSKKYEFNETDQRASLDAVDE
WITKATPEMSDNLDPKKIPWDAIRVLLGETIYGGKIDNEYDQKILNSLTEQLFTPKGFESSFTLFNVPKSSEI
VPLVAPEGRRVADYKKWIDELPSIESPLWCGLPANVDDILKQRQVLHLIEGLKQLQGVEDEEAAGLDASA
GSKRVKWMQKLEATITSLISLLPEALPSLPREEKSLTDPLFRFLEREVLLGNKILKVVKNDLSDLKLMCMG
ELKITNELREVQKELETDSIPKKWRHYKVANITATEWVIDFVKRLTQLRTLASDVNYKTHNLWIGGFFFPE
ALVTATRQSVAENHGWSLEELDLVVQIGVSKLQDDQSFIIEGLCLEGYSWSQENQDLVSSASLSEKLPPIT
LRWTRIGETDSKDISNSTQIPVYLNSERLNLLFSVKVISEQSRSDLYLRGIALVAWSS

>CUFF.21300.1 hypothetical protein OXYTRI_23427
MYMMKARVGFRYYSGRHIVRPKHVKLFIGGKWEESAKGNKFATIDPHTEEIIDEVEEAGVEDVDRAVYA
ARKAFDEGPWRKFSGQQRAECLFKLAHLIDKHREELALLEALDNGKTLTNANMVDVERSIQSYRYFAG
WADKVTGDTIPAHGNFFAYTKREPVGVVGQIIPWNFPLLMQAWKLAPALAAGCTVVMKSAEQTPLSGL
RVAELVQEAGVPDGVFNMLSGYGQIAGRHLVSHPAVDKIAFTGSTKVGLEIMQTSS**KK**NLKRVSLELGG
KSPIIVMDDADLDLAVRISHRGLFYNAGQSCNAGSRVFVHEKIYDEFVSKSVIEVKKIKLGSQFDKDTTQG
PLVSQKQMERVLGYIDSGKSEGAKCLIGGKRHGDKGFHVEPTIFAEVQDHMRIAREEIFGPVMSILKFSTI
DEVISRANDSDYGLASAVFTQSLDKAIYLSNAIRAGQVYVNSYGGQITAPFGGFKNSGIGRELGHLGINMY
LEDKTVIMARPE

>CUFF.3620.1 UNKNOWN

MADKDMMNENVNDVDLESGTANIGSEDLQPNSQRDKLEKNISIEDLPLQERLTLVKNLANPFGSLKPGLF
IDALDSINTWCVATIVEVDENMLKIHFDGWPNKWDEWMRITSYKIAPFRRHSIGYTGQTKVAIRKIDTTVE
EYEALIDKIDVLIKNDLKGLGAIETTQFYRGYIFITLDHMMGRTYEPNEADLFEVSVKFIKKCLELAAAYL
KLIPKLLPKFTEAKKEPELYLIDENVAIALCYHEFTEMLKFIFSCNPRVLKYYLKYDRDPIKFKTSICKEFES
RDLDMQRLRGEDLDFEEIREMVSKKRELDFGLFYEFLDHFHYHDGFESIRNAIKCILPQENSNTLPFELVPII
TSPFKNCGSIISQEYSKVMAEEIQATILGRLESMNDDEMKEIEKTTINSLLTELREFLCISLDESSVDEKLETV
KLTIALRFLKSTVMKKRLTGINEIKGIIEMTTDNIRRNWPDEDSPRRSKWIKPEYLCRWIYDNKLVEYLLG
DSSHVELIKRSASVLRFLSVHKQLTKDHLELLWKCQDGKHEANVLGVFETIIEICVDLSVDSLDYIFNKISL
IPIKKYNEHTLNFVKDFTTNALQVCKSTERTELIEISSDEEENKQQELYFENAKSFVDGNEVEGVEKTSEYG
LPIIYEIMQQNTELSGSALKAFLELLKHRCCENFRMKYILNSIRNLRNGVAAYQSISTIISILPRCYSY**KK**YQ
SRSKLQIVISKLQTEFDLILLTISSIDNYSTLVQKSMVDSVNKGIVPENISKTCFEGGVIHSEQLEKRLEFIEVL
IHWSYGEVQLGNANIDKLWKIFITHAGFEFDKNLFLKWLSKEKFASTMSQSKEYRKIFSSEEREHLFGSIFC
NSEIVDRKEISYNCFKCFEKYFGFYNREKEYVHYFKGYYTVYYFESIKGLETLWEIVIKSEDEKVKEESSSL
LCSLHLNLYENNYDIDRKFDIWKLFVEQCQHYLNQENGKKTINSAIMLLIKFFDVYDGRCINTSEMSLSTS
FPVHVYCQDDNS**KK**AVNIPYNQNIAYLRKMIADAYDISVNEFSLYVNQKLVSMEDNETLIKEIGFGHVFV
IKKNQISNTEHHPKQLLVGNQEFFNLMFELLSHDEDFDVENIWKLLMKLPQDEVPAAKRIENLELKDENS
WEELIDGSSLHKLLYSLQIVNRLLQIESDWQNSFLVMRGFHHLFHVFIKIDPSKVNSHLAFKSVDLLCKIIC
DSMEKHPDLMNYFKESSLIAIEKLLRLIHQVTSMSIVELKKRGESYDDLYYKNNQSKQKNFRLLSYYDDK
GKNESEDEQNQYTRQIQALNAKFDQVGKFVNLSFKLLFYLEAYNNEECINVMYEYSDISELLYTVLLETD
NFYIRDQFADGLGDILCNQSNSLKRFVDLKQKILHALLFDISQKLELNPTHSYKYFEILGKLLSTTPTALLT
KMDIDFEQVLDMEIKIIFEKETTEKSSNDYDTILCGSIKIVRILLQLFPNLKEKYGKTLLCFLLKDCLFEVPT
QNRSRHKVRPPKCKNHTTRSEVFRLISVLSRDSLENLDAVLSYIKGLQEKSSWRTRKNSDWCISTYHEEKS
TTGFVGIKNLGCICYMIALLQQLYMIPTFRQCILAIDDPKKNEIPSEDNLLYQLQCIFAFLNQSEQQYYNPQ
GFTNAFKDWDGNPTNVLIQMDVDEFFNMFMDKLETAIKGASQEKMIQDHFGGTYANELICKGCPHYSER
SEPYLAINLQVKNKKSIKESLDALIEGEMLDGDNSYYCEKCDKKVPTLKRTCIKRLPKHLILVLKRFEFDY
DTMQKMKVNAYCEFPEILNMEPYTQAGLKRREKSKVEKNDAEENEETEEQPKHPIELYDYKLSGVLVHS
GYAEGGHYYSFIKDRENDNNIDAWYEFNDENVKEFDKSDLESECFGGEEKWSDMMGHSIYLKNSEKHR
NAYVLFYERISSEDIPCSDDEGDAHATQKDAKEIHNETSDIPMASENEEASITPDLKLVRSATARIPVDIREL
VEEENRKYWQYRFMFSKDYSEFILELCTLWNTKNIVLLNYDTRNRDYHILGLDENKYKEELKKIAKGYPE
NHYLNKNIRFYPDKCLHPEESIDIYQKYGGERVDSCEFEIFKLAATFYLTVTQRAQLKEMVTEFLDLIKAH
LNKSLQACKWLITQFSNTEVLFENLLHCPVPDMRKLTVGLIYCAMIRLYEDEKDLLDKYWQYREESKDS
LNRSLLGNFINIIISNLKITRNFTEYNTHYFSILSRFASLGKEARLYLLKAKIVGRILNFYQGESSPFNDYFND
CSDLNFEVNLTPEIGLPFKLENAKLSLWEELFLRKRDAQIAEASQDCTFLFETLSWCIRSCVLHSEDCQTFV
DDLRYNISLDKREHHLLRFDEKDLMGLISTCNNKLAAKHLGSILIHLCAGNIEFDSMLRQVLLSGINDKQL
DEIKPYFPIFKRYLFIQDENSEIRVVEGIREYFNVLKNNIKYSSFMAKFTSFLVKLCNIHRGVAEFLASCPDE
WDWVIEWIKKTPVPTKGNQQLKSHTKEIATSQYKIKRLEDVQSRNIVTFDDEYDSDDDMYNHKFYKDEK
IDYKHAGQTWLPAEVVISLDEMINIQYYIYNQQKSHWLSIDNEEIAPYMAMIGRHDIKAIEKNKVEIENQY
RKSVQQYEESANHQSMSENERYGYSRDDIENESVSD

>CUFF.16975.1 Dynein heavy chain family protein

MCMQERLLNKMVQVKVNNINNFEWTKQLRYYWETEDDDCFARQTNTRFRYGYEFLGNGPRLVITPLTD
KCYMTLTGALHLNYGGNPQGPAGTGKTESTKDLAKALAIQCVVFNCSDGLDYKMMGRFFSGLAQGGA
WSCFDEFNRIDIEVLSVIAQQMLTIQTAVRQQKEKFFFIDKEIPLNLRFGVFITMNPGYAGRTELPDNLKAL
FRPVSMMIPDYALIAEIILFSEGFETAFDLARKMVQLYKLSSEQLSKQKHYDFGMRAVKSVLVMAGSLRR

REPNSAENIVLIRAMRDSNVPKFLEHDLPLFEGIITDLFPSVNVPFIDYGILQQKIEENLT**KR**KYQLIPKFIRK
TIQLLETMLVRHGNMVVGEASTGKTTVCEVLADALTELHSEGIKDRLYYPVEQYRLNPKSVTRGELFGY
TNLLTNEWTDGLVSKLVNDAVNSEKPQNLKWIIFDGPVDALWIENMNTVLDDNKTLCLNNGQRIKLPTT
VTMIFEVMDLKVASPATVSRCGMVYLEPVHLGWECLIDTWNQKIKEKDEESSMYYDLACKYIKSTFKKG
LPFLREECKEVIPSVDANLVASCLNLLESIMDPEKTDFKRAVSPEKDVRVFVIFSIVWSLGANIFDDMRKVF
NRFMKSRITEIDCEFPDEGTVYDYGIEPTTHSFESWFNRVPKFEYNPNASYFSILVPTADTVRYKFLIETLLD
HDHNVLISGETGVGKSVITADFLIHADQEKYVSAFINFSGKTTSKNLKDAFESKLEKKRKTLLGPPSGKKM
VFFIDDVNMPQYDEYFSQPPVELLRQTIDSGGFYDLEKLIFKKVKNTQFVTACAPPGGGRNEVTPRLFRHF
NMIWIPNLSKKSMEIIFSSILRGFLELNPKSSLDIFSDAIVRASVEIYEKTIKDFLPTPTKSHYTFNLRDLSKVI
QGILEIKHKNLDDKEMLVCIWSHEVFRVFRDRLINTQDIDKFNDIVVKLMQKHLNIEWQKEEFVDILFGDF
DSGPDRDYVKLNAPESLVSRLKDFLESYNVSSTSPMNLVFFNDAIFHLTRISRILRSQRGNALLVGVGGSG
RRSLATLAGHMQDMTCFSIEIAKNYREKEWHEDLKELLISVGTEDQQKVFIFSDSQILKESFLEDINNILNA
GEVPNLFAQDEYDNIVEILRPKAKQEGKENKDEILHYFVSLCRQNLHITLAFSPVGEKFRERCRQFPSIINC
CTIDWYQKWPEEALYSVAERFFTERESELSIAEYKESLCRMAVEIHRSSGKEAEIFFEELRRKTYTTPKSYL
DLIKCYIDMMDEQRRIVPQKIARYSQGLRLLAQIKSMVDQLQVTLTKLRPEIDKKEAETQQLVIDLEKQQ
KQAAETEKVFKTEAEESQKLFDEVQELKKGCELDLAKAMPIYEEALRALNTLNKNDIVEMKSYPTPPNEL
VMVIGAVCVLFDKKENWDEGKKLMNEPKKFLDSLMEYDKDNIAEKVVKKVRKYIKMDNFKSEIIAKKS
KAGESICKWVIAIVNYSDVMKIIKPKQESLKKAEKELSKAKAELSEKEASLQQIRDKIARLQASYNTSLRT
LEELTKQKELIEIQLIRAEKLLNGLESES**KR**WEKAVGELNTDLHDLVGNIMVAAACCQYVGVFTDKYRN
RLRESWIRFCTQNNIPISNNLSLERILTDPVTVREWNLNGLPADKLSIENGIYTTNAKRWPLLIDPQSQGNR
WIKKNEGIKVVKQTQGKYLQTLENAIRLGAPVLIENAGEELDPALEPVLLKQIFKRGGQWVLKLGDTDIP
YSQEFNLTITTKLPNPHYLPEVCIKVTIINFTVTPEGLEDQLLVAVVRYERPDLEEQKDILITKSAELKRQLK
ETEDKILKLVSEADEDILNDEELINTLEQSKETSIMINERMKEAEQMTKEINANRELYRRVAVRGSVLYFVI
ANIALMDPMYQYSLAFFTGLFNRRLKLSAKSDILEERLEILIKDITIQFYK

>CUFF.6523.1 dynein intermediate chain
MTDTFTYQKKREEFGRPPNFVDIDTSIVGYIEPIPSMRNMFVQQDPKYLVLDNISKLSEHGVNTERVATKS
KGMKHAEGGWPEGIDPTEPPEVAKYKKKLERDQNFGFAIAVKNTVDEAIVSINQNNEIDMFENYFTGETP
DHQSETITTKTLMLFKDPNPI**KR**AISRITWHPDLTELRLAACYAILRFQQMPKNMLMDSYIWNLSNPNEPE
KTLCPPSPLCTCSFNHKNSDILVGGSYNGTLSFFDLREAKSDGKCYPYTTTILEKSHYDPVYDIFWLTHGK
TGDELVTTSTDGRLLWWDKKKLGGGPTEELLVNEVIAEGEGKKTLGCTRIEYNIDAGPLKYLIGTEHGYI
FQANKRPGKAVEVNQKFGITSGKHHGPIYALQRNPNISKFFLSVGDWCAKIWSEDLKTPIMQTKYHDSYL
TDGCWSPQRPGLFYLTRMDGFIDIWDFYYRQNEVAFSQKISDNPLTCISLHGSRAAIGDSEGSITLMQLCPS
LYETTATEKEEMLKTFEREAAREKNLEQARKQAEIKQPVKTGKDDIDERKRKELEQELLNIEEKFYKMVG
GEQAVQPDIEREPVRVEPEPEPEPEPEPEQEPEPEPEQEPEPEAEPEAEPEAEPEAKAENEGEGQYEAQGEG
EGPNEEEKHSMHHDGSKAHMSASQKSVSHHDAQSKHEGEGGHNADGELAANLGEGDGDEGEGGLE

>CUFF.26159.1 Cation transport ATPase
MSEPAEEKAINTKFKVPLDTLKKLVDSYRQREFDEDLKMIKQEFGGVEGLAERLYTNLKSGITPIDLEERD
LAYGTNAKDPLKVTGFCRLFLQALDDLMLKILIVAAILSLVISMIFEKDHRDIAWVEGAAILVAVFVVSFV
SSYNDYAKEKQFIKLNSYNDAQNNVNVLRDGAAQMINFDHLKVGDLVEIKVGMSIPCDAILIRGTGVVT
DESAMTGETIEL**KK**EPFEMCEQRYEEKLEEEKFAKGGAGGRSSHDLPSPILLSGTQIQTGEGWFLVIVVGK
NSCIGIIMSKLVTKIEQTPLQEKLEVIAKDIGYLGMVAAGITVLVLFIRFFVEQGIKGFNWSGDIGNYLQLW
FEYIIIGVTIVVVAVPEGLPLAVMISLAYSVRKMLKDNNFVKRLAACEIMGGANNICSDKTGTLTKNEMT

VTEFWQGEVKKFDVEAEKYVMNEHIHNQVAAKLFLESCSCNTSGTSKVAGATEKAILKMLDKFGCNYE
EMREKHCKEPLIRFQFTSRRKKMGTILTEIEDNEFRYDKRLHVKGAAEIVLSTCSHYLDKDGVRRELSNDL
KNKIISDVIEEFARGALRTICLAYKDLKESEGGLTHEDDHEDTVNKVVEKFGLTCIGILGIRDIIRPEVPKAV
QQCQTAGIKVRMVTGDNKVTALAIAKQCGIVGTNHPDAVMEGPQFYERIGGLYCDICKEDSPCKCKSEE
TVEKVKNFEAFKSIWKNLDVLARSRPEDKYLLVTGLKQMGDIVAVTGDGTNDAPALKKADVGFAMGIT
GTDVAKHAAAIILLDDNFASIVRACMWGRNIYDNIRRFLQFQLTVNVNALLIAFVGSCILRESPLQPIQLL
WVNLIMDSLASLALATEYPKENLLERPPYAKDEYIISRKMMKHIICMSIFQAIILFVVIFAGDSFVPEDEGY
WPRNGKFVQTGREYDWNGNDLYKKYNENGNDVGPSRHMTVVFNIFVLMQIAHMLCCRKIDDSFNIFEG
VFSNVSFLIIFISITVIQVIIVQFTQDVFKVARKGLYWGQWLFCFAVLFAVFPVDALIKLIPDKLFCDLTKKK
KKDHDDIGGMEETKIEKGKDKENGENVLRERSSGENHPLNSNEEA

>CUFF.27002.1 Membrane alanyl aminopeptidase
MENISKNTKIAIGVSAAAVLATGLLYYWRRHRTGTEEAAKDVQQKTYEDPGYGTNVMLTKIEAETRKKI
LEGSEIMYFLGLALPKGKEYMGKIAIIVELKSLDFNEGDLFVDFQGKAITSLLINQQVVDISKYFNNQRITL
LKEYLIEGHNKVEVTFSNQYRNTGTGLHKFTDPADQGEYLYTQFEPFHAHRAFPCFDQPDIKAKMRLSIIA
PSDHVALSNGIEEFVITSEEDQVFIDDYITDNHFESLIKDKCGYTVTQFNTTPKISSYLFAVIAGPYDVVEKQ
GVIPG**KK**DPIRMRFMCRKSIKEYIQQAYDDMHEAVVTGIKWYTEFFGTPFPWDKYDQIFCPEFKYGAME
NVGAVTFSEGYIPTGKLTEIHLTRLQNTCLHELCHQWFGNLCTMKWWDDLWLNEAFATYMAYLCTSEN
EKLFNKTPGLWIALNTRKVMASNSDTLSTTHPIKKEALTTDSADDMVNAITYGKGSSFIKQLIHMISRKAM
SKACQIYFTKYAWNNSVLDDFIEALIQGCGEANPDLDIDIRSYCIDFLTTKGINSLSSKVEQQGEDIKITFIQ
KQGLHSNSLNMQKVDYQLYDKSMKFEQHSIILEKGAETQSVVFKNRKADDTFVLLNADDHAYFLINIEK
NFINKFIEGDLCKINSSINRAIVWRGLNSMVRGFEIKPMDYIEIVMNNIFNEDDIILLDTILGNLHMFINTYVP
DDKFSDTCTQLFEKLYERYSEIPDSNKDLKNVIKSALLYCIYDEKHIKMVVGWLEDDSMELSAADKETILT
TMFRSSLFTSEEKHMTLNKYVGDNASDRMKRLKITCEAYIPDKENKAKVWEIVTHPKEHELSSYDYRAY
LEGFHSRFQIDLNKEYVDKYIEEVPKFARSGEKDHMTIFVSGAFPPIYYMNQEFFDKIDAIVAEFENEDKV
KYDSFLKLIKSMNESRKAQ

>CUFF.27001.1 px domain containing protein
MDKADEFKGLYIRAKVILFEESLGAYYYTCEIKSGSEASTAKWTIKKRYTDFFNLHKNLLMKYADLPELP
KKTWFKVYEAKDLDQRKRELNDYLMALC**KK**EALTNDGDFHDFINLEENLNNKLHFNKEKLLYNFPDAN
LGVREFILIEEQKIVIAVCREENVQSRVKSYWDNIRLPFIHTEDDYTPVGSFVVFKIVSQDPWHVEKLFIKN
FKSQTNCVAFCKDMNVLAVGLSNGKIRIFDIPKGFKFIKDVYYDSSSIKAHSGQINGIAIDAPLGYVYSIGN
DSKLCVSDRTSGEVVWTKGFDKFELTTLTHDEAKQRLFIGDNSGAIHVFSVKRYPPKRLATIKTSIKLTIKS
ITFSDDYKKMFAGSIEGDILCFDLGDYGKEKKNTREYPYKLKGKGKCQSLVWDENNKCILSGNKSGNIAI
WLPEENRCLYVFNAHLHKVTSLHWDKELKRLITGSSDGKIKVWTLPHAFIENAKHSNITNMPIEEQKDDE
E

>CUFF.24100.1 dynein heavy chain
MYLMSIESSITSLNLICNWIMKPYVSQLDNAYWRSSEEEQKKEFCQVFDKFSKELKEALTSLSGIIELPKYD
KRWDKDAKNIHTLKNPDPEMKKHFQDLFELWNTKIEEYLNESETGPGKPKDRDPGPRSELEYWR**KR**MQ
TLSCLSEEMRSPHCKIVQNVINHVSSNANDNPGRNIYLLQSKWRTQDMKVTEALNEAKDNVKYLITLEPF
IDPLYNETPDVVKDTLPALMNSIKMIHTIARYYNTNDRMTELFKKITNQMIICCKNAILDGETSDDKLWDH
ERFPPEKLIPVLKSCIELNKAYQTQYEITKEKLMNMPKGKQFEFSPNQIFGKFDLFCRRVSKLMELFGTIQQ
FETLGKHHLENIDEIISTFNTSVEQFRKKKHKLLNFEQNSFDRDFVEFNVAVSSSETELQQYIRKKFEVIRSV

EDSLKLLRKFRSILKRDNLRSSLDVQYIVILQNYAAEIAAVEKQYQDRRTKVPTVRNLPEVAGAITWSRHL
FHRISAPMENFPPDLLAKRDSRKLINKYNKTGYVLFAFESVYRQSWSHEVEKAKAGLQATLIIRHPSNNKL
YVNFDSEILTLIREAKCLARIGIELPESAKIVLLQEDKFKRNCNELQYVLKEYERIVSKIRPNTKSLLVPHLE
DLEYKLRPGMVTLTWTSMNIEGYLHHVHSGLSKIEQLIININDIMENRIENNLKALSKTVLVDLPQEAHTF
SLDEFVEMQEKWIKHESLKLKSKNIEVEGAVEDLIQTICSYNLDKHVEAISAEEIQKLSKYYNWSMYQAL
LHATKYSLNAMKERICGRRNQPKMQLKPFFDVDVLLENGQCILKPSLEDIQSAINRAASHVLKSTKNVQN
WNQKDIPEDKREPFYDWIAKDKEIVKVILLLTGSIQGTKNAVHTFVESFEEYQVSLLVSLVNSGFGTKISM
TVLRHLQEA

>CUFF.24121.1 UNKNOWN
MMHNAIAVCSVDGGPDYQVPLVGESSFISYKLSTNEIDFGEVSYCNNSNDNFYIENIGKVPFEFSINLSTITR
PGMLEVSPMNGKIMSGERFRVALKFKPGIPDNISELFLVECAHFPAERFKIKAVGIYPGVLLTLPRHDDTFS
ERFEKTKRLLDKNKVKYDAKFTSSEVKVMTTGKGKQDKFQMDFYQMDIESETDRLYLCEKLLEQQEIVQ
ARTAITFNESKQNASLVQTKKAANASQSKAQAVDPDKSGIIVEDEDKIVVSNYICDFGNMVVGSSKKRTF
RMTNCGKNNLGFSFDTRILQQIGITIDPIKPPKIFPPNTSMQFTVVFATRKNAKHGKVKHVVPVNLSYGPSY
NIEFVANMTIPELSMSTDSVEFNQVCVGTRRIIKVRFENKKEVPCDWWYYYKPDVAGVSAKEGEKFSVYP
TNGQLLPGQKQTVDVIFTPTHEKVISQNLQFKCKENSKIFTLNVKGHGINYALDIIENSIEMGPVLPYDKSA
VKTIDIKNPMSFPIEVYSSDFDKQYLEEEEILKRFEPLNEKGDMIFEKLRKAGQEFWQNIKETVEKKKQYD
EMVAKAKAIQDQIDKEFTVPPPEEGKEPKVLTEEKKEQKNKLEAEKAEIESKIAEIDAENSVVKKTIPKVK
KRDRLSIVLFGPEKCGKSTIAYFLAEEQQRGVVNLNELYSWCEKNSTPTFPEVAKYLTEKEEEWKVQEEL
EKKKKKGKKDDVENPNQVLEWKYLPKELLIKMIVERTSYEDCNAGIIFDNLTSEYWRDERAIIDAICDALS
EENIHLVSITLTKDENGLEYCENFRYKIRKEMDEKPVEKEILDRTFADKDNTQNKQKKGKKKELTEEEKK
KLEEKKKAEEEKERLDQARQEELRKKLDEPEPKKLSDEEKEAYLKKYNEIIDLFTEINLRQMNQKHESNIE
QRENIEKTINSNEENKDIKEIEEDNKDINEVKEETAEEDIPYFGSRILYEVPMQYNFRFLCEQIKHNVPEPV
WPDPDKEPLPPPIIQQIIKKPGNRPERPKVILFSIWTPVDKLLNKPEDEEPQEPEAVDPKAKKGKDAKDKTN
NPKGKEEPKEVEVKEEEKGPLLDKSITRWILQPNESKTLHIKFFSSKIGKFNQTLNFEVLGSSKQFPLDIKAI
CEFPTINSNPKNFFLEQKRQRPANPPDCYLQKCYISNENVFDFGPLLIGKDPEKRHEGDLTKSPNYTEFRIS
NNGKYDLNVKFCLDSFLVSNDPKTIKSPFIFEPESMNLKIDETQSLKVWCIPDEAKVYKDKIVCLIKDNPNP
VVFNVMCTGSKPIVKVDNPIVQFDKLLLNKPSKRTLKIKNDCQIPVKWLLRSNDPLPPEYTISKTEGDLKP
CQDTDIEINFCSEKQDQFIHKMILEVEDAEGFKIKQEPQEIQLLAEAFNISVNIDFKNDENIIDFEAVRVGDP
KEKKLTLKNIGKYPVKYGFNIKKKTIREIFTIEPGEGVLAPEETKDIVVRFESKKEFKMKTTQSTSDIRLTIL
EGGTKEKFNEIPINFNVNAVFSKYTIVPLRNINFGPMQYGEQKDLTFEVKNNGQFPFNFAICDFNDQTAKA
TIKAEMEKEAKERRDEALGIASDEVKDTKGKKPADPKPKGGKDPKGQAAAAEGEELKVSQYTIRPKSGT
VEPNSSTVVKVNFSAKGAQFYEKALSIDISGRDPVDQPEGIRFDLSAESCIPGINTEDLDAIFEEQTVISSLDP
SVNTQSIITSSLYAIHERVFWFGTLIALKVPEGVVEQFKIINPNKIPCTVKFSVKPRTQSKNEGFAFKVSPES
VKIPPHENTYVKVSFIPLNMMAYGGIFEATVENGDPESNSGKFTFELRGEGTLPTLLISKPSQLTEDGFPLL
KFKKTKLNKKSTSTIVLVNEGAVPASVKFKPIKHDNLEFKGLMTTTLQPKEHYSFDIVFIPTKIEQISYQME
FETLHNPFECHKVVIQGEGYQETVTFENLPEELEDELRFGDAIVNKAKNVEFNIVNTSDKPIRFEWNVVEP
GFSFLPSVGHLRPKSSKLVKVKFLTHEPVEIKAGEITCDTKLITQASSQFIDWDDTQTEIKLIRPSELKKILAI
REAKERRKKEEAEAAAAAAAKKGKVKPPPKKENERLPEEDMPIDESEEATEEFAEALPEPQYEPVDGSDK
QLKLKVSATADYARYKCDVDKIIFKPTMMFGSRSFKFTLKNSSTIALHYSFKIINANTGISDSGSFSISPRNG
IIPAGTDEILIVKFSPEEIDKDFSRLLTCKILNLSPDQDPLSIKLDGVAERPVCHFELPPSAYREKKGKNMSPI
DSKYSIIEFDSLGVKVKNTKRFMVVNPTSHSYDFEWEEVESEEPGKESKREMPMFRCLTPKGTILSGK

>CUFF.25403.1 alpha-centractin
EFDNLIFNKQPVVIDNGTGLIKAGFSGDERPKMYFNNYVGRPKHNKVMLTTLDQDLFIGSECDKNKGLLK
LTYPMSHGNIDNWDEMEQVWGYIYKEMKVSASQHPVLISEVIDNPVSTREKIAEKFFEHMSVPALYFQSQ
PILSLYAQGKTTGIVVDIGDGVSQCVPIVDGYAIKGTSSRIDVGGRDITEYLMLLLRRMGYNFHTSAEFQIV
RSMKEKLCNNSLTPIKEVDYKNIDEKNFQEYFLPDGGCVKLVTEHIEAPEILFAPQKIGLEYVGIHSMIYNS
IMKCDIDLRQTILNNMILAGGTTAIKRFSERLHKNLSVLFPKESKIKLHAPKNRDISCWIGGSTLSSLKAFNE
MWITKKDYQEVGHNIFRQYM

>CUFF.25551.1 serine hydroxymethyltransferase
ITLEHSSLLSNSNKACFGTVKLPQEDKLLKDRDPEMAALVREEIARQKKGIILIASENFTSSAVMQAVGSPL
MNKYSEGYPGRRYYGGCMVIDKIEDLCKKRALNAFHADPEKWDVNVQALSGAPANFTIYTALVPPGGR
LMGLDLTCGGHLSHGYQTPKKKVSATSLFWDSQSYGLKDDGYIDYDGAYETAQKFKPNIVICGYSAYPR
DLDYKRFREIADSVGAYLLADVAHYSGLLVGELLKSPFEYADIVSTTTHKSLRGPRGSLIFYKKELAEQIE
DAVFPKMQGGPHNHTIGGISVALHEAQSPEFKEYTKRIVSNSKALANALMKRGHTLVTGGTDNHIVLWN
LRPHGLTGSKLEALSNYANVTLNKNTIAGDKSAVTPHGIRLGTPACTTRGYTESHMDTVAEFLDRICKESL
KIQQGKGKKLNEFIQGVQESEEVKKISRDVEEFALQFEWPGVSI

>CUFF.2730.1 dynein heavy chain family protein
MTDALEEWIKCQGQWAYLQPIFDSPDIMKQLPAENKKFKSVDGTWRGIMKRCKENPNVLAICEDPELKE
AFVKCNEDLDVVQKGLKDYLESKRAIFARFYFLSNDDLLLILSQTKDVQNVRPHLRKVFENLADVHFNPD
NTISSMFSSEKERIEFVEDVDPRDRGVEYWMGDVEEMMKTSVRHVLLKSIEDYLTKPRTEWIKLHPGQCV
LNGSQVHWTTEVEQSFKIGPDGVKEYYDKLGRQLLDTVQLVRVKLTKLQSIALGALIVIDVHAKDVVER
LVEERIDEVNAFEWISQLRYYWENDDCRVKMIQTNFPYGYEYLGNTLRLVITPLTDKCYITLMGALKLN
MGGAPAGPAGTGKTESTKDLAKALAKQCVVFNCSDGMDYLMLGKFFKGLASSGAWCCFDEFNRINIEV
LSVIAQQLLTLFDAKRDGVPELIFEESRICMKPTFSVFITMNPGYAGRTELPDNLKALFRPMAMMVPDYAL
IGQIMLYSFGFSEAKVLAEKMVTTFKLSSEQLSSQCHYDYGMRAVRSVINAAGLLKQQDPELNEDQLLLR
ALRDVNVPKFLKDDLPLFENIINDLFPGVERPQINYGNLVTQIHKSCEIFNLQAENVFVEKVIQLHDTILVR
HGLMIVGPTGGGKTSNYKVLQHTYTSLAGQDTFQKVNTHIMNPKSITMGQLYGEVDPQTTEWIDGVLAK
KIEICAADESPEKHWVMFDGPVDALWIESMNTVLDDNKKLCLNSGQIIPLTDRMTMMFEVEDLEVASPA
TVSRCGMVYMEPVALGFRCIFESWFNTFPPPFKLSEKLAIKIKDYVNKYISTLLDFMRRNCPEPVTTTNNN
LCQSFCRILDCYFSNYYDNEAHIITTEEIDEFEGTIECLLCYATVWSFGCTTNFEGRKKFDSKMREVLGGYS
GTPPREGLVHDFCYDNKNKIWTDWYDTIPSYQVDTKVNYTDIVVPTFDSIRMKYLKKLLISNKKHVLCPG
GTGTGKTVNIEELLKNEMPEEYQSLIITFSAQTSANQTQSALDEKFEKRARGIFGPAPGKRFIIFIDDLNMPK
KEEYGAQPPIEFLRQWLDHGGWYDRESKEKHFKKIEDIILVSAMGPPGGGRSNITARMTRHFNMITYTNL
QESSIKQIFATIVKAFLGNFKPEVVDCLDKIVDMTLKIYNNVATELKPTPTK

>CUFF.128.1 malic enzyme
MELHEDKDKSKDTTPSMRRKVTKEEAHAYHQFPIPGKYQIVPCKPLDSEKDLRLAYTPGVAFPCKEIQKV
KSRIYDYTAKSNSVCVISNGTAVLGLGSIGALSGKPVMEGKGVLMKKFGDVNGVDLEVNTTDPDEFINC
VKLLGSSFGGINLEDIKGPDCFYIENKLKEIMDIPVFHDDQHGTAIIVAAGLKNACLISGKKLDEIKVVFNG
AGAAGIACLQLVIDSGVKKENAILCDTSGTIYKGRTKGMNEWKEKYAVETEARTLEEALVGADCFVGVS
AKDALKPEWLKSMNKQPIVFALANPDPEILPEKAKEAVPDVIIATGRSDYPNQINNVMCFPYLFRGALDV
RAKNITEGMKIAASDALAQLAREEVPQEIKDLYGRDLEFGSEYIIPTPFDPRLLIRISTAVGKAACHEPGVA
LFCYSDWSLYERHLQERIDRKNAQMTQMTSEDVEE

>CUFF.1718.1 Vesicle coat complex COPI, beta' subunit, putative

MKLEIKKKLLNRSERVKSVELHPTLPWVLIGLYSGTVAIFDYNTQSCIKQFEVSKEPVRCSRFITRKEWVV
AGTDDNSIQIYNYATSEKIKTVSAHGDYIRAIIVHPILPYIVSCSDDLTIKIWDWDQGWKEVNCYEDHEHYI
MQIVLNPKDPNTFASASLDKTIKIWSISLTTKTANYSLVGHQAGVNCVDYCHSGDRSHILSGGDDAMVKL
WDYQTKQCLFTFDGHDENISAVAFHPELPILISAAEDGKVNVWNAITYELETNINYGLGRSWAIHAAKNS
NYVALAYDEATVVVKIGKETPIVSFNNGRVVWARQGEVQMANLKSIKDDLKDGEEIEPKFKDLGHSEIFA
QDIKFSPNGKFFALCGDSDYVIYSTFKFSNSGFGHAVELVWGQDNDYAVMTEQGVIQLFKNCIEDKTYKT
AFKGHGIFGGKLIGVSTKQGGSSISFFDWENFTCIRRIDVDAPKNVYWSQSGEYVIICLEETFYLLKYNEEY
VKSYLQSKGASSIGEEGLEDAFTFLGDFNEEIISGQWVEGDAFVFTTAKGKLNYLIGDKIINHVYVD**KK**MF
VLGYVPQKNRLFLINKALKISSYELLSSVIMAQREIVKADPGLTTEHPSYQNLKEVISQVPESHIGKVAKFL
DSMNFKEIAYHVTVEEKHKFDLAMHLNKIEEAYEIACKDPSNYEKLRKIGDVSLKQGNIKLAEQCYLKSN
DYNSLLLIYSSLGDAEDIENVGNMALNEKKYNIAFQCFYAIAMPDKCYEILVESERIPEAAMFARAYIPSK
LSQAMKLWIEKTKGKPYVPANLTDIPENVPLFDLAVRIESVLSEYYSKEREPAYYYEAAYERHFKEIAGQ
VDSGEEVELHKPLVFNNEKLEPDNHEEIHTNDDNNDDEI

>CUFF.25021.1 Drp1p

MDSDEEDKTDVKYSQYGKKIKIEEEKGGYLYVQLRRFINVIDELRDVGLQEHISLPRIAVLGQQSSGKSSV
LESIVGIDMLPRAAGLCTRRPLEMRLNHLYEEDAKPWATFIEVPGVKFEDFNKVREKIEELTDKIAGTENN
IVDNPIVVQVYSPTCPDLTVVDLPGLTRIPLAGSKQPDNIYEVTKGMALRYIEDPRTIILCVIPANADMTTS
DALQLAREIDPKGVRTIGVITKIDIMDAGTNARRMLTGQDVPLRLGYVGVKNRNQQSIIDRISVKQALKDE
REYFENHPIYSSINQNLLGTDALTQKCTKIMFTHIKSHLPDIMREIKDKMNQINERLKDLGPPMPSLPKEKA
HLLYNMVTEFCNLFKSTISGKYDSRRDRTTTKEIYGGARLKMLLENLYREFTGKYQATKDLTDLDIEKAII
MHEGDSLPGFPSVDVFVYLITPEL**KK**LTDPALDLLAECHMYIEQLADEIAQKVFSRFPAVVNDIMEIVARC
LMAQKDKANKVVEAIIESEQGYLFTNDKEYMEKCTDIIPMEESSGEPNQPPPKRSSRNQVYVDEIRKRLD
MYFKIIVRGIRDSVPKAIGFFLVNGIQEHIQYDLYAEINKNESMAETLGEPPEVTAERKILNSSRETMERAL
KILQRNPEITATLDFDDQLSRDIKESLKDANKKQVKAKQEEELRKYNPDPSRLNMSSMSEMSDGGSRKSR
PAPGDSSSKSGMPSQSSMPKGPLLERKPVDMPSVLKK

>CUFF.3896.1 EF hand family protein

MSRRSGMRPMSANPPSQSRHNIARALSDKVNTNSVDNVAKIWTAFGKYIEKVLKSGKGVGIPKFGQFTFT
PIKVDLAGSTNPDQRDKQLREPIFQVARDFVLGLEIKSGVVLDNGVIRTFEIKGTSGIVPKVRINYTEIGIFA
GVSKEDAKHGCDIVIRDLSDKVKSGQPTKLLIPNVGTFVCKNSTAGIMFAQDLKNETVGKTAKAHFIGKL
FASSVNRANLDLLDQKVSQGIRSKLTNFNQANKRTPIVAKPDHTITVTNDAENWLKRNLGVTLEGGVPTD
LKSTSSRHRMKRTFSARPGFRSTQSEQQTSIKSEDNDHFQFRKEHEDKPFVIKSGTKKRPMSAYSGGSRAS
RASTVRSAASAVPKITRATALDYCSRIATKAVLDYVQSAGISARDKLTPTELQSVLVNSGIRIEISTVRGLL
SHLGFSPHGKTCSILDLIRTCKDWNSKSQIAQPFDSSTKLQSNSLYSIDELIFKIRDCFYKSGKDIKVIFEAGS
TDGGFVDEEAFVFICKKFCFPSVTEDDSRKVFKAAHKNVGSNLYFNDFRNIFSSIQPKTNFHIEGLKLIRDW
MYSNGLTSDQVFEVFSAGKDKFDLEHFEGQLKKLFNLTSPEVETLFKAIDENHDGYIDSEEWINKVYEDS
NNPLQLLREIVNQNNLNSDDLLFKMNLRIWDDPLDFTKFAKALRILDSSLTDLQLKAVAKSMKNSNNLVE
VPVLIRNLVGKDYETVDFRDKLFKRVYNEIYGDNNERKVDKFRNLLIKYDNLNDGTIVAQDLNKALAQV
CRNIKSDDIERFTRFLEKDVRGRINYTQFIDNLESVKNHNPFKNLMNRIKAFMTQNNQNVDSFMRRLTLG
ESQSDYNAVEEKTIEKERKVSVEFFAKFLKNKVDKKKALEELIQQAELIDIDKDGYISIHDLQSCLGNLSNE
TFYKNNGATLKGTFKTILTEREKFFPKEPLPHLKSLEVIGKIKEALIAKGISFRELFARLDTNSDEFLTFAEFS

QNMDPIIKLSPLVKEQLFALMDVNKIGMVDYDAFLATLKKTAVSAKVIRVNDNFDWEYEIIHKIKDWIKD
EGITVEEAFKAFDRDFDGFIDIDDLKWVLINIIKCGDKSSIKSSQLERLFKLLDFHKDGYIQKCDIQRLMENE
NPYLSTGKTLNTKFMVGSDTFDWKNNAIQQIGIALSKNPKFSSLQDCFKIASQNLGKVRYREFKEFIEENN
ALKGFNLTDQLLQQLFSEIDPHKKGFLTESDWNAAFGGFDWHEQLIVELQNLVACSFADIESAYDYFQVI
GTNKEID**KK**CFINAVNSLRGTKMQESESRYLWKHFSNNHKVISQEMFTAGFSNVRFSGKSTLRKTSKSLH
STTLVSETASSSTWSVNVMEKFRKIIKSSNMHLREIFESFDEDGNGYITPIEFRNAVRKLNINLSAKEIDEIIR
VVDRNLDGMIDWLEFSSKFKTKENEKLIETRTRNKMAKLKEQMTLHMKTPQDAFELFDKSKTGKLTFAN
FNDLIRELSALSKQDIPPFSIIKDLFDEIDIRKDGEIDFKEWNQTFIGVQNGDKKFSLRKMPIHLAEFEVSRD
AKIITEAIVRNRKFLMDKFKEISPDGNHVTFDEAKEIIRAVQRGKEIDDDQYKVIFKGAIKENNMVEFTKLG
KSISTKFA

>CUFF.4515.1 hypothetical protein OXYTRI_07337
MFLGTVDQHYNRLYNAKHFRNRIIRIQKLCVEFKYDAVLLVVGIDTYHDKEMKKFKNWLFYGISGYDME
GTSLNDNFNDSFCVISKDSFQAYTTTAGFKDLSRLSALVPNRNIYCLTKEEEENQELNEVKKIAKFYEMVC
DKGCIGLPAREIKLEDTFINELQMIEKWPICQSYGLDLVGEGFFTMKHQVKNIREQLNIVYREYDSFAVYA
VMYDEIPKTGHYFYDSFYTFLKGTPQKRLDTSEKELIEALSFRYEFGEIGKEEHKREDGLPVPRTLFGLNT
NLENDSISSDIKLWKYGQKNDRIPVHMTIEYVERRTSLRFARTYMLSNCSINYMKGYEQDGDSDSVIT**K**SS
KFCEQSANFIMNSYIALIKSFNIIKDMYFQSKIDKHDYLKVSYACFLKMCEKELNTEIDDLRKIFSIDNFSIDI
NRYNTMGELLSAANPTYLTLYMIRIQVKNITVNNGEENLGSILYADSYYEASGHSLNITKNVPALQCFYT
AFQHYQYLTSHWFTE**KK**VKCERFGEKQFILNDVNGIYPIQNRYIDAITDFILIGKFIVYEKCMIFEDSKLHN
FIIDFNDIEEVVFYISKNTWGDFKVKDTTCFPLNIVGDSKILINFGGHFYSKNLNLLCEYIGESKIKKVTENIE
INVDYFKTLQYKNYKVQTQIQNKDIYTGYLDFELFEKEYKMFMEYTLISSFNQIRSKAFVSYSQFGQIWDT
AQTAYKSLKEESDKTKLILISGIPGSGKSSFGIYLSKLLAKESIQSSTYIMPVVESTKFTSDTFLNGLFKHCEE
NQTKTVVAVIPSYHHLKKAIFEFKKDTKFNETFNLEFVITKVSAKNFYKHQNKNTYQFLLENCLKGICDA
VVFEKGNQSQEEFSLMRKELCEVNDDANILNVRGRTFEWKDLEEILQKKDHKYSLLYGKYFYGFEKEGK
SAYYLENASTGAYFNYRIPLRPDLLEKGIHKVFNNPIQDPSSLIPEEEKRCDFHSTVDSTTEDDSDNKENIQI
GEETGSEKIERRKKKKIEEEQKRIQADLAQEKMLIEEMTKIKNSMKKNSFIIERVKGLFLAEGKELESDYKI
ISNFSDVSVRSCNNSTKRTPPDELGFLVYGKNITFDKFKDVLGAYRTQLRSKLPLRTPLTITKQEKKMLEE
KYFCENMPNEFYHDGYSYVDGNGNRQYEHPNLEKLIEIFIEEENARIAEYNRQVQKEWKQDEIRYT

>CUFF.9099.1 hypothetical protein OXYTRI_19222
MNQDDGGEENKNTPHTIEDTFNSMITFFDQKEFVVIDNGTGYLKCGFSGEDLPRITIPTVMGVKEIQVEST
QGIEQGLKRYNRIYGYESLSHRLEYDTYFPIKRGIIEDFEHMTNCWQHVITH**KN**MGQQKKCDILLTDSPLN
HKDNKVQIAQSIFGNGMKDKVEVDSISIMNSAVLSLFASGRTSGVVIESGQGLTCAVPVFEGYALPHAIKT
INVSGQDVTQFLFDSLVAKGIDINETNMDNVREIKEQMCSVAMDYERAIRGPDPLDEEGRMYELPDQKGI
ITVDHKTRFDSTELLFNPDIAGIKSPGISQIAYESIKQCDLDLQITLYGNIVVTGGSSMIPGFCDRFEQEIFELA
SDTAKTDIKVDVDLPRKNSAWVGGSMLASMSTFENLTIKYSEYEENAENVKDIILRK

>CUFF.1534.1 band 7 family protein
MLTKRFLAKGLMRPISISASMSPTQDKKHKNAGQMIYSPYRNFFSAKEGTMCFFGCVFFVREQYACLIHR
FQKFHKIVKPGLNFKLPFIDSIEYIHDLREQVIEITSQSAVTKDNIVLHIDGVLYIKIEDPYKASYGVESIISAI
TNIAQTTMKAEIGKRTLDDTFKERQVLNNNIVNAIDKETQDWGTRCVYYEIKDIDPPNNIQKSMILQVEA
DR**KK**RANMIESEGERQFKINIAEGKKMSQILRAEGNAEALIKKAEASANALKTICDALKSNKGQDAANFL
LGERYIEAYRKIAAEKNTVILNSSPTSLVENVSESFNLLEKIHQDDKTKIEVKDK

>CUFF.166.1 Otiwi1
MDVDYPFPIKPQSSKMGKTVSIYSNFYKFNLKTGGREVLFEYQVKTEPNLTCHFNEEKIKLMKIVKKMKD
KLEAQFDNHVYWEGFIYSFEKIDESEMLANIEIEEDGVPYLVSIELHSDLSFDNPKVSRFFRAFLNKMIKKS
KLKLVRGGKHFDPRDPAELDGVNMYRAYFNTTKTIDGAVYLNLNPSVKFFQQESVLKSIIELGDERSAKE
ALTGRSVMTLYNNRVYKIDEIDFSKTPASKFFCDMHNKNKEMTFGDYINDNYKIKVTSKTQPMLKHHNI
RTGQDIYLIPEFCVLTGITEQQKGFNFKAIKNDMFANADTKSSQAKMFFETLKSNRGNYKEMTEKWKIEI
DETPTKVKAYQCNFGTIVGNAKKVYNLKSMQRDFSREFNGPFKANKINSWAILYSKYSSREHESFMSSLK
QTVTTDFEYKCNKPVEVIIKGEDKKPDSWIKTIKDLVDEGNLDVIICIAPGRKGNSPIYENLKYFLQTEINIP
SQVILADTIKKNFKSLRNIMKNVMIQICAKIGDIPWGFKELPLMNEPTMIIGMDVCHRVGKHKKSVLGFVA
SLDKYVGKYYSASVSQGEKQEIAFSIEKLFQEAITTFISENKGW**TK**KIIVYRDAVSEGQSEATLSQEVPQLT
QAIRNLVDAKVLSTEPKILFILANKRVEQRFFTQDRGRFANPERGLVIDEGVTRLSRFEFYMISHAGPTGLQ
CPIRYEVIYSTFDSLDPK

>CUFF.17549.1 udp-n-acetylglucosamine pyrophosphorylase
MESDIPEDYDSLRQRFEDKGQGHIFAYYDSYTEDEKLEFLDQCRQIDLDEINRLYNDVC**KN**HSPVDTSEH
TIEPIAKEIIHNYSSLDEETKHNWSFEGAKQIIKGKLGLLVLAGGMGSRLGSNDPKGMYDIGLPSHKSLFQII
CEKFMRAQAYSAEICQRVGMNCAPRFNCMLYIMTHDLNDATTKDFFISNNYFGVPENRIMFFKQASLPPL
TFDGKIMLESKAKLALCPNGNGALFEAIRSNKAFQENLKSNKIDYIHIVGVDNALNKFIDPLQVGLAYSKN
LKGCAKFLQKAYPTEPIGVFVVNNGKTDVIEYSELGEEMANQKNDDGTIKYNQGNILNFLVSVETLNDLV
FGKAEVLNSLYHLAI**KK**LPFYNEETDKTEKPAKENGYKLELFVHSFLPYVEGAFEFIEGIREEEFAPVKNK
EGELKDSPTTARCMISKLHSSWIQKSFPDVKFKETPSESFVVELDFMSTYQGENITEDMIPIDLLEFS

>CUFF.24098.1 hypothetical protein OXYTRI_08272
MNHAQAMRDLRKIEDHFERYYEYKVKRLQATFSLDRDTAAAVLEFMEMKIATQTFSGTCGILAGWYVH
SKLTPLLMNRSLLFKKPWMKPILPLVAVYLGYCTGFKLRMRRLAGRDPNFEKMSGSTDLLSRFREVHAG
KEITSQERILEYITTCTPMSGSEIRKDLEKITNAGLKKYENKRVKRQGKDKDDIFWLMGKIHGLENIAFLTK
EELDSIDGNPIRLQHLVNQAKPTGHIASSFNECRDKLNDTLAKYKEAVNKLNLTMSDRSKLLALPFFCGR
RTQGPAPQKGQWQWDLFTELSGGKQWNQYDHLEEDPEHKINYHEYEKYFPEGLVNKIDSQSPEF**KK**TIN
LMTILDQTEYEKWQELKENFRELMNILPDNEKEGRALIHLIKNNHGDNYLEQIHGRHIETKLAKISEKENF
AKKNYYRLNKTKLQYIDPERMPIEKAKVADLFRNARDFKERFDKELGLHDFLPKVIDLDNRVIQYFIETCS
GPMMELRKEIGLKDRWGTSFMRIKDLKELKDKAQENPVIDHGWMSLFTNLVFPIDMTDYEDHYIGPGEL
QNVDLGQQRDFYSAFQRTNIFGRSDPLSEPYGTEEAEPPNFYRTSSMEDYMKALAEEEDDDEEGEGEDEN
VVTIEDPFFENPDYNPEDYEEPDWPTTPPKGANEYTRPNAFFHEGESIKEKFDEIELENFMKLLNVSPFRN
WKDHTMYHNRIGVHTPHDHAQKIDPEYHMLGEVEREEFEKMIFKKHRSGTTVRFSLAQKKPKFYNDIRY

>CUFF.25748.1 Calpain family cysteine protease containing protein
MEIGLHQEDQRILGADRRPYLDLSLIILKREDNGTLSVAGITDNETDRDLESIFTLDPGHYIAVPRTIGALLK
TPNKQFDPIKIKVSTQDGQIINQKAYSALNDLFRKIDLQLDGILTARELNLFGKIIDDKFFTNLTSESFKTKE
FEGISCTEDGITRFGMNQILSQYSDQKIQDTL**KK**MGYDECLCSKKSRVFVITFHSSENIRVRIGNAAKTSLN
DKAITLVLDDYLQQFGANKGREDRNVIVFRKYHDKAYANSYGALNKTDHTVEVNLDISSSENCAFTPTS
GTSKIIVPPKSLKYIGASIVKPDAENFKT

>CUFF.26296.1 serine hydroxymethyltransferase

MMKILGEEAQRQKKSIDLIASSNIPIPEMNEITSHLSNKSSPGFPDARHFNGDGIIDKVERLCYERALKAFNL
YPDEWKVNVQTLSGSIANLCAYHSQLKPGETILSLEARTGGGHFTHGLEDDSGHGTNLYGQVWNFKHYS
LDEEGKINYDEAQEMALKHKPKLIVCGASTYSRDIDYAR**FR**RICDSVGALMMADIAHGFGLVIAGVNKSP
FKYADMVTASTSKTMRGPRAGVIYTRSNISKTVNPAVYPGVLGAPQNSIIASLAMAFKYCSTPQYYEYAD
RVIQNCKALSDELKQLGNKVVTGGTETNIMLWDITNYGYSADIILDLGDACNLIFSGCTSPRAKAAGFVG
QDMIRFGTNVVSARDYQPEDMKQVARYIHELALIGKAINKSEDAIWHESFRIHEMAEEVENFAVKFPLPGI
TTN

>CUFF.26295.1 serine hydroxymethyltransferase

INYISLADKMLPRNSKLAGKIVKSLKGMPKSQRSTALLAFEDYYKHLEGKSFSIHKQTPHLERTQRTVKTE
DVKLHDFDPEMMKILGEEAQRQKKSIDLIASSNIPIPEMNEITSHLSNKSSPGFPDARHFNGDGIIDKVERLC
YERALKAFNLYPDEWKVNVQTLSGSIANLCAYHSQLKPGETILSLEARTGGGHFTHGLEDDSGHGTNLY
GQVWNFKHYSLDEEGKINYDEAQEMALKHKPKLIVCGASTYSRDIDYAR**FR**RICDSVGALMMADIAHGF
GLVIAGVNKSPFKYADMVTASTSKTMRGPRAGVIYTRSNISKTVNPAVYPGVLGAPQNSIIASLAMAFKY
CSTPQYYEYADRVIQNCKALSDELKQLGNKVVTGGTETNIMLWDITNYGYSADIILDLGDACNLIFSGCTS
PRAKAAGFVGQDMIRFGTNVVSARDYQPEDMKQVARYIHELALIGKAINKSEDAIWHESFRIHEMAEEVE
NFAVKFPLPGITTN

>CUFF.29472.1 26S proteasome non-ATPase regulatory subunit

MENIQQLISRSGMGAGADGGPAPDQPTPDTSEQIYISSLALLKMLKHARAGVPMEVMGLMLGEFVDDYTI
KVVDVFAMPQSGTGVSVEAVDPVFQQQMLDLLKLTGRHEMVVGWYHSHPGFGCWLSGTDVNTQQSFE
MLHERCVAVVIDPIQSVKGKVVIDAFRLINPQLAMLGIEPRQTTANLGLTNHPSIHALMRGLNRQYYSITI
NYMKMLINLQ**KN**QWSECLENEDCNEHHEENEETLKELASLAQLYHKWIQDEITKPKEELVVSTVGKVNP
KNRLAEKIEDTMTSNIKECLGTMMNTEIF

>CUFF.5755.1 UNKNOWN

MESFSAIGIDIGTQSAVVAAVKKGGIDVLLNESSNRLTPTVIAFGEHERLIGDSAVAQMRSNFKNTIQYVN
RFIGLNSECKEQIEEESKYISTKFSFTPDKKIVFSVINRGEVLELNPEQIYSAYLKKLKKLFCHEDENIDVVLS
VPSYYTSIERQAVLDACKISKINCIRLLNDNTATSLAYGFFRRQEFNDTPKNICFVDIGHGKTTCTISSFTSK
KVKIMSHCSNRNLGGRKFDQLILEILGDEFSKKYGCDPRKAPKAKLRMLDTIEKTRKILSANSEASVNVEC
LMEDNDLHRNITREELEILIQPHIEEVRRVCEQTLKDSKLKDTDIDCVELVGEATRMPIIKKVIEEVFKKDT
SYRTINSSECVARGCSLMAAMILPQYHVASFEIHEANNFDIDVSWSVSNNNMKTKTLFVKNCNFPSIKSLT
FDGRSEPMDLGVSYHDMDGIVAGLPQLLARYRVEPPTPTEEKFSLKLRIQLDQNGIPALNSAELLEEYTEI
KKIPVKSQSSASQASASSVDEEGKEGEANTGPTPPVPGAEQSYEEKEVKKTRSKEILFKFEHHGFGAKQIE
DFTNLEDDMCKFDNAVLEVKIMKNHLETYVYDMRAALDTIGNMKEFIKEDTRNAFLQELNETEQWIYD
DGESAAKNVYQDKLSHLQLIGDPVKMRFKFHEAYPSKLKEFQDILANIYQNAVDIPDESHITQEEKETLIK
QCEENTNWISTSVSVQSTLPLHEDPAIDLTELEQRKYALQDLATKTLNKPAPKQEEPKKEDEKSGEEINKD
S

>CUFF.10313.1 coatomer alpha

MSVQFHREDNLILSASLDNKVFVWDYSELKEKHQKYAGDAKSKKIEMFTGVEVEVKNICEGHEKGVNF
ACFHPNRSLIASGADDKLIKLWRMSGARAWEMDTLRGHQNNVSCVAFHPKLEILISNSEDRTMKVWDLN
RRTCIYTL**KK**EIDRFWVVAVHPTTNYFACGYDKGMTIFKIESEAFHSVRVGNQLFYVKNKVLMIEDLTN
MESLPQANLDAEGKQVLMNQPQNVYYNPFNPNSHDVLINYEGEEGYSILVCLNKNLSKNTNVVQKKIDS
AKGAVFIAKEKICVLSKSKNLFIYLFDGRNKQLEWTSKCQIQKIYQATLGKILIKSGDDILLYDIATNEIVN

KVLFANCKNIYWSSSMNYVALTSKSLIMICTKDLKPLCTCKETSKFKSGCFDAENGFIYSSSSHIKYLMFS
DKMNNLNDSKFYHGIFKAIDNPVYICGYVNKNCFYINRDGKVIREEVNSAEYDLKVALKNKRNSELISILK
KGQLSGNSIIHYLKQENCHDIALLFEKDPKVRFSLSLSSGNIQEAYKIASEMKCKDVYLQLAEQSLLQGYH
NVAEKSYQSIKAFNKLSFFYAVQGCTSKLKKMQGIAMDLNNRNDIFENTLLLGNVKDRIKLLMQSGQIVL
AYMSAKAHNLTEFIPLIEEEMKNREIKLVDNYSEQIEERCQKAKVLLPCRPVFITNEEFTTKNWPYTMLLE
SNLEDKIKHEQEEYYDAKSAPENTEQDMNDFLSSLKDQKPAELEIKPIQKKEEAKKTKEDDDEFGGQNW
EGGIDIDADILNEIGGEDIDLAELEKEIYNEQTEKENQSAASPNPLFVFAGESVIPAHHIAVGNFEKALELLK
NQIGLCNPQPLKDSFDFIFNNTKINTSTLSKYGSFNELIKDKNGENTVSLINLEYLKQVFKEGFSETTSGNFA
SALTSFQKCIQYASVSFAANSDEEEEIKKLISNCVEYIIAMRIELKRRDKNVTKTEEENLLLASLMSICRLHP

>CUFF.14318.1 Enolase
MTDHPLTPDPVKEYLDTQGLEKVLNTGINKILREFPSDALSALAVYLTSNAVKKPVFSKFECEETLIGGKY
RSFDTHVYIDFAGETKCRHTHTYTYELSDAADTDEGQNKMRENIEEAMNIIQNDVNQILNKADLSLIKKT
DLTL**KK**FLQDKRSKQEEGEENKEALDEQVGLIVVKVCSEALVHGIAKCYDSFNIYDSYAKLITGSAVEPN
NVMKLLFTVLNGGKSVNSKVKFAKIYLILGAIPGVNIVDTFLKIHKGISNSINSSKAGANGFKMGADGSYF
NACESIPESLKILADAINSSGANSEKGTIASIGVNCESESFYNAETNKYDMEGPKNLFETEQMADWYIKML
KDNPLITYIEDPFWETKGYQIFPGKLKDAGLDKHVSFGLSSFYKGNIDTL**KN**FTQFVEKEKEVFQ

>CUFF.28436.1 hypothetical protein
MGIYLSSPNTTKMVHDGETEHVIYGSTEMQGWRKSMEDACITQRLESSASGKDRYLFAVFDGHGGNQV
AEFARDHFVDELSANKNFIEENYETALIETFDKMDQLLNNPKGDEEL**KK**YTKKDSDSNVFAGYSNMDAT
SEIAFTCGCTACVVLIAGDKIYCANAGDSRSVLCKNGKEIPLSFDHKPSNPHEEERIKNAGGFVSMDRVEG
NLNLSRALGDFSYKQNKELPSTEQMVLNLPEIITETIDKDTEFLIIACDGIWDCMTNQEAVNFIKNTAQKLN
PDGCSSFKVSDITSAIFDKNLAVDTFGRGGTGKEGAGCDNMTAVIVMFKHQESTMEVDMETTGDNA

>CUFF.5039.1 calpain-type cysteine protease family isoform 5
MDEDPKIETEGEGGDQEPIDEQNEENMEGSPGDIEGSPDGDASPERYEGNEPEGENEAEDNYNKNEEDDT
NYNMNQSPDREESPEKEQEGEQEREEENNGNDEPQRDQESPVESPEGQAVYDRHQLDGQNYEEQEGED
YQYDEEGKNTGFNQEEEETKRLYDENEEEIQPIDREHEHEIDDEDPIQKKIRAIIELCEQTNGMYGDSDFPP
NDASLYFNPAEPPDYAEGTPVVEWMRPEEALKSGDSDKVAMIKNGMKPGDVKQGTLGDCWFLGSLMV
LGTHPELLQNLIVKDCIKYGFAVFQFFKNGDWKQVLVDTRIPYNRETKTPLYGHCADPTEVWVPLMEKA
YAKLHGCYQALNGGSMSEALVDLTGGVSEKILLESPETIEIRDNGVLWKELVKYYQQGYLLGCSKKVAD
EDGNPEEGMGNQGIQYNHAYGILDIRLALGIQLIRIRNPWGYAEWTGRYADEDEAWDDSKGLKEELNYA
FEDDGTWWMRFDDWYMNYNQVYVCKIFPAAWQQFSIQSKWEGNAAGGPYPPMIDRDETTGVHSQLDT
NDKWFNNPQFRLSVTKKTQVYISLMQEDEKISKKPYIPVNFLVVRVYSKRQRLWEVDKDDVVVEAAKG
AQRFGQREVCVTCWLQPIYEKKPVHYIIVPNTEVDSKKDEERPFVLRIFTSERVDLVELPKTIEQTFVGKW
VQSTAGGRRILQNGGDNKHWCRNPQYFLNLKKPTHLKIILRKKGG**KK**IKGVTIGLCVTKAFPPTTRPAAK
IKKDGQEIKSSKGAATANMTKTGKSSKKGFNAAAITYKRNIKEDNFPEIVPPELGKLERKLQILPNEWYIES
QYKNEDVAAIYQFWKPTNGPFLIVPSLQVEGYTSDYTLTIFSSSPVEVEPLEDSKNMVISGEWNDKTAGGC
HLYDRAFETIPDNFTWINNPKFKIHLFTTQKTHVKITLSRPEKAWKKQIAVSAVDCMIGFYVYPCNVTPTK
ENCMSKMSFVPMNEYSETIELDGNPEGYLIMPTTYKPKCKGPFVISVSTDVEFSLDMY

>CUFF.6691.1 long-chain-fatty-acid--ligase 5

MDTHKYIYGKFKEGTGVDGHSEELYSIWSTEHDYQFQTIAKGGFTTLLEGWRQSVDSFPENNCLGYFDG
EKYVWRTYKQAHHEAQSLAKALYSRKLVTEVNCKSEYGKTLNLKLMGLYS**KN**RPEWALVNWAIMHFS
GTVVTLYNTLGEESLNYAFDHTEMTICSCDQASLTKLLKLKQEGSIKTLKTVVAFDNYSEEEEKQFSELD
VNIFSYKDLIKEGSSLDESILEEMQKPEPDTIEVICFTSGTTGTPKGAMISHLNYTCNIAACETTGIDFNEND
VVLSYLPLAHCYEKWIHSICLSRGSAIGYFRGDPLKLVADMQILKPTMMACVPRILTRLHDAINMMMAKE
DWKHKLYKIALNQKLRNVSQVKFTHLLWDSILFKYTKALIGGRMRKMITGSAPITPELLNTMKCLFCIPIV
EGFGQTETNAPASITSHLDPYAGHVGGPLSCCIFKTEDIPEMGYSSTDKVCL

>CUFF.7264.1 SpoOJ/ParA/ParB/repB family protein
MVKIRKFCGYLANKDNLEKIISPPYDVCTVKEGREWTGDNEMYYFHIEKPAIDCSDDATDEEIALKGKEN
LLSFIEKGYLIKDDAERIYIYSQQMGDHVQYGIVCLSSIDDYENDII**KK**HEHTIPETVLERTNLCDTQCANA
SPVFFSFRGGEDIQKHISDMTQAEPYGRCTTHDGVIHTLWRLNEDDSDAIVKSFKNIESTYIADGHHRSAA
AYNVGLRRRQREIDQGINVTGDEDFNFFLTVIFPDSHCMILDYNRLLKSLNELSEEEFLEKLSKNFTIEEIKD
DHHKPKEKGHICLYLKSKWYDIQVKPELLSSDEKAKNLDYQILTDYCFKDIIGIENIKKDKRVEFVGGGRG
VDYLVKRCHEDCKAAFVMYPVSMEELFSIADAGEIMPPKSTWFEPKLGSGLVVNVFEDALGIKN

>CUFF.8557.1 tyrosine aminotransferase
MEDSKWNKKRKASRRSDVLNMIRNIIEKEFKIPENPPVKVLNLTLGVPSNDNGYPMSVETKQAIIESVESE
KFNGYTPSNGLLSARQAVVDKYSTEEATFKADDVFLTFGCSGAQYAVFTTMCEPGDNVLVPKPGFPLCY
PIAQNLDVELRYYELDPENKFDIKLDTLESLIDERTRFILVVNPSNPCGSVFSKDHMKDIIAISEKHQVPIVA
DEIYYGFSFDEERPFYSFPHIDTKIPIITLGAISKTYSVPGWRLGWLIVYNRYNYFDDIIDKL**KK**FFMINLHPS
SIVQSALVKMLRDVPQSYFDETKAKLKQAADFSFDSLSGIRGIKPIKAIGAMYMMVLIDTDEFEDIIDDVD
FCKKLLSEQYCFVLPGKCFFTKNMFRIVICHPEDTFEELSIRIKAFCESHYKNKDV

>CUFF.8736.1 classes i and ii family protein
MYTNLPKRIDDNSCLFAEFGQLARKFNAVDLALGTPELDPPQFLKDALVEAIEKAPQQYIDPRGYGPLRE
AIAKEYSLIYNNLGRDLNPETEIIATAGAVAGLYCTLMSIISKGDEIVTFEPCWTGVLNMIKLSEGVLKTVP
MKMINSVETQTITWEYDWEKFEDALTDKTKAVMLINPHSPTGRVFSNKDLEDLTHILDTKAPNAYVISDD
VYDFCKFQNEESHFAGYMHN**FN**RTITCYNGGKRFACTGWKVGWVIGPSELIKSIALMHETVVWNFNTLG
QYAFSKCISGEADKPYEGFDTYLHYIRHTYQQVNKTVSEAFIQSGLPVHPCLVEGGFSMTLEISDCESCIPS
KYFEDDYIDDPKILKKNFSDSKVPLDFAFCRWMACEVGIAMIPGSSLYKDETTAKHDFVRISICKKPDIAE
RITEKLQQYFEK

>CUFF.8783.1 Cyclic nucleotide-binding domain containing protein
MESDKKDKSIKEKMNRAYLDSKINRIIEPMVVELMKKTPDDPITFMINWLKDNHGNRASIHANERFELEH
LRKEVPKLKERIAQLDNGEGDDEELGSEQESAGSDEGDYVDELP**KK**AEAKANKQRTSVSAEAFGLYHKK
SDFKPIVIEKSDAVKNKIRKRLAESFLFNTLNEKDQNIVLDAMKEENFKKDDVVIREGDDGDVLYVVESG
LYNCSKIFPGNKEPTNLTKYEAGAAFGELALLYNAPRAATITCLEEGTLYSLDRNTFNHIVKDAAAKRRE
KYE*DFLSQVKILE*SLEPYERSKLADAFREEIHKPGDYVIREDEQGDVFYFISEGEAIATKTLEAGKAPVEVM
QYKRGDYFGERALIKNEPRAANVIAKSDLTTVSLDRHSFKRLLGPIEEILKRNIASYK

>CUFF.9912.1 peptidyl-prolyl cis-trans isomerase-like 6-like
MEHESVKHHIFVAGIINDQDFQQAKTCALYLERNHPEDVRATCFQLFETQWEEMLKEIKATKKGKFHYH
KGNYIVFVNDDTYLGNYEGLEQWALMNYRYLDNSNLVIYKRKAILEF**KK**AINDTKGRQYCFMEIRLGED

LPQRVIFELFTDIAPDSCENFRKLCAGSFTNKKGEKLTYKNTEFHRIVKGAFVQGGDLSKLGISKFGQSIFD
GYFPDETFEVKHKTVGLLGYCKKSGLKYTNECQFYVTTGAPLSFMDGKYVVFGRVIQGMRAFRQMEKM
DCLNERPKDKIEITECGEY

>CUFF.17268.1 Isovaleryl-CoA dehydrogenase
MMFKRALFNKKSNYISQFTKRSITKYDSVIAMNEVQESMKEMARNFSQEHIAPQADQIDKDDFFDRSIWR
KMGSQGFLGITVPEEYGGAGLGYFEHCLVAEEISRASASVGVSYIAHSNLCINQIKLNGSEEQKHKYLPKL
ISGKHIGALAMSEPGAGSDVTAMNLTA**KK**VGDKYILNGTKMWITNGPCADIIFLYAKTDSKANKHGISAF
IFETETEGFTVTQRLDKFGMRGSETGELVFENVEIPAENLVKQEGQGVYILMSGLDFERLILSAGPVGIMQ
NCMDITMPYVVDRKQFGKSIGEFQIMQAKIADMYSALQASRSYLYSCARVADSGSRSPKDFASLFHFVSS
NCVKVALECMQSYGGNGYINDYSCGRLLRDAKFYEIAGGTNEIRQWLIGRELMKDYSK

>CUFF.21695.1 DNA topoisomerase 3-beta-1
MEKKESNENGRQVDDWEENKQDREDYGSDKGENKKEEERSKPLTSFHDKFLNRDDYTEEEINEYKKSLV
HDEREVKKRMEGIRRRKGRLNVLMIAEKPKIAQLISEILSRNRYQTEEWNGFKTYKFDNLKFKEFDSNFN
VSSVYGNIYGVDYDEDLNHWETNPLALLSKKSIYTYPVHRDTRDRKMKKNSNVNLEYLETHLMYLLKD
ADVIVFWMDCDKVGENICFQVIDLLKDSFPNFNLENIFRAKFHSLKHQEIVESFENLNMKPDERKSLGVD
ARLAIDLRVGISFSVQNTSKLARYISKNKNSVDLITYGPCQFPTFWFCYERLQEIKNFKLENFWTPVLFIEV
DNQRVELNYKEEKLTSLSEAKEVIKEIINKNSLKVVKVTEQQHKLYKPKPMNTNDLIRMASNCLGFPAVKT
KKIAENLYSRGYISYPRTQGRKYLSEKEIKTTLEEYSNNKYYGCFAAQVRKNFDYDFDDLYEGEKDHSPIT
PKANLIRMSILKEDSDEFKIFDLVMMNFFASLTKNGKYKSVTTYFEVNGKIFKETHLQMIENGFTDFIGPDA
LSLEVMTFKDYSFKTNETYKIKERFVKEWQTAPKQIMSESEL**IK**KMELGRIGTDGTIPILIKKIINRGYVEV
NDIKGIRRLAPTSMGEILAEGYLEIDKGLITPEVRVFIEKCCNKISNYKKNKDSVINKMIHIFNEKLKNLIKN
FSVIEKKFKNLHKTIK

>CUFF.24110.1 Asparagine rich protein, putative
MEAPGSPKIDFDRLVIPDFSEIESEQFKKEVVIPYFKDIYKDLASRSDKKSAGINKVTIIDYCQLPGILAERFF
SLLDANNDEYIDLREFVYILFKIYYSNFDNQVKLVFDIYDFDKDGYITKEDVRIILSYIPILKDKSTKGDKEG
IYNQEGGGVEDFSMRIKIQEEISQLLDLTFKKKEKINLEEFQEINENVTSDMLVTVLSLLKDKLPCSETFYT
LQKEFEEKSTKLKKDDGSKGEI**KK**QKTIASPRILKTLSPLARMATGGETEENKEAHSYLRKLARGEPAVA
ADSKGIEEKDIQLDKLKKNKKRQNAEISTKITEDPESPLVSSDVVRLANKNPEPKSLLLKNKPKNIFASPTT
FLGGSGVEREIEELTEDSKSAVVFEGEMMRKAKENKLKKYWYKLFDKELRVYKHKDDENHKTMVNLIS
VFLRVDPEEPLDKKNTLYPFSLIFPNKERMFYLLSSEERDKWMEQIKKAIGYASLFDFYEVKDAIGKGKFG
TVKLGIHKKTGKKVAIKVMKKKQMTLQDIELQKREIEILKICQHPSIIKLLDVFENQEYIYIVMEYLKGGDL
FNYLEKRDFTISEAKARDLTHSIATGLYYLHSFGIAHRDLKPENILMTDDTDDAEPKLVDFGLSKIIGPSEK
CNDPFGTLSYVAPEVLLQKPYDKSVDLWSVGVIIYLLLSGTLPFDDDDDREIARQTIHDEVDFSYHVWKK
VGADVKQMIKKLLDKDKDKRMTLEEVLQHPWILKKSKTLLEARKKSGDVNSAEQFKAFAMTEETMKGS
EDS

>CUFF.24315.1 nucleolar gtp-binding protein 1
MSTGTMSRYNIKGIAPIPDAKKLIDVVLSKTNRKTPTEIRANFKIHRIRRFYMRKVKFTQSTTNEKLQGIID
GFPKLDDIHPFYADLMNVLYDRDHYKLALGHVNKAKAIVDKIANDYVKFLKYGDSLYRCKMLKRAALG
RIATIIKQLGPSLGYLEEVRKHLARLPSIDTNTRTLLITGYPNVGKSSFLNNVTNADVDVQDYPFTTQSLFV
GHTEYKYTDWQVIDTPGLLDRPISERNTIEMQAITALAHLNACILYFIDISEQCGYSIAQQIKLFQNIRKLFF

TVDPESGESVMSKPIVIVLTKIDATPYESLKQEEKDLITSTAESSKALIVKMSNIDGTGIDDVKKKACDILLD
HRLTLKAKNPKKAESILNRIYVATPKKKDNKSRPPCIPASVVAEHQRPES**KK**TILDSQIEGGGAGVWSYPY
PEDYILEDPDWKYDNRPVFMGGHNVADFYDPDIEEKLNELEKEEEYLRAIEEAEAPEIKTDLEERMWKSL
DEIKKKIDYKRGIHELNRDKKRVIKKSREADDMMEGLAEKIGEEKANEIVDKSTKNRKTLFEVMGVSRKR
RKLDDDMEVEGDENKDQEQPMEVDRDNMDMRKKMRDRKIKFALSRMEGADPKKLSIKSQENKSMERA
RHKIEKQLKTTDKIHFSDRTQITKLPKHLFAGKRSNGKTDRR

>CUFF.25027.1 Kinesin-like protein
MEKEYVRVVIRSRPTARFASKNIKIDEENNTITITKPKNEDAGVVNNQVESWKFKYNKILHNRSQEDVFDF
CARDIIANIVEGYNGTIMCYGQTGAGKTFTMNGSTANYKYRGVIPRTLSLIFQEVDSRFDQQVTIKVSYVE
IYNELMFDLLSPTPTHEQSGNISIVEDAAGNITLKGLSLHACKNEEEALNYLFEGETNRTVSVHELNKNSSR
SHCIFTIHTESKSKIESTEKIHVSKLHLVDLAGSERTKKTGSSGITLKEAAFINKSLSFLEQVVVAVCDRKRD
HVPYRQSKLTNLLKDSIGGNCKTLMIANIWPELENLEETISTCKFATRMMRVSNEATVNINQDPVVLIKKY
EREIRDLKRELVMHNTLRNKGRITYEADSPEVLYDMARKFLTGQIDEIEQLDSILQCRDLMHQMRLVYRK
LEQNARIGSSKHGDGNGGADDGLDIRGDPAELERRKTLMEEGRVGDEDEGMNGFGIGRAPKESKP**KN**QI
LMKKGDEKDKQDDDSESKAEPDADATGEEEKVSKRDKKKKKAGKNKQDAFKEYKDEEGKNIEESIKQN
RTDLRAKKKEMEQLKEICNKAKKEIDTLKEKLDSKNDEKQKSLELDEEEDIIDEEEFNMLKQLKDYKKLY
RENFDKFKQLKGDSFYIQNCIDQLKEQLIFKFEVWYDENFDQPHDSKEVQRIMSHDFSEE

>CUFF.25040.1 hypothetical protein OXYTRI_05506
MGCSADKPKKSDAPLRKRSTLNKNNVGSQPGRAPLAPPMSINENNKSNADGVISAKAKIAKTEFQTEARK
PKQAEAASEQKDLIANAIVNAYNAKSDSPSYKMDKIEDIAASFKDLKDEKIVGEMPQSVLLFGKHKYLTR
HWTPLLFAIHNKLIKVVRYLVEYKQVNRRLATINREIQDDMNQSQVLPLLIAISNEDSIMFEYLWSMTEL
WGIEHLKQILSSIFSHTTWSKGVEILLDSEATQDIYNGLSYEEKKQFMIEMNYRYMLQASGDIKKIIKKMM
HDSPYGIVTLHFLMAEENEANTPLIKKCLDLPFKDYAKMKLEANKEFINAWNATLSRFQSLGGGFAKTSS
SVTNQISAWEQEMANSQYFKDQKTYKGYLDELDKACEDGDFEKVKQLVLALDLRHLRRIETPLSVLIPD
VITAETYINCIMIAILGGHLDIVRFFLEDCRIDVHDAVKLSNTRDSTSRAKSVENEAFCLFLCISEGHQDIFN
YIYLNHSYIFDKEHLKACIKLCEALDREKFIDTLINSKTSHEIFNSQELDGKIEMINFLLSDDRKY**KK**QYIAS
LKKSPYHWAFCVSKANNVHDLSDKEVKEVAEVANKIQDHEIEVQDIEKRSEKIDRVDSFIKKLSKLDEDE
ERFEAFRSVTEKLVNTHYPQYKAEDDDKLLTEISEENVKEENENEDSDDSSDDGSFPSGEVFNRYAEEGR
LHKVMNAVHHEEFISLFRLDRYETEITIEDNQHDTELWNALLFAIHKERMDVIKFLMEANLNTRLALTNP
EKRVDEYDDEDLIEITPDDELYGVKVAIGTRNFKVFKYVWDNNRDVWTEEHFLDLIDYLEEVEWDEGLK
CLLESHASHEIYISCTLDNKIFDKLLQIALKDRKDNKKKPGDLMSILTKSPYCINMAITESKDLQDEIAEAK
TNIQDNELNILILRNEVDKYETLIKKSGDQKFVQSLRQYQAFQEAHFRVQIDEAIKKVIAKKDNDIQLFVD
NPKLAHCNLHTHLKFDLQKYPKYIHNINWTFPSLLLLCENIECFDTVVAKYRPMLGMIFRDEEQIEGEDDI
QRSLLQLLCELSQDIVMFNCLENHPSMFTFKTLYNLVKHVISNGKPQHARILNSKAVKSYFTFLSPESKRK
FIEELIDWAEDDKMKEVVGVILCNPTYEEFFIFANEDLHAEMKGKSIDLFNSSKGGSKHEHSSGEASEADD
SSEDDDSNDEDQSEKEDEDEDENSEDEDENSEDNEENSNEESD

>CUFF.25365.1 actin
IDDDNDSVIIDNGSGKIKAGFADYDSPKSVFPTVIGVPKAPGILVGMDQKDFYVGKEALSKKAFLNLTWPI
KQGRIEDLDQMKNVWEYLMNDELKFAPEDHKIFMTEPPKNP**KK**NRSELTRLMFEQFNVNRFYLQKQAV
LSLYASGRTTGTVVDCGYDISHTVPIYEGFALPHAISEFSIAGKELTEYLKQQLVQKGFDYASSSNNMDVV
NEIKETKCYVAQDFDAEHKNALDHDTNHVVYNLPKGSITLTLERIQTPELLFQPSKNDKGIDGIHKSTHDS

IMKCDNDIKRDLFRGIVLAGGSTMFEGMRERMKKEIQALAPSPMGPDVEAPADRKFSCWLGGAILSKIKA
FNDIWITKAEFDEDSENIVHRKCF

>CUFF.26432.1 hypothetical protein OXYTRI_01330
MSKKKIFSTINAISFNHDGSMVALCAGTEEIFVYITNGSQDPSKWEKKYKLKEHTQKATVIDWNPVTNLIL
SGSYDRSIFVWKYNEEHDKFLPIMVNMDEKLSILDLKWNKQGTKFVVGTSSKRLYLGYYNEKNNWWST
AEI**KK**QHKSSILSVEFSPNGKVIASASFDGTCKIISAFLEGIDTAEESKSSFSDVTTATECLIEYKCQFWLNH
VTWSPSGDLICYCSHDGTLNFCDVTGGTKGKIFKYTHKGLPFTKGKFIDENTYLAVGYDKAPFLFKRSDE
EWALSKVLDEGYEKFRIYSVKKGDANYFKIKEIESDIMIPEKIKMKERDTQHENTIQQLVSFKPESGNKEIC
TCDENGRIIFWKL

>CUFF.27467.1 wd repeat-containing protein 52
MSDNADQPKTEEPPAEKQETGSSHPPAQEENKEETKDTPSAEKKEEVKEDEHQEKKETKEVKETKDAPTE
KKEGDEQKPVSTQNNEAVKESQPKKNESEETKEAPKEDKTEKEKKEAPPVEQKKPAPVKKAEKEPKKKV
KKVINGEEVEVEEEEEEEEEEILMGENEFYPENYVDKELPDSTIPKKYSKYHGCYGVESLKRFNIHLIEPYV
LIYANGNIYELYDMKEKSRKILFGKDTDGIGSICVHPSRKYFAVAEKGTLPNINIYDYPDLKLYRICKNGTE
KAYSHIEFSTTGEKLVSVGSAPDYTITVWNWKAQKVVLKSKAFAQEVYRVSFSPYTDGVLVTSGMGHIK
FWKMANTFTGLKLQGELGKFGKLELSDVTGFRELPDGKVVSGTEYGTMILWEGRNIKAHLVRNVDTKEP
LHKGFLEIVLLYKEEFITAGQDGYIRFWKFQDIDTAEFSDELMEVAIIPTREIYVGHEDGTPAHINQMLKEG
DRWILGDARGRIWEINLDTEDKFEVMNFHSGPIADLGVCPKKNVAVTLGSDGIVKCWDFINEKELFSRMF
PGNGTCLDWLPYTDQNKGRIFACGYSTGVARVFSIESDRFKLLNSVKSLDCPMVKIKYSPDGYLLATVGK
DGSIFFFETTHDKYDWLDPICMCKIESEINDCTWDSNSKKFMVCCKNGRVYEINKPNKNEVNVKESFMVE
LKYRYWQIKMMEFQMKKNQKKDEAELEKIKRMRLRGELPMEEEDEDEDWDPEPINTCRYTNDGSGRFI
VTSCGLFLGYYYICAFENERPLKAIDMPKSGICTYFDYTYSQDFLIAGMSNGSYIIWSTESEKKFLEIKMHD
GHRGAIRSVKFNCKENYVLSVGKDGLLNVQNINREAVKMFAQTLMPGEDVTFINEMDGFETLETEQILK
AREDESDDISDPEALSIQEQKIRTEEYWRMKKAEERKANIRNKVTLLREEFEKLTNINLAEDKWIRLQDEE
FNIDPEFFQMLERELDNKSEITRKEVAWGIEYRRVSLAKLMNVFFDCLHFRKFTVKSIKNSSFVSSFRVKM
NPVLEENVRRFKEMIEKEYHPQSGSDDDIQSIGSSQRDEMNVEQKSPTKLQQTTHQFQNQN**KK**KNKTEA
ELKREARTKAREERKRELEKHEKEEAKWNQDNPEDIAKVEQARTSFNDYKLKMSVDYIVPEKERVNAEK
KRQQMVLLENSIFNLKVDFNKKLEELKSVRKTSIIEMVKKGNKRLSEINGELGVEEELFYPQIDEAVEYPE
NFYKVNMEDIIKYNHKKAKESKVVSKASMYGSKKDEGAKSLEEKQAEEFEKVYREEQKKNEVIVEEVTT
LIAPDRKSRQRTHDTEVDLEMKKIRQIELEYEKDQIKTEINEAIESFDIEISELQKEKYRLESDLKQAEMKLI
TYCEELIILNGMEYRDKELTKSLAECRKDKGKILKDINEISKELKGKKREIEDIREKEEDLMKRFHSFCPEG
SPMYEDILNFFKKILVNRRKGDRGDGGDGDEDDDMDEDVDDEEDDEANEASYKFNQEEHKIDDIEKLRE
ERNALYGDKQKIESAISDLEKNRKKLEISESSIKQRLEETEEMIQDFQKEKMDKLNVLTVPIVLKISQMQNL
EKNDEEYKKWEKFREEQINPGGDDQNFDNQHQVEDDWRGYFIPKSLKDSTLFTREGLLRLIERKRELDIQ
YAENNETLKAKNEEKKAIKTQIKANKKMKKQKLEEYAEKQMLRFGDLVDLDSLEVSGPSQVVIDLRNN
QLRVEKDCMRKREEAEAELEGTQRELTKAVKENTGLLELIIELGKEQIDCNEKLDQTKQAILEDENADQK
KQILREKQELREILRAQAENIEKLKTEINLYKRKGGHIYTKVTTNRRNANLNQNE

>CUFF.28399.1 coiled-coil domain containing 108
MSRKEKHSKAAPDVKDILESVPSEFDFGYCSVKSNTTRSFTITNVTAGIVKFLIRKHENCPFVLSHESGVLA
VKGKQEIGISYLPQECESIASSIILSINGEEKTIKLSSVSKYPLLSVSETSLDFKELLVGKSDSKDITIQNTGLV
PALFKIEKELSEEDDSSLSVSIKKGQIQPGESVTVSFKFQPKLVGVCSNAHYTVRSKGGNFISVSCYGVGIG

YDVHLSAKSMNFGEVSKGNSTNRIVNVVNDSDLATSFQFLVDHRNVFSYSITEGVVAARGSIRVIITFTPL
DTVCYYERIFCLIRNHKLLYLDLMGTCYDEDVKPLPLMQRHVDGFRDKVIMGKHYTMKEIKDDDDVSN
HMESNLEEDTKFQFEIPEDDPNHVMHKEMFLENDSNYRDIALNMQFIDFGYCEYGKSSEAHKIIIENRMPY
KVEVKWTILDVLNSQGEKTDNPYKVDESVMIIDAKSSANFIIKFRPFEPDYYFFQILQCFVYLLNGNERKM
RKQEVGGNPQSVMMTKGKITRTSKSSKFEESMYEEIDPPLCLNLRCIGHSFNPGSQPFIPIVKFLPMKNVVF
PPCGPGESKYQTLKIMNTSDTPVYYKMIQDPSKVFRIYPLLGLIPGKSFALVCFEFSPKSANHWSFTSQCVL
NYMFTNVQNIHLSGKCFRPQITLGNKGKLFFPPTYTGVSTEQKLMIKNETRIPLEYKCLVPVKHQEIIMFDP
PKGVLQANEERMVTCIFTPLLKKEYSLSIVMQVTNIIDTVKELIGYFNPGSGVAIKAEPKREQTKYELRVFG
VGNDGILSIKPSKLDYDTVTVGFSKILSLVVVNKSKTNLYIDFQLEQMNIENKSQEEQDNIRNILCENFNLD
FKEGIVPALSKKRVKITFRPSLRFDYNIRFTCNAREKPVQDLMASIKQNSYLSQKYSINIVAKGDYPLLRFA
DIRNDQMSVSNLWEKFQLTSLNKELLTELRTEEIEYSNSEKTNQSVQDLQ**KN**LKIFNWDFGKVPIKYNHK
PRKVTLTMKNVGGVETEWIFKLPNDSEVELEPWADPGEPTPEQAFEQHIIDNSIFMIEPRTGSLKPGEQTD
VNVYYVSKEVEFHHLKVILHISHGKPIILHFKGETLSRRAHLRLCKDEFHIPPVPVGLEWSVTYPIEIKNLGI
TKLKYKVDLDNLEKLNNENHSFRVFDIENPEGVLMPNEVQYLYTTFRPLESKQYFVNLPIKVSDIEGIVQN
IPLKLNGVGYQGETNKPNEVQFYEDLPKCRAHCRNEDELQAAFSIEELDFGEVDPEVPSIRMVILYNMSPT
QKLTFEFFMTGLLCTDNINLMPISGEVEPNSH**KN**IKLVLKAAVIPTIFDGEIMCQIEWEDQGNKGNAQTINE
THTGTKSVVTHASDNEFLFIRLK

>CUFF.28683.1 hypothetical protein OXYTRI_11093
MKGNLKIMQCSNKAVEVRMKNEANPNADFQIIMTLAINIKGIDCYDNFTLFWDGKYAQIYEVFGDKNPT
LIGTFETNSKIMALHEDSVFQSKENKIEVLNYQGEVKQIIPLTEQDGEIILIEVVNKNMSVITSNNMIRVFDIS
RRNIKQLGMTRKFGEKNQTSIKGVALNSDGKRLAIIADQNVMPSVKVPDIKFYIYDVEMDTFMNYELEND
RVPLEAFWDQQDSRLLCIETEYMKMSEDNEEGDEGDEEGESKKKVEPFTGKTVEKYFVTGDYGIKKQDG
INFEEGEETLLGSNVPYLFFLGHKKSKKEDEDEIIEEENKGGSFVILRKTMKDFASLESVDEETKKAILNFSF
FLTCGNLDDAYNSVRTIQNISVWQVMAQMCVKNKRLDVAQVCLGNMRFARGTKVAREAEDEKEIEARL
GLLSIQLNMIDEAKELFIQCGRYDLLCNMLTASGEWNEAIEIAEKNNRINL**KN**IYYQMAQHFEQSKDYES
AIENYVKSNTHAREVPRMLYKNGMFDRLEQFIEERQEPDLFKWWAQYNESKNNIEEALNYYQRAEDNA
SLVRLFILNKDLNSAMQTAMETQDLAACFYLARSLEEMGNLKEAIQYYSKAQRFHHAVRLAQEHHFDN
DVFQIAQSSNV**KK**IMLQCAEYFERRNRLEQAVILFDKGGNTKRAMNIAIKNKMPDLMKSLSINDTENIKD
PEDIEKSAKFFEDNQQYDKVVELKINLGKYEEALDIAEKYNVELNDKMVEKLIPEKGKDPMREQIRLGML
TRIAKLVQKNGNFQLACKLFTQAKQKVKAMQCLLQSNNTQKIMQYAQIAKNPEVYVLAANFLQTSDWH
NDPNVMKTIIQFYSKAEAFDKLSGFYDACAQVEIDEYRDYSKALGAMREALKQLNKADIVGKDDKVDFL
KRRIGIIEDFVESRNLIKTDPSSMQKLCNQLLENPDVESAIRVGDVFAQLIEHNYEKGNLEAAFTYLQAMK
KRKIIITPYLDQEMVDHINNANGVKSAKRVEEDEIPEDF

>CUFF.3641.1 hypothetical protein OXYTRI_10687
MKANSHIKQEDKNVESEQTPKYELAINRFENVELYEGTDLIRLGFEDEQSRIKVFEKECPSYNHIMVYDIK
TSGYEDHEDYLYSANPDLQYNLRKGHTIIEVYDKDDFNI**KK**FTMVARKGQQKYYSIRAEDLEEDKEYFM
KQDIENIKDQNEYEEVVKNQGLRNYTLGPVFNSFANDGTVEVYKSTYVIGEHAQISWIDSEGVWVFASRH
ASIFAKELEDLKAYNSTPTSVSYIPCMIAKFWFKYFDENINDKIGFQKALNGKTLCASYIDNSLFDSLIKYE
APTLIFNSIVENNSSKINCLTPGVSYRFFTKWGLIASPINKVGTYTDYISLINDVKLIHNKISNSSLHDDELGA
VIYFVEKGEPDRVISMGKIETKQSIAFLSLAHMLKEFWKVNESFTSWNKALNSQYKSSFDIHISILKSLNIK
DYDFYYNISKSAYDRLKEDLDLYHKLKTNLIGFFLNIIGKSRETKEMFVSTTLSKESDDISTQSLVGSRKNS
SQFDEIHFPRSEDAIDRLMKNEHIRRQSNINTDEEERLEDIICSEASKSTPNKAKQIKKKKTKTKKPNNEKE

DIKEVEIKENVPEEVEISEEIEKSCTIKTPEVKIYNAKIIARESALTSKDKKLRNLLMNKSIIVEAGFDKESWV
LDCEESDVSTQNIYNAFVVEGDDIHNHVHLLSHMLKLGERSRRIYRSYNPFGLKKKRCSFKIKNIKSNETI
NLTGIYYDQYIEKDLLTQTFSE

>CUFF.606.1 UNKNOWN
MNPKSSSKKDKKVKGTSGKNKTAKEAFIKKIDVCMKIYDYKDETKDVKGKNERLVAINELQNLLQDQ**K**
SVVQLIVPNLDHCIHMIEKNIFRCLPNVKKSNLAFSETGIEQEEETDPAWPHIQGIYEFFLQLVINEAVEVK
MLKSFITPEFVSEFLELFDSEEAVERDYLKNILHKLYAKLVPRRKMIRKAVNETFYQLIHEGHKFNGASEL
LDILASIISGFAVPLRDEHVIFFNNVIIPLHKVQTCSEFFEQLLRCSMLFLTKDKALAVSLLKGLLKYWPFAN
CVKETLFLTELQEVLEIVEDDKIQELIVPLFRRIVRCIGGTHLQVADRAMCFFENEYFLSKLKTYRHLTFPM
LVPVIVELSEKHWHKILQESLVALKTILSEIDPIAFEDALKISKNDERRFIVKPNKDKRDKLDQKWNKVNST
LKSSNPSYEAPLIPFRTNKLVMDFNPLYKKIYDKERYMNE

>CUFF.7193.1 UNKNOWN
MDNNLIPDRVIADFEPPPAAPLSDDLLFPEKASGKTIPDWKALRDHLNKEGRVSKESCHQILNDTLSMLKK
EPNLLKLKDPVTVVGDIHGQYYDFVKMIDVGGDPENTKYLFLGDYVDRGSFSVEVVLLVYSLKLNYPKT
VFLLRGNHECRQMTAFFNFRIEVLTKYDEETYNLFMDTFDALPIGCIVNNKFLAIHGGISPDLKTLEDLNNI
KRVKEPPRTGLFCDILWSDPVEDDNGYCESIYKNNEVRGCSFFFGSEAASKFLK**KN**KLLSVIRAHEAQLEG
YKMHKWNAKSGFPVVITIFSAPNYCDVYNNKGAIIKFKGAEINIQQFNYTPHPYILPNFMDVFSWSMPFVI
EKVTEMLYNVLKYEGCESDEDETKELSSESVKDKLSEEAIASHKLRINAMKNKIRSIGKMASIFKTLRQEH
ESITKLKGLCPGYKIPSGTLTSGSEAINEAIVQFEKAKKIDRMNEGMPEESKLV

>CUFF.7325.1 RNA-binding protein 12 isoform X6
MGNPLCRRQQNTFDNPIFEFAHINNDLDKAVDLALGTCRMYNRVLLKFSCDKLPNMDYFSLTDAFLVLF
ELDNKNWTEVGRTEIVEDNLNPVFIKGIEVKYFFEQDQKFKVVAYDADSFESKTLSIKDANYIGEAEFTIQ
SLVAKRDRSITTDFYQNKEKRGTVTINYEETNSEYNQAIQIVFGTEGGEFVDGSEYFFVICRLSNTTPIKTTP
VMRSEVVRYSF**KK**EENSTNCWKRIRIPISSLATTIDPNHSEGYDPGNIRFQFQLYLYKTNGSHSLLATHDST
ILKILEEESVFECKGVKGEQYQIERVSAMIETVPSFLDYINAGMNINLTIGVDFTGSNGDPQYPESLHYIGD
KTKNQYLNAITEVGKILLNFDTDGDVPIFGFGASIPKYFKEVSHCFAMNGNFFRPEVNGIEGISECYKNTLS
KITFSGPTYFSPLL**KK**WNEMVTFESTNFLSKYYVYLILTDGAIHDIDDTIDCVVESSYLPVSIIIIGIGDANFS
TMNFLDADDEPLYSHKLQKFQERDNVQFVEFNKFKNNPQMLARETLQEMPRQILQYFQKRKIFPNKQAG
GADFNFVNYETRDYFASKGVEYMQNCAGAMHMSEEFIMGLVNEGIPDYETLNINSVKKGYQNPLK

>CUFF.8158.1 UNKNOWN
MYDASSDRPQSFKSAILLEILENGLKLDIYDKKYFILYLEHPLKKWHMNKSKVIKEYQDYAWNSYIQNIH
QREGGIMNSNLEAKMYKRYLEQFYREKGDLKEFEEYFSPKFLSALVDEFLFLSGKELTNDKVDFEKYDRL
ASLVIIELLSSNKDFFKPKERVKIHTELKNVSTLHLKIFEINSLNYYKKNCAPFKSDVNLDGFITSYEKEFKF
DKISPH**KK**FRHTFDFPELDNKLGLFVIEFISNGYSSRAIIKKGTLSVIYKQTISGQVAYILDDEREICSSEDTG
VYFKNQFFKADSDKGGKITIPYEKQLTTGNAILVHNGFAQLVEFMRFPEKYTLDVAYITSSESFIMGNQAK
IILRPCLKVNNRKCNLECLSKTKITIVTTSFIDQVPISKIFEDILLKTEKDVELK

>CUFF.21916.1 asparaginyl-tRNA synthetase, putative
MYSYGQRVRIGTFFSEFGSQFIGKEVKVCGWAKELRKAGKGAFYFIELSDGSSIDHLQVVVDKGIEGFEGL
KGEGVGTSFIFVGTLTESPKCEQKYELKVYDSTIHSMKICGSCPQKAYPLP**KK**KHTKEYLREIMHLRPRTT

LIGVIARIRSSLAVATHEFFQKRGFLYVHTPIITASDCEGAGEMFQVTTLLSEKGTKESSYIKTKADGKIDYS
EDFFKKPAFLTVSGQLDVESYCCSLGDVYTFSPTFRAEDSHTTRHLAEFWMVEPELAFADLKDDMDCAE
EYIKYCITYILKMHKDDLEWLEKSETKEIISPLEAIVREDFERVTYTDAIKLLQKEESAKFIKYPEWGIDLQT
EHERFLAEKIFKKPVFVFDYPKDIKAFYMRMNDDGKTVAAMDCLAPMIGEIVGGSQREERIDYLEQRIKE
LGLPVESYEWYLELRKYGTIPHSGFGLGFERLLLLITGLDNIRETIPFPRWPGHAEF

>CUFF.23399.1 Radial spoke protein 3 containing protein
MSTGFEAPPRVVESRSKYREEDDEERLALNIMYDKRVFRGNTHNMNALAKNLTPAQLEELRVKEEREKK
KVEMIKRQLIEFKKNKNKRDETIYDLRPGPPARIEVDLTYFLTEQGKEYGPDCNNVVSQTDTFLPKPPTPK
YVPKKTGIDAETQIGDYDLFYYDDAVIPILNVVVDKTLEVALLEVEEESELKALEKFKKECERKKNKDKE
EWKKEIKAERARLKQKNELLEASR**KK**KRMMVETTHKLQRLNIAKAFLQNTFTNSLQFLNDSKYWRDEF
EDQLKSNYKEHLFAGIEQLLKNELNGKGTCNELCDSKIGGYVKKLEPIKKNYEYNLAQKHKVRMIENPN
KRVVNFMFVNPFPVRLNEFSNRLRKYFDGKLENYVTSLDAKISAYIESIKNDEIEEPEEESNPFPIRYIDTKH
LTFDISAVGRIGLSCADDPFFKLPPTHQKYFPEIIAYSHDGSIIEVIDESKAGIETITKGTFDKACRDSHLKIND
DRKACISLMLQKEVQMVVLVVRSRDTNLIPDVKKGQFDRAQFRLFDDETNQTLDEAVIKDLRLTVPTPEN
EGDEENKEPQQEPEPEEDEDQPKKAQPQNIIVVGRVALNEGKWIYERYNYVYKEDKHQDFFQMIGKLEV
ESRTYIQDKENLIREEEKALKESREAAAQAAMAKAAGKKTKKETKGKKDAKGGNDKEEAKEESKVEEE
KKENVLDIEFMPGFREALQGIYSTVFGPVTFKLKDSEWNVEHTSEAIMKTLKSQLGDKIKDCIHGFEFRMG
AEYYQSKNKKKRITKISKKGILKYSRNIQNLQIMPIVPPEKVVIPPKPEGEEGAVEGENNEAQ

>CUFF.24114.1 ubiquitin carboxyl-terminal hydrolase family protein
MEPESISKNLPKGDAVIHASGNLSFTYNSKAKTVKSEKDIEAYIEESKNYQISKYFRTNLYLDVKDDFNWL
VAEIKSIDFDKSTMVIHFDNWSHKYNEELKLNAKKVFPLRFYSEKYTGQKKSTIREFDTGLTRMDAIVDR
MKEFTKSYFATQNQEEIQEMYQFLCGECYLYIDSLITCDEKDSKNCSQQILKFVEAFFEMLVQWLKQIPSL
LEYYPFMKKYPYLYLVCPEAAILNLHKSFITSLDLIFGLSPRSEDFFKNSSNILTRSSSLSQPKTKKINLTQLL
FMDAFIDSGCLKALSRLITCVQSNKSYDMTILISILCSISQVFVKIDHTQEDAIEFMEILTKFIYSEDSSISKKI
KTLPAEQLKELKEEDIMTVIKAYYDISGDTQGKEIFCANAQLEILVGFLHSPFLD**KK**LTALIEIKKMFDRNS
RSKEVPMKTIAKWIADQNIIDYIYKEIRHPELILKSVDLLSGLARNSMLSEETLIMIWETCINEHKHEAVTES
ILQVIASIAVSLSPEMINLIIEIIHKLPIQKIGSYSMYFRTFYLNCLSVYKSSYGKGTAEKKLSKLINLNIYWEA
IQDETELPDKTKLQTLDVLVELMDALDHSNTSEFLKKAIEGLESGTSPIKCMIMIEKVLQTCARRGYPATF
KKVDLILLAIQSAEIYLNFARSNSPMDGNGIEATVFRGSLNHKDTISKYFDFISLLLRNDAHNKLSSQHVDL
MFKVFIQDSICSHERSCFYKFFTYVESDSPMQDEKKVMTSKVREHLFQNILCKYLNGENTGLCEFSCFESN
FTYINSLKKSSKPDPKDKSPEQTNNGLSLVQDLSMFSKDESIRKRCNDFLVDLYLNDKTEDYIRRGKNNR
GFLEEWLEKIQTINEKEEGAVANTLALLFNFVNRYDGHHADSQEFEKLDVEIEIENQDMPRDRRRKIIIKV
NRDMTIGAIRKRIADKFGYIPSEVLILSSRTYLSETCMLDKLSAYKECRNINIRRRTLAEREGELPRYLAAS
NRNLVKNVISKGLDSNSHYLRCQTLQFLRYIPQNSECQDKMVQCKMLQKKVETEDWLNFLCCSDLKSRE
VYFGLKIMRSIQVPYDDYQTKEEKEAFKDNLIGFYKRFISFDGFLLLCETLLEFDANNILKDVVWLSIFQM
TFDILRLMLLDQSFKIIAVKSEKMDSIIKAISKTINHCIYILELITKSIAHPYNLEESLFSKEEFKSLSESGRYLV
TSEYRKFETEFIESLFGLLKGCFYYCPEKINALYTSDFIKSGSVINLCALHPNPIAMRKAAECLVEFCDSFKD
CENVSPIPAQYFLKLLCNQLEYILSMGSLEHTDYFTLWDHILRIANAKVEDIGNIEEIVQTLFNTLQTLPVIE
DDIVHCPILAGCLLMLKAFDELFHKECKDACPDMYSPQFLDMLLSKCLFNRDKFDPSNSEITFPLCKSEVS
RKCALNLIKQIFGHDNLDQISNFLVPIIKNGSWRSNKREKWFIQSSILSHRMTHVGLVNLGCTCYMNSMM
QQFFMSPFIRSLILICKDYKKNEIAPEDNVLYQSKFLFANLIQSKMPTYNPIQFFNSIKDSNGEPMPTNEQKD
IDEFLSIYMDKIEQNIKGSEDERRLNMIFGGAFAQELICQDCPHYSSREEAFISIMVEIKNKTHVLEGLNLFI

QGEMLEGSNAYYCERCDKKVDTLKRCCIKKLPNTLILTLKRFEFDLETFTKYKLNSRCEFDFDLDMRDYC
QETLSKKNLLKKMKADNISYELLSEREKEIYNFSLPDIYYKYKLKGIVVHYGTTEGGHYYSFIKHREQNK
WYEFNDTVVRDYDLSNLAEDTFGGFIRLEQTKTQGGKQYVQSEKLHSAYVLIYEREVFIDNEKLFDLRET
ESASDLTPFLNSYRFEPWPIVIDKDIVYEISLTFDRHWISVKIYDECFLDIFSDMLVSELSYLQDHPGQISKQS
MDVDCCEDEIRKLKFATTFLLTVVLRSDSRLKYCEKLVPPIMAAISRPEFAKWFVSCFIRRHTINEYFANC
WSTQACRVICSLLECSIIQVYAEEKTNISKIVQEF

>CUFF.24546.1 Armadillo/beta-catenin repeat family protein
MEKEKLKCFEPHIVSEHLKDRHPYLQKDWDSIIDPNQITHAVFDRALSKYAEVLSNRELSPENKRDALIKL
NQLATQKDTVDEMINYNMVVIASTLLLDEDKDVREQAALLIGSFMSHQRARERIEETCPLLKLEDDELKV
REATAWTFYQMSCSRNGCDIIVNTESAVSVISSFMNYAEPKKIREQSGKYLTYLLECMANTTMYDNGIEP
LLGKGAVSCLNTILKDEEQIQKLGVHKEKIQQLSLRVVGNICLHSTGKEEAIEEKVILYAWKFLNSDNLST
CFNASHVLMNCTIHLDG**KK**QATEHHDDKNNPIIIQKIVEKLYLQDEMLRENLKTTLINISELPAGFLKITHE
LSDKFELIDEVFGTRCIKSLCELLPKFDYRDDPFNINFKEHTNYVKYVHTINKAFEKYKQEATEIAATETIN
FVEKIFPFIHPNYPTFKMTIETIKEVCSDEYNALLMKRLLGKYGEQYLTLDNERTCSLNMYLCENHKDLIDI
ISKAEDE

>CUFF.24621.1 spermatogenesis associated 4
MRQEAAAKNDPTQAVQDLDDSDDMKLDSVLIDIVSQNIDVKMSKGDPEYKIFKENYHRILPYFTERLKHI
PDDLLHDIFSELNDRVDELVAILSKNLSEFCDLSQFFCKCLMQLKPLNTVENVETT**KN**KNIFLLVVQTLALI
GNKLLNDDPMQTEVYFLEYTIDDLISIIETNTFKRNEMMYLFFCFVPQTVNSHLRVLNRLKEKVTKRDVF
YYSLSKLLVYENESSNADLDPDLYSFYFQSAAYGLMSTSPVTRTKCITILSYLSKISSELIVNLLPRIEPLVH
DNYWELQGQILILCSNCLIQFNALDEGGSQGEMEFIEGEGEVEQPVNDMDEEEELKLDQRSIDPNAHDTK
TGSQHQETMMKLSEEYTPILFRMIESIFKVDAPKATLKIGLIYLAKILHFYPEFTDRYLQILLSVPENIRSSVV
DVSPLPGTEEEVYVSGSSTEKYRTFGAPLEWNPLYVAQSLEKFIKDSGLENLEWPHIEIFEACLQQEFYEED
IDKWLEIFSSLKAYFFISLCYRDFSSTTIEILKKIYSNQHMQDKFIEESKSIFVKTMKLIYQPDVEYECQQNV
KEFLEYLHTCESGSPTLRKMVYDVIKYFADHNTKAFQKSNLIDLTTKVVEEKRGDIFRQ

>CUFF.25565.1 spliceosomal protein sap
MLALSSRPWFCYNFMSKYFVAPLSYDPIEQASCFASEQCLEGIVGILDDTLRIFKVDKLGEMFNQVIMPLQ
YTARAMTYNLDFQKLIAVESDHLCYTSKLRTEIKQQIFAATGDEDYNKVPETQVGYPKASEGTWASCIRII
DPRGPSTFDMIELPHNEAAFSLLVTNKLGKGDRTYCIVGTAKDMTLHPRTCAVGYIYIYYFDDEGKLKQF
HKTPTEDVPLCFDVIGNRLCVGIGPILRIYDLGQ**KK**LLRKCENRKFQSSVNKIRTDGSRIFVSDQSDSIHVL
KYKPEECQLYIFADDVIPRWITDFSLLDYDTIVGSDKFENVFVLRLPPGCEEDAEDDPTSTKFKWESGYLS
GAAFKFEQIAQFHTGELITSIKKCKPSSIGSECILYSTTMGSIGALIPFD**KK**EEVEFFLHLEMYLRLEALPLCG
REHVAFRSTYVPVKSIIDGDLCEQFASLDFNKQKALADELDRHPMEVLKKLEEIRNKINQ

>CUFF.25667.1 Protein phosphatase 2A regulatory B subunit (B56 family)
MNNKSSKKEKRAKGTTGGKSGKTAKDAFLKKIDVCMKIYDYKDETKDVKGKNERLSAINELQNLLQDQ
KSVVQLIVPNLDHCIHMIEKNIFRCLPNVKKRNLAFSETGIEQEEETDPAWPHLQGIYEFFLQLIINEAVEVK
LLKGFVTPEFVSEFLSLFDSEEAVERDYLKNILHKLYAKLVPRRKMIRKAINETFYQLIHEGHKFNGASELL
DILASIISGFAVPLRDEHVVFFNNVIIPLHKVQTCSEFFEQLLRCSMLFLTKDKSLAIPLLRGLLKYWPFANC
VKETLFLTELQEVLEIVEDDKIKDLIVPLFRRIVRCIGGTHLQVADRAMCFFENDYFLSKLKAYREVTFPM

LVPVIVELSDKHWHKILQESLVALKTILKEIDPIAFEDALKISKNDDRRYIVKPNQDKRDKLDQKWNKVN
NSLKQSNPGYEQPIVPFKSNKLVADFNPLYKKIYDKEKFINE

>CUFF.5318.1 MIF4G domain containing protein
MDKQTEVSDENKILMMLVRIGDENVEKIDMHIDKLCTYLCKQIGRLNQYITEVFLKCVFSIPNKQFIYAYI
LYSMANEKHEYVVDIINEVFNAFVQNINRGEDVNIVMKFIATLADTGLVTIDAFENLLRQLLEIHSNAIKE
QNDNDYFLHLAVVGYVFGRRLIRENVDPDSKPEIELTIEKYLKQRPKTTDDYYVFKSCTFEASIDQLWSA
VSYSYVDGVPLEKKIYSLYLIPSDEFIIKLNCEPISSLPDIIIESKTPYYKSPFCFELFENKCLEQSDKGLYAIS
RDLCKNTLIAFQDNPYFACQRLSSFSCIPQLPYLLFDVIFDEIFRIPSAIKKTIFYTSICVTMVKEFTKEDEFEF
GPVVGEALQIIFRSGEDANESQPSKLEEMDIELIDRLVDFLAFFLSQQNFDWFWESWNFAIDLPDYSVHKV
FIRNLLAKCCNITNIKSFVNTLPESFQVLINENK

>CUFF.8160.1 hypothetical protein OXYTRI_24202
GIVKISGLGHGYYKISFKESNISKNLIVHQGSYWRDESFILKKNSIVEFKANKKIAYVKMKKIKSHEEEKKD
IISMKICNFNENTRVHVLASNFVSSDDLMCYTKLNSVVKNSIVSEQFRLSQWKNAFMSNRKLGDEFRYVF
DRRYLKRFTGNSLDKPQILLKRMKIRDTTYEEEVVGLGDKYQSLFLGENKKMDYPQLHVEHSVCGSPGY
SPQNACLYRMESKGSNFAYPYRQTLQIANKQKYYNSGPTNYRDSLVSDQYKSYLNFLKHAGLVRSNLTP
DKNGNLSFSIPSGRYTKLSAIVCDMENVVQVDVELPTKNYILNKRNLALLKPLDPKKHYNEIRKTVNFLK
GEKIKITDISTTYYNVVDSIDKVKKLQLEIAKMDGCDICPDLLFLANWDKLSLEDKNKKYSSYFCHETNLF
LYFKDREFFNQVVQPFIMNKYEKSIIDNWLLGNYEKIESLASVEFYDNLNAIEKILLIYILVLKEDTVEIAKR
LVERIKLNSEAVEENQEYRNICFDAVINTLKEEDLKSKKSECQEIISDE

>CUFF.9356.1 dynein heavy chain family protein
MYKEGIFILTDLDEIQLQLDDCLTNINNVMGNRYIHRLKDRGEKMQRNLNLFADIFDQWKECQRNWLYL
ENIFLSEDIRTQTRNDYQEFEKVNKKVVGLMTNVNKNNKVKQHCVNKTLQDLQSSNQTMDEIQKHLETF
LENKRKDFPRFFFLSNDELLQILAAAQDIRKVEKHLNKIFENIMKIKLGEDLNSNQIYAIISAEGEVVMYSSP
IKIRTDENVEFTLKEIEAKMFETLKKRLFRFYDGYDLTNIDKSNWVFSEVGQIVAAFSQVIWTELSEYYINE
MEKENYTSLNGFLELLCAQLLQMTELIRGKLEPVERKNLISIITTDVHNRDIVVQLAKCKTSTTEDFEWQK
QLKYYIEDEQCYVRQVNATLKYGYEYMGAASRLVITPLTDRCWMTISGALHIKLGAAPAGPAGTGKTES
TKDLAKALGIQCVVFNCSEQIEYKMMARLFSGLVNQGAWACLDEFNRIDEEVLSVIAQQLLTIRFALLNN
QTLFEFNDSEMKLNHMCGVFVTMNPGYEGRTELPDNLAVLFRPVAMMIPNYGLIAEIMLYAEGFENAQP
LSVKMVQLYKLASEQLSQQKHYDFGMRAVKSVLVMAGALKRENPKLTEDVVLIRAMRDSNIPKFLADD
LPLFRALIQDLFPRANIPEVDYGELQSQIEESIQHHKLEPKDTFITKVIQLFDTFNVRFGAMIVGPTGGGKTT
CYKILADAMTKLRKDKRSADQRFQEVSISCLNPKSITMGELYGQENPDTKEWTDGLASKILRESAKDESK
NRNWCVFDGPVDALWIENMNTVLDDTMALCLANGERIKLRPEMRILFEAQDLAVASPATVSRCGMVYM
TPTDLGWVPYVNSWMKRMFSQRSDGIEPCLNEEGQEFLRELFDSYVNDSLEKLNKLKEFEPIPTYEIQSITS
VCNFLEYFISPEVGGFNISDKKNQFRNKISKYFAFSFIWGFGGSFSTKAYRYVDNIMRYNFEKLKIPPSESVF
EYCLEPEDQKFVPWSVPEFDYDIKSTYFSILVPTVDTVRYSCLLDICIQKKHIFFTGETGVGKSVIIQKNIF
SNQEERQLMPKTLNFSAQTNSSSTQQTIEADLEKKQGRKVLGAKGNNTLVIFIDDVNMPSVEKYGAQPPI
ELLRQLLCDGGFYDRQNFYSKQIEKFVCIAAAAPPSGGRASLSPRFTRYFHMFCLPQPSEETMVTIFEAIVL
GFLNSLQFSDSVRKCGSVVVGCTVDLYKQVTERLLPTPSKFHYTFNLRDISKVFQGFLMCKPISVSNPESC
ARLWIHEVCRVFSDRLIDEEGRTFFKEIIDESLKLKWRFNWAYQEVIFSNLLKLESDEQLYEEIIRLESLNN
QLENQLFEYNISHSSKMDLVFFTDAVNHICRIVRVLLQPRGNAMLIGVSGCGKQSLTRLASFMMGFKNFSI
RLSKNYKLSNFRDDLKLALLESGCEGAPYTFLLADTQIVNESFLEDINNILNTGDITNLYEDEDINKIMADII

PYVKKLGRAESRDIKYATYIERVRDQFHIILCMSPVGDSLRIRCRMFPSLVNCCTLDWYDSWSQEALIDVS
MKSMRELEDASATVKECLANMCMFTYKTVENIAAAFDQELRRKVYITPKSYLDSISNYKTFLDEKRAEL
NETINRLSNGLHKLSATGAQVSDLQKMLTELEPELK

>CUFF.9674.1 hypothetical protein TTHERM_00295790
MDSCKIYSFRRHIGVVLIIAITTMCITTFCAAQPPPYVNQGGYQGNTGGYPPTGGQYGGAQPYGAGGQPP
YGQPPQAPPYGGGQQPPPYNQPPQYGAPPTGGYGSQYPPYQGGNRGPPPPVPPVQPADGAVPPEDDVYQ
KTIMAQWDEHMGDFEPSDMLTINIPSRTDEIFYEQITEVPSKVRGAYYIGSGE**KK**LIDFWIIDPSNRIFNSIQ
GKNEGLFVFDAYQQGIYQFVFSNSRYWEAKDLTVAIHFGNHTDDHASKNSLDPFENSLNNALKDIKNLY
SEVKFQVGRQDSHNSTVKGSLRISFWMCVLEALCIIGLAGAQIYFVKRILQHKRVI

>CUFF.10955.1 hypothetical protein OXYTRI_02678
MEDNKSESLTDLRDIIREFYSLAEFSPIKAKDYTETEYCLLDKGSHAQYALNSMLQLGQGYQSVDSGQPW
IPYWLTNILEITDNSLEDLPKMLRTKLIKYL**KN**LHNDESGGFRGAKSLQSHVASTYGAILAITNIACEDAYK
IIDKRLMKNFLKSVFCTGESSTSSNQKSSELKVGQKGAYILHENGEYDLRGCYCALVVADILGLLPDEELT
SGMGDFISTCQTYEGGISCSPFGEAHAGYTFCGLASLIILGETHKINMERLLEWTVNRQLEIEGGFNGRINK
LVDSCYNFWIGSVFELIDIALEGKGNVNDGEWLNNQLALQGYTIFCCQNTEGGLKDKPRKHVDIYHTMY
SLAGTSICQYK

>CUFF.10963.1 proline-rich protein 6
MESTSDDRVTGIATCHCEKVRIEFDAPKDIVVWNCNCSICEMKRNHHFVLPQSRVKFLDGKIYIF**KN**ICIDS
EDYLTTYQFNTKVAKHMFCKICGVQSFYQPRSNPDGFAITIYCVKDYKTLFNSITWKDYDGQNWEQQIET
ADIQKYSK

>CUFF.13288.1 hypothetical protein OXYTRI_06240
MGCPRIVHSAFINLGAKLFSYFGKADEGFGLELVVEPWIENFWKTLPEAVESAKASPTEAPVSENHEEKK
QIEDFEQNKDENMYKAKIINKTTEANYFKTVLKFEIELDRELIKEDGIKPGSYCLIYPKNDPNLVNQFIKLC
NWTETEELKKDLTENLDFLQKINDKAISIINKTDKTLNLKSTAACNYYDVISYLNPESPTDKEILKYIPKLK
PRSYSLSSDCFNTNKIEICFTLEEHEQNDVFENGYSIIKKGVCSHYLSRLYDNGLEFDLKMNKSSMFDLSNF
GISCTPMIFVCKFFSVYLYKIFMLIIAHGTAVTPFIGVLRNIKSKIELKEIDKAGDIDFYFGIRNKNHDYLYRN
ELESIFKFFESQNSDGKYNLIISESRPGKLYKFIFRCRRRRICS**RR**INENISKFEFKLVASKGVLLSCGNGHTM
IKAFEKVVNQGMNKEQFLSDLTQEGRYLKEIWI

>CUFF.16824.1 hypothetical protein IMG5_076500
MELKLARFRKTTFNVAKSQFSKMMYCCIKRKYAFASKYFRPNFLSTKLFETKIDVNKDSNLSLELAKYAV
NFMNSKQFISSKVYDRAKLFHTDSILCGIAAISLRAKAPTILRDTNLAYYSNFESKPDDIRVAKMIGVSKYT
NIEKAVAANVSATHDLNSNGFSLGFANDDPSRKSEGEISNNSFYPVVIAAAHVNNKIDGNKALKAMVLLD
EIRGRLNESFDIKEYHVDNDLFGGIASTIVYGSLLNATPEQIEQAVNLLIGNYTPWRAIKTGMYELADSSEC
CPSFTSEMGVLCMNRVLDNLNVEDVQLPSHNLTLRTSGDKFSIMGMHFRFGCYSALSSGAIYSLVNILLE
NPEIFEKYEYEDIVNIRVRTFSKAYEMAGKLPRSDAPTSRQAAIYSIQYLLARMLS**KK**KALKADVKRTFQ
DFYSKLVLGPLDFTKKALLDDTTCLIMSKVDVIDGGEEFNMHYPEGLPTSISIYFNDGKRYTSGMTKFPCG
HSNNQTVDTNLMLLNKFSIFGGLGLMNKELINFIVKLQNINEMDNDELTNVYNCKIHIQKHESIDD

>CUFF.17446.1 hypothetical protein DICPUDRAFT_154051

MKFHPLDDGLLACYGNLDCIIIHIDPLKNKVLQTLKIDLMLDQLGDNLCIIDCCWLPNSRTHIAVLTPQFV
KIYDISKDTLSPAFCINQIEDCNGLNMMTSITIARDTIHPDFIQNNNLCRVYVGTANGVVYTLQLDIQEEVP
EHDSLYLVEDLQINSDITEI**KN**STAGSVIFLFYSSLIDALFISFECGVTLY**KK**PTLSDNGVLSLCNPVHLDPS
KTPCNSQSSIANWNKSNCKNVHFQDLCIKGNSAYVSCWRVSNDNCFPLILEFSQKSIQIHMYSNQIHTIGT
AYIRDISSVSDRPGLLVVNDKGTVSLLPMSGALPFSEASLSASAQGSNLKAQFTVDMLPKEVKMNVDLFE
NSIELTSQQFDSVFHFTGEFKTLLASNRSSVNIMRPGVEALIKGGTKKSIKMLAKLDCPSGDKQANDTVIV
GMRVCIWNSDKFGSSNAEGKLSLKLFNREIKLRPDHKMHEIGFCDAEILYLNLVMKNELTVEFVTKDPTN
YPINIYGVDVFGKQK

>CUFF.21364.1 arrestin domain protein
MGYPASSIEIEVKGKEKCRWVTRESKQVKEGENTKTEFVDVYHKGEKQVLSYQVPIFYFPGGMAPPGQY
SFPFSFSLPSSIPATLYFCGLDKAVARIKYNLKAVLESKIGFNV**KK**MQFKQPLVIRQPNYSTALNPIQTDTR
NVYACCCFGNKGQARITTQFEKDGYTPIEICKAMCDIDNSQCTGVINSISIRLEQFVELKSGDGQNYHNRH
VLEQKNYDGLGANQSTGGMNRYLELDLSGIKQQAKSFEDGKILRGDDLYLAERLQPTSTGSIIKLYYQLT
VFANYGSCCAEEPHCTIPLTITPPPLPSYGIVSAPPQWAPTMYSCFNFSLPSPGQVMAATVQPVVPLNANIS
FKVNEEEKFSHVPVPVPVPVSAKVSMPVPMPVPVPGIKLSIDTDLANRENPIPVPVPGFHVSAG

>CUFF.21956.1 serine threonine kinase
MSDIKLKIRHVKRGGETKLDISGMGLIEIPDEIFELIQLETLDISDNRILSLDKISSLYKLKNLYAQ**KN**KISILP
EAIKDLPKLENLKLEGNPIAVNNTELSSVFGVKKVQETLDAYFDKLKDSPVTTSTKPGFLSSTSSLNDVVF
LR**KK**IAELQLELAEYKGASGSATKSLEEQKDWMSSTAARPATASAQVKKVKDLEDELKTERNINRKLNN
EIDMLKDEISKAKVLTSTSGEEGTIGSVPGVMEIPYDELDMEDQIGQGGFSVIKKGSWRSTDVAVKIIFDPV
ITEDLISEVRNEVQMLSLLRHPKIVMLMGMSSKPPNLAIVFEYMPKGCLFDLLHTSSVEIPIEQRLRWSLDI
AQLFSFLHKSGVVHRDLKSYNILVDENLNVKLGDFGLCKFKADLNRGTMQFSGTPTYMAPELFQKKSYD
EKVDVFAFGTLLWELVAREVPFDGLEPADIKDKVMAEEHLKVPFGTNKKIATLIKDCRTLKPSSRPDFEYI
AGVLKGVVL

>CUFF.24220.1 UNKNOWN
MAESTTRDFSKLITIYGEFNYEFKFFTQTNYVYLASNLVAEAKDKETGLPFAMKCKWQRIRGKRVYNLN
AIHGNVYQLSAEDVGCKIKVEVIPMEEDLYEGNAYAEFGPVTVEPSARQSLEYILGSGGSRFPVTIYYPED
RHKMIDERMFKEGTLIISNKTIKLEEKQDLSGKSKEVIFDCKYTIDHPKIDISSNDTKMLSIDYYDDDDFYG
SEKRLNFSLDLRALSRQSRDLIALSIRCFSALNYFKNSKIINTLNQDDDDLDGSPLKKSQKEKDALTELLIE
VDFVKRELYEQIGINKELEVERNNARKQFLDLENEMEQTLEGYRIVLQEQEHSPEDTQLRTEMRNQLKLN
ETMQKEKNQLAEENMVLMDEKKTLN**KK**VTEKEEMIDSIKGQLFELQKGQVMPKNEYEKLKQEKEKCR
KDAKKFAEEIRMLE**KK**LLSMKSDAKAASQEKEQLETEKRTTFSYLKSQEDQIEELKTKVDQMEKENRQL
KRKVEPEEKEGSTADRLKDENEALIAQRNALSKKNESLLKDLERKTKELDKYRVDSEGSVTRLTETNKRF
LDVQKDMESKIDELEEKNERLLSDKNEIIFKQKEEIESLKGKLKEKSSVANKFKELFSKKQEEDENKPKMI
SCEIQTEEEQKTLETSQSTVELGMRKYSKAPKGSFLNNTQNMSLESMNGTSKELEDMQRKLKLVEKERK
QWVREKKESEENYNSLKRMLEKKDDQLEEMRVEIERLAGKLCAAESKLYEVTG

>CUFF.24259.1 mycbp-associated protein
MSRTQSRVPSDGRDMRDHTSSKNITGPQIVFDKEYLCMKSLNDEISTDQVKITNVGSTTIYFVWKKVERG
DYIPA**KN**SDGVQRFFWNHCKNKLLPKESKTYTISFRSTVHGIFFEEYEIITEPTCLTPLKVLTLNGVSLQEEC
DVNEFERFDNEVQSITQKNFFNEIVTDIVEGVRTPTPPLPDMIVPEVFAREFERNNKKYGLYFGNYEMNAF

YELINEAHMRIGTRPEDDYWDGSIDYIYDLIQRVDSEISRNNLMITFNRLVNICKKQPFEKSLAYSELRDVII
RMSQVIPALDEKFREELGMMEHLFEYITDEMTPEEINKMKEDKENRKNEWNKKRKKPPKTEQEEAHDNG
ELHAKISEAIKHKVVSENEVMGGVEENIEMKTLEKQLNEHIIMNREKWDEFYRKKSVIDIGCEGKNVLLR
LDLDVPLSEYIPPEEKTILDKDGKPGAKVVTEAPHDASARSDFKSNPPTSSKRSKLDNLNTSERNSKERTD
NNILATRQIIDSTKIKKCLPMIKYMIENLATRTFIVANLSDKSGKFKAQNTMRFVWNALTSKVDQPIHFQD
EYELEHDSEGIFMLENLNFHPEEWGFIEPDPIVKPENPEGEGDEEKEPEPVDDKNKGKGNPKDKKAEEAK
KKAEEEAKKKAEEEKKQKELEMKKAAEEGEQQNMEGEGEGEAEPEPIPDITYKEIEAFKSKLSSLADIYIN
DALDASLTHSNTIADLRIPVKVMGIRMTEEVRKLGMFFKYPHSPTFAIVGGTFKCNISDRILLLNSLIYSTD
TIFICGGFALYFLKALGIKLGSQHSIIQDKYLKLCKDILLKAYHKGVKIILPTDFVTLSKPKLGADTEVKTGE
IGHETTDHMTSFKPGETAIDTVGEVNWIDLVYDESKQIVDFSNFIEKRLHELYNPDEVSHTPINNSPPTEEN
KEGEAALDSTPHVDPVPSHELESKAYILEYGGSTMQNVLAHTKDAMKLFWDGSISIYPDTFAHENNKTM
TFDLLRIRTENEDRTEPKYSLIHSEETEIVVNQSMAKIKLDQMDSKDPEGSMGGNEESDSRTSTFQHENISP
SIFTCAEGKFTLKILQGIENKWMLNVDEHPALTKQQIEDDLAVLEEI

>CUFF.24744.1 kinesin
MSSVKVAVRVRPFNKREVDLNCVCNIEMIGKMTAITDPSTGQKKKFYFDYSYWSHDGFTTDPETGMFEK
DSPDSNYASQTHVFNDLGCEVLDNAFEGYHTCLFAYGQTGSGKSYSIFGYE**KN**IGIIPMACAEIFRRIELGQ
ESGEDIKYSVTISMLEIYNECVQDLFVKPKNRPKAGLKIRETKDGGIYVEELTSIPVQSYDDISDQIEYGTA
NRTIGATNMNATSSRAHTVTTITFKQTYYTAGTPTNQKESNINLVDLAGSERQRTTGADNDRLKEGSNIN
KSLSFLGKVISILADKAAGKKSAANTVVPYRESKLTRILQTALGGNSKTAMIAAVSPADVNFEESYSTLIY
ANQVKSIKNQAKVNENPQDKLIRELKEENEKLK
>CUFF.25026.1 UNKNOWN
MADLLVELLENDPYVEEINLDFKDIDELESILPLLSKFPNLKALSLSDNRIVSVPDDLSCLEHVEDINLNGNI
FEDLKQVILALTTMPSLRSLHINLHEEEQVDFIFRNMPHLEMLNDTEVEREELEGEDNQTYEEEEHDMING
DAHTPGEREEVDRHQRNTDEDSDDKQDLNDDQNQAAHEDMEGEGDYNQKLESGEDIEDIVIKPEDLEIIA
VLYDSIRGLRRRIDEENDKNLADDFDRHLKNVMTELKESLTTDDPTHIKNANLLKAKFSLHEICFTKAVE
YLKAMDEEVGDVFEKIQDIYNSIFGNCYELIRTSGGNDTEAIRNQLIEKENEATEVIETANELNKRFEKEM
KEKETLKRNFERERNQL**KK**QVESLESENKKLLDTLIRHSKGENIDNAVSQKSAQVSGHPLFKGTQSRKFN
KLSGPVGKTQVRTLTLKQLKEVINDMYAQKLKYDQKCVESKLPRETMEQYMYTYLNQRYGLKSLIIEW
VAAIINGIKTYLKQDHDVALFGKILKNECDEEFRFIQTHVKETLVGLLRSLLRERFPLKSESEINKLMKEVQ
NGSMEEWQWRKIIEKMYDPNDYEILEQKFIGCIQDRKAKSFYNALTDSRKLTREEQLIRMQQKDYERLLF
SDFQRIILDFQLKEHEKFLEKFIVVFKNVDTDSNGIINEPEFRELVQSMEVITDEEEINYLLQMIDPFNNQQM
TFSELVHLFSSHMVPIDERDPQKSIPLLEKFVSQSQESEHVGEILKEEDSNEERDEEDEEAIEMM

>CUFF.2506.1 von Willebrand domain-containing protein
MQSILLPNSGINLFNSGRRPCKCRCIRRPYWFINPIVSLVSNGLTDKEIPLKSSKLEARIFNSVASIKYSQTYV
NNEKVPLECVYKFPADPYFTVTGMHITLDDKEIDAVIMEKEEAKEKYDDAVAAGNTAAKINYDENIPDVI
ELSIGAIQPDKKVKIEVSMVAKCDVIKHGYYSFIFPINFIPKYNPHRADKINQSSSRGKCSPGKFSCNIIVEST
SEISDLSVSNKGIKYVQSDDGKKVTINLGETKSVLSKDIVVSYSTEEIRAPSIVLHQSDKHPNKVAAHISFIP
RVSDEHVEEEVKEEADEDVEEKKVLSKIDDKDDPDIASGEFIFVLDRSGSMMENRITTAIEALKLFVQSLPS
DSKFNIVSFGTKFVFMYKTSQKYDKNIVDQTLTKVNRFKANMHGTEMLKPMEAIFKLKNNPKYPRNVFL
LTDGAISNTDEVVSKIRKFNYSTRVHTFGIGSGASAYLVKETAKAGLGTSAMIADNDPKIKEKVIQALKIA
AKPAFTDIKVDWKSNSNALEFQCPRPPISGNIYEEESFDIYAIFSKKDLVESDIELSFFNTFNQDKGSVTLHI
DPAKIVDAGDDDCAFKMAAKENIIHLKRSAEEGKEPSAEILGLSLMYSVLCDQTAFFGKIKNKEKSTEEM

KTVEIPILKYRDSSISHAPVFIGGYGRGYLKTGHGFGVMRSAGLKYKTKCKSRSINSSMRPQSAVL**KK**SST
LASAHRKRQAKADEKECNEDLTFCDDIQEPDDFQVPNKELQKKKTTPSSLSSTRNNYQEIVSLQETDGSF
KSLPDKYKSISKKKHPAKLEALVTKKSNLTCIWMTILALAILERDCQPSKGEWIMIAKKAKAYLKKNGVA
DFSMFVQLD

>CUFF.25193.1 Eukaryotic DNA topoisomerase I, catalytic core family protein
MSESKSSKKKSELGKRKAGDHPTPLPSATKSTKHTESQVEESVIDTNRSVTHDNSGLDKEKFEVLSDNFNE
EEEECKWKSLEHHGVRFPPFYKPHGVPLLHKGAKIHLNAELEEIANYWAQIIGSEFAEKETVKANFSNEF
MKRLNPECGVTNFDDLDFTVI**KN**HLEKSKEERANRSTDEKKKEREENAAFEAYYRYCMIDGEREKVSTV
MVEPPGIFRGRGEHPLAGKLKSRILPEFVTINVGPDNLIPKCNVPGHSWKAVVSKPECTWLGNFKDENNE
KANVKYLFLAADSKIKSKNDRKKYKKAKLLKENIAKIQKDYMKKMASTSELDAQLGTATYLIDKLALRV
GNEKGEDEADTVGCCSLRVEHVKILDNNKIELDFLGKDSMRYNQVISIDPLAHAAISRFVKGKKPEEDLF
HLINASRLNEYLKELLPFLSAKIFRTYNASFTLQKELGKFEEGPKDEVEDKVKFYKNANREVAKLCNHQR
AVPKNYDEQLKKLEEKLQEKKDKLKELENHKKLLKKDKGADKKSMPKTIQACDTAITKAKKSLTLEEN
KVYEKKENKNFALSTSKINYMDPRISVTWCKNYEVPIERVFEKTLRMKFAWAMNVDPEWKF

>CUFF.25539.1 Protein kinase domain containing protein
MEPGYDDEEDKKNVFENIETTFSTTDIRTVYKFKKLIGGGHFGTVRIAYRKTDPE**KK**YAVKSILRESMKK
DVAMLESEINILKELDHPNIVRFYETYIDYKYIHIVMQLCTGGELFDRIVKLEKFSEKDASELMKKILSAVQ
HLHKHNICHRDLKPENFLFKNNKENAEIKIIDFGLSKKFSKQEENMTTIVGTPFYVAPEVLSGKYDTQCDL
WSCGVILFVLLCGYPPFDGDSNKDIFRAILKNKLEFDEEEWGNISDEAKDLISKLLVKDPKKRIKIDIALNH
PWFEKWENTSEDADTFQTEYLKRMKSYRAPNRLQHEVLSFLMKNLDTSERVKIKEVFRNITSKTSGDLTF
KDLESAFNEASIDGATDNLLELKSCLNFDKEGKIKYTEFLVAAVNKNEALTEANIEFAFHHFDTDNNGLIT
LDNLVEAFHREGKGLTEEEIKEILAQADISNTGQISLEDFKKIMKYDLHKHMMEDK

>CUFF.25544.1 hypothetical protein PHAVU_002G014600g
MHTKATISAIDWKPDGNMFITAATDGVCRLWNAKGELSVVMYNDNAMPLKSKDTYFVYPNGTNDSSQT
HIATPDDIDSISDCKWNKDGTAIVTVSEKNNVILWNTEGKLRHSYQGHTESVVKIDW**KN**SNIFATGSQDGI
IKIWDVQSSSAIKTFNAHENSVKCLKWDYSGALLASGSEDCTIKIWSPKHDKPLCTFNENKEMIHSLKWTP
TGLGTNNESFEVRLASCSADGTIKIWDVNESK**C**IDTLHGHNGMVMCIDFDPTYKYLVSGDDMGRIIIWSV
KDGTIIKTFENKNKKCIFDAQWSYDGLMLAACYSEVSIIDIRNI

>CUFF.25615.1 Long-chain acyl-CoA synthetases (AMP-forming)
MEKKLNPSNKEFIIMSVEGYKKIAKVDYTGKNLHWTTDPNLEMPVKLHLQKLGEEVLLPKTVSQCFIETV
EKYPNLPCCHCETTVGNWKYWTWAQAWDICFAFAKALVSCGISKRSAVNIIG**FN**SPEWLFSFYGSILADC
IGVGVYTTNSPGACQYVAEHSSSEIIIAENEKQLKKYLEVVEHLPNLKVIVVWGETSIRNRPENIKVLTWD
EFLQYGIYCDKEENGKLTQIINSRIEEQMPGMCCNIVYTSGTTGNPKGVMLTHDNMVFTFDTFFKELQIQG
IEAGKERIVTYLPLSHIAAQVHDIMSNLMIGTQLYFARPDALQGSLVETLQYARPTMFLGVPRVWEKMEE
KLKEVASQAPLILQKISGWAKGKGALNSAAKMQNKGHPFGYSLAHFLVLGKVKKALGLDCAKILIVGAA
PIKASTYEYFASLDLPIINIYGMSESTGPTTNSRAGKFKGDTVGYALKQTHIKIDKRDKTEGGQENEGEVCF
RGRNNFIGYLNNEKATRETLDQDGYIHSGDLGTKDSDDFVKITGRIKELIITAGGENVSPVLIEDSLKNLCPI
ISNVMIVGDAKKYLGALITLKVEVDMTTGLNTPTKALTTTVRNFLRDHLELGDITTTDEAIKNDKVTRYIQ
SKIDENNQTTISRAQNIRKFKFLPIDFSIEGGELTPTLKLKRKVV

>CUFF.25798.1 UNKNOWN
MSRATEKDLWLSNMSEEEIRKKRAFVWGWGKNKNGELSLGVTKDAVFPRLIKGLSGKVVVFISSGANHS
AAITADGELLVCGSYLHDKLGIEELRATNVLKFTPCTYMRGLVVKKVACGDYHTLCLLEDGSVHAWGG
TLHKKLGKPKDAHYPLKVPLPYLIESLLHTVVVDISCGDFHSMALDEDGRLYTWGGGGASYNKGQCGH
GDENDEDDPKFVEFFDGVRI**KK**IDAGGFHTLACTENNDVYAWGQGVYGECGYGEYYNVNKPHKVKMP
KADLKYGVAKSSYNRSQDDSDSQDNIKIKCIAAGAHHSMILSEDGQLFTFGYGQHGQLGQKSNQNYCTP
QIVKDFLAQPLSCIAAGWHHSLALTRKGDLYACG

>CUFF.26157.1 guanylate-binding n-terminal domain containing protein
MGSIDENALNSLSFVISLTKHIQIKSSGMAYDTDPEEYSQYFPSFMWVVRDFALQLVDSEGESMTPKDYLE
KALTNQKGFSEAAEQKNRIRRLLKCFFKERDCSVIVRPLTQEGDLQTLETKDFDSLRDEFKQQVLQLRSK
VMKRVRPKTILNGQKVNGAMLAGLIASYVDAINKGAVPSIENAWSYISKSECQKAVEKSYEHFVDEFMS
SFEMNAPVYEEELIEMYKEAKASALTIFDQNSVGEVAPDFKNDLKLKFKEKFSQVKAENEKEVQNQAQIY
LQNYFTPIEDKLRNQKYSTFAEFEKDIKDVEQIFLERGPPGPNRKLICLTYIVSALTDGCDYFTRSMASELK
LQATMSEESRSKYEARIIELKSDFNKAKDDYENKLRTCETEKAQSIAKEQSARESLNDMKREKENSEKEY
KARMQSEKSESLRLIEEYKSRMYSSEEAAKESQRRMLSTESENDKQRALLEQKITFMESTIENLKQKDKD
NQAEIKNQKKELLSSIKENSSKYEDKITALNKRIEQLSEELGEKDNKISEFEQKDYLLTSTINEKNIELESLR
VVSTEYDQLKDDYDKLKKDLDENKKFYSKELQEERDSLVLQLDELKQFVAQKNVEYTSDKNKWEKNNI
IQQQKIDFLNSQLHEKCEQIEENRKTHEAMIKAIQSRESEKGEAIEEADKKLEELRQSHYQEMKELEERYE
TNNKRLTEELNKLRKSESESKMQFKIEATEKDKEIADLTEALAQSETLKERAQKDVKQLEAQKQSIIQQTE
DRYKATIRQLEQELEQKEEKTQSEVGDMQQRFENDLSQLKSYYEAEKERLERRVQEEKDKGIKNLKSQQ
EEYEQRMMDDQQQHDEDMENLQEELREKEYQLQQLSNQFDHESSLNAQKITSLESYIKENKKAFDDAQE
KHTVVLDQQINSFNKERKEMLTKINEYSQLLTEKEKRITTLENQNESIIAQSKKKDEELDVLRLETGSSKK
GLEEIIDKLRAENQTISDELMQRKLESGRDQALTQQKIEFQEKKIEDLLKALADCNKNYQEKIETQKNEFG
QEIDSKILQLTNEKEKIAQRFEEKRKELKELERNNAVLSAKYEREKAVFIEKIENLERKITNEQESYQRQIE
NYKEKIFELQDQGSKSVGKYEIKFDELKVKYDSLEKEKNDLVSRYEKDEILWKGNFNLVAIKILF**KR**QTR
FPRRTEIPS

>CUFF.26213.1 Dihydrolipoamide succinyltransferase
MQIVTRRIFSKAYNRAVCSYSRLLMNSKLRFFASRVVKCPTMGDSITEGTVQSYSAKAGDFVKRDDVVA
VVETDKVMVDIRAPEDGVI**KK**YFATEGQTILVGADFFEIETGVSGESKQTSTGSSSSSSKSSEPPKPKEETK
EVPKAQAPPKQETQQPKAESKPASPKPSCTPSSPEKKPATPKGVPPPPGMQVGKEGLSTITGSRTETRVKM
SRMRQTIAKRLKDAQNTNAMLTTFNEVDMSAFMELRKQFQDSFSKKHGVKLGFMGAFVRGVVQAAKE
QPVVNAVIEGDEIVYRDYVDISVAVSTPTGLVVPVLRNCENMGYADVERSLVELSLKARDGKIGLEDMK
GGTFTITNGGIFGSMMGTPIINPPQSAILGMHAIKNRPVCVGDKIVARPIMYLALTYDHRLIDGREAVVFLK
KIKECVEDPNNVLFDL

>CUFF.26214.1 cation channel family protein
MQIEEMILLIFSKRYEINCSTSISTFKPIPHAQVGEDPLNKFIKGGQVVRLQHTELGGYLTSDDLDFTDDQL
AEVYVRAYNGDRFDMEATTSGDLFEVEIAHNSDRGQICSFNEEIETTTQFYYRLRHLNSGRLVRSQTVTLS
GKKINTLGLADHIDDKNAGELMRSSLFKLFSTTVDNDNRIRSGETTKIFSIKTLEYLCTKEDQEWTGGVQG
GGGRAQSRAQSRGFQRPKTKGGYDDFIEDPDDVKHEETKGAAGKRNKGKSNEIYRPIDDEFYNSRNLVV
ELNPRAANKDAFLISLVNENEVKDLLFVQTVGTQLYTFTKYLRAKRINDITPDMYTKAITNLSKLIFFVTK
TESTDPVRCEGIPIKSRQKLMRELRIIELLVDCLYYPFSSGAFKIKELTNDMPIKMICVLCYRLILHSVKNYK

NNELYASQWIDLYFDHSMTSAETNLRAEATIAVLISSNKTLLEKQITRQTIEKFINLTKEQEKDERFITLLGA
LCSCMEEAIPSNQNNIIELMFENQKNKDALNIPIRENGDHVEICFEDNLDQSWININALKEYSSKQEGNNKL
YKYYLAFIDLVSDTCIDRNRRALNSLCDIYQLSIIFKVITNKKLEYPIRSRFLRMMYHMYINKDPYGEIVVP
NLTRIWNEVNVIGNKVVFYKEELPQYINDLKMYILYYIKDIKGIMSIFENDKNKMTLQVVKLARCMITFGF
YKTKDELREISIALITLLNGSNDIYELLQEKLARENRFGSMKRSIRIDDTNQNVKRYEKNEDNLIIMKCKQY
ICETLLKILDLEYDLKISLFLGFLKEEIESYYSMDNSPLDQEFANHAATSKVFPQDNIKKRSTADNDEYKNR
LLYIPQPKEKVSFEENSAIIWMEKVLMGEQLFDGYSQTTYVCVLMDLIAYQHLPLSNKAFELLTKFFTQRS
NLLELLKQIQLLEHPESIQTLNKCIDVLQRLRKFIEAIDTWLNEETLKLNSNDIAVGLVSSRESVDYLCQILI
DRKQIKKQGTLKSNNDSNYEIIHGFKNNSGDLVIIDEEGNVLRKSEYSNNLIISNNENQRLLRNLGAHEIIIEI
IKYNISQKKTHSDYEDIIKSAYIFLIRFCKDNSVNQGLVGEYLDIFIKELNSNPLCIFLIREIFKDNKFYLSIRG
PKVVKHIVKKEESIPITLPEKYHYLNTLKTFARCKSKIVKKNQNEILINVSNDDNENICSYFNTKDGISKIKG
IIEKLSKAKHDQKGDIAEIEFPIELYNLITFLDLICVCCEGKNEFAELKAQSSVCKIKDILQLLQASDQFIPFK
FSLVMYLYHAWLIVGNNEVFSEEENEDTLWKIVNILVKDLEDKNNEDSNDDSKIIYGPPYNQSLKQISDSY
IYTAVISALCSTIKLALNYHEKDDIITRIITTTCELYYKADSNELKKPAFDLISYSYNSSTFSKYLENLRHPILS
GDEPNSDDDEDTEIGKNKSKSKYNSRRATQVVGIAERTKASIFAHKINSITKYEEMEELLEKEFEELIKWY
AEFSEICDPEFQGFDPITHLFNLLDPENTSLSIDLQITGLKIIRKIVETCNDQMVTPAADWDNEDWEHCMAK
IDIRQDYLVTKGCIHFLCNYISKCENVEAFDEAILACIALCLGGNYESQEEFLNYMMADNSNLFLLKIKDMI
AKKFEVVKILVAEKSAQLLRQYMMEEEGDNYNSRIHQTTMTGVGDYIITTEDADDIGDIEEDNEINSETET
VESAVININRIFRFLQLLCENHFLGNQDYLREQKVDDVINPKSFDFVAYISNLFGIFIKGYVNCYSSDIGDLL
MSLLTELIQGPCKGNQTTEINNKVIENCTELIFSYNSPKLLKQKGFVGDKAIELDELKQHCVTLLLSLIEGK
CDNFLKKSMIQAIDNFYIVFQRMHEIYEKFVDEELRLNPKTASLSQVTNHLKNDSFDCFIQEGFELYILIKL
LMDDKDPEAMKRYKEYEQQLPDEDDPDSMHRSIKFYKKFVGSCEVVVKGELFRVYFPIPPVCRFLSSQA
KDEFLLNVDRDSPQLKIAGFIEAMPDLEDNMMNTEKLNRGILKITQDTVLKIRTISYLVALTINIIILSHYEIQ
STSDGTKPYYMSGESDNYRYKNAMDLGLLRLQ

>CUFF.26556.1 casein kinase
MVISLLGPSLESLFNSLKSQFSLHTTLMFGIQALDRIEHIQSNDFLHRDIKPDNFLIGNKEPNIIYMIDFGLAK
RFRNPKTGAHIPWKDGKNLTGTARYASLNTHLGYEQSRRDDLECLAYVMLYFVKGKLPWQGLAAKTK
NEKYEKIKECKKNITVEDLCAKLPAEFTKYLNYCRSLNFEDKPCISDLKKLFTKLLKKKNLDLEGKFDWV
TKRAKIPQMIEFEEAENDEPDYKRKLKDLLKNKE

>CUFF.27841.1 UNKNOWN
MYFDLIQVLIITVFAITLLLIPVYILYNSRTFFEEESVNQYSLGNLGSSSVVCSSSFLDEDNFVESCMIGKIAS
LTAVGVLPSGLSINDACVRNAKTSTCDGYFDKQKFLADFNRNCVGKSNCQMKGFKGTYIKSGSQECSDN
QSIGFYRFTCKHDNQKNSASVAESWFLTFINILIGIIFLTVLRCFRYRTGQEFEIYDQETVTASDFSTEIDISES
MFDTFKNKFERRAKKEDSNKEIDRYSLIYEFKLALSETIEKEISKIPAISKEIENTNIISINFSFNNKDVFDLLS
ERGGLLVEGEIIEAKEIESKIDELIHFFYSQLGVPCTALIIFEEEEACQRALCTEEGDITFCNEPIKFKEAPEPS
DVIWEYNNQSRYEFWRKAGIAMLVVLGVVFVNFLIVFVLKSWVIHDYGDINCDFIDTIYPTKEALLKSAL
DEAYAYSKRGNKSLLKGALKCFCDEEWEENGYFSTKEAQYSHAGVVDQKGGEYIDNICEDYLKDSYWS
FFIMLFLGIFIFVMNIIMKQIIDNSVKWVGFKTQSKLTNALIIARFVTTFIDTAIILIVRRGSIAGSSFPYFGAFI
DSIYYDFDLEWYTEIGEAIVIDNFFNAISPFIDFGFDYLIQNLERLNDKGGCNCDKYNTKCKSVQQYVDLY
GGFDYKLYSMYSYIISMTWITFMFGTGLPLLYPVYLFSLIMIWVSERLFLSYIYKQPPMFDDKLSNTTVDL
CGYGFLIQMFFGYRMFNNGEFFDAASKTNEDLFSVDTITGSFEINHTFPFIVFGVLFIIFTICDTIFSRVSKFG

KSKWGYEGLPPFQHCLKENDVDWILSEEKYLREKENIKILSDRFYESLNSIKNAESKKESKNIRKIQNTPTY
DILANQIYQERFQYFSVNERGQGSDPIFMSNNVRKLLFLPYLTPNQTSNFKFSNTLFTKLKKK

>CUFF.28074.1 Serine/threonine protein kinase
MKSRIEKYEKIDKIGEGTYGVVFKAKDTSTGELYALKKIRLESEDEGIPSTAIREIALLKELQHPNIVKLHD
VIHTDKKLTLVFEYLDQDLKKLLDLSGPEGLDMQTVKSFLKQLLKGIAHCHKNRVLHRDLKPQNLLINRE
GVLKLADFGLARAFGIPVKNYTHEVVTLWYRAPDILMGS**KK**YSTSVDIWSVGCIFAELVNRKPLFAGASE
KDQLDKIFKIRGTPTTDDWPDMTELPLYEEDLPQYDAKSLAELVPDLPDDGLDLLEQMLQCNPAKRVSA
AEALKHPFLEDAIDIEG

>CUFF.28134.1 Inosine triphosphate pyrophosphatase
MNKLGRFLGIKLIMESTSTKKITFITGNKKKLEEFQAIMSDVTEVEFDSMAVDLDEYQGKPEDIAT**KK**AKL
AATYSSNPILVEDTSLGFKAFGGLPGPYIKDFLSNLKPEGLHRMVSPFDDHSATAMCIFAYCENKDSEPTL
FVGKCEGKIVEPRGSRDFGWDCVFEPTGYELTFGELDKTIKNEISHRARAIAKLKEYLRTKE

>CUFF.28766.1 Phosphatidylinositol-4-phosphate 5-Kinase family protein
MNLEQYRLMMGVLGETYLTERMFSAMDHDGDYAITLEEYLSYNDVISNGTTIEKREQNFRMLNDNKDSI
VTYSEFEDFVLKILDMYSRSVSEKIDTNREMIREVFDKIAKRGKNEFTFEDYVKALEKDPDLFGWLERPKE
MLNEILNQEEGKYSKHFVNDLIDILFKYITTTELAMKQCMDLVRAIKSNEQKAVQTHEETVDYSAIGGEIV
NFLLDIGRSDN**KN**DFSMFNTKKGKSKDQVRKIFTGNLLSTKRESWNANKRNYTGNSSDEEEEKEGGTES
DSEEESDAGSYANDSDEELTKSYSVEAEEGKKRDEIEIIHSKFERKITNALGNKRDEPIDTLEKICSSLVDYS
KNLDLQLQEKISNEINKGKVEIKAKEYIKDSTIYNKTNFDARPSSAKRKSEANNENKDLPQDREFIAFGHE
KFDLVFNIMLGIKRSVDCIFHSPFSQIRKSDYKAQYEYKNEWYSAKEDSANLFIFIDYAPKVFEDIRIKDGV
SNDSYIEALGPNNIYNYIWTNDFKSFTSLVSSGKSGSLFYYTQNGKFMLKTIAKDEFNKLLATLQDYHIHL
VQNPDSLMNRFYGLHKIKYQDSNRAKEQYIIIMNNMFRNFQPDIKYDLKGSTIGRTTEFKDGKIDKKIALK
DNNFTESHQKVYLEKSDLESILSCCKKDSDFLGRNTTLDYSLLLGIIDLEEVRLKIKDNPKLQSENDPIVKV
AKEGKLETKERGIYISSNKKELYVIGIIDTLTNYTTKKKLEYHYKRCKDGHQMSCIPPPLYAERFYEFMKD
TIFKQ

>CUFF.28794.1 cAMP-dependent protein kinase regulatory subunit
MKHEKQKAQGAIISPILEKLVNRLLIIKPDDPIPYMIQFLEDSSGKGARPLSKKETEELAKLRKQYVKYRAK
LAETHENEDAKMADEDHSSSDEAEYIDELPDVAMRKMTGPARNSVSAEAYGVYH**KK**SMLKVRVVPKS
DELKTQIRTRLLQSFLFSSLDDKDLEIVINAMEVKTFEPSSTVIKQGDDGAELFLVGEGTLDCFKVMKKGE
EAKLIKEYEPGDAFGELALLYNAPRAATIKAKTEAVLYSLDRDTFNLIVKDSAAKKREKYEKCLKNVKVL
DSVSIYERSQIADAIKEQKFNPGDYVIKQGEIGDTFYMISEGEATAYKVFEEGGKEEEVMKYTYGDYFGEI
ALLKNEPRAASVVAKTELVVMKLDRNSFRRMIGPLEDILQRNMENYKMILDS

>CUFF.29253.1 ubiquitin carboxyl-terminal hydrolase family protein
MKMKLLENLLYPDAFYNIDLRDEFVNNCGIKFLIDIFVQALTVLTKDDNKEKLPLCASLISKTLKLFSKVIA
NIEFNSVIDRENSLVLVKSGLDFLEYISTTMQNDLETAGKLANQFEKDNIIIDIFQMFTIIVMNDHSRFKKIY
SHKKLREIIFYFLIDTNYDYMKKDISYSLIDITRRCTSIGKANFDCKTMPGDYFINVLHQDYLPIILEKIYTDS
NINVNKAKSKKLEEIEKLKISKCIYFFDLFANLINEVEELDQQQGTQILEPLLKEFKSCKRIENNEDVEDKRL
AHLISLISAIFRKSSQLKQEFDDGEFFEFVLTDCLFRRRKNKSEINYPICKSDLTREKCFELLIEIMNTKKNKY
FAKFAKIVTKWMQRAKWRTAQEKDWEIKSFDRIKNRRSKFINSSEYIGLENLGCTCYMNSVLQQLYMIVP

LRLAIQTVKNQRENDAKDLDTLYHTKLLFASLSNAGSSYHNPEHFFKTIKDIDGSDLNPLEQRDADEFLAR
FFDIIEPQIKSTKEEKNISNIFHGSFANQMICIDCPHKSEIVENFTTISLQVKNKHSLEEALDSFIESEILQGDN
AYYCDKCEKKVSCRRRTCIKKLPNVMVIALKRFDINYETMQHSKINDRVSFPFEIKMDKFTDKQLEKVDL
LKEMEEMNWSYEDLSEDKKRIHDFEYPEEYYSYSLRGVVVHMGEANSGHYYSYIKDTRTGEWYEFNDT
NVSSFDPSEMDEKAFGGEYGEDNKKYSRFRNIGFKPYNAYLLLYERNFYIETNDFMEKVDIPGEDLQKFF
NMRFSRLESNVANDNYHDTEVGDVVSNHNETLWESKQLFSYSFAKLCFQISNSYSYDKAGKDIMREVRS
TNLHDFDHLPKSSKVKWLSTFHRQAITVIYFFTVILRSTAKPYFSEYCSTIRHALSNYYPISFFLIENFCKSDI
LQEFITLRKLSVLRMMVPYFIRLACKRVYEEEEELIIQYCKLTEDYPDNPDKVVKNSKGKSKTEVLYDSIKI
KDHRDRVKEVDECVAYVYDNNHDIPLLVIFANKLLKLARDFYEDIDITQKYHALSNFFELFYHVADSGPE
MKRYMIKNKFIGRLLDIYNYKVTENKHYARDLSYLPTFERVCNEHKDSRSSKNINYDEDSIDISNYEFDFG
LRYIKQKLSKDILIVEKEKKNKETKNSDDDDYSDLAVLRGNKEGDEKRFAYLLRTISLLVCSCKFKLSGSY
IAEDSKFLIIPEPFTVSDSEERVINEICSEDMMNDLIVRSNLNISTKEDINNMYAHLCWENDDVSDRLLKLLI
SDICNAESAFITIIRHTPLVVNIAKINDRLSATRMVYLIQNLFEKCFINDYKTYIKDSDSLLNMVLMIIRINYD
ACSYMRDLEDPMEHIQKFYNKNPIPRSYGSSQVLFRGISGNEKVERFLLDEDKKTLTNYADYRLQVFKDL
LEDDDWENRIKDFVKRELPFFDKDVIFSKGEHLDYYKDEYKYWIEAQVIEDFGPILQVSYRIPPAIVYIERC
DDKKSSVKINKDSIRNAGVYTGDMYYQINSLYEFIYIKICEKERSYRK

>CUFF.4455.1 hypothetical protein
MFGSLYDDEPDDGLKIGDLPFASASKSYRYRPDNCASLLEMPEKIRDPREFVGLKNQGATCYMNSFIQTL
YMIHDFRNAIFKMPLCVGDPLTPSGFLEGQKYQILYSIQKLFVQMKAGAKIAESTKELTDSFGWKDNQQM
VQHDVQELIREFLSVLERALKGTSYETLISDLFSGIKSDILQVPEHGICRARDEHFADIIVQVRCCGDLEASL
SNYFNFEFLTGDNQYYCEEVDAKVDALKGVQIRKFPKILTISLLRFDFDFEKMTLAKLNEKFKFPLELHLN
QYLTDDSDQKGEDIDYELLSCIIHIGNVGGGHYKAYIRDYANEGTWDFDLYDREEEKKENKKDQGVGTS
DDAEDKKDDSKEDDKIDYNGDFPLPYKNPELGKNWYEFNDSFVRPIVAGRLESQFGNGSSNQNAYILIYK
RKNDKDVVEYENIPDYWVDEIMKQNEASKTERLAYDFEANHLEIYLQPQAIFEIQEGVQLKYIDLKDTEE
QGVKIKFTFDNTISELKAKIRESLSFGSDSVFDAFEVCKLNNDLCQVFRSISEFNDEIIKQSQVTHLSTWMI
TTDQNIISYMNSIKGIDFEPIYINYRFNEIESRVIMYKNMTCGDFIQKTAEVFGYDSWKKVKCTYFVDGVM
RRLDRLKYDVEKQRDHTLKSLNLKDCAQVVVEEKSEEEIKIEEQNKNMQADFIDPNSSSTPTTTETYINVD
ETENIRSVLMHKEEDPEFLERFNVDLNWTISELTEKLKQYMGIKQDEERRLRREFDNSLIVKEELSIKLFDY
PEFREGGVRLKMEYGRFPSVEELALSVALYNEKKLRLRFYLKKESTILEGKEKICKEFGVDPHKYRLWRT
DFEEEPQYIG

>CUFF.4753.1 Ubiquitin carboxyl-terminal hydrolase
MHQVELMQNKSKSGKEKPYQKLRNVNKEEQKEEVEKMWREMENSKMDIEEDSKWYIISMEWFKQWK
AWSGFNLSLKSNDSDSTKFPIEESETATDKSVDEPGRIDSIDILNANEIMLFGEYNLKDNLVEEQDFVIVNP
DIWRYLYSIYDGNPILRTAIKNIDSKSGGDDSDCIIEVNQVKLYIFEVPRENKQDYYEVMLASRNWQMSEV
KFRICNKKKIKESDIRIWKVEKPADLEKFYCELEYEWKKYKSLRIDGMLIKDLSILVKDANFSRDDFLMIE
YPIPTSNDNGYALVEIERKDMHESLNEKAAKALREDESLKEALSNPKTLDFIKIPINLVVNEDSVIGACGLS
NLGNTCFMNSALQCMSNTIELSKYFLFNIHDTEINHTNVLGSKGRVAEAYGDLMKEMWIGDKRKTAPFN
VKKSIGTVVAQFRGYNQQDSHEFLHYLIDTLNEDLNRIKEKPYIEVPDSNGREDSIVSKEQWDAFTKRNDS
VLIDLFYGQLKSRLICLECDNISNTFDPYSILSIPVPVVKIVKLKVTFIPHDMSKHKGIMNIEININDNLCVTE
LEKMIQNEIKSECELLFYIYDSNKVGKRIKKNSFVCRELVGMNIGAFSYEIDKSKPSSNLYILDIYMKRQAK
KMLFFSGEDSVCLPFVMIIDSKSTCRDIKLQIFKFLYPIINFPKSIRDKVASIDSLDEKIEAAFVITFEDSNFGD
EELYELQLINNRESSEGCPSCRKPHKGPCKFDFTQKSYKAFLNHCHNDPELCILWKMNTTTDLSCFERPEK

RVIGEETVKS**KN**KQLDLDCCMDSFRQEEILDGDNKWYCGKCKDHVKARKKMDLYKLPPILIIQLKRFLK
NDHEYSFFRNASRKITDLVEFPLKSLDMSEYLINEEAKEEKLIYDLFGVSNHMGKLHGGHYTASCYNQEL
DKWLYFDDTSVSKMSEKEVIDPAAYILFYRRRRS

>CUFF.6394.1 cullin c
MLMNFIRILLDETFENDFLKKLIQLWSSHDTAMKMVRDINMYLDRNFVVKEKQKDIYTMGHILFKKYVL
RQEQVRTRFLNLTLGKINLERNGEKIERDEVRGAIKILLELGFGNNNVYKKDFE**KN**FLDKAGEFYKNEAQ
DKVTTHSVPEYLKVVQKRLDEENERCLDYLIEETKSPLIKKVLGPMVEEYAKVLIFKQESGLGTMIRLKQ
YQIIHLMYKIFNQVPTARQEFEKFIVETVTEDCNNIIQDKTLTEDPKEFIEKCIETKQKYNAIISQSCEKDVD
VALAIRKAFESSLVEFQSSALFLAKYIDLKLKKEIKSLKDEEINTLFDKIIEIFKLLGDKDQFEGYFRNHLTK
RLLNSLSTNDEAEKLMISKLKVECGCLYTQKLETMMKDMALSEGLNSQFKHSGYSHDLKFGFNIKVLTS
GNWSNDSQTTHCNIPKSIKLAIENFTDFYMSNHSGRVLTWKMNFGNADLLGFFSERNYELTVSGYQMAV
LLLFNESDKLTVSQLKTLTGISPEYEFKRHVLSLIKAKILLKNTKEFDLLDTDQLKVNDGFKNKLHKLKVP
LLNQKDQIEIDKKEVEPKIEDDRKHLIEATIVRVMKAR**KK**LEHNQLISEVMKILSSIFQPTSIMIKQKIEGLIE
KDYMMRDPEDRRVYLYRA

>CUFF.8294.1 Chaperone protein DnaJ
MFLCKVPCRCKLAKLTAINRSAYILQQFTYRSFMNLNSKVKRNRINAHCLLFNNINKMGFKATNILLQKD
FYKILGVNKGATKDEI**KK**AYFSNAKKFHPDVNKSPDAKEKFAEINSAYETLGDEQKRKIYDQTGMTGDE
QAQAGGGFEGGFPGGFPGGFPGGAGGFWENFAGANNSNQGGMPGGGSFRDIFEDFESFFNMGQSKKGD
PRSSASVKGKDVALNIEIEFMEAVNGTQKSVTYNKIDNCSRCNGTGAQPGTGETNCPTCGGTGFQTMRQ
GSLLFQTSCQSCGGAGKVIRNPCLSCQGHGSVQSRATETVNIPKGVDNGVNLRMNGKGNTSKNGPAGDL
MIKITVKEHPYFKRNGFDVHTDKYITVSQAILGGETIINTLAGDVKVSINPGTQHNDKKRLVNCGINKLPP
NHRQKGDHYVNFKVEIPKSLSAKQKEAILKYAEVEDKVMEH

>CUFF.8375.1 kynurenine-oxoglutarate transaminase
MEQTIYKKQTKVEDLLNIWTELAEIGDKYGAVSLGEGTPYLQPPEFLIQNLIDAIREGYNQYTSSYGHPDA
RKLIAEFYSPKFNRIIDPNGEILITNGANGSMDCLLQSLISDEKDEIIFIEPMFPQYIGQAKFAKATLRFVPLD
LKEDNKWYLDIDTLKATLNENSRVIVFNFPQNPTGKVFSSEEINEISEILENFPNCFVLCDDVYDFLTFDDH
DHHMFANIKDNW**KK**TVTIYSGGKLLCCTGWKIGWAIGPREIMRQAVVINDTCTYCHNVPGQIAVARSLK
TAYNNSYKDDLTYVEYVKNDLKKNHDLLLEGLKDVDLPINPVPASGGYFIFVDVTGLREIIPDKYFKQEE
YEDDKHTIIEKNDYGDPVPLDLAVSRWLAIEKKVVTLPGTFFYDRNSTTKSDKFIRMAFCRGEKIINQ

>CUFF.8992.1 Peptidase putative
MELDGFNLIQHVTPNNELLKNQIVQAIQKNSYYSHNFYDYDSSEFLVTNPQDNPCRTVLAFKCFGAKEIL
ANGGEDLLKENYKDFYAVDGHPGYDVALEVKPEDKPQKIKTPSDATEEEKTKIKEDNAEIKKKVEEFAG
QVGEKWCRFKSDFMGAPIRKALKEVLEDSKETYTVEIPYR**KN**DKYWIKKGENNAVFYFAVHFTDKTDM
ALARIFCNELKDSKKAISQAVSVNYFQKIDPSIDVLSELKVNEKYASCGIVSFALSSVHVKKNLETSVYFLV
SFRQYLEYHIRMAKCLLRTKLRKRIAKFEIIFEKSCREGALKSVEYKTTHGGEKKSDKVEEEKIAEFKKKQ

>CUFF.9901.1 morn repeat protein
MAKKPNEPAPQEEEEKIEEVPQLPQEGSGKFEYINGTQYEGEWKVFNNIKMKHGKGRIIHGNSTTDTYGN
EIYEGDWQQDLMHGDGTYKFTSGAIYVGQWIKGKRHGKGRIDYPDGSSYEGEWENDEMHGDGRYIDN
AGIIWDGIFVNDGYESKIQKKLQTERKVILRKKLHEEAAVSFF**KK**FFSAFQASDKKTMKDNLAPSFIRPEE

LQNYYKEPYPKYEDRTPDAWNDVFHKIIDGDEYNCSASSGPGDIHFLDPQRILIEQLTDDHGGQIVEFWRQVEAKTYCVALCELSNENWTIIHYQEKAN

>CUFF.10208.1 elongation factor 1-gamma
MTGFNYELIRIKPEEKQDFRKDVNPAGTFPYLHNAKTGEGIGESMAIARLMCYSNLESGLYGSTIYETGIIDEIIERHLYAYNQTIFKIVPSVLGYGPISKKDYSEFS**KK**MKEYFTLIDKLLKDKNYFVGDKITLADIYVAVSMNLLMATLIDEEYRKELPNLTAWYERVRNNEHIISVLGKPR

>CUFF.10375.1 UNKNOWN
KTNKKLLANCFTYSLYQRNFCTVQSSKSNQELSNEFFKFIRRKEYDSLSETHYNAVVNQLESKDRSSISKVVTLIESSDVVHRLKADELFKRIFKSYKKSHDNENLDKQLPTFRIGICGAPGSGKSSLIEKVGMSLIRKGLNVAVLAIDPSSSRSGGSILGDKTRMDELSREVNAFIRPSPTKGYLGGVSLNTHEIVSLCEHVGFDAVLIETVGVGQSEIEIDNVADFIVYVVPPALGDELQAS**KK**GVMEIADCVIVNKYDSEFVKSCRIAKFQIQSALHLSRPKVDGWVVPVELVSAHSNINIDSIWDNAMKFKECQKDYIVDKRGKQLLHGLWAYLGDMLLKKLKEDSDHKYANIIQKAESRLVNQEITPN

>CUFF.10414.1 zinc-binding dehydrogenase family protein
MEPDNIDEEIIDMEEDILMDDDNEDEFEFSCYGQTNEEDDKFDTFVGCLQEIVISNEFESMQSSFFQ**KN**CMHFENTEENKLIYMEIFKRYKEIIENYIEERLAEAIDGFDMSEYAVLLPDRKDEIDDQLLELLTSFADFGTFKELMISYKKMVIATTPKHKSAKAAQLEKELLEKQNIEVPEGLELLHISGNKTTFHEGDD

>CUFF.10660.1 unnamed protein product
MQYNHLFKIVIIGDSNVGKSCLLIRFADDCFTENYITTIGVDFRFRTLQVAQ**KN**VKLQIWDTAGQERYRTITNAYYRGADAIVLVADCTDMKSLEDIPEWLQEVSKYIPEDTYKILLVNKSDVAEEEKVINQEIMLKFYEETGIPVLETSAKTGTNVDEAFIIITKELLKKKSEERSKPSTKIGAGNMLSYESIKRKYEDNCCGG

>CUFF.1130.1 hypothetical protein OXYTRI_17154
MDAKEVGLVKEVLLDLLSPNNDIRGDAEKKLDALKANSPDKYMVYLIEGIRDTDLKEDVRVMAGVILRRNMCPADPLEPTIWMLLSPETRVYVKEQVLTLFKTEESKTIINKIAELAAEIAVRINDVDRTDIWPDLFILSKELIAKGSDIQAEAGLNVYTEAFRSMVNELVENDPDLIEMFKVTLEHNNLKIALSSLQAVSQLLCVVQPKYAKLFLSLLEPMVRVPLKALEAEDESILEDALIEFNAMAEAEPKFF**KK**NFGDLFEIFKQIISKSDILNNTIRHQPLEFLTTIAERNPSMLKENEQYLKDLLDTVFKLMIDIDNDIDPQWENPKDPAQIKEEVDEDPVVFGKEVIDRLCSSVGEDTMLPLICILVENTMLNEDDWRFKNAGLSAFSQIAEYVENIDQIKTMVPTVVDHVKHPHPKVRHSAIHCLGQFSTDLKQQFTENFHETVVPALYEAMNDSVMRVKAHAAGALSNFLEKSNNEIGMNYCEKLLEKLLELSKSDSSYCAGNAVICISSLAESCQEEFGPYYEIIFKEFLPILIKPVSKEFLKFKGQLIESICISGVCVGMEIFRPYSKTLIEALLVIQKEQLGKEGDPQRKYLLAAWQRLCLLMEKEFAQYLPEIIPEIFKMATLQPSLKVAGSGEDIIEFLTEVRTTSGAKGVTVSTDELDEKNVGIHMLCVIIDELEELYAPYVEQTSSLFLSLLTFSYNATIRNSVADSLPTLLKAIKATEENREIVLSYAQNYIQGLFEAMRRESDTDTMQHQVSGIKRCVETMGEFLDESQVNSMCEIFFNAIHKSDQRKDLNFKYAEENEQEEDDVDNQNKQFMEEENEMEDDLQLTISEAFGTLFKTHKNQCSELLSNLFTSLLPEYLDDNAPFIKQKFGLYIVVDLVEHLGLEHLGEKIDDCLKVIEKYAESINPVCRQAGVYGLGVFAKNSGDYFSSFADTVVEKLKKAIEMKKGSQDKVEYGHAKDNAVSALAKVLKHQYNHINLEVTFKFWLSQLPLKNDLTEAKECNEFLAEVIEFKPEMVIGEKGEYMDNLITLLKKTMKKDYMKPETIEKFKSFLKKHKCLNKV

>CUFF.13256.1 UNKNOWN
MNCFRRSVRRFCNKHKSPPNLVQLMPSYQEALKLAVDKKYDESLSKFNKLMDEITKEHSTNTPYHVFVL
YKIASVNNISGKIADNEAVFEQINEISPMAYPNKPSMIFMCHSTLLKYYLHFDVDKCCKYGEELQKNKYF
DLEELPLFERNDYNHTLGTAYSLEGTSHE**KS**FDCYNKCLKLAEEINKEAETTHYKVSKGHALNNLGISKF
WYFMEKTREMGELKESDQNKSDLTKNIAILEEGIKDLKNSVLEFEDFGNRITSNLKQNEKLSEIQLKQKLF
VDEFFNTEIKETLSQDFKHYDLHDNHMNSKFIQELFTKAESYLPIANLGEVSLIFNKMKEALAFLDVSLKI
MTEKDPHNLLRNKIISDLSVLVDLMGQTEMTIKMNNTLLNGLREVDDYVKVFVLRNYGHILSRHKQHLE
QAKKHMHDADELDKKYPYWSERKMCLFTPLL

>CUFF.1331.1 Rab11b
MESTDGTYDQLFKIVLIGDSGVGKSCLVSRYVKGIFPKNKVATIGVEFAAKNLKLKSGTRVKAQIWDTAG
QERYLAITTAHYRRAIGAFVVYDIC**KK**ESFHNCEKWINDVKSQADPEITIMLVGNKVDKATSTTREVSTQ
EGEEYASKFKIMFCETSALSDTNVTSAFEELMNKIDEVKLKNPSKGSSSKGYGLKSNNMQEFEDQEDSGC
KC

>CUFF.13339.1 hypothetical protein OXYTRI_18109
MCFLSNSIDKEEMNKISDDLFSKKYMEKMTSCPKCKEKFLFDVGSIDNEKDEKGNAVSKEAAECKAKYR
LKCPKCNTIACICCNAIPYHDFKTCEQFGEDQLKIKCIFCKCDILEMSVAAADKNFATCSKPECLKLKESRC
KHKLKCGHYCLGSIKSKNHLPCLNEKCSKLNESITNGTVSTDYCPICYCEALEDKPVVKLGCNHFFHEDC
LTNKLN**KK**WDPPKVCFTYLNCSSCNTALKVDHSELEDIIDEDRKIEAEIIEISLKEARKEGLDKLNYSGPPF
NGNFEAYAMDQIVVYQCYECKKYYPAGMKDCRREAKKAEELICGECTFLIIGATQGVDSCDTHGRDGII
YKCKFCCGIATFFCWGSTHFCTPCHDLQNQGKFLDSKKPEEFPVCKGRKEC

>CUFF.13781.1 B-box zinc finger family protein
MARHSTVDIREAFSDAIREFTSDDVNINKKKHLITTNILKIEDKMKELKNNSKEVEEQIYELI**KK**TLKQLSD
EYSKKMYILRSDLLELRRQNDEIHFVQEYAKNQTMCMKPVSLLKIWTSYKNYRKKLQEQRITISDIQTNL
KLDGKPFITSDQAHRNVDIENLTSEQKNVLDTTRRSIVNQYAEKSTAFRSKLINRAGKFGMKEFNPFSGSA
LAKPSLVPTAIGKKSTAFGDPNRDFIEKEQKNFEDKSKVRNKIEDLLVKFEKNLLNSKQNKVDSIASEAVR
RSLFGVFAENYQNFKGLLSDTVKDDIF**KK**VFQGSKILSDVYRIILYFNLPLKSYTEPIIPTKIFPVSEDRTIKT
MLEAFEKKGLTHTQPNVILMKVDKDLVVGGYASHGWSISEKKRGDSSCFLFNIKQNFRFEAVPGRSHYQ
ETISKDGIKFGDKDLVIENDFKRVTCEISGEYFVFGTHLLQNKLDSLIPDTKQFEPEMVEVWSLVK

>CUFF.1452.1 hypothetical protein LRAMOSA07404
MEYSDGECDYMIKIILIGDSGVGKTSLVSRFNNPDPR**KK**IKTNSTIGYDYCSKFFEQDDKLIQVHLWDTTG
QEKYRSVIKNYYRRAMGAFLVYDITKQETFDGIANWMEELKENCETNCEIMLVGNKFDLES

>CUFF.15237.1 Acylamino-acid-releasing enzyme
METTNSKNDIAFQNALDIFETLNQNFPSTTAANVIGKKYNEDGELKSLIVKIFFTKTNLQKFETRTHSNIYS
VDVETGVSTQLNDIPIDEPVDKSIVVKTENKGQRIIIRKDTKNEKQLYLEHWDQDGLSTSLKLNNVSKIHN
NSVFGGISWSKNLDKIVFIAEREEISDYSPYWDSENKKAKDSNANTEEEKKSIPVYDKFRYNHKNSNIHSN
YGETLADLKYTVIVVYDLAK**KK**STILNLMKMKEDKKIDSDVFDDVHPAHPLFDESGNGIIFHGYYLPIDK
LGLVFCINRPTKLYYIKEYEEPENSKIDKGELKISEEESKEGQLKFKYVVNILTPEYYFSGFAKFSDDYKYL
AFFSSKEEFHTHCTSVALSVIEDFDTPEMAIKAVIEREHSPSESFSGVYGKSDEMQGCKFIKNTHSLLLSTIN
KGKEISLLVDFSTKAYKLIKHPDLLENAAFGVFLIYEDIVLYKTDSINERPKVILVKGITTETPKFINVAQVD

TQNKKNEKCISINEHLNSIKIDTFSLENESEGYFVRTQNNDTGESQNLKRPTILVIHGGPHFWSLKNTYLKS
RALWLCLGYNLCIVNYRGSGGYGLKFMESLSGHVFDYDISDTLNLFSICLEKFSDEIDDTKLGVYGGSHG
GYLSCSIISHPDWATKFKAACIWNPVTAMNMSTMVSDIPDWHWSVALANTNQWHYSRDDIIEMFDKSPI
NRTSNVKTPSLFIIGESDIRVPKYAGIQFYRAIKENGVETELMYYPGQGHAVEAVEEGIDALISMTKWFIDH
F

>CUFF.15242.1 calcium-translocating p-type pmca-type family protein
MMMFEENFEKKSNDNKQSEESYDSPDPFLLASTLVFAGRMKALVCAVGNNTYVRSRNLHSPYYTMMGE
YTTVLQSKIDRIVNSIGRYGFIFCVIALLCIIFRLCWMRISEDTSSVFLNNLSFFCALFYSLIYFLVLLLILIPTS
LAKAVDCVIVYSIIRLSGENVICLTKTAPENMAKINQLCIEKGGCLTSQKMEVLHIDTLDGVHQKLDTIDSI
KREIITMNLSINVVHLNSVGRIVKNLNNENKHFGNRMECAFLEFLTDNGVDYEKIRMDAKEILQTNPSIEA
RMVMTVIEHPERNGYLRILINGSSDVFLPHCNEVLLNNGEKELFTYSKKEEIKNNLILNYKKKRLRVFALG
YKDVKKEDFDQMISDNKKDHEFIEKQYVLVGLIVLDDPPRGQEVTEAISDCKNAGINIKIVTGDSSEAAKA
IAKETSILCNFDEIYARNIVLEGPDFRNKIGGIVTHSKKTRWGKDYI**KN**EPSFREIIKDLKIISLATSEDKYML
VTGLKNEGFVVGVTGSSPADTKALLKADVGFSMNELGSEVAKHSSDIVLKNDSLKSVVSAIMWGRNLYI
SIKRYTQFYITFVIVLTMTVLLSTIIEGHIPFNLFQLLWLYLVTEVLGMFSLICDRPTRQNLLENYITQEKRIIT
ASMWRSIIMNSIYQSVVLWLVHHEGDVLLSSGSIFVLQDLEETNGVIFTLLFQIFFYFQICNILLSRNIKSYEF
NFMAGAWTFTLFIIVLMIIIQIMLVNYGGALVKFIKLDIYLHIITFIIGFTPLVWGTFVKCCIPLKFFKLKINEN
PLDRSDTVKCIQHYFRKIVHAPSKTPVKQKWRSNSQNQYDTKGKKIISWGDDEETGIKNPDQKKGENMV
EKLKD

>CUFF.16329.1 NADH-cytochrome b5 reductase
MILITLCLNCQNKDNEFGLHVGGHVLFKATFPTSEHPEGELVTRKYTPISQVHEKGVVKFPIKIYRRNVHP
KFPEGGKMTQYLETLKIGDKVEIEGPRGSLEYLGKGNFKINDKICKDLFIE**YR**MYLFLTLVKKKKLGMSS
GGTGITPVFQVIQSILLNDDDLEITLLFGNRSEDDILLKNELDEFARKYPNKFKIVYIIDKASKPDEWKGYT
GYVTKEILQECMPAPSEDTLILHCGPKPMNIAIRKIYCEELGYDSETMYHKF

>CUFF.16572.1 tubulin-tyrosine ligase family protein
MSLRDCDIDIIRSKPRLFFNRYPGSELLAR**KK**ILCNIMNRMEKYFGEEFEYTPLSYMLPEEEDLLTEDMIKY
KDMWYIAKPSKGRGGDGIFLVNRIADIPRWHASSELLVQHYVTDPLLVDNKKFDLRVYVLIKGLDPLECY
FCTEGMARLCTESYKIPDRGNRRNRYMHLTNFSVNKNSAKYVKGDDETGGTNGNKRLLSKLLNLLEKE
QGIDPNRVLNQIKDTIAKTIIGLIPYLSDFARISINPNIDQLRVFQLFGFDILIDKKLKAWLLEINANPSLNMYI
DKELPNGDLERTLCDLDKYLKSMIMQDAISIVKSKHSPDQYGCFERILPSNDTFYEQFYIWEESRKLFEKL
GGVKSPEFISSSQFQRLSKLRGMTNGKIVKGSYDIMYKNVVNRSDSKLMAMEHFFDALEILASKLFKSETL
YDSLNNLICCVKKQI

>CUFF.17415.1 hypothetical protein H310_02276
MNLATLISKQKDAIRLVIKNATLYRQGLVKEFNIKNSLNAVCSDEEFKSDEHLDENLNEWIKKIQNQYCEE
QRYYHTLTHVASMLYFYCANYELTKLKTCNEFEYSAAMFCIFHDVVYDPKAKDNELQSISEFHEFIKDFP
SELETLDEFVSECIRDTIKHQKHLDDDSDLSVLNGFLLDCDLAVLSGGYDAESIEKIKESDSLSEIYSTYA**K**
NIREEYIHFDDDSYCKGRASVMNTFLSKDSIYFTEGVRCLYEKVARENIAKEIDLLKNKSI

>CUFF.17975.1 Putative ABC transporter

MDAGVLCISCCMFFVCFHLVGQKVSYEIRWRYMKSILTKDTEWFEDRSLEELPTKVHTNLNEVENSSGKT
IGFIIYSISACISGVVYGLLLGAVFCCCILFSPVIVMMIGGLNMFAI**KK**ANEQEENNFIRTGADIEQALNAIKI
IKAFGQEKYETDKFIKHLEVDTNIKKKYTFLYAFSVGIIETVIYYGLLSTFIIGGAFVIGGVHNGNFDRNYR
MGDCIGSFWCIQYGTYCFGNSFLNLEALQKGIDALKSIMVVIDHTPKINIDEEDLSSISSIDSVEYKNVSFQY
KSRDKPALCGINFKISKGKITAFVGESGSGKSTLVKLISRLYDPTDGEILINDQDLKSINLRQYRRKISYVCQ
EPSLFNESIKENLLNGNPHASDSEIEEVLKTCMAYDFIQQLPEGINTNFGEIGGILSGGQKQRIALARAVLRK
PDLLILDEATSALDSKSEKKVQEAIENVNKEYKMTTVVIAHRLNTIMNADTIYVLDKGQIIEEGTHNNLIRS
NKTYTEYFNSQQSAIEKLRIVNENQNIKEEPDDAISYENKNLIETNKQNSENTESVMSTADNSFKLFELTKP
KKYVFFSFIGAIYVGCSMSLLSIPYGKEALNLVLKESDHEKWKAVWYSLIIIGSMTISVLVFQTEARYYSR
RTTDLLSIKMRSETFKTLIKQPVQFFDSKNNSIESLIRILASDIRYLNGNSVEYYLLIVQGISALICGLIVAFCY
SWKLALLALPIAPFLSIGFAIQYQLQFTPPNKSKSSKKKQLSMVSDCIMNYQTIASLAYEDEYLSKYEMKS
SHDNFRTGLFNSEYIKIGNALYVSIIYGLSQATLPITYFICNMSMAERIEAGKSQSDQFTAFSAIIFGSFVLSN
GMINSPNFAKGKRVASKILSVIRNPKEGSKESSIVDGTQTLSKEQASQDIKFHNIWFKYPLENSKWVLRNF
NFTIKGGQKVGLIGESGSG

>CUFF.18289.1 hypothetical protein OXYTRI_07482
MRINSNESYILYADKVYTTLCFKDIHYKSYRPYGEIDTEHLMYYIDKKSAIFDYMDNFDNLSKFLSFFEND
PLYYCMIVTQNNDEKTPLDIVIENNSVKMIELMLNMLVKLDCFSFSRVLYTKFPTLFGMNLKSFEKYLNT
CYFVTFQMKSINKINLSKDETIREMHSCCILDKDFYHKYKIG**KN**DENLKLKENQVAPVDKFRSSEDVMNL
REAKENEEKQLKRVSIKGIEFDWIFDTPDGASFLKELSETSNINIFAQNIIKDIVYFQWSYFQLNIILFLMIPYI
IYFVSFCVFATYIV**KN**QVNESSNSQPYHISAWVVGIIILLFNIGWGYVELLQIIFYKFSYFKSFWNLLDLSSII
LNVAVVIMEFAEADFVQINRVSSVSVLILYFKLFYFLRIFSSTAYLVRMIIEIVKDMKFFVSVLMIATMAFA
NAFYILGRNSSDDAGNLAGSKITDAFIFAYRMGIGDFQTGGFGTRDEEILWIIFLFNTILVLVVLLNLVIAIM
GDTFDRVQETQEYSKLKEITQMIRENEFLFSRKRNFKKAKYIVVIEPEQAEGSGNTTWEGKLNQLKSFIEQ
SSEDHICNLRKLQAKIDNIATSALEDKLKPVEDKINNKFIAYDHKLEKIKGRLESLYDLIEKDKLKKKE

>CUFF.18401.1 fatty-acid amide hydrolase
MSILSTSNIFLVILLFLLVKLAIFFLNRAKNRQQKEAVLLMGEKLRKRRDDKVSKVLEKYKDKWPNKEKM
DIIIKKYRTLTSLRKAMDSGELTSTELVLTYVVYRAATEGMELNALADVMFEEAIQEATECDKLLKEGKTK
GFLHGIPVSFKEQIIIKNTVCTLGICSMSENVFNEDGIVAELIRKNGGIPFTKTNVPQLLVMCESMNRIFGITK
NPHNKERTAGGSSGGEGSLIGSNCSPIGIGTDSGGSIRIPAHYCGIYGFKPTMKRMTKMGVKETCDWQLSD
GVSEIAGTTGPLGHCIDDLVEIMKIFYSKDLFIKDSLIPSIPFNLDLY**KK**TLKRSSTIRLGYFCYGGLFYPCKT
VRRAIEMAVESCKEVGIEMIEINPVKMFGFIEGFGKTTFGGDRLEKLLKNEPYIIEDDISHSIKYIPKILKNIM
IFIMEKINMKREAIFLKVMGDSSIEHYSQGILNLREFSQNFDKIISENNLDGFICPSNLLPAFRHYESKEVAIS
VVINVISNIVDYPSTVIPVTKVTKEDLSEIYDDPFFPNDHFVQETRKVLVGSEGLPVGIQVCTRSFKDEECL
GIAKIIDDAIKKY

>CUFF.19493.1 transducin beta-like protein 3
MADAVVVDPAILSNIQFFNKSEQQPDASVIKEDKVKISKTWKPERTISAVCSGGNFEFSTDGEIAYSLHDG
KVFACKTENMNVEKTVEFENEYIMTFTVYKKLMVTTGKNGGLRLWNLDTSTCIKYIGTGSSVILDMKFD
PSGRYLACGTADRKVKIFDILKDKLTHDFIGHKLSVSLVRWLPCKSQSKNKFIVYSASEEGVIKVWDLVL
NSCLGTLQYHHSQIPCITFTNDLKTLIVSSRDQKLSFWSLKDNTFTKIGALQLNEDVEGMQYVNLQISKTK
TAPYLITGGTDGKLKILDINNQKYIYEEEDPLKQEIEKVFYLQKESQILALTNDQVLTYYSLLINESTECPQL
KRLYSLCLYNDEIIDLKFLKCLDDHIVMCSNSELAKVVDLKTQ**KS**KLLTGHEDIIIAVDTFGDYFVTGSKD

MSCRVWKLVNFNEYESSFVCLAILKGHNMSITSLCIEPKKGSYFISSGQDNTIKKWSLSDIVNKDYLKNPS
EFPILINTAQASEVAHLKYINVVRISPGEKNKLVATASHDRLIKIWSASNFELKATLKGHKRGVWDVEFSP
YERILASASSDKTIKLWNVSNGQCLNTLEGHLNSTVKVLWVNSGLQIFSAGSDGTCKIWNVKKSTCINTF
EEHDDKIWAMDAYVDKILTGGSDSKLIEWRDVTQEVEDEEYKEKAEKSKEEHLLSSMIYEGRFKDAAIQ
AFKLKKNRDLFSVIEMILDQTKEHTNDNDPVLVVLNNNKRFHSQFQGTKIDEIKASIHSPSEIAIKEIVQEMI
KIDSIRLLEMIRDLNVHQKYCKIAQILLFQIFKVFGLSKFIDFIEKQFKDKNEELKLKDPKNYIPAGRKEIISK
IKNYLTIIDFYSKKHIDRVQRNQKLSYYVSFVLSKYTVDQEKLELKNQFDQNAYKKQTKRKRELKADR

>CUFF.1984.1 glutathione s-transferase domain-containing protein
MDKSNKDSKIQLYVDEASQPSRACMIFCELNNIPYEKIPVII**FK**GGHLSEDFQKINPMQQVPAMKDGDFILS
QSHAILKYLHASRNCPDHWYPTDPKKRAKVDLYLDWHHNFLRQGAGSFIFKTIFAPSVGMVFSEAEIKTA
RSLLTKSLKIIDSVFLAETKYLCGDEITIADLSCFCELTQLGFIEEDISKYPNMNKWYNTILEIKEVQKVSEF
VHEAIKKSLQRRKAKL

>CUFF.20034.1 calpain family cysteine protease containing protein
MDNERLKGVIRKVDYQKIISDFDKLSRSKPSNGEEGPQKFTDAIFKPTQTSLYDPKFTQLDTETLNLWKKF
VWKRPSEIFKGVYKVFENEVDMGDIKQGSLGNCYFLSSLAALSLIPDRIKSLFVTTDVNAAGIYVVKFLIY
GKSYNIVVDDYFPYDPKTRRPAFTQTKGNEIWVLILEKAWAKINGSYENTISGLTNDALSFLISAPSTVIDH
CYPKYNEDDMWTLIDNCENNHHIICGSSQSNEDNKDVESHGIVSLHAYTISELVEFTTSSEGRVRLVRMR
NPWGHKEWDGDWSDKSSKWNEKLR**KK**LNYYEGDDGEFFIKYEDYLTYYRTTSITHFDQNYKYQCIQVK
QPKSSYNIIEVEFDVKTHLYVYIHQPNPRLMQKAYQHLPPSPSRIIVAKVLKENNEGIREFEFLDGIFSYELS
TVWDSKETLLPGRYAFFIEVEWTDDAKVREYTACTYSQNDVLVEMGQKGGKNINFIESAMMSCAIKRTE
KFTFKDYGQPNILRYMSLKDSNSDYG

>CUFF.21141.1 Oxysterol binding family protein
MHVSIFKEDVGVDPIDGDNPDIYSSVESVGPYSFVDCRQDYDKKCPPPTYMKYEDVNCFLPIEKDQIFEKF
EFLEKGGLKCTDEEAMERQKGVLKDVIKEFAKNFIKGLGISHMSLPVRMFEARSTIQRVADYFCFAPIFLK
RAAKCKDKIERFKNIVAYSIAGMQVCTGQLKPFNPILGETLQSTLPDGSKVYCEHISHHPPITAYLLEDKD
GDYKFWGASEFTASLGTNSMKAGQEGDNYLLFKDGQKIKTRAPHYTLGGTVMGDRTINADGYFLFEDIE
NNIKCIIIFNPIMKTGGIFSSHKYAGKTDDFKGLIYKPSSKKKDKE**KK**FDKFKHIHEDAEEIYAEIEGSWLRN
LVIDGIEIWDIDDPSMRPQRHIPNRTCLPSDWRYREDLIYLYRNNMKMADKWKVRLEVQQRLDRKNRKE
IEKKRKKGRKFLSLE

>CUFF.21189.1 pyruvate kinase
MDKADIDKGALERVVDPNQKTGFVVATLGKSCKKPETLINMIDNGMNIVRLRLCQEERKKQVIMLDYLK
EAFTHKPH**KK**CHLMVDIRGRDITIGSFDMKQPYIEVESGEDMEIFIDIEGDIKSTSKRLVTSEIGIDRIIQKGD
IIYLGDGAVKASVLAVEDGITQLRTKSEGRIYEYSSIIVPDKHSSLSVIQEQDITDLKYLNQKHKIDYLCVPY
ANSDEDIKVVRRLLPFLDQVVILARLEDKNATILNEADGVIIQRRSLGLSIVSEKLFALQNFLIEQCKLKAK
PVILANEIIESMIDDKKALRSEIADIQNSLVQGADGILLDKETSYGKYPIDCVGMVSKTIAETNNIIDPYKKF
KTLHSICDFTDKDEVLVMNAVKIVLNKNREPIDYILTLSKQGRIARILAKYYLPIDILACCPDSKIVKQLNL
VTGIKSMKVPNYTSKLLGPDHLIQIILRTNRSMGLGTPGHWVI

>CUFF.21195.1 hypothetical protein OXYTRI_19162

MLVTAYNFTLNHSLPQGLVTINKFDGINPALASCTTGGKILVHTPHSKDTSDPTSSMKGKDVQYLNVNKD
VVCLTGGKLNNSIDRDILLIGSKTNLLAYDINENSDVFDKEVQDGINCMIFGEDLNPDLPLVITGGNLSLTG
FDYTAEEQFWTVTGDVANCMAILDFDFDQQKELIVGSDDYSIRVYKNEEMIFDISESAKVSLLSKIQKAAF
AFALSNGTIGVYSGSAQKWNSKVDNKITSLLGVDFEMNGSQQVMIGYQSGKFEVRNSTSGEVTYKSNMS
STVSKIMYEDYRLEGYPQVIVCNTDGDIKGFNLTLNTEAYELNDVAQ**KK**DVEAINKMNMEIYALKAELE
DNTVESRK

>CUFF.21282.1 nucleolar rna helicase 2
MESSDVFQRNKPKVDETEEVINSQDLMVGLKKENEEAKGEQENESNFDQFVEITDKSREDLVSREITSLFP
IQVACFKDVYDNKDMIARDLTGSGKTLAFCLPLVERFRKLGYFKQTKTYVRNLYTIIMAPTRELAIQITNE
LKKLMHRENEYKVLTVYGGVPIDTQTKELRYGVEFFVGTCGRVLDHIERGNIEFSNLKTVILDEADQMLN
LGFQEDIEKIMSSVMEKVKEKPQFLMFSATIPEWLKNVA**KK**YLNKDYKTIDLVKNLKNKTSNTVNHLAL
NCPFQNKIPVLADVLRCYGGLKGKAIVFAQTKVEANSIILSEKMRNNAEVLHGDIAQNQREVTLKRFKEG
KFNVLVATDVASRGLDIPDVDLVVQLAPPKEVEVYIHRSGRTARAGKEGICITFYSGREYSSIQDIEYDAGI
KFKKISPPKSEDIIKVAAVDAIENLKLVDKKILPLFQDAADKFIEEVGAREALCMALAFISETSKEHLTSRS
MLTGEDGMVTYLLKGAKEIRNVSYAYKIIQRSFTEDVSSKVKGMRLIKNGEAIVFDIPDKESESLDSQFKQ
EACSKRGLPFTLEKATDIPDFEEENMKSSKQGGGYYKKYQGGTQTQRYYKNNDYYNSYSNDYDYYDDS
YAHQSRNYNYRANDFSAKTSEKYEAKSKTNEIKKELEKKDVSGITFGSSKPTFSRNKANSNLERPQTEQK
SNFKKI

>CUFF.21709.1 hypothetical protein SCHCODRAFT_47866
MDSGSTTRSATHAGSWYTNDSDELRGELDGYLEMAEKTLSKDSILKGIIAPHAGYSYSGPTAAWAYTNID
PTKYKRVFLLGPAHHTYLDGCALTQFTEYETPFGNIKIDVDTVKELKDKGSFKYFDPENDEEEHSLEMHL
PYIKKMFGDNEFKLVPIVVGSIDNKKE**KK**FGKILADYIKEDENLFVISSDFCHWG

>CUFF.24005.1 wd repeat-containing protein 65
MEVKDVYGVAEDEDIEEIKKRPRVKLDFLNAFGSLFSTSNHEIENCVNFLQDSNQSTIIYPVGRHIGVRNID
SNDMRFIRQNQNLKEITAMCLCPQKRFLAVCETHKNNLSAYIAFYDMKNIQFRSEKNCINISETEPTHQKV
IKSICFSSDSNYIAIIIEGPEYSVISFDCKNKSKCKSNATCDFGKTVITKISFNPSDNHQLCTSGHNHWKLWR
VQEATLKPMPAFQGVGSHLFTDHCWLGDERLAACTAEGEIIILDDFQQKQIIENAFMSEDIYNISCIKAYSK
GFFIASDNGIMALWVRSEENNQSANKENQLYDFIRRWSPVVTKGTKIISMDVNTSEEFIIVALQNNNIALV
NIKSIGLNDSMTREVKVDLVCRGFHSGPITCIDIAVQRPIIVTCSQDDSTIRLWNYYTFKCELTREYFAHKE
MMIVEAAKPLLTVAIHPSGYYIAAGFKDKIRIYHVLHDDLRIFRNIERKFCTKIKFSNGGQWLACAVQKNL
YIYRSYTLECVFSDKTSSSHVTDMRFTRNDTCLCMITADGFMQRWIYNNGWCKLNDGPVSNKSLDLRAC
PFANAIEDELRVILVGGDGLRSNIKVLTKKDEIFQNFYGGDAKITAGDLVLTPSKISNLIVGTDKGLVKVY
NMPIFHTEFDEFCAHYGEITQVVISPDSKYAFTAGNDGCIFVFSITEYLNEHELYKPIGLEEEKIQNEDFTSM
IVDEALADIVLVKRAEMDEWRKKQEQLRQEMEETSNRVESTIADVKNSFKKQMTEQEISH**KK**EKKNLEE
RYKALMNEKAAQEHEYSQNIKRMELNHIEMIEELKAIFDKKSSIDNSNYLTLEQKKIEMEQSYLKKIKQM
EDDNRKMIEDLESNYKSRIEEIMGEFEESKKTADGLKSMYEMKLTQQEDEHEQEIAELKAVFEKEKKELL
DIIDNIKTEKDNFKRDKETAESEKAMLVKERDKAILERDNKLKLCDELGKKIELQDKEKKHNQEQLKQKE
HDLYKYKFKIKDLQKSKHVLTHRATEMRASLEPKEQQIESLKEQILNLEKVFEQQMKSMNESKKDLEKK
QFKITQLTKELNAQKNLTNESDKKYFKIITDIHNYVQNKDEKSYASGLMQLNQDYVRPRQSELLEKKKKD
PETIEELDRQLRYMERSITQLKVNTIKNENRTKLHIKKKTDENQKLLGEINKMKESMYELNISNKKLTNQV
REFEEKMRKDVKQGEYGIGKQPSVQSMTMKGVMEESIPSLVAGRPHSVKKQNQGKLYKGTPYQYRKSN

LEDKAKIAELSAQLEDTQQMVMIQKLEIRSLKEKFISLVNDRNRLLAQLEGSGSHQDIDQNRQPMTPDHE
AIDGDGDGFLPRVSSKGGK

>CUFF.24130.1 adenylate kinase domain-containing protein 1
MLMCSMHSNLKLAKLRIKQYLVIKEDEGIRREEDFIPSETDFKQMQLFMLNKKFESFYSMRAKAEEKEQA
RLKKVKEREEK**KK**KSGMVPQKLEQIRDPREDYQHTLHQHIRHLISKDGIVEDEITKKFDLIVQANKPPLCI
LVIGKPRSGKSNVSKSLSESLDLVHVCVKSYINSLLLKISTYEPPEDLEEGKEPPKFLTDFEEDIFQTLKAGK
GPCDSQMVKMLAELVGSAQAQTKGFIVDLPLHQREESWFDTISRGALNFLPQDLSYVIDLQMTDVDIKQR
ANGIRFDPETGEIVSKREREDRRKPQKNKKEQQTISNEEGEGEGEGDNQEEEEPEPDPDDPDAPKKPKVLD
EDKVLIRVKDFDDRLTEELNNFNTVEKQGFSKIIKNLYHSQYIQLNCAGMKADTIRETLVARIKGDNTLLR
PLGIPFEAEGDNKAYLTSGKEEGELPRRWSLWRQTDPVALIKGRVVQGQTEFAASYNDRVFLFENDQNL
KLFCQQPKIYLNTPPAMPDSFRLLLSGPTGSGKKTVANILHEKYGWKIVDWNEIVIRKIDELRARESHWPN
NPLAEGYGLGLSEEEWNSVLEGKPIDAFSLLPWFFEFLGFKCEKRRPPPNVIENEEDLTEEEKAKREEDRK
KEEERKKEEERKEKEKKKKEKEKEKK

>CUFF.24134.1 hypothetical protein OXYTRI_06194
MRKVNKYAIAIEIVTDISITYKDFTPANIIKARLHIINAEWELVLETIQKVLYYEQYNIEALRIYIFYLLSREK
DEEALTEKLDELKTAFEKHES**KN**AEAYFNYSRLFARICGRKPEILKKSKELIEKACQLRPENCKYTAEQAY
QKCLIEDYNDGFMTYQKAATYDESNMDPLYGMIYCRIMQGKIEDAQQQVELVNEISEGNPKSAMHYFLE
AMISYRKAQPKDTTIKLLDQCLNLHITDTKEVPAGFEFYTKLNPDFLLELAKEYMKHAGVKPLPKSEEIPR
YMNKAIKLLENIIRQYPINSEAQILLAKARWLTNEVNIALKTLHECLKTDPNLVEAHILTAIINMESGDIQA
ANNALQQAFSQDFTIRDNPVFLLIRAQVDIKM**KN**FEDAQKCLETAYELPGVKDKNMKSKDTGPKKYNLA
FGQEERVKIFILLIEVKAELGDFASSKKILQKAIAEFTGTAEEVHIIISQSNLFMKMGDIKKALNMLKKVGPE
NKNFIEAKKKMAEIYLQQLRDRKNYKRCYMEIIDVDGSEANFRMVGDSLMDIQEPEEAVIFYEKALHMN
REDISLVREIGRAYVMTHDYHKAIRYYETALSEDPKLLDLYADLAELYFKLKAFDEAKRVIIEAMKSLTS
MDDPDMTNPKRVQYTLLMAKIFLEEDVQSGEWRFKPNEDALKALKDALKVQESVFDKVKEISIDRIDEE
RKVTADIHYKLGKYLEEREGDIEQAIMNYESCIKRYESHREALFSLAKLYLSQSDTDRCVYYCKQLLKLD
PSDEETSFMMANLMLLKGDTEVALSTFKTLLDQKPDNFKALAQLVQLFRRAGKIEEAEKYIENAEKNAV
RSNEAGLAYSKGLFFRYTGEPQKALKALNRARFDSFYGQQALLLMIQIYFNPHDELLYSSKEKGPVYKTS
PENMKAAESLIKELSMKEYDTTILECYGMIHTQKKEYLAKAMKMLQDMLNSNAEYVPAIVCLAFCKFIS
KKNSEAKSLLDKMNNQNYLPEYAEDFETCWLLLADYNINNSNYAEAEQLLYKCLQLNKSLVKAEEFMGI
ISEKEGNFANAADHYQAAWKMSLMRNASVGFRLAFNYLKTKKFVRCIDICKEVLKNYPEYSTIKKDILEK
AQKSIRSS

>CUFF.24206.1 UBA/TS-N domain containing protein
MESYDIANIEDIITDWYFTNNDDMDRLGLEFVEVVKNEEDGTFDFMFNRNGHLWHLKYEFSIYFFETEHE
ADFGLIEMINEQVTQMDEEKGIFQEDPNSVVISILEQILIKILKDQKSGKNRIESDENFMNVDEHEEEPHDEE
EHDEGEGAGWSDEEYDIHDNPELKKKSSTFDDQITRYNINLGSKDSIKIFRNMRAVLEAKEIQLLVDEHLK
NYPDTFQVQIFEPLLMFKLTIDLNFLSLSPHTFQCLGFDLNELVEFLFIFDDNKILMFLDDDTIYEKDMEEL
AKLGIMKVEFIQQTNEFSQARYQNYLQLLHESFFSKDKKSKSYSEEFKHIEDEEMLASKSTLLEMGFGSKE
SDEALKSNKFSVSKAINYLLGRKKSKKVEDMKLEDDDEIISIDPKHLFFSNITIEQLKENLLLNYFRYMIYG
LDSITNHCCICRDKLPITSSKIKCCEKELCEFSFEEALGIFITPEIRFDPDSFTLDLSIFSECVMGGRAAKTFEPF
PTFFLKDKELRSKRGYLDNIKEARIKGGEAKDVKESNKDIKKIRDLFKYIPSIDKCLAKCSDDAVLVSTIQK
QTKEPSDANSIYKLLRYLICTNRVNFKHLTGENAVSGDAAIQEFLIYNQEATQEQKFQELKKTHGSVFTFH

GSSIENWYSILRNGPRNLSNTKMMTAGAAYGQGVYSARQFATASGYCGYRYYGATTDGLGQATSWKHS
VVKQKCVIGILEIIKLASYSKSGDYDITVCPDDQNIMLRYIWVFNQGTPFSTTGKTTTDLNFSTHYYDQIQK
IKEERTVEK**KK**RLKHAHDRAQKRLEEQKEMKERLEQQLKERDKAEESKKYDDKIEKLENKFIGKGSVTA
TKRILKEYKHFQTNANLENFEIRFKNGDNFYIWTILFEPLKIELTPELKEDFEYMKDKQGNEPTLEFEVTFP
SSFPFDPPFIRVIKPIFMFHTGHVTIGGSLCMESLTPSGWTSAITVEGLFAEILSVICQGGARVDKTRIGHSYS
LEEAKSAFERVARHHGWLK

>CUFF.24232.1 C2 domain containing protein
MYAGEDMFRERRTGYIPPHEHKDQKILTDRRKTNLMKEFDEIDINKDGFLE**KK**EIKEYIYNKAKEKDIELE
EIKNHPEIRERLQKHDELLERMMYDLDLDGNDKIDKEEFCQTYLYKIQELEKNKYRLQEDLKDLQTEKFV
IEDREWERTYGRSAKEYDRNLVDLRVTVVEARELEASDMLSGSSDPYMLLVLGFQICKTTIKRTTLYPVW
KETFTFNKLPNENILEVTCYDYDTITSDEIIGSKKINLNALKMNDVNDEWYTFDAFQGKTPHGKFRLILHL
VQHIPPDYKKIKLDLE**KK**IKAKEIEIYSNDLMIEAMEKPFAMFELEKLELERDNAINDTVAPHGFEKSISDQ
IHKLTSNTPWVEILLVSLLVYSFFTCLVCFFRPDFLDLVNCCGGIYLIVYITRATNRDYRYYFFSLLSTLLFD
LLFLLFLAAAWWADQHFDGEMEAGMRRYSIVISVVLIFIKIPIVIIFWHTSVHYFELIQPIIDKIRRLSR

>CUFF.24237.1 hypothetical protein OXYTRI_16010
MDSGCFIKKLSPNCLSNVFQFFDGKEMLVYSTVSTQFYKTHKIDYLWKILASNQSIFVSKRKKESWRDAY
VRHVLCISKNMKGGYIDPKTGKSIFTYQMCPIRQHKEIIKQVEIFDNIVISLDEDGIISLSFISYEDPEESKSCK
LDEYQGRDVICFNYLATNNTLFVIDSKLNISLYFIEVDEEKESCSVRGTSYSSHVDLFGLGDNMEIEEYKLP
GEI**KK**PRHPIVNFFGNKVIISSDFRERLAESKGNIMIINRENGDILKTITCKFDGIEDAMNVDSDFGVYIDPV
QIWDFFGSNIQKNTICYDYRYDRIYFSDFLCNIDVYDTNNCKFITNILIKSHRSEIDQREDRLVNSIKEVVFSI
NLNDSFIYVTTNFYIYAIKRNDLKVVASMKLNGIPIKINFFPVSKDERELLKTSKNELFMSKITFGFSDKTNL
CWSTIYHFINNEEIKEADGNIKETQTSSFKLASEGAMEEVKDNYFNYRRRYFPYYNIYQFNEAINSVDVSN
SLAVCAARDMKVSVFDIGTGKEFLAFLE

>CUFF.24250.1 ubiquitin carboxyl-terminal hydrolase
MKQRNLLRNGWSIENNLSFQYKDILQRGKTNYYKKEGEIGYIISLNWLEAWQKVIYFDFFSRGYKPEYDE
ERSKDIPPVDNISLLRNKHSFLTDPDETSYLNNILKVNMKMNVDYKIIDEEMWYFFYTKYGGTEVKRYYH
KAFSFGAEIEAKLKEFKVVILPTLEDWDSNKIDKPKSIYASKHDKFQSLIDRIIKIINVSEKLNPELTSENVRV
WKLAYNHDLDKIIQSVSEAKDSNMDIDTDNSAQDSKSKTVERNTGIKFPGTSFEMM**KK**FDLDDVEASST
DTLVFERASKTSGKFIFYYEETKILGYGKCEYCYSHKPLVVQCRCEEVKYCSEECMKKDERFHIDKCNAPI
DVGNDVPFTKKDRARDGLTGLQNLGNTCFMNSSIQCLSNTYCLTKYFLLEKFKNDINADNVLGTGGKLA
VQFARLLNELWNEEAPVVTPWSFKKIVGNFQPMFSGFAQHDSAELLSFVLDGLHEDLNRVIKKPYYEMP
DLLPGISEEKNAELSWKYHLLRNQSIIVDLMHAQYKSTLCCPHCNNISVTYDPYMMLSLPIPMNEIESGFY
YFVFYDNNKCPIKSKYYMKKSCSIMDLRKQIAAQMKADPWSFILCQIHDNDMERIFCRNRTVSDVADED
GILFAFQINPDLFQNERDPDSYKMLTKLLEKEETALDMSNDDDFNNSISREWVKVPLRFTMMEKSKYSYH
ERKNEQSFPRIIWVNRNWDLVTVHKMIFNYLRYYFDFEMENFKNLSEEEAFMDTFEDLTEDNWKEVLGC
GEDAGEYAYSLQIVNTEKKSYYSKGIEFFGLKNFDNIPLPFKHDITFGELIDQFFLEYENKDESSDDDMGFG
FGKTKEKPKKEKVTQVQNNKNDGYYFDRDSKYEKKRMIFELEIFFNNKRQQAAIHKLTRCKKHENLTEIT
EFADKIKSEEITLKQCFHSFMTAEVLGKDNAWYCKKCKDHVEA**KK**KMELYTTPPILFISLKRFKSGKGSY
FKDKLEEKVFFEIDDLDISDIVLSNKNPDGTSKQDIIYELYAISNHYGNMGFGHYTAYAKNPKDGNWYDF
DDSHVSLVRDSSGIITEAAYNLFYKRKDFAFDEEPNFDLLKHSCDFEEFKGEVAQYAVPEKKVETKEEKA
NESSTINQVESTDDIMEDDS

>CUFF.24540.1 DNA topoisomerase

MDITADIMETTTEDTMENIIRRALRRVDFMVNNQFNYVKDRKDYEEDKDYNEDHKHSVKSSEKSFVHNE
SKIQSNLSKIGHGKALKILMITEKPSIAKTIASILSNGKAKESKSDIKPFVVWEYDGDFKGFDAKVKVTSVA
GHVYSRDFSEKVETWKVDPKTLFGEETIQIPTSKGLCKHIQTVGKDIDLLLLWLDCDREGENICFEVIENV
KKNLRHPIEDYIFRAKFSSLANRDIVQAYENIIHKPNEDESKSVEARQIIDLKIGVAFSVYQTNKLCEKYPM
LKNDTKTVSYGPCQFPTLGFCIERAERIKKFVSEPFWTLNVTIRENVESVIKHELKWSRKRFFQQELCAVIY
EQIKSEKKAIVIDVSESIKIQGKPMGLNTVKLLKVASSAFGMSSHLAMKTAESLYLRGYISYPRTESTTYSS
NFNFIEILEAHKKHSDWGKYASKLLESGYENPRSGKDAGDHPPITPVKCAEKDNLDDLEWKMYQFITQNF
LATISKPAKYKVLRVTFKVGLEYFELKGKQIESMGFLEITPWLKPKKEVDLPEFKVDEQYQIDNMRVQDG
KTSPPGYLTESDLISCMEANEIGTDASIPQHIKNIIDRGYVRVDQKKGRALVPTNLGMSLARAYCQIDSELI
LPSVRAYIEKSCSRIAKGEIEFQKVVDHVISIFKQKFEHFESNFKTVDDIIKKDYVDKYGTENKKKDKDKNP
TKDLIGFPEELKSYEHCTVFEIDKSQITEKVYSLHCNNCKKSQLKKVAPKNKRDTNVYLKCSHCKYEIACF
KDCTKFEILKTGCDICKNAFIRVNYPVNDSPFPMYANQHTGCMFCDEVFQRIVELPETDYVAADENKDAE
ENNDDPNSSEPKAQDESTESKPKKAKEGEIIVKKKKKK

>CUFF.24602.1 DUF1126 domain containing protein

MGSVHALDPKPPKKDEKKFYKSDIHVLRFDAKLISTEPDDENRKFIVAFFCGDDTIEVFESCDRNSGRIGG
KYLER**KK**HKHPAYSHRYYDEKDFRIGETVFLGGHKFQLNNCDEYTHKYMEDNFDVFPEASIDKVMEKIR
SGALSHPSLQDYAISLIKTLDKNGDEIISFDEFRDGMYEVGIFLTEHEINTLLRVFDHNN

>CUFF.24757.1 Vacuolar assembling/sorting protein VPS16

MLSSDKKNLFEQLFAATYDWIPFENVHYSKTTLAEYSFRESDKSSFLKIDLNAFMIATAVNGGPFAMLLN
NKIVPTGASDYKDKIIVLSSYGNRINAIDLKTALSGKSDKENKHWILFEFTKEEDILLISPNGMIFILDPMNG
DVLYKTDYQSQFTGSNMIENAKAKENSVIIKTGTNCFYYIHDIYHPEIVEFGQPDFGLADVITIEETKINTKS
GTKPIQRASSTPVRQIIDDYIMIPKSKSRS**KK**MEILITHPREGLLILDDQKNSLYSSDMSKLCSDDVGKLGHI
TNLALSSNESYLAFYSETHFSIYVFKSDLLKMLNKVETGMPRPEKLVWCANNIPLMIYESSITMIGPNKMQ
NLPLEDEDKIRGIAYCCELDGIRLITSSFIYFLERVQPATSKSLTFLSSDPSNQLIDAYREYQLKSENTERIFR
EIGQRLKEAIETVIEAATYQFSIPFQKLLCEVASFGKKKIEAESYNSDMYVQIIRSLSLINKLRYSSKCRRAIT
YKQLKAITPKNLLPIQLKYGDYFLVLKEAKNLNLKQRYTNMVYEEWACALLKNSMRNPNEIQNEIMDKL
SQLEAEIKLKGGSEKYNSLTGAVSTIDYTKIAKVAQEKGYKEIAIQLVNNEHSVVKKIPYLLSLNQFEVAL
EISISNGDMNIVNKVISKILEKLRDNNAAFREFLEKMRFAHRKFITYAKQEGDIELMKQLRSSMEEVYNFS
EVKMFLKRESEISRRNTEERDKALNDIEENFKKVYKDSFKSGIVKTQRKLISKQDDLNKTQKTQKYAGMT
TRDSIIQLLQSSKQEEAKKVAKSVKMSEICYLGIEAKVFADMGNFEQIEKFLEVKKSKLPYEYIAKLCLEK
KKYTLAGEFINRITDEDLKGE

>CUFF.24761.1 CAF1 family ribonuclease containing protein

MKDLCKTFLAFQIGFCVFKWDESEKKYTMCPFNFYVFPSSRFKDSILSFQATTLEFLTKHNMEWHKVFTN
AIHYWQRNKAEELKTSIENEENESEESKRIYWYKLGDRSEND**KN**EMIEVISTYISKPTKEKEAQIFKANRN
KSCQSATIKEVSKLCSKNKNVKFYVDHKDKSITITKYPSVDEDTKADESEDAKDNQNESMSLSQKLDNLS
ILSEEKKEEIIDLFQEEYGFTIIIDRLIESRVPIVGHNMIFDIMFLYNQFIDDLPDTYDEFTKNWASCFPFTYDT
KLLSSYCSLINKTWLKAAFDKCLDYNDQMGKLKFEYFTDSRRKSFDDPDGVDFKTYDEEAQEHKASYD
AYMTGVLFASICKFKEIVHEAKGKLDTIEEYQSLKKNKEKDDKMKVSEKISEIEKEVYAESRKRINGKKIE

TSYITEFENKVVVVSSKNKVFYFGTNKDEIEKVKKNQFNEDKIIWVKLDKEHNNIDKLIDAVHDLADVYI
LKDDKDAYYIELQTIYEDKLMKTKDIIQILQEKLGSGCKVTLFKDAEKY

>CUFF.24771.1 help domain containing protein
MIIYINKISVFGGGEVPMVAKRLSKDLQSHTDDILCLCLNDTRKLAASGQIGQKPYVYVWDCETGEMRTQ
MRLEMGSIGVTSITFSPGEDSSFVACLDASDDHNLHIFQIRDGKFIQKLSSNQQKKFDLDWGKTSAGENVI
GVAGLKQIMFLVGNKNNFDGCQFRAGDVKSAYRTDYLCIGFTSKGDCLAGTSSGKLYLFKQSGTNYSFK
QSINAHTKAINCIALDENYIFTGSCDRKIGIWSQKLKKLKEIATDGRVCSVDYRGGSLLYSLATGLII**KK**IV
KIDNEVVTEVPNSDEIVMCSHNSGEVWGICVYKHYVYTCGDDNNIYKWNFKTKSVDEAMPCWTQKNED
EIGVKVHSKKNISKKKMTASTMSHVKEMYQTRALAVNTKFKHIAVAFNDCKIVIRNIMSLSNIVQVLYDP
DEWCEILEYSPSETKLAAGSHDNKIYIYNVNENGYSLYCILTGHSSFITCLDWSDDESYLRSNCGGYELLFF
NIDSKVQDPSGASSTKDVTWATQNCKLAWNVQGIYPHGTDGSHINGVCIEKTRKLLATGDDYGLVNLYI
NPCLEDFNKGRSYRGHSEHVMRVKFAEQGKYLFSLGGYDQTLIQWRRVGEESDEEEVDDSDYEESKSLN
KSRQETSQIPSKTVEDHKNTESLVEDSKQDLLKKNDISKKDNTIQEESSEDEEDSNDKEDHKSPERIDLNIS
PIKQLNEESNEGEEIKEEIS

>CUFF.24785.1 UNKNOWN
MFKEQQENIEHSSSDNSSSSDDRGRDYNSDNGNDRRRGRVADDNSDDNSSDNNRGRRGRNRRSISNSSRE
GDYMKYDEREVDSKRYRNDSEDRYKRSKSKGSEVIMPGLNGPLLSYKAFMDMQENPISMSDAEKHYSK
YKDAYERKQCEIFYQIHKDEHWFREKYDPQLQFKFKNDQMKQSQTLARRFMEALERNEFDQICLEFQEN
FENAKQNEKEISFEKKGAQDDEDISGSPYFGFDANKTTLYLKLIPIHISRWDLLDAVKPTPGFLGLSMSEPL
KTQDFVRYAWVTYDSDENCQRSKTILEKVSLKDFDLNPVISISTTRKKVKVTPLLNKGCINRDKQLSKSLI
EIFDKQRQIEDNRIFENESERNERLQLDLQLLYLRRVHAFCFYCCEEYEDERMLASKCGPMHVRLMSIGG
NTQQDEAKDLDEGEVYDENAPTVFEQ**KN**IEKIQKIIEQGPQSMQDPLEDEELVDRRNEYCQKKTKELNID
RYSCQICDKLFRAPHYVEKHILNKHENKVYAKVDKKRFDELLFENFIKNPSKSCLINSI

>CUFF.25022.1 P-type ATPase
MSIMEKFKKCFDRSADGRHSVQRIAIGFGDGKEETNKHMRYTNKTRTAKYTWYTWAPLSLLFQFTRVAN
IYFLFISILTCMSFSPKSPGSMIGTFSAVLIFTMFKELFEDIFRMISDYKINNTKATRLDQNSKNIEEITWKDIK
VGDIIQIKKDEAFPCDMLFVYSKSDVIFVDTMNLDGETNLKPKTIVSKELFDDIKNKNNANIEEIEEIDNSK
LALSEVSGKITCEPPSENLESWDGNFEIINTKDNFKANCHGDINSLLLRGCYLRNTEFCYGIAVYLGQRTKI
MMNAKKPRRKVSNLMKLMNYMLYTVFGLQIGIIILFATLSCIWINNKGSKYDYLNIGSGNADFGKWIIQL
FTYWVAYSHMIPISLYVMIEVLKLVQASLIKWDDEIGKEDSEFKPAECKNSDLIEELGQVDFIFSDKTGTLT
RNQMVFKCCSVNGDIYKDEEEEMKVEDIISSERQNQSKHKVYENKKDTWRGAVIGRKTADNHAYQFFK
HMTICHSVMVDKDSKKNKISEEGKQKSDDKIVEEEFVYQCSSPDELALIDAATEVGIVLVDRTKEYVIIRD
DNEERKYRMHAEFTFDSKRKRMSVIVEEDKEYYIYTKGADNEMIKKIEFEGNHCDTLKEHLHHFAIKGLR
TLVMAKRKISVSEFNDIISKLEQIKSSDKANKEEDFDALYEKYEDKLKFVGASAIEDLLQDEVPETIEKLME
ANIRVWVLTGDKQETAIEIAKSCRLIQEGMETLILSIDMKALENLKGLEAEELNRRRKIYEEGFKSQLSDKI
NGYIKNYLDDQKEIDNPDIIFKKDLKDLKIKPITIVIDGLTLALILGDPTL**EK**MFLSLGFYSKSVVCCRVTPK
QKSVVVGLAKKYKNSCIRLSVGDGANDVPMILEANIGVGIRGKEGTQAVRSADYAISQFKYLQKLLLFHG
RLGYRRVSWMVCYYFYKNVVLVFTEIYFAFYNGYSGQIFFADWLPMLYNSLWTSLTCLFAYALERDVSY
KVTVNNPKLYEAGQKKEFFSFKTFWKWIFLSIFHGATIFFGCSYGFRGVIDSSGKTEDMWFASTTAFSCIIH
LVTIKLGIELIFINWIVIAAGIGSVIFYWLFVIIFNTSSISQLFQPELEYVYFRMFSNGKFWIVLFCLPMIALIPD
ITLKYFNQMYNPSVSDKVIAAQSKLGSKNQISQNSKQISQ

>CUFF.25120.1 hypothetical protein OXYTRI_21990
MDNKKEEAQKAHEEVNHLLAGQEREKQNASEQISLQHIMELREAFDAADKHKGGALSLSEFVDAFGGII
GKDMNKKQINQLFMKIDADSNGNVDWDEFMNYMLLENETLSSMKAEHFEYIKSDRDDPAPTKTKHCHN
DMITSILILNPENEGNKLSDDNAKEEDLKGNQKSNKNLQYITSSKDGSVKIWNAKTMQCIKHIDTFPEE**KN**
KDKKKGKKYWVNCIQYMTKSEKLVAACADRTLKFYDLGSTNYNTPVSTISEIAGLPLCMDYFIKEKQEK
ETLVVGDDLGVTHMYDFDKEWHACVWKINVNDETCCHKNDISSNIYSNLKEEMKKQKLEKHTLVSNKT
HIKPVSKQYTKGITITEVYLHEGWITKIKYIEDLNYILTSSFDGFLHFHDVDKLQYKDRTFSLHQKGVNSFV
YSQKHRFVASCGEERHIIMWDPFTRRAITYLNGHNTSVHDLTINDDRNHLISLGTDKVVKIWDIRTYKCIQ
TIFDKICYRPEDRLTCILFDDYTNNILLGSRKINQWQFKTQEEIKTSHEYPVSVALYNTSFESVVSCDDGSFI
AVWDIENGKLMSKFGNAHGKGIKITSACFDGSQRRLVSSGSDGSIKIWNFSNGQEISKCETQDIIAANQRE
VTNLCFVADSNNSSLNMGFILAVGWNKRVYLYPDNKEEEIESTIVLPPKEQEVKHKDDIMSVVYSKLDYL
AFTGSHEGRLIAWKLDGLKMKYELHLEDPTCLSDNPAKDAKSVDCLLVLDQHRTLISGTADQYLRFWDT
KTGKLRNKVKVGHHPEDALTALATSKNNDVLFSGDTSGCIKKFDLESFDIDNSTSLTSEWFIKAHRAIINNI
EVVELENCKEKFIISASDDRNINLHRFDGIFIGKYQSINLLGQFGQDEEWNIYKTHVFDDVRTRKDVIIKNY
GTYAKNEDIEKYIDGTYTLEEEMKEEAEGDQEEDDKMGGLTIKGCAALFKEDFAVSNIKPRRIKPINEEKY
FNAISKRQPKYLLKSDERNDFFLEKVGVPRDYYKDIISKIHE

>CUFF.25142.2 hypothetical protein OXYTRI_23998
MALKFASQKTKDQTKIKNESKSLDIKDTEQIKTSAEMTNQKYLDNLISVLDVVKGYDNLYSDEEKQMIDE
FLSMELIGKVLIARMFFR**KR**IWFNAFHLKDYDKSHENIENSINLIKKSKICDIHEHALQNMKYSWQFLDSL
TIDELKLLNSNLIKQFKCFPPVKINYNICMDYTSYSLCKINNYIGTIDQLNIQIKGAYDEYKIYESEYYKTSK
SQKIKLICSIIERVEAYKTSIHSNLKENVKKSTLHTFFSMNSSKLNSDFAQNSISTQNKTSREQLLNKVLCESI
SEYFSISNIFRKCLNKCINLFYFYRVGDPRNALLNHDFGLSKYAQYYCYLSVKLNHLNDIDSKFEHLIPVFE
NKESYDAYNNCRSFYNCIQSIKDSALPKEMILSIYILTVQCILFKLKYVSDLDEINEDNIDKFIESKVHCIIET
DLFHDTPEYYRENGTNIFLSRFREENIYQKILMCCIDSLEKLSQYKFAVISLLCLIQTQTKISQRDAWFH

>CUFF.25160.1 NLI interacting factor-like phosphatase family protein
MESDKKGHLVAKRDHLDSEYTHDYFPKASKKGIDSDFSNWSERQDTAKTKDKNPQDLNSFDSHLPNIKD
DKAFDVNPVFGEEGDERLPTETLNERETLTRFYDEYTDKKIKLQNRDKRIQSLTRSSISSDNEDRKSRSLER
PGKSSSINQFSSQYQFNRETDPAAFKKQENEVSMSKIPLSFNLEDLVKEEEKLTEIFDFLKREWDPSTLCDD
WWELTEESCLMSVNKYFKEEKLCRTLNNAMKLQAIVIGYTHFMSNLFPYDSLIKNTFKNLNTYIHQNYLII
ILFFLQRLPSDLRNTNSYAITLKEKLKDNKIKTSLTKREAYMLMKQ**KN**DILCKMLKTASHGKPKRSIDDIIS
LILK

>CUFF.2529.1 hypothetical protein DFA_07283
SKEVDIKNKLLKLDTVLKYSIDRFRGVKIDLSDQFDSKEDFKVEISKFITNVTENEGRAIWLKIPIKKSSFIQI
AADEGFSFHHTSNDELTMTKWLDLKSKNRLPGYSTHYCGVGGLVINEYSELLMVQE**KN**PLLGSPIWKLP
GGQVDLNETLEQAIVREVKEETNVDASVSGVLGFREAPNYKFGKFDIYFVFLLNAY**KN**QNNILKQENEIS
ECEWIPLNEASSKLSNYSMMNRRIINICNPSIDSIQKIIQKHKDEKSKESLMNFFSMTRDTYVYMNSNNNLY
LGRLAKECIKHIK

>CUFF.25306.1 hypothetical protein

MASKRKVNVSDYDLLQTLGTGSFGRVRLAKEKATGKYYALKILKKHDIIKLKQVDHVISENTILADIDHP
LLVGLKGFDQDKQYLYFLLEYICGGELFTYLRTEGTLEPQNARFYSGQVVLMFEYLHS**KN**IIYRDLKPEN
LLIDNVGYLRLTDFGFAKYCDGRTYTLCGTPEYLAPEVLMNKGHGKPVDWWCLGILTYEMLAGIDPFND
EDPMAIYQKILKGKIKFPRDFEKKAKSLVKHLLVADTSKRYGCLKGGVGDIKNHKWFREDWDWIALAEK
EVPAEYIPEIRDEGDTSNFSEYPDSPEPSPPVSKDDDPFKDW

>CUFF.25515.1 Low quality protein: proteasome activator complex subunit 4-like
MDNNIYLSHKSLPSPFKEFNEEKGKIIYESIFTGTSAGNMEDVDNNNEIKSILILHLSGDTEQLIELLESKKN
YLLKSGHMTIEDQTIIIEILIDHAFKRKDLHHVNLIKLCQLLYEFLQVNYEKLNLVLNWRYFYNTLFSILND
NELKIEMYTLSERKLNFFNLVEKLFIKLNKFFPQRSGTEIFLALKPYLFFGNSKFSTCCFCFSVLIPT**KR**FNT
DEDEIQEWLPFIFDLWRTVPYNKFYRGLFVRLMANVTQNFCHIDFREYDKWIAFQLNLVITQSNESGHPIM
HVEQHCEKYFAEYYCNTFKSIESLKIDDEMVDESYRSNLDEIVSLYRDFIHPSNKFNNKTSVIYFLKHLSMS
LCHRVRYEKTRLSNDPDCKFKHYFFTQEDIARLIRTIKPLLETALQGDTLLEQVSFCLKNLATLDCDEILSM
FLFVNFTELEQLANNRFIALLNELIIGVLVNTQNTSLYKYDFIQRSIVYLINQAESMDVNIMKGIMNVFNSI
WSVLPVVNKGWSKSVVESTSKSGDYMKYMKTVKKVRIQEYQFYKLYEEIQDYAVKYFNILKSYMNLGI
PLKDDIIRAFWFLLNSMQTDVYAPLILELIDSANQEHSKVIIGGILGNIAARDSEQACKITKYVLDKLLNFEE
GKAQLNYSNFAISEFYLIILNSVLYQSSEAVTKNIDKLFELLKLLVEENKNDKTRTYTSLVKAILKSYSEFA
VNYSGLLIESSTKEHCSNLWLNNCLFDQNTLSVKFRLISSDDINSIYSFISKKSIPWIQELISKSNDRSVIKIAS
YLIKEIEENLCSLLPSKNGSPTANFTEIYYKYIELTTDQIDSL

>CUFF.26015.1 alpha-soluble nsf attachment protein
MEGKGNYIKSAKENEEKAEKKAKGSFFKNFFSSKSERLDDARDLYEKAANSYKLAGEWKKAGEMY**KK**
CAECEKQTEGMPAQYIMDAINCYKKISLHDFLEMAEDAIKMLAEEGRINQAARLRKEVAESFEQQYEFEV
AAEEYQKAAALFEMEEGISFANQCYVKAADLMVMTKDVDYEKVINLYERVITEYLKKDILKGSAKALVL
KVCLCFLANDDLTGAKSRYNNFSLEDPGFSGSREGDLLSNIFTTKETNDADMFLKTIHGYTRITPFDKVVN
QIVVHIKDSFTGFEGLNLAGKDETDGEPDFT

>CUFF.26160.1 hypothetical protein OXYTRI_17931
MNYILKDNNYNGSIETLVQGTNRHKYFKRPLIPVLQSVPPDVVMSIPEEQIIAAKISSEELDEPLSRTVEIQT
DYRESGTQTDPFTPDYVIERGQTPEVLQIAHLNWSRGLPASMAEMELIEQMREKRAFENALPPTSDEACF
VLRRKLMEEQELREW**KK**REDDLKRLQNERLNLLQSALIERNRSEDAHSRRIEDIRLKKTEDKEKSIAKIQR
RRIKVLRKMFKARKNLDNKGAKRDIISEYSNFGSIVYAPITRDGLSLDKIATKFEVQPDALSTYQGVKELS
ESLPRKLLHSKCDVNKIKHKFKKEQTRNQVQHKAALHKMQEIIDQTNEPKEDEEQKKNNEAKKFQQDLR
RPKTANFASARSTAESLDNYKVYKAALLLQRLIRGRDQQDMMFEGKENRLDLIAELRITEEWRQAAEGQ
DERALIQNYQERVLDGVAEGLQAEIIAATMDSLSKELVRFKQERKIAAMVHMAEQDRRRREVEESGRRQ
AEIILSKREDFLFKELMGVHQGKVDNYLQNIISTAVEDNSKKQAYEEAKLKVQRLNKILDNMEEKNNKPE
STVKDLMSCFLIPDIQRRKIQRQVQFEQKRFMESARKAIQSAVSQAGQKLEQEDVLKYDPSNREK

>CUFF.26682.1 hypothetical protein OXYTRI_19477
MLKVRPVTILNFRKIAFSRSLLSYARISSFCEKIDKDKMTDEELIRKRIFKDDKQEEKFIKLVKENAIDFSITK
TPPGIRTSFLILYGPLTIMTLKLAIFQFTSPIEFCITARTCLRILCLNYAFLGGIHYGIGGALYEISTLKHLRTEA
VRQVLYSFVPGITAFGIVTWMLNANPLTVGVLITGFVSLNIINILSMFIDVSYGRNEKLPIWYSGSNYVKFL
SPLHSNHRRFSCAYLIIISMIIFSCMFTKLEYVQ**KK**NEGNRISTLKELLKLDDQEFLEQILEKQITFDEEDMEI
LTKRLEKADEYQKLKEEYSTEEQ

>CUFF.26973.1 Calcium-dependent protein kinase, putative

MDDSKKIYIDDFMSDDEEDSQPQAKIIDRKQYEENKSKFDIKNLISNFILKKEYFISHKTSNIHDEYDIEKKP
IGQGSFGTVFKAREKNTGIVRAIKQVMHESIDNYDGFMNEVAALKTLDHPNIIKLFEVFEDSKCVYLVQEF
CEGGELFDYIADRERLSEPDAARVFHQIVSAILYCHKNCICHRDLKPDNFMFSTKDKNSLVKLIDFGLSRSF
FKYQEAGKGEVFRMETRAGTCLYMAPEVLNNDYSNACDTWSLGVILYIMLCGSLPFEGSNDSEIEESIKA
LNYDFDDEVWESISAEAKDLISKMLVYENQRITPKEALTHPWAKNMLEVSDQVCSDNYVEKFEDFKKAN
HFKKAILSYLATKVNDDDIKDEIELFNSFDTNNDGYITKKEL**KK**GLLKLKKRTDQEIDEIMDSMDTDKNG
AINFNEFISATLNTNITKDYERIVKAFEFFDLDNDGFIDENELKSALAGQEFAKIDINIFTGAIKECDLDNDG
KVSFEEFAQIMSLKLDKLAKDNMDKSIQLTTDSC

>CUFF.27114.1 phytanoyl-CoA dioxygenase (PhyH)-like protein

MFAHRFGCHNVRNLRNWINKDILLKSFSTASRLKELKLEKRYQDILTPDVLTAKAKQLDKDGYTVIDELY
DGETVDELRNEMERIISEAESIQFNNKAIFTTSKQIEHLSKSTDYFLDSASKISFFYEKDAFDKDGKLTGPLN
TCLNKVGHAMHDLNPVFHKFCYSSVVKAISSHILQFADPILVQTMYIFKSPKVGGEVNPHQDNTYIISNPL
TCKAIWVALDDATIKNGCLWGIPGSHKTTPITYFMKAER**KK**IFDKNGKFEKYESSVRYEPEDPPKYNIQN
DIPIEVKKGSIVMFDGSFVHYSNHNHSDIRRHAFTMHIVESSSKWDKSNWLQRTHDLPFRQMYKEESY

>CUFF.27162.2 conserved unknown protein

MLFPREYKDDTMKFGMLVFRCNICKSEVVANPDSEEDNCVY**KK**ESKALASSLVIDKNLINDPTLSRSKGT
ICPECNHDEVVFFQSSSATGLNSLASLVYVCANRNCGHHWVHVPSTEPYEDDDDSD

>CUFF.27168.1 hypothetical protein ENU1_047410

MKFLMDKKRGSKVADLITGINKNNSGSSFKQENRNSCQDNYLKEENIQIKKLN**KK**LIDQKCQLEEEIRYL
KQKLHVNDLLSDKNNSTYKEPKSAFKANLKNEMNEISQKHERELCKLSSSFAEEIEELHETIGSLSSQLNIF
VNEKNYHGQKFIEILDKAKAKNQIVKNENKRLTKKLDKLQLEFDKLTDSYTQLVNNKTELEECKRKYDL
YIEKLENESKELKRKLFKFEADDDIINLDPYELHRKIRYYESQMDYNDKEFLKVRKEHNQIMNYLNLLGIT
KEDLKISCATGVMQFQNFNSKCFELNYKKGSSPKKRQLRPRKSISCNKDEGVATCRKLPAEKYDEMRRM
LNSSKTKKYDVSMDDTLDLDTLSLSSESNKTNNASSFAINIERQTSNIRSIGDFAKSKQSSNLEILKYLNKT
KEADEFCFQENYSENDSGRESFDMISRKRGYEEKIKKSRL

>CUFF.27340.1 arginine kinase

MLVESMAALNPGVIVLKNKVPKDYVNYDLYYNLIPNTEQNIDTFDQCPQTGAFEVSYKGRLIFSKLLSKR
WPHI**KK**VAEKCAGMIDAVENGGDPSEFFAEPLKRVQGKQSSAKQAKGATVRTTTTTNGSRPSVTKNRFV
SSKDRAMSPVNTSKKSSPTKAPQIKPKAVRKTEPDEDEAIEAEIIKKSRKEIREESPEEEKEMPIEAHVSQTY
EDEEIVDEDNKPNLKDLFQKEIQYSEKKEEPHSEPKIKEQVIEKQTKEEKQSKITEEHHEQKIEKSHHDKEM
IIKPEIESKQHHDYKVEHEEAKEKPKMHKENLDHELKEHHEVSEHKDAEHQDKQSIDKTPEVQHHEEKKE
HHEDKNMHHEDKKEHYEEKKEHKEEKKEHKEEKKEHKEEKKEHKEEQIFKAVETKHAEKVEEKHQDPV
HHVDLIKPLEMPKGNNIQDIEQKQPATSNIQDHQQENEEVVFNLIYLLGCCGCRNCGNSCRSSPT

>CUFF.27471.1 hypothetical protein OXYTRI_24270

MKEEIETLIDQLEWEKVDRDLINLRNKKAEKGSKIASITADEFQNQFKKLKRKMVQFWGKEDKVACLKIC
IQCAKLLNDTETPAFYPQKFFIITEIMDEFGNMVYDRMKKLSLQTQGIPNWQNVMDNEIDFRSTPDFVKQ
KTNNWFLKCACIREVLPRVYLELTLVSARRFLNKRMSVNDLDRLALMVRGIAEPLSASYTCMYLARWGE

SIDPYAKNYIFSMIESMYKHWNHAAEYGNPYLEKDKYFRLFEPAIDWLFYCAGQNADEKLFKRSIKTYN
K

>CUFF.28082.1 UNKNOWN
MNICKLSKMHNKVSLLKSKLLLSQFPSRCFCQEYVKTATIKEDEQFIQKARQNIEKKTKEGVKALKVMQE
LEYDKEGYFYNRKQYYDAFISKMFQIYNAERRQDKFIRKNKSIHKSIKEVLTNTQIPPIATLDALVKTIRAK
GFAASAFDDFTMKELDQSTYITHLIEEIQYSLLPGQFFPPHHIACTVSSLASINYKNSELVPMIRNKMLAIID
NKDEIPQNDLHIDEIIFGGKIGQTNRTNLYRGFKNSNEFFTHVETLLKWKSKDENIALKEETNTLPQNEREE
VEEITALMKNILSAVEDTKLIQKEMGEAIESVRSQFTKMTEAFDKFKFLQDHKYIKYELLELEEKMVEAGL
IDPNEVTGKTPIEEMPFLFKMRRATEVFYDLTKDYFPEMVPSTETLFPGFYKKEKRSDIDLKRLEQNNQLN
LKYIGWILGKLSEMKESVRKDVVDKEFMNAYFRDKCYPSEGFNDPLVKKPDLEYDSMHKPYTRFWNEV
TLTYNSMFPKIISLLNSENQLSHLLMLNYGLTQGNVIDSKLHQMIESKTMDALTNSSIEFEDNELIHSIQGL
GQSIFSPENLRKIMETLESRIDNINIDKLCLNEKLRLAWGLCALEQFQSKTLRSLIADINLMPFEHARNELSY
SEFLMLKDIFYSLECVNSLSDENKEGAKDSVEPLKITNYNIRVFCKGSNELLKKYPESRKRLDPFKDSVLS
GIAKVLSKGDIKNSQLIKFQTDGEANKINDLKYPYTPDLLFQNKGETIVNLFINNLGFMVAKRCKYNTTLD
TCNKNENGSHYSGVQRLHSKILQKLNPDKNIKCVYLNIEDMIDYDLENLSVKVNTEAIVDQLGFLQQNFN
NVYIDDLATLGKWFIDYSKRRKSKPSYGLHSETIDKNFIELLGEFFEKNQIYNDRVLNTQESNINTLKSILL
KLSLIEDCMTPEAKSEVERFAQECGYSNFQGLLLSIKQDLDSNFKTTHIKQDPKLEKSWIGTRYGVEVPAL
PESKLQKDLSIELLNRNFMWTDDYFHYDDWATKMKDVYDGFNLCVEANKDIASDEKYFYQDNWRRSP
KGIFPDAKAYRKISIYNVKDSLKEAYLPKLSLLKHRNTFTSVAGNFFLIFD

>CUFF.28108.1 molybdopterin biosynthesis
MISDQTFTQSPYPMIDMEDAYEIIDKEASAFIKRTMIVNLEESFSYVLAEDVTSTVNIPPFRASIMDGYAFK
RSEYESESQFILVSERSLAGVKDKALNEIEIGICCAMYVTTGAPVHSHFDIVIPIEHVIKSTEGVDEEEIKINL
LQVSKPDGGDILFLRQAVTNNWIREIGCDVNIGQTVLTDGCYIGCAEIGILASIGKAEGIKVYQKPTIGLAA
SGNEIVNCEEKEVKDGDGKIRDSNTPMLISIFKSHRFDNIKSIGIISDDYDSIDEMFNKLSEDCDLVITTGGV
SMGAKDLIKPYLAKNGEILFGRLNMKPGKPTTFGKLNSKLIFSLPGNPVSCFVSAQVLIIRALKIMSGYKD
YEPSIINVEIPKPIKIDPIRPEYHRVLIYSESGKLNLQCISTGNQQSSRLLSTVTANGFLLLPSKIEYKKDKYDE
ETVQAILIGEIYHKR

>CUFF.28206.1 hypothetical protein OXYTRI_15272
MSSKKKSGCCERKPGKDKRQTEEKKLKMHEDKSSKTSKKKSGSIEAKVVLLGDSGVGKTAISTKYTSGY
FPDTTAPTLGGSYNKKEVVLKNGESIILHIWDTAGSESSRAMLPLYYRDAAAGLITYDIGNERSFEHVEYW
SNELSQKLKPDTFTIALVGNKKDIAEEDKKNNNFICI

>CUFF.28405.1 hypothetical protein OXYTRI_08530
YTMEDIVELYKGQNLDGKHGEEEDKDKKEDDEDDILSHLNYRKLEKISDDFRKNPKGLSLTEYLKVMLK
HLPDVQDKPNLVKNLIELFKQIDVNGDETLEWDEFSNHIIELGMVRKDKTFIDAIKNYYASDIIDDEKHDT
EVEHVFFIEKLKHLLVMERDQKRFKVYDCKTGKFKQNVPDKSGSSGGAVIAADYCEHENLVATTSNNNS
INLWDSNNYIFRQRIPTSEIQLTVKWCEPIQQLFTGGCDSVIHTYNVVDCEEIGVREGWNPLKKDKVGHEG
PILDLLPIYEKGYLVSCGIDAQICLWNLKTLEGKHTLVGHQLGVYCLDWYADSDILLSAGLEHDVYVWNP
LVDKRIFLLKGHNHSLVGVKHLKGTYQLISADISGMFRVWDVRTFTTIQTFNCPLNEINCYALTWPPKRIV
AGGRRLIFYDYDEPTDHHLADDQQCLCVLYNPIFYTFITAHPKCIKVWDACTGKLQSVFRELSTKDITCIC
LDERQRKLFVGNCKGHVFSINIKNGAPMKKFEDHKGDVSCLCYWGDKNILLSSSWDKNVQLSDDSSSKP

EGPKRFEMDKHKSAVNFIDFKPDHTLCASCSDDCTVIIYNYGSYRQEGLLTEHELEVKICKFLNPYHVLAS
ADLDGRIYFWGVMPSASRNELLCVVKNDTESEVGTIENFPIRGMDFDPVNKILYTGDEMGFMHKWDVSR
LINKVQDLDRIVEETKGHKTAEKLIEFKKKFNQFETSKRSREEAKDAKPLKSKKAAKDTTTFMTEASVKE
GKDISEEKDVILLKCWKAHTDGITWVTFNSDPPFFVSSSFDKNVYIWNEDCVRIGSLVLGHDKFWNIHIDK
SERLEREKKEADDMLAELEKEDNEFNNEKDKSTKERDLKIMEQIKASTLRRTQKDRTYYDDV

>CUFF.2860.1 UNKNOWN
MEEEVCYIPIEAVDFEPEDNDIFFRIGDKKQRLIFDEPLEFEDEELEKIKEFNLYLEQNNLKLFEEYDWRLV
YRFLQAESFDGEKTFKAIAEHKDFL**NK**HVPPQIKNINHYHDNGVLYVCNRDKSFRPIIYFDLGKLVETNM
NEDDVLNYTVYLLEWLLKNALVPGKVENFFVVVDCANLGFTSIPNDKVRKVIGTIRNVYRGRLFCAVMI
NMTWMLRVILAAVHAFVDEFTSKKMHTYGDDYQDFLMQHIDSDKLEERYGGEFSMTCESYFPPSLL

>CUFF.29246.1 amino acid dehydrogenase
MAIKEILKGHSDQRTKIFFKTIFESQFRFWLKGFLRNSTKSKIQKNNKANIEICHSTCLMMDELVSEITNNK
FDLVDYHKDFFLELGNESEQGLKDLEKDAEFMRTYDKEYTLLSKDQLAKYPGFNYGYNLPIKILNTAKFC
QLSKEFVEARGVKFVQGTVNQINYKDRKIYSIDYLCKDQTQHTLNDIQEYVFCGGTESINLGKLVGLRVP
MYGFKGYTFNIFLDKDAMPDTSFVL**KK**EGIAISRIGINTTSMVRISGYADLVGLNYDFHEFRKEYMIKFTK
EFFGEKYFDEKKANFWVGLRPVTIDDVAIIGRSTKYDNLFWNTGHGCRGISSSIASALLLSNAMNGSGIPE
NLVPESY

>CUFF.29505.1 Armadillo/beta-catenin-like repeat-containing protein
MSEDIGAILTGCAKPKSNEERLSNESKIREFKSQDPLLFIYTMSEQLNNSDLDVSGRHLAGVLF**KN**SVKGG
EAEPYWYSLSNEQKEELKNRILAPLADDDNNVRLSACSCVATVACLELPRGEWQDIIQNLCNNSYNEEDK
IKQSSLKTLGYICEELESSVLDKEQTSIIVTALLEALVSNTNNEEIMKISIEAVLHSLIFAESIFNEGHGCIIIER
VLQCGLYNSVHVRTSTMMCLAEIVRLYYPHVHNNIEAIKELTFKIMSEDVDEVITLAIEVWCSLCEEEIYL
KKKKEPCNNYVIVVFKELLKLMLNLLNDSSIEEEDDSDTWNKSTAAGCCLHLMAQDVGDEIISDVLEFVS
GKIQQDHSWRDKYFGLLALGAILEGPSKDSLVNILTPAMNTLLLLFEDESVKVRETTAWFFSKVAQHHVE
LLGTESLFPDLYAHIDKGLKDDSRVACNSASIVTELAKSLKPQEGQNGNILSNYYSNLVEIVLTCAYRTDD
LKHSYGRSENKIAIACFDALYSLFEYAPPNTEPFLLESLEHFFNKLKETNAKPLDDKAKDMQSFLCVCIQTI
INRVECSLQKEVAESLVNVIIDCFKARDDVFEEGFLLISALCSKFKEHMEDYVPNIGPFIFHALKNSDSSDTI
KNACGLISDLCTMVESQSIISAFEEYVPLLHGIMAKRNLHRDAKLSAVTAIGDTYLMTKDKFFPFLDSTLK
LFSSAANQCIDVNLNDDDLVDYVVKLQGSLIESYTCIIQEINPQDESYRKIEEYIPDIVKFCIICVQDRFTPTL
SKVKEVAGLIGDIASTFIKKEYFQYPELEEIVQFLRNSEDPEANGIGDWVFATISSIMNQ

>CUFF.2970.1 Putative vacuolar protein sorting-associated protein 13A
NELVDTEEGIGFFKLQKQQLMSIFKYLDYSTNYTKFQTGVQKKFLERKFTKDESKKYIKLYEEWKMNDG
DDKNKAEKKKADKLKVDLCEFERDFSYESIAAIRQVSINKYNLEFRKKIERERMLKRIKEFEDSKAQGYFS
GFWGGKSEEEKDKDEKDVNDFKNKLNKEFQDKFSEEETKVEDKLNQMLTTDPIFDQEALQDMPLDWVA
SIYSIIIPKLRIVLLDEDSRANKEKAIIEIQSSGMRTKVLMGRDWQKVDLSFGKLNIIDNFSGSDIYQFLTETV
FPGDGNSSGKDAIQISF**IK**NPRFEDGIIKLKIYSNACQYIFVNMPLIEKIKHFSQTTSKAEDKIDLSYYTEQAK
IKALEYINQGADYMESNIQTEYVHQGIDADIEILAPVLVFPESISELTN**KK**TLMFNLGYLRITSELRPYHKE
VDYKLINIGAELYDQYDIKINGFKLTMVEELIDYK

>CUFF.300.1 hypothetical protein LOTGIDRAFT_101291, partial
MIRSRTKIITMTFNNKSVVTLFNLYRRYFSRSMLLKNNLTNFIVQKGDLTSLKVDAIVNAANSEMKHNGG
LARAIVVKGGNNIQEECDQIVSANGEVKTGECITTAPGKLPCKLIIHAVGPVWKGGDNKEPELLSLAITNSI
KKAEENKLQSIAFSAISSGIFGFPKPHCADVFMSTIFDYFNEKMLSTTIKDISCTIIDDETVEEFYKAYDRKF
R

>CUFF.328.1 UNKNOWN
MKAKSKEFTDEKLKGICMLPQFDEPGIHKQENDDFLKPKPMVSFKFVTVEEKCVKIWKYEDEIMTTYRKI
NIKYNLEDVSNSEMKGFIFVLSSNGKVLILDSEGEYVTSLQRSDIEFSSISCSYENLYLGTTNGTVHCYNVM
TLISSSKKQISYTDLLKPFEMKSREQEDIEMKFTGKVSNIRSS**KN**GRRVLIRFENKNFYVYNTRRDCIDGVY
FAQHSHKIQSLEWLSKSGKTIITVSEEPCLVKTKISEAGAWSSKAIDISQDMYMKMIHDGKNEQLETIGTK
YANSKFELDLKISLTCCCNNPIRPSIIYCGDNKGYVHVIDVNKCEKLNTYNVADVSVASLTTNGFYIIIVFV
DGSCSVFDSAFGFLSSLEKPFKKDVVSLSDETKNCLKARIIEDSYSKKYAQGYNSAHNFRIITLHTPCSLRL
QTFDAEYRTKLFQCSYDLDGIVAGFEIHPSNEYLIAISDQGFFLYFKIETGELRGKVPIMSDPLGIAIDPSGL
YIATSVNNNSIEPNSVIR**KK**WMLNTEKVSSCKGSRTRIVFYEFGTGNFATEINCLFEIACFGFSPNRKQFIAG
SKYGCVSIWAIGERLQSVMTMSPDLWSSFPIYIKNEYMKEIELENDDKMYRVPLKENLEYVKEEYPAYVP
IDHQFDVAPHNKPINREKNIQISPRQPLRTTQSDHHKLMKEREIQYAHGVNETSPNRYESPKHKQFEERKE
KIIYPRKTIDISEKQRDNYSIIPPASSYFKVDEPSVQPDHIDAQIQTPVQNKVAEVGESQEKLSKDSLIYSDN
KESNKKVTKQKFNKWEDDEESQQSRNSPVVYPYPGPYQHPMIMPTYPANIQQIYKTPDGKLLVPVQSVQ
LANKLQRKNDIREERSDSYSRSRTNLTPLDDVERNSDIEDRKSDIEVHESPIEPDQNIMHSGIPPYSKSRIIKQ
AYVPSKLSELYKRRENGVQINKYQDERISGVQREDFEDNPAPNKAIHEPIRLVRNEQARPAHQDLPRMAQ
KVAHPISNPHFIARNQNEVRPDPIDIDDGLEQITNDELARDPESLQRAFVSGMRKADTNQPMSLNRFRKEF
RDDIAPVKQDSDDIDRLSDSNYSVVEQAYRDMDEFDMKVETYGRGKHYTEKVVDDRAFGEKSHKTINNI
FQPQRMQKNKIRY

>CUFF.3381.1 serine threonine family 2c
LWDIFNSASAIIPHPDKIEKGGEDALVVRKDLLSVADGVGGWADKGVDSGLYSKQLVKNIAEIHKIDENL
SP**KN**LLVEAANMATNIGSSTCVIAILNPRDHTVHTTLLGDSSYMLLRPDLNGDLIKLYRSVEQQHSFNYPY
QCGTNGDDPNLAIDEEHKVQHNDIIVLGSDGIFDNCFDNEIIDIIKSSINSQGELTNIQETTNLVAKLAENHG
MDDNWRSPFQINAEKTKRYRRFKGGKQDDISIIISQIKFEK

>CUFF.4058.1 tetratricopeptide repeat-containing protein
MKPDLYNERTARAHYKRLKEILYSPGVLNLSSDIDSLFSATSVTKSTENESISAENKNNQSSNEVQNDSIA
NSEVVTHDNTNNSELNKSSDQVNTEIKKNDAVDEAKEAEDEYVKMVKEFQEVIKDAKRNISPPSMDKETI
HLEHAYRSPIDSDYSAFIPVKCIESIGFSCFNPVPSSQEMKGDLLYFRVKTLEGTEHVITSNVKGFYINNSIES
LLFDPEVYSKGNPCYSHSLVGLLCQLSPRFSNKLEEHINRILKTDPFQMSSANIPTKDWIASINEAKHTVED
FKLNHEDAVSGTYGYESKGSRDWNEEIQVCKDLPKDSIYQRIQRDRAFYKIYFDFCEAAKKGATAIV**KK**A
IPPLNPMDDEEQHVFVYNQIFFSFAVEEGEHSKDAVTSDLNPTYTTTNHDLLGLRTLQVIDIDGLHIIATCH
VNYKGSRVIAQSIIPGILTNTDQTSLTEYGSVDNGSTIHNNEEFNDLMKKLCQHLSIKECSVVDKDGNVHQ
IAGSIDIKGIRGTDKRKYLLDLVRLTPRDSNYLGKKHTNCLVRPELVRIYQKTKDIEYASNKLAEIDKLEPE
ETKQTQDES**KS**YFDMNEEEKKEAVEKSRKEIEEKKSKQIKRLQMFDQLLKEAPQFKYNLNIFTDVKLAEG
DYKEDEKAIRNLGDFINNTIIPKLVQNFESGENVPTDNENLSEIFHSQGLNMRYIGKVSKAIDEDKLPHIKT
LLERCMVCRCATKIFNDLVKGVPSSCISKFIAHFLNILLAPEHLIQKLNNGEVLKNEIREVIKTQPKQKPNN
QVSNEQQINKSNTKRKGKNKKKNKTEKTENNGEHKIEKQSVYKISSLFNRNFKTLLDDNRNLKCLKLKP

KELYSRILRSVKKKYDFDLSSELVGLKCRKSYKFKISFLRDLCLSIGIKIRAKDYNLEELVQTPEESKNSNK
VSQQNNTLPFSEEDILEIIPSVRHIEIVNYDYKSLISNAKTSMKEGYFEQAFDYLNQAININLQIAGPINKETA
SCLSKLSDIHYKFGDYSQAIQLQIKCVILNEKIFGKIHSQTAKSYASLAQIVSIKI

>CUFF.4196.1 hypothetical protein OXYTRI_10687
MDNEESKEYPIGSLDVDLVSESKEISTEDLMKFGNDPQSGIKVVVNDCPAYPNISIVDVSLNASGKDEDNL
YALNPDLQ**KN**IRRGNTYIEVYDRSDYNLYKYIKLARKGLMKFYDLKPSYLVTDEDFRLSFKSETDKSKNS
KKNNEMNIESNLRNYIFSGIMKSLNDGAYIEVVKTLKINGENAQISWVKEEQVWCIASKNVGILAGSKSD
LNYYTGNNSKDRYYYALMIANTWFKIIDKIYKKGKSLAKLREALTNKTMVGEYVGNFYHQHLIKYNKET
IVFYSIVDNESSDKTCLLPEEYYEIFTEYEFDCVPFERIGMFDNIESLSDSLFHENKIVSEASIKNEEEGAVIYF
IRRGLEDDKVISLAKLKTLEYRIFRKLREKLRNFWAKHENIESWNSTLQNEYDKSFNLFIKECKELIKGLDL
PNSFEFYKEFADHAYNSVQNDISYYEQLCNYYVEFLETITKEFEYDPKIFSSFVFSSKIRKNYTKILNKELK
ADNDSWAKRKPNTSYYVEETRKDKRTQKQQYKEAEDTKTRFEGKNQSNIKRIIFRCKIDCN

>CUFF.5430.1 calpain family cysteine protease containing protein
MESSTEILQHLDGSESTKKLLEENANVYDDDFPPNETSLYNSTNSKLSKEELDLWKQFVWKKPKEIFGEN
FVLYSDGIDPNDIIQGCLGNCYFLSALSALAEYKNQI**KR**IFVNDEVNESGVYIVNFTLGGENYKVLVDDHF
PYSEKKGRPAFSQSKGKELWVMLLEKAWAKVNGNYENSIKGFVSEAFRALTGAPVVFYKHLYIQDIWDE
IYNADQRKYIICASSGEGQLNKKRYDEMGLISEHAYAVIKAKTINVNGEEVKLLQLRNPWGHKEWLGKW
SDTSELWTDELRQLCGCKEKDDGVFFICVEDYLSYFRTTVICKLHEDFQSNAIRCSHNLGEFSLIKITIKSK
GLIFFTVSQFNQRWVRRSQEYEPSFVRMLLSKIVPEHEKDQMNFPLKFIEGKCWKDEDTTIEFECEPGEYL
AYVEIHWFNDKQFNNFVFRTYSESVPELVEVKDKENEYPDFLKDTLKSCARDSSSKKTYKEKEEPDIFSKI

>CUFF.5438.1 cullin c
MFRDSQSKLIDGLMNEFRRFREKEDVSWEAIHKVIGTFVKIGFKDKVGFKKIGENFEWTGDKDLKEYEDY
FEKRFINATRTYYTAKANDWVEEYNCPEYVTIVSRTIEVEEKIADQHMESTTKDLLVAVLNTILIEENAQV
VIGKEETGVKDMLNNKKFEELKELFCLYSRVESTFKYIIFEMEPFIELKGTAIVEDQEILKDPIKFTTKLLDL
KREMDEIVITCFNNHEAFNQARDRSFLKFMNKFSDTPQYLAEYCDNLFRVGIKGMSESEIDENLNAIIRLF
RCLHNRDIFAKAYEKYQALRLLNKSSLNSNAEQSMISKLKIECGFNTIQKLTRMFTDMDLSKTAMVEFAK
KKGAEIKGVEINVDVLTSGIWPEQIIHPLKLPSELNECR**KR**FEIFYQEKHSGRSLTYLFISSTAVINTLFCPKS
YSLSVSCYQAAILMCFNQQEVLTITEIQELTLLPEQELARQLKELCNPKARIINKENLKIPKFESNEKLSVNL
EFQNKLIKLSYIPKSSYKKKEVGEKTDISNKVDEEIKSERGMVIDAMIVRIMKARKKEVHVELMNEVMKQ
VSLFKPQPQMIKQSIERLIEKEYLTRDQNDRKIYIYIP

>CUFF.5439.1 microtubule-associated protein tortifolia1-like
MNKKSHFPAHIETKHQFRQCLLSALSKFNNNYTLATAIEEIKEIMVEHITNTDRMNTFLYHIAEQTDHLKS
TQ**KK**EYIKVYGIASEIFEESLIPFLPKVLSQIQQKLKESDVQEIHNAYAESIGVILHNVLKNIQNIDEATELLT
TFLKMVFTNLNQPGKIVQSGAALCLTKIIQNAPVEALKLKLEDLCQGLLEILNSTNCRAHTQILESLISLLLS
VERDFEPFAGNFLPILLECMGLSEWHTRKIGIDVMYTIAAILGDVIAPYTKEILEVLNHCKFDKMKPVREA
AIEAINLIKDLDPSLIVEDSISQDSLTRRDKVMRSTTNKPWNK

>CUFF.5488.1 WD40 repeat-containing protein
MVDIELTETETMTMFFIPRTISTDPSDDSMKPTEEYKKLLVQKIGADSYTNRGVQTLNLAQKTRDVAPEN
KIFMPKDVKVQVNNYEIENENRKEQEDEGTIMIREYEKSIEDIVSDKLSEPRALIDSEELASHISIYSQATTK

SDKEGGKTGSKSGTSKNNPSKKVTSSNKEGEGATSQNLKESDTKSGLSDKNSSSSNTQAQIGGQSKVSGL
PDSTIPAKMEFTDSEAIFSSTTILKAINIIERLLTQTKYHEQNVLYTDYPVVKITKASDDKDGKEEAKTNKN
KFDIAAMVGEEAQQEEEESEDEEELARNDPESIRIKPLFKFQCEITDGRNITCMDFNKSNHDLLAVAYGEY
DMNYKNSQDKQKGIVAFWTLKKPNFPEKIIFTDYSITALQFSKLTPNLLALGDSMGGIMIYDVQSETLDPI
ADSREIDEKHTDVVWEVKWVEKPSKGESLVSISGDGRVIEWYLKKGLEFNELMQLKRQANSSKKETNV
MPAGIDLEKKTGMTFINTGGLSIDFPRGDSATYFASTEDCTVVRCSVSYSERSLDTYAGHTGPVYKVRCTP
FWSTDCQIFLTCSYDWTVRVYNHKEQIGKQEKLCCHEQYLQHQVNDVAWSPNTSSVFASVADDGRIEIW
DLFKNNLEPKLIWFDKINKKTFSNTPKTCVRWSRQSPVLATGNSKGVVDVYRTFGLEHAQVSQDDQIKRL
LSSITTNDFSDSKGDGPNAGTGNANS

>CUFF.6125.1 Acylamino-acid-releasing enzyme
MFSKVNLQKFEPRIHSNLYSLSYESGVSTKLNDLPDNEPSDINVVVISEKKGNKVIIRKEKNNDKQMYLEH
WDTDGLSISLKLKDFTKIHNNSVFGTISWSMNADKIVFTAERQDISDYTPYWINENQKKDEEKSKNEKDK
EKKTPHAFDKYKYNNKNSNHMSSYGEMLSDCKYSVIVVYDLIKKKLTVLDLMKMKEDGSICSDIFDDV
HPAFPIFDKSGNGIIFHGFHLPIDKLGLIYCFNRPTKLYYLKQYENNEEVKEKESDSSKIIFETEILTPDYYFA
AFPKFTDDHKYLSFFCSKDKFHTHTTSLGLDVIEDLCEAEKKHEIHTVIKRDYELNDIFSGLFGFHEKYNTK
FIEGTHIMLISTVNKGKEIVISANVETKSYKILEDPYNPNNCIDILNVQDEVAFIISSSTSNPPCVYVIADIESE
ECKWYKLAQVNCQHKSSEIFKHISATLNNVKIDTVSTKDGAEGYFIRAYDTESTLNIYENQKKPTLLFIHG
GPHSWASKNVFDKQRIFWLCCGFNVCIVNYRGSAGYGLDFLNSLSSKVFEYDVNDTLELFSMCLDTFKD
EIDKSKLAICGGSHGGYLSCAIISHPEWKDKFKAACIRNPITAMFMSYAVSDISDWHYSVALGKENSYYA
KREEIEVMYDKSPINRVSNVTTPTLFAIGDSDLRCPKYGGMQFWRAMKANGVETELLLYPGEGHA

>CUFF.6668.1 MAPK-related kinase
MPEIDDIGGVKNRKSDKKIKEITSDDADFATIEDEVGHKYQITSQIGAGSYGNVYEAIERKTQKKVAIKSIH
SIFDDLVDCKRIMREIKLLRHMNCPYVIKLYDVIPPDNRKSFNRLNIVLEYADSDLKKLLKSSLSLDEIHVK
TIMYNVLCAMKYMHSAGVLHRDIKPGNILVNEDCSIKLCDFGLARSISGVVTGIDLVNKIVKDIEYGKDSD
KYEEYKDYVESRKATIDEYVDFDKNGTRTYEEKKDIHFKLQKSKLTRRKMTRTLTGHVVTRWYRAPELI
LLEKSYGHPIDVWAAGCTFGELLTMIPSNSTSSLQRRPIFPGNSCFPLSPAKRPILEKDDPSLSDHHTDFPID
RKDQLKTIFNIIGTPKDEMDVSFISDELAEDYIKIFLKKPGISFEDKYPNSTEESIDLLLKMLTFNPFFRITVDE
CLNHPFFASVRDLDKEVTAPSEISFSFEGEGDLTESRLRALILEEVDYFN

>CUFF.7003.1 UVb-resistance protein uvr8, putative
MVPVTKIACGFAHMLVLLENGNVLVSGSNGQGQLNLDPDKTTEVEGLLLHEFLCKKYDVIDIGCGSAHS
VFFANLQKDAVKKEKKILACGYKGCIGVADVSEDNYRLQSVVFPDLTDFDKIKYLICKFNSSAILDVDGN
LYYWGDDFDGFRERTPEKKVMFKHKIIDISFGFRHAVILLENGEVYTWGDGTYGEISNESKALESTEPLKI
HYFDKNNIEIIKVEAGERHSLFLDNKGNVYGCGLTKGASNTERIHSPIKIEDISDFDCIDIFCGEASSYAIDSS
GIPYKWDGVTGKLEAISDVSGRYIYQVAVGNNNVVILT

>CUFF.7028.1 Nucleolar complex protein 3
MEKQKKRDKKFKQKQTEEKKNQTKEKEVVNKRIEDKKNSKATLTKDILSVASKDDKISLIKMISNEIASSI
EGNYKKLDDLLLLCEDTDVDVIIYSIKQITLVFCDILPDYKIREDQDTKREGKVTLSKEVKRLREQETFLLE
SYRKFLQILETFSKFKAVTLSETLQNKYMQIKIESFDSYAIFLNRLSHFNFIKNVIKMVASKLASKIVEINKK
CTKAIFDLLSNKAPHTQELKLFAIEELAKQLRTRPHILFQTNILECLGLHNIIIRKEDIALLNEDTLKIEQLKK
DVRKKFRKGKFSEAREQKLEIIKELKEVDATGVDIEKSSNLNNKIIMAILGIYFDFLKNRATSPLLKGVFLH

LPNFTTHVNIEIVWDLINVLREYVQVELKRKKLNLSNLLTALLCCFQIITIGAGNAFNDIEEKDFSNCLYNC
TILMMNEISTLHIEDLLAYLKTINIALIQ**KR**QFSNDLIIAFLKRLSLLALHSPTYFQCGILLFIKKIVDKYPNV
KEVIDFNYMGNEELTTKEDERIMTNEDPQLLVNIHQISIYTAIGK

>CUFF.7485.1 Protein kinase domain containing protein
MDKYRKIKRIGIGSFGQALLVQSVVDRKCYVMKIINVGNMDKKQKQDALNEVRVLKAMRHPYIITYRES
FMDKKFLCIVMDYADGGDMY**KK**IEFQKKISKLMPENQLLDWFVQMALAIKHIHDNKILHRDLKTQNIFM
TQTGEIKIGDFGIARVLQHTYDCAKTAIGTPYYLSPEICQEMPYNQKSDVWSLGCILYEMVTLRHAFDSNS
MKGLVLKILRGTYPEIPSHYSQDLKDLISEMLIKDPVKRPSIRKVIEKEFLCSRISQLLTNTIAKHEFSDTFLK
KHLTPITENKENEDDESK

>CUFF.7729.1 hypothetical protein OXYTRI_02888
MVEIAISKLSPDERKMCTDIFQFQVISSLQACQICDVDISKSVKVICCGCKSADKNSGPFVLCLECLRKGRE
KNDHKKDHAYSILDRLDFPLFVKDWTAQDEITLLKGISQSGIDNWPDISQQSGFKTASDCEAQFYSFYYKS
QEDPIPRIEEICVIKRDKLSNSPFLNNEILNFNTKKKEEEIKSRKPAKESRQRSSKNEDDNISSEQSKKQENPI
NNVRSLVGYMPKRGDFDTEYDDEAETRICEMEFNDDDNEEETRLKFQVLEYYNARLDERIRRKKFVIDR
GLLDL**KK**IQKQEKRRSKEERDIINAMKPFARFSDKKEHERRVSNLLKEYQLRVLIGQLKYFRSLGLTNLD
QIESHIEEKKKEGKFDDTKDGATINFIKKQKTYIGTETSTSGRSRRAPNASVEIANASGFSQLTEEEKDLVIK
IGLMPDQYNIMKNLFIKEFESNGEVKESFINKAAQNVDEKSNAQQACVVFNFLVGNESIKRAPHEPMEE

>CUFF.8052.1 hypothetical protein OXYTRI_13751
MINMKKQKLDDYERRKSDFILFKGNQRKEQANENLQRKDRDISDAIHKHEMYLENQRNSYLRKEQEKD
DKFNEFQR**KK**NMQLINLKERSNSRSLNIGRTIEKARQIEEERKQKIINKEIERDERLKEHLQDTNSIKQSQS
VQKDYYRQAVKLASHKIEEEKRNTYLKMLESKEDRFRQSKSRRDDEYKTKLDFTSMSKSQRQDYIKNFQ
EKKEKEKEEKLRRLMLKDEYIDEIKKNREEDNRLRSVIHVQNKFILNNGKGSALRNPKLANSSSYIIGASN
RVYDSSSFTSNNINLMTVEAKPVHKKIRDSEIE

>CUFF.8161.1 UNKNOWN
YISKSEKLCNIIGSNFKSSLYENFFTKFKPRHLEYFPHVNARAHNCGDQSRSNILNNEFRRQYNAYLVNSIE
KTKLDNSDKMCLTYFLLLQDRINEAIEIFQQVSKDEIRYDGGLQLQFDYMTAYLDFYLGCDTNYTVARDI
VEKYKDYPVLHWKFLFEEIKDQLAEFDGDMDVDHEINQEDEDSKERNLKKSKGLEPYLSAELKN**KK**IHI
DFANIQSIEIKYYIIDPEILFSSSPFLASDTEDFAFVKPSETASIILNPTNVSETIELEKSLINENMIIEVNGEGKQ
IFLRYFSTSLKVHINENYGELKVTDKDNKSLCQVYIKVFSKSKSGEVKFFRDGYTDINGKYEYAQINSKNL
SDVEKFAIFVKSDDYGSVTKECIPPQTIEKNEIDSWYANPMSFNRKMYQKIRHLQNKS

>CUFF.8183.1 Oxidoreductase, zinc-binding dehydrogenase family protein
MENQTFKAIVTDHTSIQVVDAELREIRKGEVLIKNHSMPINPNDRYIVSGFFNFGVDGEFKGVGFEGAGEII
EVGPDVDPNLVGRKVAHIGNPFTPIYQGSWRQYLYCEVGDIAVYPDDADFDKMSSTFINPFATLAMIDFT
TKKEAKCIIQDAACSSLGKMVCKAA**KK**HGIKTINIVRREEQIDILLDEGAEYILDSSAFDFDLDLRTMIRDL
KPSIYFAAVGGKLVEKVMFMMPPSSTIVLWGSLENKDISFSPSVFIFSKLTITYLTMFEWLYMLTKEQKKQ
CIKTIVEDICSSDSIFSSKIVKTFGFDQIEDAIKYSIEHASEGKVILRAFD

>CUFF.845.1 DNA topoisomerase

MEKKGTAAGSTKETDTKYSTTIRENQDSTEGGRSDEESKSEQMDESLTNISTARPTYENEFLNQDDYTED
EINEYKESLIHNDKEVEERFKNICERKGKLNVVMIAEKPKIAELISQVLSKSQA**KK**ELWKGFRAYLFNNIR
FKGFNANFKVCSVYGNIYDVDYERDQDLWKIDPVKLLSEKEIFTYPVKRIRNKKKNWRKRRQMTADLRF
LEEYLGYYLKDADVVVFWMDCDKVGENICFQVIDYIKNCVPKFDLENVFRAHFFSLKHQEIEKSFNNLH
MKPDEKRSLGVYARMTIDLRVGISYSVLNNSKLNKYVTKDKNSKNLITYGPCQFPTYWFCYERMKEIRN
FKPKEYWVSILEVLVGEKTIELKYENEEFSSKIEAKEILIAIKDQKSLKVVEISEQKHTIYKPKPMNTNDLIR
MASNLFGFSSDKTKEIAEWLYLNGYISYPRTQGRKYTSEEEINDILNEYSNNRYYSQYVARVKKCFEYNF
DEESDFESDHPPITPKANFKKMTKLKENSKELKIFDLVMMNFFASLSIDAKYKSVTTSFNVKGKIFKETHK
ELVEEGFIEFIGPDVLSSEIMVFEKYSFKENQTINIKRKLVKKCQTAPKEFLSESELIQMMEINRVGTDGTIP
KHIEKIVERKYVEVKEIGGVRRFIPTPMGEILAEGYLEIDEELINPSVRAFIESCCNKISDHKKDNEAVINKAI
EIFERKLRNLIKKFSKIEKKFKNLHKIVKEKESSLKIINQYEGSRVGGIYVLGSEDI

>CUFF.901.1 GTP-binding protein required for 40S ribosome biogenesis
ESKGNPYASILKHEEINAPPRVIVVQGSSESGKSTLIKSLVFHYTKQKVNDIKGTITIRTNKNNRITFIECPND
INSMIDLAKICDLALLVIDASIGFEMETFEFLSILKNHGMPSCMGVLTHLDYFKENKELRKTKKRMKLRFW
KDVYDGAKLFYLSGLMYDRYLKNEIHNLARFVSVSKYVELSWRKKHSYIVADRFDVIHQANANKSNCL
VSFYGYVRGTYLESNSKLHLMGIGDYPITAATRIQDPVPIEAKKPAHMKKEEFEQQG**KK**KRRTLKDKERI
VYAPFSSLGALNFDKTSGYISIPDKYVVFTKTDEVDQVIPDNEGQMMVRKLQERKFKEDIEMNDNNELDI
LDGVMYDQKKDKEHEIEKRNENRRVVLTDGKIKEYASRKEAQIVNNTEPKSKDSFLYKENKNKSLADLI
YDFNPTEEDSQDSEINRIDTTNAKLDDAPCKPIIYKRSGANFKVLHNTVNEQIDEEYQQKTVFMRKLYKLL
IKRKFVTGGAKVEGLDEMLPSDSDDEELKKNLAHRKIKVGDESDEEDEKAYVKPHLRNAPTKEEFIKIKK
HQDFIGEEYGFIKGTYIRVEVEVDKEIAMMMDPHTIVTLCALEKREESFGYMRMRI**KK**HRWYPHILKNKD
PLIFSIGWRRFQSIPVFVTEDQNERIRMLKYTPKFGFCYGVFYGPLYPLSTSFICIQRLDDKISHFRIAANGIIV
EQNQNFRVMKKLKLIGEPFKIFKNTAFVKKMFNSELEVSKYEGAKLQTTSGIRGQIKKALVTAGVPPGSF
RATFEDKIKMSDIVFCKSWVTLNETEIPKFYNPVYSYGNTRLLKMTSEVRRENDIELKFPKDSQYIMREEE
EKLKQIRDEKVNMPLHVPKNITKALPYKAKAKVKLIHDHEEEDRRRRTNLLQRLNLPSKRPFKSQFMNDS
DKKIYSLVQRLGTIGKQKYKENQKKLGVISEKKQKEEAKREDAIKKARNRKRDHKMSRKK

>CUFF.9040.1 UNKNOWN
MIEAMCEHNLAKEFIPGIADLNMKGYRLSLMNEDFSDIKVELIDSCQVLSADIDRRKNHDLMKIDLSYILK
DFSNSLKVYWP**KN**TQSNTKIKLNLEVVAKITTDYKLNLEDDKGNKMIPFSSKNNSEIHYVRFEGLCKEMN
LSFKDITSLKNDYYSGDIDISDWTITDFDRVLFDNPHA

>CUFF.9615.1 casein kinase II alpha-2 (macronuclear)
MIASKSSVYTDVCLAEGKEYTEYEDYLIKFGVQKDYEITDKVGRGKYADVYSGVNMITGEEIAIKVFKPV
KKSKIRREVKILHILKGGPNIMEIRDVVRDAATKTPAIITEYINQRDMD**LK**KMFNKFTLDDVREYMYQTLL
ALDFTHSKGIMHRDIKPHNILFDPVNRHYRLADWGLAEFYKPEQEYNTRVAALFYKAPELILNYPYYDYS
VDMWAIGCIFAELIYQKHPFFSGKDPLDQMLKIAKVLGTKKLGEYAENFKIEIDSSMKEMIGKHSEKPWEI
SKLIDKTLANHEEIHTNVVNLLSRLLQYDHTKRITAREALDHPFFARRE

>CUFF.9750.1 cysteine dioxygenase, type I
KSTLTKSKKLSLESLIKIMNEGLRECKTELEAKEFGLEVMAKYDYLHDTELKKYMLGDPVFGKYYRNLIF
RNDLFTMKNLYWYPKSITAVHGHAKSDCWVICTHGELVERVYKVSDG**KK**EIIEEKHLTPGGVTYIHNSIG
FHQVENVSSKTEASTLHCYHPPFDSVETLDNKDKMFDQVLT

>CUFF.7897.1 Leucine Rich Repeat family protein

MNELIISIVNFIELICKEAESTSNRLHKYFKEVSDVLNDIGRENYLFKCFVIPDDEVRLALVNCILQVPLKQL
DYQEISNLMKTISESKNIGAGKAEEIFSTIFLILINLVTDTEQTAFKNFRIKDGSNAIFHCLEILHKNQKRFIEE
REEAEEKLMLSISCLFFLKESSISPEMKVHMIDNQEFAEAFQEILISEQNFTFEFHRHIPIEIETTTLGSKICNL
KQLLIGKKQLEPMGGVSFRVIQQIANVLQNIELNKSTKLKPTPGKTKIDCMRQVLIENYEKRIESELDLWN
DKDEEAYIKSIARDRKLKERMIEEHKDFNDGSGLYLLILFLGKVDKKYDEAFNEDSNINSLLKREILTKIYS
QLESYIEEYKLWVKYKKNPQKGIKELILLKMIPEDYKSSRKKDENAVDETKLDMSLQ**KR**IPQSVSFYQRE
YIYDNSLYFGQQTKEKKEKVVNIEYQQKEIKQKIRNCTIAAMLRIIYASIAYAPHHSFKKKLIRQLYDLEIF
KKITKFCYETGWIKGNIQLKFLKIVRNILECDEDFDKEIIKGGAKTTDDEKGNKSRSKSVAKDLIEKRLSFY
EIVSRAIAQILINVNTAFATKTSNSLNEYTVEIKILVEISTCLRLIIERCSQFKFVDDSTEDIQEMTSKDYHKE
HKSRSHDKNLVDKESIYIEGYGAADFRQPQIRLLDWITSTLISKECIVQLITLSLHFKEDGMEQKKVNYKK
EFAYDFISKQISPFEVHEKVVIESDDEEEKIETGGATTKTKDAHITDTKKST**KK**EDEEVNVLPPEYIEFACFK
DTLKILVGQYISKCKKYQYEIIETFIRMSVFDKKELKKEYLEEIMNFRSLSKYLMQLERYMRNKKMLKYC
YKSETKVDVTTDTTKTPQETTKGDKTTKDLDDEDNETLIHTDKCEFKVEQESYEKKIICMTNKGIYILKPS
SKESCKICPAEMFCPEGPTFEYSWSFDQIRDIYHSKLKEDDPMNTFIFEVPQEKIATSGGGGFFSFWSSSETP
GETKGKDGKYYKDEVLQKVYLKFE

>CUFF.7816.1 glutathione synthetase, chloroplastic

MNSTSSEMLDEAIKQDIIDYCMQNKICVKRIAVPSSENLSTNIEENKVIYEDAHVPLAVLPSPYSKASFEKL
TENQIYMNKMVHNLMCNMPRVRELLKDLGSKDEFVGGLLRISKLVEQSEHKQKGYLAVLRMDYMFDK
DLKEPKMVEFNTIASSFGPLSYGVNCLHRYLVEKYGVDIIMENLERKKNPKILIAESLKQAYNLYFDSDIN
DLVDANKGIIITVISDEEKNLFDINAVLDTVFESYGITNKFLTFADINSNCVLTDGKLYFDDKEIAVIYFRHG
YDPSQYLSESDWKAREKIELSVAIKSPSIDLQLLTL**KK**VQELMSIDSIWEEFHGSDLDNIRPFFYGMWALD
KEDCFTTKIINDAISNYDKYVLKTQREGGGHNFFGADIATQLHKEDELWKYSLMRKINALSFPATLMRNG
FVWSGEAVSELGIFGTIVAKFDETYEVYVSNEIGCMMRTKPKNVDEGGVNAGYA

>CUFF.7652.1 WD40 repeat-containing protein SMU1

MEVDKKIVLNMIYTFLEDNGYAVALQTLQKESEHAYIIPKIKSGEWEFVLHILSSIRVSNDLLYEVYEQIVF
ELVEEDDGKIALILLQQPIAQKLKETHEDGMKKLESLCTSSISSNRNNRVDMKIVYDQEEKGDRQDRRER
LARMFRESLRLFDEDNEQFGIFKLLAIGNHDEDSEEIGIERVRRGPTKGKAVMEEVEDLTKVKIRKPYEIINI
IDKAKENIENQREIECINLCPNSKYLVVGFIDGNIEVWNKYTLELIKEAPFEFQNDKFMGHSCHVLFLEFNC
PEFIAEENDDNEKIEEQKKSKRGKVLASGDSKGSIKLWNFFNGKLI**KK**IENAHIGNGITCIRFDDNTSCIYSS
SFDSIIKVHGLKTGEMLKEFKGHTGPISDFHLLENDDRMISSSGDGTLRIWNLISTFCVKIINPLSLNKSEEN
LINVSIISCHMYPDQEDKEMKSWCLICYEGSNISLLINTKTNTLINSYHNDKQNTTYLDSYFDEEGKYVINL
CSDNCLYVFDTKSGKLVAMIDYSDKNGQQYCNSTIFCKLIGLQY

>CUFF.6618.1 TPR Domain containing protein

MEEHNIILLKTEEARLDQLYHSRCDENELMNFTQFLLLILERGIISNKFGIELFYTTFREASNGEPYLDKKKF
NYAIILLSKTIFVHEENPVETMFTTMLMDKTITYTNTLVGGRIPKTDEETLAVLSEEAIIFYIAYIDRLKMLF
VNAHHQNKMTNGKGITWKEMCDKKDCGILAGSFLNFCKDYCLIPHMFNVEALQEILLSIIPPLQKEEYEY
FNQQKIISQYEKDKSCISTNYEFVLGEPQLLFHEFMFALGKIAYTTVTVSDAETLTEKLRVLFVEKLHFMEI
VNPLEYVEKYLMGTLEQEGELYSSEEELEEEYEDDPHQLLLDFIERRAERDQNFVIDYKKVLNDLDVILPP
IPDKPKIEVVNPPPYSIPRVLFGKRLPKPEEEGKDKKK**KP**QPKKKPVKSKKDEKPKKIYPFAEFPPKPEEPS

NKEHLDDYAEVMEESVFPIHYRASHCNSGVAPCIIKEVLFPPEAPQEYATLIESALVYQNTANYEMALATF
EEVREKWRTEEGVKSLRSEIELFFELSLGSVYESAGRDELALSKYLSAKEIKLVYNHPDQAFPYCGIGSVL
YHMEEPKWALRAYCKAREIREERLGGDTVDTATVYNNLGCCMLMLERNQEAYAYFELASAILEVELGP
EHERTLTASRNLLKAKRSVLNVKPEYKPLWSFAALNPMPKLAKKKKKGKKGKKKK

>CUFF.6032.1 high mobility group protein
NQYKSSHLEYTFGYKPLLHCKCKMDKNSNSESNIRRSSRINEKKRTNKINYKELEEGKEYIQDFDLSDNLN
QKQYEEEVNTKKKKEVKLCHPKKSLTAYTLFVKLKRKELQDKYPDATTPEMMKEIGRQWKIISEKDKA
WYQSMALKDKERYRKEMDDMNKLKEFHKLDNCELKRPKKCLSSYMIFVREVRSRVTQEFPDMNALDV
MKEVGRRWQSISEEDKNYYQSLADKDKERFKRENQQYMKELEQLDTKLKSCKKNPVMTGEDAEIEDEN
FVQGNSKSFEEDSKVSIGANGKKMRRDPNMP**KK**PLSAYIYFSQETREQIKKENPKMPVASIMKEVSNRW
AAMSKSEKDPYINEAREDKRRYESELSEIKSKPGFKNIIIEEEKEDFIEEEPEVIEAQLDNKRKKIKHSEDDS
WTDPKLNNRGKKKTDNKVKINLIDVGVKQKPKEPEPKKMELIAEKPKPQTDQQFRFNLTENKQMPYPN
MNQPSPLPIKRDQISFGATSFGHPDTISGTPDHGFSGGYMYKPFAFSPSPFGQNRSPGPSPNMMPMYSPMM
FRQNDNFRDYPNRNMGNIPQTPGVLRPGETPNINRHDTPMLWDEGKIPSGGLYQTQNKPPEQYPMFSPGG
AMENPNYFNPSPNICNFSSSSFGYRRTPFTNYARTGPLFQGVNNQMPQGGNQRNPGNDSDRIFELNPFGN

>CUFF.5628.1 DHHC zinc finger domain containing protein
CQKNLMNMISENLIHFRTPIEKKRLKDMVSIFIVTHIEKKRSLKTSVLTKGFIKHSRFVKSSSCHKEKTKHIR
NSSVGNNLMTQSNQQVANWGYTKLNDQTFKSGDFSKIKTEMEDVDLFETNPNEQKGHHRRKTSTIKKIG
TVPQYDLFDNEAKNPERDKVFNMMAERIANSRATFETEQVPHSSPLSGGDTNSIFHHKGKGNEENQFQLN
SEDFKGVLKSFKDGEESTENKQLPLASNAVTNDTDFVTANNTGLKKRIELQDFEEIKEEDENYENYDVKK
HHENVEKNHDYPNIEHNKRCVSDIGRKSIALKMQQDDLEFSAHDEVFKNQDTKEDDAECEDNDANMIFI
EQKFCTACNIEQPLRTKHCRTCKKCVATYDHHCPWVGNCIAE**KN**RKYFFCYIWIQTIQVLFGLIYTSYVLS
TIVVDLSQLKFYMIILSLLSEIFFALMLLSLIIFHTYLILNNFTTWESLSWNKISYMRLWPRKYGSPFDRGAS
QNIKMYFCEKSKDKITIWKMPKKLPSIQQGERIIKSRRWSYLLEKLCVRCQ

>CUFF.4268.1 Haloacid dehalogenase-like hydrolase domain-containing protein 1A
MEESKKSVQNIYIELELIIDIYSLLFSIIRIDLINKHNIFIINKDITYLYGKNLKQGAMDLLRYVGNGSISEKEI
DLFIIEVIKSFVETFSSTLKPTVPSIHVIKELHKNNITLSSFTHFNIDDLQEIMKDEFIDMFECIYPFNEFLNSIA
SINKEQSLIVFGSRNKFNEIKSVINEENISFINVKLPMEVDETSLIQEYKSLDLIPFENYGLLKLSPLFEGSLHK
FDLNKDLNTPELQFWDKTVSIEGILLSGKVVHGFQRGSKQLGVPTSNIEMTKENIDLINKLLPGVYSGNSY
FINS**KN**LDSSIKYRAAISIGWNPSYDCVERTIEAFLLEDFEFDFYDEEMCIEITHFIRAESNYSCLDHLIMAIH
NDIETTKRIVKI

>CUFF.3916.1 retinoblastoma-associated protein
CMEGRKFIEIIVRCAVLISFKVQHHDGSSVEDKNSTPKSKKDSSAKKSSFQITEILKSSQTENMDDFLNWLK
EMISDISLDIKTIGDCKEFIRNFGFIFSFYDKYIRIWNSLNFKCISENINKSDLNEAQTLERIKKIGWLIFILARI
NILRNQTEIVDCAWMLMATMYVLLINLDHTEITCSIIDECIEEKLTDKQTQLRIFDKLCKFFKVTEIEPVQFS
IDLLISMLEKLKE**KN**VIITQSCITNNNIDVEMEDEEEKVMSDNKHETKRSSKCSDEIKGMFDHVNASSNFD
SLYKEYSRSMTNDQLDERFLFEECVNINPVNVTPFSRQGNANKLKDPKLSRKQNELTQIGNTQLTSKRLL
DY

>CUFF.3863.1 deleted in lung and esophageal cancer protein 1

TVCTKFHSKLQINLSFEDTSSTDHQLRKADKIIYREHFSIRKVKFPSKDESIIAPGMSIIMYISFFAPSFADFD
DVLVIVTEENSFEIPIRARREPPVIKLVNPMDSKSCWIGDRVDMVFRCTNTGGDGGFKFFCEKDEDDSKQE
EADTIKLGPFTLFPSEFYLYSGNALDIFVSFNPVNEGLSEENLILACDNHTSEFYKLQGYGAMLDLDVIQVD
GRDVDTKLHPLDTIFFSNTNPQAQTCRVITI**KN**SSPILVSYHWSLYKSKTSHKIVLQDEEIHYRVEPNQGKI
NGGESQEFKIFFFPDHAHPYYEFCDFIVEDIPINSMRNPSQALKIFAENNTNETKIPMPTYIGSNTQYLSIPFL
QFNLKGQGKYREMFLDPPILEFKEDLYIFKTYTRTIMMKKKLKSEDKSLSTDNNGAGYKKSIRVEGKSDE
SFTVDIDSKHFNESTSSEEEIDIVVSIQSKTCGVKTAYIMIDIEDGIPLSYFIQAKFIGPLVSIVEPDVEFGLQK
VNTHTSFTMNITNHSPIEAPVLIKNSKDFTSYTFEKYLEEYNNELNENMKQERKIKPKSHKSVRLMTTHIG
NKITFQPQYIVIPPDSRGEITVTLNCTHEEEISEILEVLVQNSQSQYIKLNANIQKLKVCLNRYSIDLGKIYAG
IKQTIDSSHPQAIILKNYGNIPAKFQWNEKIVPDQLKTMFEPARGTIAPHSEFVVNVRLTSYVGGDLSEMFI
CDVTDLDFPLGFCLTANVFGLRVAHVLPDFVVEAQKAALAMNMTSTKSKFMTSTIIKEDFEEPEEITEEQK
DEK

>CUFF.3841.1 sperm flagellar protein 2
MTELLANWINNELELSKHVENFEKDFSNGYLFGELLKRYNQQDDLDSFSKKENRDSKINNFTLLEPTFRSL
KIKFNSQIIDNVMKEKRGAALGLLCQLKMALEKVYQPTDISVQNRTGKSSDNKPS**KK**LNPGKELYDNRA
HTFFQRRLQEMNRSQKGINLDDHLQKFEEIRMKQEEQARKGDKDEEDMNTRMKQDMRKAQIDKLQRN
AGFMEEWLRKGIEDWKRNQTIKKEREKKTLEFELTQTKKIEKFTMTQIKSAVSEVIDGIGEFENNLRKQGI
DPELPGSNASSPTKTRITTTQSANKFSKMTGGLNISATIGAETGGAIKDRGNRMSEDTRKERQRRRGKLIK
TLNNDVLRDLENQTREDQYVKRLKRQSKQEEELAYEIWRTQQCKNVIIENRKLREARYFKRKELDTNNA
YAREEEMLRTLDEQRNIDEESQQERDELLRINNSQNKRQNRYANCLDMFNEIFEIANQAYILLQKSDSEDI
DQRSWREWMNLFKNEKSIKETYTDGKQDPDHDDDKVIGQINIEVHEATTVDSDKVLDDSELDDYLKNK
GQWTNWLVNGEENKFNVADIFAPAEPVGGKGAKAAPVENKLDEKEMTIPEELPKNNLLGDVVEKIICLN
YEGEKDIVKPNIPSHVPLRISIVGTSFSGKKTQAHLLAEKYNLIQYHPYELINEAVERAEEELEPTEEPTQQP
IEQQNKIEEDGQPIEEGKEIVLENMNDTIKVKEEGKNEEVLDIQEDFERHGSNKESQSNQDKEALEKSRRN
MFRTIGKQIKEQLLNGEEINEILVVDLLIAKIKADFNYKTQEQVDEEIKKVIQREEEIKEELEKYESIKGKTF
KNSKPVDEAGLKQELQDLSKFSKFGWILVDFPNSIDQAHNLESKLSGYLPSIDREICERNRKLASACRIIEPS
EKPNIKSALIESGMDCVLWLDTLREECRRRALGRRVDIVNDTEYHIDDNPPPTTNPPLCERLMPVIEPERAE
EVIPDKHLAFDLQKDNLIRWFDKFGYEEENNKASKISLCHYVDGNSTNSEDIMNSIEILETIVNRKQQKWV
DKREKFRTAILEEKARIRQEEEARLKAEEERKRKEEEEERRAAERAARGEGEGDEPPPQEEVKEETKVEEP
PPVEEKVVKEELPPSKDNIDDDFAPVLMNIWDKIEELYVKRMKKSLNQYRNQRDRIVTGLFKTQKYFVQ
YLNRPDFKQAKLDKFVFDFNKFSDEYPDLREDTQTKEELHQRTDTLSDQLWEISEQRKNESVAERKKIME
NGWIEFELEQITSMAQNLMQTEIDKFRNSVYLIYDYYYAIEDRLINDPPESLHYDLMTYTDEGGSEELPPV
FNKEGDGPEAKENYPRLNKLYEKALKSQILPEFECTPPGGAGGDKKAPPKGKDPKKGPAEDEKQEKFYF
DQELKDAIATEKAVIRFRLTMVRNWALNLMKEIRSKSIKSFDKLATWIEVAFKAETDAILELEKVIKRSIEK
EEKLQFELRIKGMDFHFDEKFLNFQDPPPEIFPAREEPALNRFTIKQLESLINELIISSENGMIKNEYFVDLM
MTKTKNSSSFNDNNGVPQLLKNLTKSDYELIVKSFDLILSGSISLKKIAITMCLISSTLPDESELREYRDKLY
ENGVELENNRVGVKKYDFMNTPAWFDKNEISIDRPQSYPYPRVKNLKSIIYDSVKNESGTLDIEEYLSLLII
KVPGKEIKQYSNVLNLQI

>CUFF.28922.1 Adenylate kinase family protein
EMDKNQKGEYIEGIEEYLEDYKVYDYFYELMKDIILHRPKNPIDFLIERISKSECYRCVIVGPPGFSRLGLG
RLVAGKIGWKYLSMTDWIGKEKELSKEKKEKKEEHKDEKKEEHKDGKKDGHKEEHKENLHHVIRDDAT
MKEVRDNIILPDAKDDIKNSKIYSEVRGVLSGRNIFLSDQQAITIF**KK**RVKEYEHESWILEGFPKTKVQALS

LGQNKTVPDKIFILKYSDDVAIEHILHNLKQKYGNEKDDFELAEIAKEQMLEYHTNINGVQDLFQNIIHIID
AHGYVKGYKDDQNKISIFVDEISRLIQTKRMSPDRKQRIIIVGPPGSGRSTQGQIIAKNYGLIHVSTANLLK
NEVRLKTERGKRIKECFAQSKLVPDEIICSLIESRIKQSDCKLNGWVVDGFPKTIQQITVLKAMKIKPTRVII
LECAKDVCVDRIYYRSFDPVTGKVYHTVHNPPIEQEIKDRIVPYFPDMTKDKIGKRWDHWNDFKFKIEES
YHDLVLKFNTEEYSIKEVTDQILEYVQNP

>CUFF.28154.1 hydrolase of the alpha/beta superfamily
MELNSLIFPAPESSYDEKMFEKDLIFIPRITSNGKESDTEFIPCLYLSYSQGSSKVMIYFHGNAEDLGQAYQL
LNHIRNTLKIHIVAVEFPGYGIYPGSPCSKNILEDANTVFDYLVNNVGWKSKDIILFGRSLGTGPATELASR
KDPGALLLMSAYTSIRGVVKNISGKFTQYLISERF**KN**IELMEYITCPTFLIHGQLDELIPYSHSEQLHEKCGG
PCTLVLPVHMDHNEFDFFDDLSHPFYFFLIQCNVTIKQKGELIFPQEVYEVPKNISSKGKKKRACCSLTKLF
W

>CUFF.26802.1 hypothetical protein OXYTRI_15103
MAFTDEEMKLPEQNYANIDAEDDESNMDIYGEGEFTDAFFRTLMRVQNNVVNLPGGKEHFDIYLA**KK**IY
PVLVPGLESLSREVERLMQDDGKIDSAIKERFNPCIYLAEYLMSNNPKFGHQKEYTDIFEKYARIERNRRF
WSEQRQIFLKEFMGQSYQSNFTIQHISEFVDFIDKYLEAQGTIKDSIVVKEAYSFLEEDEVITFDKFYEIFSK
SLLDSEKISYDDIFETAEEQKRKREIEKFKRKTDRLI

>CUFF.25646.1 ccr4-not transcription complex subunit 10 isoform x4
MESISGESSHLAFDHFKKCNFDAVLEIQDKIKDLIDKTDGDDENEGFLRQKKLIETLVEYYKDNAKDPKK
AYKALSEIDNEDKQEPFCETNFLLEYNAGVFAYLSQMYGKALEHFCKILENCEEAELFLVVKSAFICIQILI
DNSYIDPAKLLIVKLEELLPLITKLIVMKQGYRQTLESDKKGAEDIIPSVGEGGLKLEYFSVSTGSYLSQEA
KAPKNPCTLEYEFFIIYFKTRIAIIDCEDERSRSKWLKILSDKYNQLEKRKEEKMPEMDEIVIDQAHSFLPYI
NSFSAMQDIIVRTQKAGSTETTQLDGIRLMSFNDRDLSKSGPNAHASCFKTLKESDKRKEQIIQNCNQKHP
IHFFNNLGVLHLNLKKYGMAAFFFSKALKYLSLDGKQNSNDLKSPFKFVSNHTSQ**KR**AEIMNNLGLAFY
KLKEYQKSMNCLSEAAELYCNRYSIWFWMGVCCVKNYSITIERVSLEKNENDVYHTKFNFTFPYNANKP
THSKTKKSKICMVQRPTMKEIEEKIPEKMRLDQAIKYFENVIYSNKNYEKNNDEIVDIIDKFDFKTIFANIP
KILPGEEAKEEILAIQKDEDKSSYSQGYQKKVYNDRYNEMFKYSYLYLIFCYLSLENPDKALEYCKMLKS
DFKLNPRINFELKMYMAEIYLLKGKPNEAFKCLKIDQAFEESKEAATDQSDNFIPIENLVSGVRETSLPKRA
IMFLNIATCNYFLNIPESANDAILNALDSLGYKSEGDKSVSKGSKEFEIPGFLIHALVYLNLYNDDKESALK
ILKRRRFDKGADIILEMGPQ

>CUFF.25563.1 pyruvate kinase
MEMRNYDAEDLLQEVDQTKRKTKIICTLGPACKDVDRIVEMIDAGMNIARLNFSHGDHKMHGLMVDKI
HEA**KK**QRPGNNCAIMLDTKGPEIRTGLLVDQKPIQLKKGQHLEILTDYSIKGDNTRIACSYKELPTTVNPG
DQILIADGLLVTKVRECYEDSVCVEVMNNATIGEKKNMNLPGIKVNLPTLTEQDEKDIVEFGIKRGIDIIAA
SFIRKGSDIDEIRDLLGIKGSYIKIIAKIENQEGLENFDDILEKADGIMVARGDLGMELPTEKVFLAQKYMV
QKCNNYGKPVIVATQMLESMINNPRPTRAEAGDVANAVIDGADCVMLSGETANGMFPVGAVLMMAKI
ACEAEYMINYELLNKRLIKFNLQPRHTPDQLAIACTNAALSLNVELIIVSTVNGNMPRFISKYRPKQLILAC
CTSHHVVRQLNLLRGVLGYRIPSLQGFDELVLIVIKAAKSMGLCKVGDKVISVKANLEGQDLRILDVEE

>CUFF.25352.1 viral A-type inclusion protein

MIDFDICIEKGPWDLEIILTFANQFRKMKCTYEHSCDKEAYSINKEDGSSMCYNHYSLCFQDHNEIKEKYC
RINEIVLNINSTFIEFQDTFINIKATIAIFRTNKVAFIGYDDMLDEVCKVEKNLDLQVAKIVKYATGTLNSDS
YKIYQLMIREMNQMTPDIIKINDICKQVIYRIFNDNLVQTLKDACMIENEEFKYDPNLSPVHPIFGKNLTLF
TMMCQNS**KK**VNEINEELCNKYEYVVKVENDFNDFEDFKNDLFSQSQENIKMKNVIEDRDNEIESLMETIE
LLKNKEEVNRLNDELKKENKYLCKENEYFSSQNIYLILQNKKLVQKSANLLSCDYQIKAKINRYFRKLNN
IDENEDTDDLDQWIVDEELEQKYEDLEQDENNNSEAIINKMKKLNQTLRKF

>CUFF.25074.1 UNKNOWN
MPSVMILIYDWSKDEGNISWSLREEDIKVCISKLRKRSKEAKLMTLHLCMNVLENRESIDNSPTNKIEYKI
ASLKKNTELEAKGIFLISSGLDGFTMLSKKFEKTMYEYSNSFYKEMKEVTKTKQRKIPKDDILNIRYNFKN
GYFTEIIKDQAKSVKFYQESYDLLITMKESGYSKYSSTEMREVGDLIVLKFLTCYLCHYNIESALTLFKKHF
DLFSRDVSKIKDKIKFIEINWRLNWMKIFGLMIQKTQLNKIDRFKDFWYFPGYYYLNCLHLMQQKVKIFQ
VNNYFIENEDEILKSIEGKDDPTHPLFTVKRRLWFDPHEFQSQFMIQENDFIGKNPIISKEPNPMVALDHDE
YKNALIMFKVYNELSFDYEYEFENMLKKTLDCYTNQEHAERMIDYIHSIASNFYFKQSNFKKCREMKSFV
AKRLARQSWNSVAVDLIENVKLCSQKLNDYESFMQSEFELNNVKKEDIEIKKQRMNRILDQFENARDKT
DQTFTLNNPLIRVYARFDRKRAEIFDSVTLSIRIISAINFSFNKLNINFNEKAFNKEIFDEDGGELKLLENNTF
QNDITIFIHSHIKSDLKLDYIVMEKLKDGKKLCLNITPIPDINIDNLIFGIPAKDEAETNYANEDNKDALKLKI
SDTRQKIDLKVSYKENVFLGELAPVDFLLKCRKGCEIVDARLELGLLEDTEEPPPYKGSSINSRKSTSITVP
VDFDASTGENPFDAASKNPFISNNDPDIYFSFLDPNNSDPQDNDERNMEILPADKIIHIPDFDEHKDINIRICL
RLYYEGSKKFKVALKYKIIKVYDESKSDPIPMSISKLVLMKCHPPFKIEFDYEIKDWLTNELNINDYEANG
RNYESFIKLPIAEKVPLSISIASISERPMTIQNVDIEILNRELLRKVSRNQFSPVHMFEEGDVVCAAFIIEPIKC
SNDTEQYGDVLIEWHRESGIDGKMFRSICRLPIPSVSIVSSPLCVDIKVKDTCFKLCETFPLEIEI**KN**TTEILM
DICYEILQSPDVMVSGERKSFINLAPLDSEVFKYICIPLKSGLISLPSVRIGKDEGFHFIKKADKNVLIS

>CUFF.24417.1 hypothetical protein OXYTRI_00559
MESNLEEDTKFQFEIPEDDPNHVMHKEMFLENDSNYRDITLNMQFIDFGYCEYGKSSEANKIIIENRMPYR
VEVKWTILDVLNSQGEKTDNPYKVDESVMIIDAKSSANFVIKFRPFEPDYYFFQILQCFVYLLNGNEKKM
RRQEGGGNPQSVMMTKGKITRTSKFEESMYEEIDPPLCLNLRCIGHSFNPGSQPFIPIVKFLPMKNVVFPPC
GPGESKYQTLKIMNTSDTPVYYKMIQDSSKVFRIYPLLGLIPGKSFALVCFEFSPKSANHWSFTSQCVLNY
MFTNVQNIHLSGKCFRPQITLGNKGKLFFPPTYTGVSTEQKLMIKNETRIPLEYECLVPAKHQEIIMFDPPK
GVLQANEERMVTCIFTPLLKKEYSLSIVMQVTNIIDTVKELIGYFNPGSGVAIKAEPKREQTKYELRVFGV
GNDGILSIKPSKLDYDTVTVGFSKILSLVVVNKSKTNLYIDFQLEQMNIENKSQEEQDNIRNILYENFSLDF
NEGIVPALSKKRVKITFRPSLRFDYNIRFTCNARAKPVQELMASIKQNSYLSQKYSINIVAKGDYPLLRFAD
IRNDQMSVSNLWEKFQLTPLNKELLTELRTEEIEYSNSEKTNQSVQDLQ**KN**LRIFNWDFGKVPIKYNHKP
RKVTLTMKNIGGVETEWVFKLPNDSEVELEPWADPGEPTPEQAFEQHIIDNSIFMIEPRTGSLKPGEQTDV
NVYYVSKEVDFHHLKVILHISHGKPIILHFKGETLSRRAHLRLCKDEFHIPPVPVGLEWSVTYPIEIKNLGIT
KLKYKVDLDNLEKLNNENQSFRVFDIENPEGVLMPNEVQYLYTAFRPLESKQYFVNLPIKVSDIEGIAQNI
PLKLNGVGYQGETNKPNEVQFYEDLPKCRAHCRNEDELQAAFSIEELDFGEVDPEVSSIRMVILYNMSPT
QKLTFEFFMTGLLCTDNINLMPISGEVEPNSH**KN**IKLVLKAAVIPTIFDGEIMCQIEWEDQGNKGNAQTINE
THTGTKSVVTHASDNEFLFIRLK

>CUFF.23530.1 sec23 sec24 trunk domain containing protein
MINYYGDPYFDAEPIPTISYGTNPIVRCRDCKAYVNPFIRFTDGGTRWICNFCGVYNNVDNYYYAPLTNG
VRNDIEERKELQYGSVDFLASADYMNRPPMAPTFLFAFDVSKNAIDSGYLSIITASILKAIDSDAIPGGERT

MIGFLTFDDKVHYYNLKSTLKQPQMIVNIDEDPDFLPISEDLIVNLTDSKELVIELLRQLPVMFSDSSEYEA
NINNAVKSIGILTKVTGAKAFLFDESPLFTKYPYFQVTQKPGVKDRPDLIKPTSPILKNYSVELSHYYVSID
YYVISNHNTFNNNATLSELSRYTNGRFYYYPKFNSHLHSTKLDTEFYTSLTAKSAWEACGRIRVSGGYRQ
TSILGNYLVKARTNDLLSFPVCDEHRVIFYELEKVDTPEDAKIKRMQEMPTDVETHIFVQTALLYTSSEGE
RRIRVHNLAMPLTDIASDPFENCDVNALCTLVFKKGIDNVEMLNSSFLGTRGYIEMQFSNMICSVQRMYR
NNLPESIDYIIGYCMGILKNEVFSPSQSLQANSYIDYLNFIRYQCRHMNCDEIMNIIWPQLFQVNDESLSSQS
LPPLINLNRTCFDSTGIYVIFNTFYVYLWIGNAVDSYFLNLLFNVQSFKEITNIELSEDDIFFGETQESKGWI
QELYAIIQSLRISQLIYPEFKILFEIDNKSEIILKDLMLEDATKGYDFNNIKRQLTSH

>CUFF.16923.1 porphobilinogen deaminase
MEGDITTRKFRVGTRSSDLAKTQTYEVIDLIIKKCQENSINVTQDNFELFEIHNSVGDIDQ**KK**KLFEMGGQ
GVFCKQLQQELLDGSCDIAVHSMKDLPTTPYSPLLTIAAIPPLKPREDVVLFKSTSEYSNLDNLPEGAIIGTS
SLRRICTLGIKYPHLKIENIRGNLNTRLRKLEEGTYDAIVLAKAGVQRLNWEDKIGQTLSKNEFEYAPAQG
SLAVECLLDDKETLKILSFIECQFARRIIEAERMYLKTLEGGCTLPISVNSQIFTCNKDEKVEITEFEGIDTSS
CALRINGRVFDRNNPSDYLEDSVEGDLDNWIEIGQELASKLRDKG

>CUFF.16821.1 zinc-binding dehydrogenase family protein
MDTLDTKSKMKAIYMIDSGKQSTDEGLTQVEYGEINTPVPRYGQVLIRVESVPINPSDIYCMEGKYSETID
FKYPFVPGWEGSGTVVASGGGPMAWYLRGKRVSFSKAEEQHPGRKIKFNLGGTMAQYCVTNAYQCIPL
DNDVSFNQGALFFTNPLTALAFEDIVVNNQVKVVVITAAASQLGRMIIKLFQPHNIEIIAIVRKDDQANYL
KENFKLAYVLNSADDDFLAQFSKLVDELSPSYMFEAISGSLSGKLISRMTKRSKIYLYGLLSKESLSDVDP
MAFIGKGITVEGFYLTDWLEK**KK**SFLFLLNIIRRVKNMIKTSLSSEINCEFDLKDYKKALECYENNMSKGK
VILKPNLIEEEFKSKL

>CUFF.15958.1 DUF3250 domain containing protein
MKPGRKNGNKSQDTRMSLEEIKEERNANHDVEDEDPNIINERNANSAKKSPSKVPRGMKSGSRVNPNRE
LMTQAQRAEKYYELLEENTRLKTHQTELDEDIKKMAARLKRIKELISKERKLAGGILGNEFDKELDLIIDE
NTQLKSEKKKFETLAKSLQAQIKKGGIGKQAGKGYGVGKAADIAKEQDQLIGKLKEQLKHNMKTIESLK
EEILLLRKGKPESEPSREIIQRIQDNDNEIVRLKCSLQEVTSNYEGLNAVLDRC**KK**SNHDLLDEIKIKREEIIN
LRSQLVALEKTGGVVDDLKEQIKEMEEDKVKLEERLNELLTDPFLKRETGTSSHNRIAKLEMNIEEKEKII
RNFKEKMLNYVQQIGDLEAKVQKEQNDSKEIREKYEELKEKYEGTGEMTIDNVQKQLKKLDPSQFRKT
MEDLNYHGSEPLWSMVDYISKDEENIAAEIDLNDPKSLLAEIERLKNSKREIAAELEKCQQMLKLQSNLEE
EKLSLVREEAEQLKIQCKAYNTKIEDLVMQLDLKQKEIAELRKVLGGKATLAHTGLKDIRFDQTMDSISD
FSEITEEEKLGVQENVLDIAVDRAEFHGNTLVQMLGKTKLREESFNTFIAISFYDHDTQTTEICQGFSPNYA
TQFAFRNKFDDFYIEFLDTHTMKIEVYISKVDKPQLIGCADVLLKDLVQLGKMTSSKIINGVGEVMSISNS
DVRIGTIKYKMRLRNDFQQAIKLYYDRKLANARKDDREISKAKIKCISFEVIECKDLSVKGVDAQSLKPFC
YYKFYNFNEHNTIVSAGPNPRFDDIQNYEVAYKPTFIDYLDKSNLEITVFDDSAPIPVEDAKSRLDDRDIIG
FAKVPLQLLSLSKDISGPISILNSKGQHCGVLFIKITVTDPMRVYSGAQGATGLAITTLWERDMIDTICEHIT
KSTRFRDVDTIFDIFSKKKDKITQEDFKDAVMPLKCGFSEREVAMFINSSGLFNSGKKDSIDKKEFLGIFSQ
PLFNAFDRYDKRSRLIVEEETKESKEVPIKTTTDFERDDLASMTLKETGKVFTVTKKDMMDNVNKIKDKI
EKYLRRKNHTIKNFWASLTKKKELSPKRFVKKMLKVEGLLIIEKEAEIFYEFIDKDDNGKIKYSDFAIALK
DININLLLDNFKSKLISNDEAYLEVCDKFEGKKEDINARDTFTMINNS

>CUFF.15473.1 Peptidyl-prolyl cis-trans isomerase

MEKISSDSSYTKHEKYIDIVHCTADGGVIKKMISRGNG**KK**PEKLDEVIVHYKGYLEDGSVFDDSHTKEDG
FKFIIGADHVIKGWDIGVIEMEIGEKAEIIIAPEYGYGKIGNPPKIPGDATLTFVIELLSTHERRPTKWMMND
EERIKVTLKLKEDGNLKFKNKEFKEAEGLYREAISHLNVVQIDNSEIKNLKKTIFLNIAVVCNKSESWKEAI
AAATQSLSYDPDNAKALYLRGIAYRNIEQYDESIYDLKNAA**KK**NPTDKAIRAELQISKNEKKINDKDQQN
AFKKFFNEGVYNEKKSEITKIYDELPFYNPSNPKCFMDIKIGDQELQRIVLELFADIVPITVENFRCLCTGEK
LNKNQNLHYKGSIIHRIIPKFIAQGGDFINSNGMGGCSIYGPFFNDEKIWLPHCTEGLISMANHGPNTNNSQ
FFFTLAKAPWLNGKYTVFGRIIKGISILDELDKIETGANDKPLTSVIIVDCGQIYEEILESELQLDRTCKESDK
EVPEESKR

>CUFF.15418.1 low quality protein: importin-5-like
MESDDISRLIGLLKDVLKPDNTARKAAEQLLLSLKVDNPDGYCYSLFAILSDTNNESQIRVLCAVFLRSAF
LSSLGKDKNMWVKISIENRDYIYTEVLNLISNEEDVQVINALSNLISEMIGSLYELEDQVRCKEPHELCKEL
IDKQNSVNIIAALNIYIGMFDKIAEQMMKYKNDLIKVFQFTIGYSDKEVALLGMKAACKIIISLERKDSSYF
KDILNNIFDLAQKELDEMDEDLLEKILIELKELAGAEPMFFMNNFDYAFSVLEKIMLNKTYEKPTIRILPIEL
LSTIVVRLKTKFKKNMKAVKKIVAAIYNTMRTIDDEISDEWLNPEDGCKIEEEEFSIDPVHVGSKCIDSFIR
ELGATKMKPIVQELIEAQFSSKKSTWQEIHSCLMIIALLGEYMDNINDAEPFVEIAIKYLIHENPKVRYSAIH
TIGQMSTDLQPAFQAQFCDKLLAKLITCLSDRYPRLQAHACASLTNFLEGATDDLIENHIKELCVKLLYVI
ENGNTMCKENGITCLATVAEAAETKFGDYYCDVIKSISKYLTPKIEDIKYYQFQGQLIEAVVIMSVSIGYE
YFKDHADGLIKLLLEIQNKIFDEVRECSESVKSSEHHILQAYLLTAWEKLCYLMGKDFAPYLDEIVPNLLLI
ASLNPKFKTSENEVLIHNDDEQSNLVTSEIDE**KK**SALEMIESFVKELKDKYAKFVRTTSEIIVPLLTYKHSE
SIRKLAANCVQGLMICTIEGSPEDRKDHEEIARDFIKLVWEAVEKENETEILGAQIHAVRDIINEMKTPFLN
QDEVDNACKLCIEMIIKSDRRKAINTDYAADNIDPNDENIDHQDLELMQNEDYNEDEFQIAISEIFGSLFKT
HKIYCKPLAEKLFSEILPQYLDENSNPNKKRFALYILVDMVEHLGYEYIGEQFAEIMNTLEIYSSCEVTALR
QSAVYGIGVALSLNIEIFSKDILKYISLMEEAIKIPLGDQDKEEYETCKDNAISAIGKILKYYEDAINKNSQL
LNIENAYKKLFEYWMSLLPLKLDMPESKIMFDFLADKFETNPDLVVGDGFEKLLNLIKLIGEHLHELYMN
KETIARFGRIINRLKELPDLKETIDQFIDFDLEELARKRIEKAIKQC

>CUFF.10872.1 Vacuolar protein sorting-associated protein 33A
MEEESDFPINFKVIKEYFREQFIKYFNKFEQGFLLMIDPEIYKVFSYVLFPIDKDLKAKIKRHFPMDDKIDDI
LTDEFQPTKSKADLEKLQASATIETIIFILKPKKEIVQKAFMIRKILERTGIIDCNYLFVPRGMKYLSESYLIN
NPEKVEFINMNIIPFERDVLSLEISDSFFHSIQQEDLEYLTQSYEAISRLEKVYGTIKYKFACGSNAVNVLNK
LLSVSAELSKSFSISDNSFYEGYSSEVKGGEIDALILIDRKVDLITPFCIQQTYEGMLDEYFGIHATNLEAKR
SIIKGDDEEEKKAALAGEPPKTETMSLRSDQDLIIDELRDLHFVALESKFSQRVIDIDRIIKEKDNPKNVEDL
QKYIEKLKNMKITIVKDKLTSHINLAHHINTLINNFDYSDCLNLEQSIIYGESTKEMIDNLQLMMAKGVDQ
DNILRLIALITITNSGIKDKIYQELFQQYIECYGFEEMNTLLNMEEMGLFR**KK**QGKYDWPRIMKEFLIINEE
TQLKNPVDYSYVYNGYSPLSVKVVEYLMNSKGFGAIESKLKYITNKFKYPSNEREFFEKRATYSSSGKKI
VLVFYIGGCTYAEISAVRFLNKMFTDKVFVIATTQIINYKKCMNQMRKYI

>CUFF.10501.1 hypothetical protein H257_03463
MEGEESKDKDIQEINYHWPALESDPQIFTEYLLKLGMSDEWHLCEVFGLDDDCLSFVPQPCLGAIVAFDR
KTREDEKPGLGENIEIVPFYMKQTGTLDNACGIIACLHAIMNHLGEISLFDESILDRFAKETNIMTPAERAK
YLEEFKEFKEEHKHHSSKGQTEVPTSS**KK**VNHHFVAFIRNENGKLIELDGTKDGPAVIEEECEDLLKGVA
KELQRRLENQIITESLSLMGLAMRPY
